# Impact of Culture Duration on the Properties and Functionality of Yeast-Derived Extracellular Vesicles

**DOI:** 10.34133/bmr.0201

**Published:** 2025-05-06

**Authors:** Gyeongchan Jeon, Yang-Hoon Kim, Jiho Min

**Affiliations:** ^1^ Graduate School of Semiconductor and Chemical Engineering, Jeonbuk National University, Jeonju 54896, Republic of Korea.; ^2^ Biological Resource Center, Korea Research Institute of Bioscience and Biotechnology (KRIBB), Jeongeup 56212, Republic of Korea.; ^3^ Department of Microbiology, Chungbuk National University, Cheongju 28644, Republic of Korea.

## Abstract

Extracellular vesicles (EVs), lipid bilayer nanovesicles secreted by cells, carry nucleic acids, proteins, and other bioactive molecules that influence recipient cells and modulate various biological processes. This study investigated how energy depletion and fermentation processes influence the characteristics and physiological functions of EVs secreted by *Saccharomyces cerevisiae*. Specifically, we analyzed EVs derived from 24-h cultures, representing the glucose utilization phase, and 72-h cultures, representing the starvation stage. Under energy-depleted conditions (72-h cultures), yeast secreted a higher number of EV particles, albeit with a smaller average particle size. In contrast, EVs from yeast cultured for 24 h, during the glucose utilization phase, were enriched in Pep12-rich endosome-derived vesicles and exhibited 71% higher cellular internalization efficiency. Proteomic and transcriptomic analyses revealed distinct protein and microRNA profiles between EVs from 24- and 72-h cultures, highlighting their potential roles in tissue regeneration, cell proliferation, and collagen synthesis. As a result, EVs derived from 24-h cultures exhibited a 15% greater effect in promoting collagen synthesis. The differential effects on collagen production may be attributed to the efficiency of endocytosis and the specific protein and microRNA cargo of the EVs. This study emphasizes the functional potential and unique properties of yeast-derived EVs while proposing strategies to modulate EV composition by adjusting the yeast culture duration and the energy source in the medium. Further research is needed to control yeast-produced EV components and to understand their mechanisms of action for effective therapeutic applications.

## Introduction

Extracellular vesicles (EVs) are lipid bilayer nanovesicles ranging from 30 to 1,000 nm in size, derived from almost all cell types. They are valuable in pathogenesis research and disease diagnosis as they preserve the characteristics of host cells [1]. EVs can deliver various bioactive molecules and regulate physiological processes like cell differentiation, proliferation, tissue repair, and immune regulation [2]. Given these characteristics, extensive research focuses on utilizing EVs as drug delivery vehicles and therapeutic agents [3]. Specifically, EVs from mesenchymal stromal/stem cells are actively researched for their immunomodulatory properties and high regenerative capacity in regenerative medicine [4]. EVs derived from mammalian cells, such as stem cells, show great potential as therapeutic agents. However, commercialization faces several challenges, particularly regarding yield and economic feasibility [5]. Leveraging mass-cultivable microorganisms for EV production could address these challenges by improving the cost efficiency and scalability of cell culture and isolation processes. In contrast to mammalian-cell-derived EV studies, research on microbially derived EVs is still in its early stages, with many unknown areas remaining. A marked difference in EV secretion by fungi compared to mammalian cells is the presence of cell walls in fungal strains [6]. Historically, EV research was limited by the assumption that membrane-derived vesicles struggled to cross thick cell walls. Recently, several potential mechanisms for EV movement across cell walls have been postulated [7].

Several studies have demonstrated the benefits of various yeast-derived secretions to host cells. Recent findings indicate that fungus-derived EVs mediate fungal intercellular communication and are internalized by mammalian cells, regulating various immunogenic responses, including the activation of pro- and anti-inflammatory cytokines [8]. Specifically, the probiotic *Saccharomyces cerevisiae* is effective in normalizing immune function, barrier formation, and tissue regeneration [9]. Due to their high biocompatibility and potential as immunoadjuvants, *S. cerevisiae*-derived EVs are likely to have additional valuable functions as therapeutic agents. Previous studies have shown that the beneficial yeast *Saccharomyces* produces metabolites that promote collagen synthesis during fermentation, while the pathogenic yeast *Candida* can deliver toxic substances to human cells via EVs [9]. Based on this, we hypothesize that *Saccharomyces* EVs can be internalized by human cells and enhance collagen synthesis. However, challenges remain in analyzing fungal EVs. While some elements in the EV production pathway, such as endosomal sorting complex required for transport (ESCRT) proteins, have been identified, general markers for EVs have not yet been established [8]. In particular, there has been limited research conducted to distinguish between plasma-membrane-derived microvesicles and endosome-derived exosomes. Unlike exosomes, which are recognized for their high biocompatibility, apoptotic bodies can provoke a robust immunogenic response [10]. Utilizing EVs without a thorough understanding of the components can lead to unforeseen problems.

Controlling EV components is crucial for their medical applications. A recent study revealed that under energy-depleting conditions, such as glucose starvation, cells secrete an increased number of exosomes, enhancing their efficacy in tissue regeneration [11]. This study investigated the effects of energy depletion and the fermentation process on the characteristics and physiological functions of EVs secreted by *S. cerevisiae* by adjusting the culture period. In particular, we investigated the functionality of yeast-derived EVs in fibroblasts, a common cell type found in human organs and widely used as a model for aging and disease studies [12]. Under the culture conditions for *S. cerevisiae* used in this study, we confirmed that the glucose in the medium was fully depleted 24 h after the start of cultivation. Furthermore, protein analysis results clearly indicated autophagy activation in cells experiencing energy depletion at 72 h. Based on these findings, the study utilized EVs derived from *S. cerevisiae* cultured for 24 h (EV@Y_24_) and 72 h (EV@Y_72_). These EVs were analyzed to characterize their properties, protein composition, and potential effects on human cells. Additionally, we proposed Pep12, a target-membrane soluble NSF attachment protein receptor (t-SNARE protein) localized in late endosomes, as a specific marker for endosome-derived EVs [13]. We then compared and analyzed the presence of exosome-like vesicles in each EV sample. Our findings showed that EV@Y_24_ had a larger average vesicle size than EV@Y_72_ and contained more endosome-derived vesicles. Consistent with previous research, EVs were confirmed to be internalized and affect human cells [8]. Notably, EV@Y_24_ exhibited a higher internalization rate than EV@Y_72_, suggesting that endosome-based vesicles containing many SNARE and ESCRT proteins can be easily imported into cells via membrane fusion or endocytosis [13]. Additionally, proteome and transcriptome analyses identified distinct protein and microRNA (miRNA) profiles between EV@Y_24_ and EV@Y_72_. Consequently, yeast-derived EVs likely exert related effects due to the presence of miRNAs that positively regulate the AKT, extracellular signal-regulated kinase (ERK), and Wnt signaling pathways, which are involved in tissue regeneration, cell proliferation, and collagen synthesis. In this study, human fibroblasts were treated with EVs to compare and analyze the effects of the 2 EV populations on collagen synthesis. The differential effects on collagen production may be attributed to the efficiency of endocytosis and the specific protein and miRNA cargo of the EVs. Our findings reveal that the culture period significantly influences the characteristics and composition of EVs.

Ongoing research aims to use yeast as a model organism to understand EV development and provide strategies to control EV composition and function. This study is the first to vary the culture conditions of *S. cerevisiae*-derived EVs, analyze the characteristics and functional differences of EV components, and identify their collagen regulatory function. In conclusion, this study demonstrates that modifying culture conditions can effectively regulate the characteristics and composition of yeast-derived EVs, significantly influencing their functional outcomes. This study is novel in its focus on the variation of EV subgroups, highlighting that yeast-derived EVs, like those from mammalian cells, can originate through endocytosis (exosomes), membrane blebbing (ectosomes or microvesicles), or cell death (apoptotic bodies) [6]. Additionally, yeast-derived EVs have potential as promising materials for regenerative medicine. Research using the nonpathogenic yeast *S. cerevisiae*, a commonly used model to study various aspects of eukaryotic biology, is highly valuable [14]. Further research is needed to properly control the components of yeast-produced EVs and understand the mechanisms by which they affect human cells for their therapeutic application.

## Materials and Methods

### Yeast culture and EV isolation

*S. cerevisiae BJ3501* (ATCC 208280) was purchased from American Type Culture Collection (ATCC, USA). *S. cerevisiae* was grown in YPD medium (1% yeast extract, 2% peptone, and 2% d-glucose). Overnight-cultured yeast was diluted to 0.02 OD_600_ in YPD medium. Then, the cultures were incubated at 30 °C with shaking at 180 rpm. The pH, d-glucose content, and cell growth in the media were evaluated at each specified time. EVs were isolated from the yeast-cultured medium using previous methods [15]. EV@Y_24_ was isolated from 24-h-cultured medium, and EV@Y_72_ was isolated from 72-h-cultured medium. The medium was collected and centrifuged at 1,000 × g for 5 min to remove whole cells and large debris. Then, the supernatant was centrifuged at 13,000 × g for 30 min and the final supernatant was filtered using a 0.2-μm pore size filter. The filtered medium was centrifuged at 100,000 × g for 70 min using an ultracentrifuge (Optima XE, Beckman Coulter, USA) with a 70 Ti rotor. Then, the pellets were washed with phosphate-buffered saline (PBS, pH 7.0) and resuspended in PBS. All of EV isolation procedures were performed at 4 °C.

### Nanoparticle tracking analysis

The particle concentrations and size distributions of EV samples were analyzed with a ZetaView PMX-120 instrument (Particle Metrix, Germany). Before analyzing the samples, the instrument was calibrated with standard 100-nm polystyrene beads (Particle Metrix, Germany). For analysis, 1 ml of EV sample was inserted into the instrument. In this study, all EV samples were suspended in PBS at pH 7.0. Data analysis was performed using the ZetaView software. Data were obtained in triplicate, and the average and standard deviation of particle size and concentration were calculated.

### Bio-transmission electron microscope

Preparations were fixed to transmission electron microscopy grids (Electron Microscopy Sciences, USA) for 10 min. Then, 2% uranyl acetate was added to the grid for staining. After a brief incubation, the grid was left for 15 min to dry. The prepared samples were imaged using a bio-transmission electron microscope (H-7650, Hitachi, Japan) located at the Center of University-Wide Research Facilities at Jeonbuk National University.

### Western blotting

To extract protein in yeast cells, the glass bead lysis method was used [16]. The harvested yeast cells were suspended in lysis buffer (1% SDS, 1% Triton X-100, 50 mM Tris-HCl, pH 7.5, 1 mM EDTA, and 1 mM phenylmethanesulfonyl fluoride [PMSF]) added protease inhibitor cocktail (Cat. No. P8849, Sigma-Aldrich, USA). Then, an amount of glass beads (0.4 to 0.6 mm) (SI.45015, Sigmund Lindner, Germany) equal to the amount of yeast cells was added and was vortexed in a cyclomixer (Vortex-GENIE 2, Scientific Industries, USA) for 5 min. The EV sample was dissolved in lysis buffer (1% Triton X-100, 50 mM Tris-HCl, pH 7.5, 1 mM EDTA, and 10 and 1mM PMSF) added protease inhibitor cocktail. For collagen-related protein analysis, the Hs27 cells were detached using a scraper and harvested. Protein extraction for western blot analysis was performed using lysis buffer (1% Triton X-100, 50 mM Tris-HCl, pH 7.5, 1 mM EDTA, 10 mM NaF, 2 mM Na_3_VO_4_, and 1mM PMSF) added protease inhibitor cocktail. After lysis, the protein concentration was determined by Bradford assay, and 20 μg of protein was used to prepare the samples for western blotting. The primary antibodies used in this study were as follows: rabbit anti-COL1A1 (collagen, type 1, alpha 1) antibody (#72026, Cell Signaling, USA), mouse anti-COL3A1 (collagen, type 3, alpha 1) antibody (sc-271249, Santa Cruz, USA), rabbit anti-matrix metalloproteinase-1 (anti-MMP1) antibody (ab52631, Abcam, UK), rabbit anti-glyceraldehyde-3-phosphate dehydrogenase (anti-GAPDH) antibody (#5174, Cell Signaling, USA), rabbit anti-autophagy-related protein 8 (anti-Atg8) antibody (ab4753, Abcam, UK), mouse anti-cytochrome c oxidase subunit 4 (anti-Cox4) antibody (ab110272, Abcam, UK), mouse anti-nuclear pore complex protein (anti-NSP1) antibody (ab4641, Abcam, UK), mouse anti-protein disulfide isomerase (anti-PDI1) antibody (ab4644, Abcam, UK), mouse anti-proteinase A vacuolar sorting protein 12 (anti-Pep12) antibody (ab113689, Abcam, UK), mouse anti-alkaline phosphatase (anti-PHO8) antibody (ab113688, Abcam, UK), mouse anti-vacuolar membrane ATPase subunit B (anti-VMA2) antibody (ab113684, Abcam, UK), and mouse anti-GAPDH antibody (ab125247, Abcam, UK). The secondary antibodies used in this study were horseradish peroxidase-conjugated goat anti-mouse immunoglobulin G (H+L) antibody (CSB-PA573747, Cusabio, USA) and horseradish peroxidase-conjugated goat anti-rabbit immunoglobulin G (H+L) antibody (CSB-PA564648, Cusabio, USA).

### Cellular uptake assay

Labeling exosomes with lipophilic dyes like 3,3′-dioctadecyloxacarbocyanine perchlorate (DiO), 1,1′-dioctadecyl-3,3,3′,3′-tetramethylindocarbocyanine perchlorate (DiI), or PKH dyes for detecting cellular internalization has been widely employed in numerous studies [17]. The EVs were labeled using Vybrant DiO lipophilic dye (V22886, Invitrogen, USA). The extracted exosomes were incubated with a lipophilic DiO tracer solution at 37 °C for 20 min. Excess DiO was removed through 2 successive washing steps, after which particle concentration was measured using nanoparticle tracking analysis equipment. Hs27 cells were seeded onto glass coverslips placed in 6-well plates at 3 × 10^5^ cells/well and grown overnight. Then, the labeled EV samples were treated at a concentration of 10^9^ particles/ml. Following a 4-h incubation at 37 °C, the cells were centrifuged at 500 × g for 5 min, washed, and resuspended in 1× PBS. The prepared cells were then analyzed using an Attune NxT flow cytometer (Thermo Fisher, USA), and yeast-derived EV uptake was observed.

Hs27 cells were seeded onto glass coverslips placed in 12-well plates at 10^5^ cells/well and grown overnight. Then, the labeled EV samples were treated at a concentration of 10^9^ particles/ml. After 4 h, the cells were fixed with 4% paraformaldehyde for 10 min at room temperature and cells were labeled with 4′,6-diamidino-2-phenylindole (DAPI; D9542, Sigma-Aldrich, USA). After DAPI staining, the cells were washed with PBS 3 times. To capture the fluorescence images, a Zeiss Axioscope A1 fluorescence microscope (Carl Zeiss, Germany) was utilized. The number of EV particles in Hs27 cells was counted.

### Proteome analysis

The proteome analysis was performed through liquid chromatography–tandem mass spectrometry (LC–MS/MS) measurements. All analyses were entirely managed by the Korea Basic Science Institute (South Korea). For proteomic sample preparation, yeast-derived EVs were lysed using lysis buffer and the lysate was centrifuged at 13,000 × g for 10 min at 4 °C. The protein concentration was determined by Bradford analysis. LC–MS/MS was performed by a nanoACQUITY UPLC System (Waters Corp., UK) and SYNAPT G2-Si High Definition Mass Spectrometry (Waters Corp., UK). The peptides were dissolved in 0.1% (v/v) formic acid solution and delivered to a column (TRIZAIC nanoTile 1.7 μm C18, Waters Corp., UK) and then separated at a flow rate of 400 μl/min in 0.1% (v/v) formic acid in acetonitrile. Then, the solution was sprayed via electrospray ionization at a spray voltage of 2.5 kV, a cone voltage of 40 V, and a source temperature of 120 °C. The mass spectral data were acquired in the MassLynx software (Bruker Daltonics, Germany), and protein identification and quantification were performed using Progenesis QI for Proteomics (Nonlinear Dynamics, UK.) In addition, Gene Ontology (GO) terms were searched against the UniProt *S. cerevisiae* database.

### Transcriptome analysis

The transcriptome profiling of yeast-derived EVs was performed through an Illumina NextSeq 500 sequencing system (Illumina, USA). RNA purification and analysis were entirely managed by eGnome Corporation (South Korea). The EV samples were separated, and TRIzol reagent was added. Then, TruSeq Small RNA Library Preparation Kits (Illumina, USA) were used for proteomic sample preparation. The quality of the samples was evaluated using Quant-it RiboGreen RNA Assay Kit (Invitrogen, USA). The small RNA sequencing was performed for 75 cycles on the Illumina NextSeq 500 sequencing system following the manufacturer’s instructions. The reference genome indexes were constructed using Bowtie (version 1.3.1). The quality of the raw data was assessed using FastQC. Low-quality sequences were removed and filtered using Trimmomatic. The miRNA expression levels were normalized by count per million. Differential expression analysis of 2 conditions was performed using the DEGseq R package (version 3.36.0). After visualizing the volcano plot, 66 differentially expressed small RNAs were selected based on a *P* value <0.05 and |log_2_(fold change)| > 2. Human messenger RNA (mRNA) target prediction of the identified yeast EV-derived miRNA sequences was performed using psRNATarget (https://www.zhaolab.org/psRNATarget/), a miRNA target prediction tool. To identify the significantly enriched signal pathways in the dataset, GO and Kyoto Encyclopedia of Genes and Genomes pathways were categorized using DAVID Bioinformatics Resources (version 6.8) (https://davidbioinformatics.nih.gov/).

### Fibroblast culture and collagen analysis

Hs27 fibroblast cells (CRL-1634) were purchased from ATCC (USA). Hs27 cells were grown in Dulbecco modified Eagle medium containing 10% fetal bovine serum and penicillin. The Hs27 cells were cultured in a 100-mm cell culture dish until they reached 90% cell confluency. After starvation for 12 h, retinol (R7632, Sigma-Aldrich, USA) or EV samples were treated for 24 h. The content of procollagen type 1 in the cultured medium was analyzed using Procollagen Type I C-peptide EIA Kit (MK101, Takara, Japan) according to the manufacturer’s instructions. In addition, western blotting and real-time polymerase chain reaction (RT-PCR) were conducted to determine the expression of protein or mRNA related to collagen synthesis.

### Real-time polymerase chain reaction

Total RNA was extracted from Hs27 cells using the GeneAll Ribospin II kit (GeneAll Biotechnology, South Korea) according to the manufacturer’s instructions. All primers were synthesized by Bioneer Corp. (South Korea). The primers used for RT-PCR are listed in Table 1. RT-PCR analysis was performed using the CFX96 real-time PCR detection system (Bio-Rad, USA). The CFX Maestro software (Bio-Rad, USA) was used for analysis. The RT-PCR results were calculated using the $2^{-\Delta \Delta C_{T}}$ method [18].

**Table 1.** Primer sequences for RT-PCR

Gene	Forward primer sequence	Reverse primer sequence
COL1A1	5′-TTCTGCAACATGGAGACTGG-3′	5′-CGCCATACTCGAACTGGAATC-3′
COL3A1	5′-TGGTCTGCAAGGAATGCCTGGA-3′	5′-TCTTTCCCTGGGACACCATCAG-3′
Elastin	5′-AAAGTTCCTGGTGTCGGTCTTCCA-3′	5′-AGCAGCTCCATACTTAGCAGCCTT-3′
MMP1	5′-TGCTGCTGCTGCTGTTCTGGG-3′	5′-GGCCGATGGGCTGGACAGGA-3′
TIMP1	5′-CTCGTCATCAGGGCCAAGTT-3′	5′-GTAGGTCTTGGTGAAGCCCC-3′
GAPDH	5′-GAGAAGGCTGGGGCTCATTT-3′	5′-CACAATGCCGAAGTGGTCGT-3′

RT-PCR, real-time polymerase chain reaction

## Results

### Characterization of *S. cerevisiae*-derived EVs

**Fig. 1.**
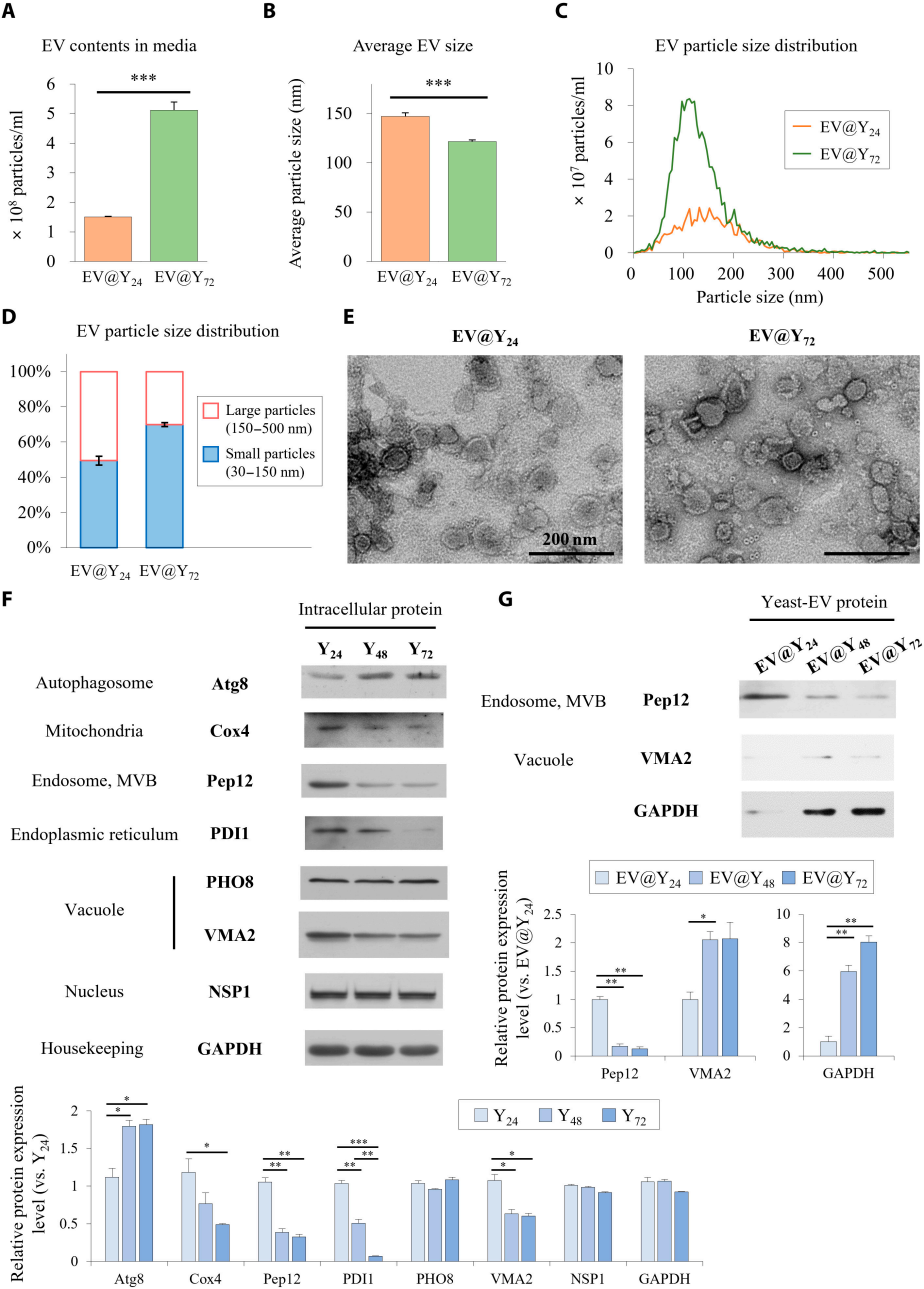
EV@Y_24_ and EV@Y_72_ were characterized, and the characteristics of yeast and yeast-derived extracellular vesicles (EVs) during each culture period were analyzed by western blotting. Particle size analysis was conducted by a ZetaView PMX-120 nanoparticle tracking analysis (NTA) instrument, and the instrument was calibrated with standard 100-nm polystyrene beads. (A) The contents of EV particles, (B) average particle size, and (C) size distribution were evaluated in EV samples. (D) The ratio of small particles (30 to 150 nm) and large particles (150 to 500 nm) was confirmed in each isolated EV sample. Data are presented as mean ± SD (*n* = 3). *P < 0.05, **P < 0.01, and ***P < 0.001. Yeast cells were cultured in YPD medium (1% yeast extract, 2% peptone, and 2% d-glucose) and harvested daily until 72 h. (E) EV@Y_24_ and EV@Y_72_ were imaged using a bio-transmission electron microscope. (F) In yeast cell samples, the expression of an autophagosome marker (Atg8), a nucleus marker (NSP1), a mitochondria marker (Cox4), vacuole markers (PHO8 and VMA2), a multivesicular body (MVB) marker (Pep12), and an endoplasmic reticulum (ER) marker (PDI1) was confirmed. (G) EVs were isolated from yeast-cultured medium and the expression of Pep12, VMA2, and glyceraldehyde-3-phosphate dehydrogenase (GAPDH) was confirmed. Y_24_, 24-h-cultured yeast; Y_48_, 48-h-cultured yeast; Y_72_, 72-h-cultured yeast; EV@Y_24_, EVs from 24-h-cultured medium; EV@Y_48_, EVs from 48-h-cultured medium; EV@Y_72_, EVs from 72-h-cultured medium. Atg8, autophagy-related protein 8; Cox4, cytochrome c oxidase subunit 4; NSP1, nuclear pore complex protein; PDI1, protein disulfide isomerase; Pep12, proteinase A vacuolar sorting protein 12; PHO8, alkaline phosphatase; VMA2, vacuolar membrane ATPase subunit B.

EV samples were isolated from *S. cerevisiae* cultured in YPD medium. EV@Y_24_ was isolated from medium cultured for 24 h, and EV@Y_72_ was isolated from medium cultured for 72 h. Analysis of yeast growth and d-glucose levels over time indicated that at 24 h, d-glucose was depleted, marking the beginning of the stationary phase (Fig. S1). This 24-h period is generally considered an appropriate culture duration. In contrast, 72 h represents a long-term culture period, indicating the stage where glucose metabolites are fully decomposed due to the depletion of the energy source and an extended fermentation process. The EV samples were characterized using nanoparticle tracking analysis and compared to each other (Fig. 1). The average particle size and size distribution are similar to those previously reported for EV characteristics [6]. EV@Y_72_ contained approximately 3 times more particles than EV@Y_24_, and the average particle size was smaller (Fig. 1A and B). Analysis of the particle size distribution confirmed that EV@Y_24_ had 50% small particles (30 to 150 nm) and 50% large particles (150 to 500 nm), whereas EV@Y_72_ had 70% small particles and 30% large particles (Fig. 1C and D). Additionally, to confirm the characteristics of EVs secreted by yeast at 24 and 72 h of culture, yeast from each culture period was placed in EV isolation buffer, and EVs released over 1 h were collected and analyzed (Fig. S2). The detailed experimental method is illustrated in Fig. S2A. As a result, yeast cultured for 72 h released more EVs compared to yeast cultured for 24 h. This suggests that the significantly higher EV content in EV@Y_72_ is not solely due to the longer culture period. However, unlike the results shown in Fig. 1B, the average particle size of the 2 EV samples was similar, at 160 nm. Additionally, the zeta potential of EVs released by yeast cultured for 72 h was higher than that of EVs released by yeast cultured for 24 h. Zeta potential serves as an indicator of colloidal stability, influencing surface chemistry and bioconjugation [19].

### EV@Y_24_ is enriched in the late-endosome protein Pep12

The characteristics of yeast and yeast-derived EVs during each culture period were analyzed using western blotting. *S. cerevisiae* was cultured in YPD medium, and yeast samples were collected after 24, 48, and 72 h (designated as Y_24_, Y_48_, and Y_72_, respectively). EV samples were isolated from the corresponding 24-, 48-, and 72-h yeast culture media (EV@Y_24_, EV@Y_48_, and EV@Y_72_, respectively). According to the culture method used in this study, it was confirmed that d-glucose was depleted at 24 h, indicating that the starvation process had begun (Fig. S1).

Figure 1F shows the expression levels of specific organelle-related proteins in cells over different culture periods. The expression of the autophagy-related protein Atg8 increased with longer culture periods. Autophagy is crucial for cell survival during nutrient depletion, making it a key response to starvation [20]. Atg proteins are essential for autophagy, with Atg8 playing a significant role in cargo recognition during various autophagy processes [20]. Previous studies have shown that Atg8 exhibits the greatest change in synthesis upon autophagy induction in yeast [21]. Additionally, proteins located in the mitochondria (Cox4), endosomes (Pep12), endoplasmic reticulum (ER) (PDI1), and vacuole (VMA2) were down-regulated. After nutrient depletion, 3 types of macroautophagy (mass autophagy, ER-phagy, and mitophagy) were induced, leading to the degradation of cytoplasmic components, ERs, and mitochondria [20]. These findings indicate that autophagy due to energy depletion was actively progressing in Y72. Figure 1G shows the expression levels of specific proteins, Pep12, VMA2, and GAPDH, in EVs across different culture periods. EVs are categorized into exosomes, which are formed in intracellular endosomal compartments, and microvesicles, which are shed from the cell surface [1]. The ESCRT is a peripheral membrane protein complex involved in diverse biological processes, such as cell division, autophagy, and retrovirus budding, and it plays a key role in the formation and release of exosomes [14]. Pep12 is a SNARE protein that interacts with ESCRT components and is essential for membrane fusion in the secretory and endocytic pathways [13,22]. It specifically localizes to late endosomes and multivesicular bodies (MVBs) [13]. As the culture period increased, Pep12 expression decreased, while VMA2 and GAPDH expression increased in isolated EVs. This suggests that endosome-related EVs, such as exosomes, were enriched in EV@Y_24_ but not in EV@Y_72_, while EV@Y_72_ contained more proteins from various organelles, such as VMA2 and GAPDH.

### EV@Y24 is internalized into cells more effectively than EV@Y_72_

**Fig. 2.**
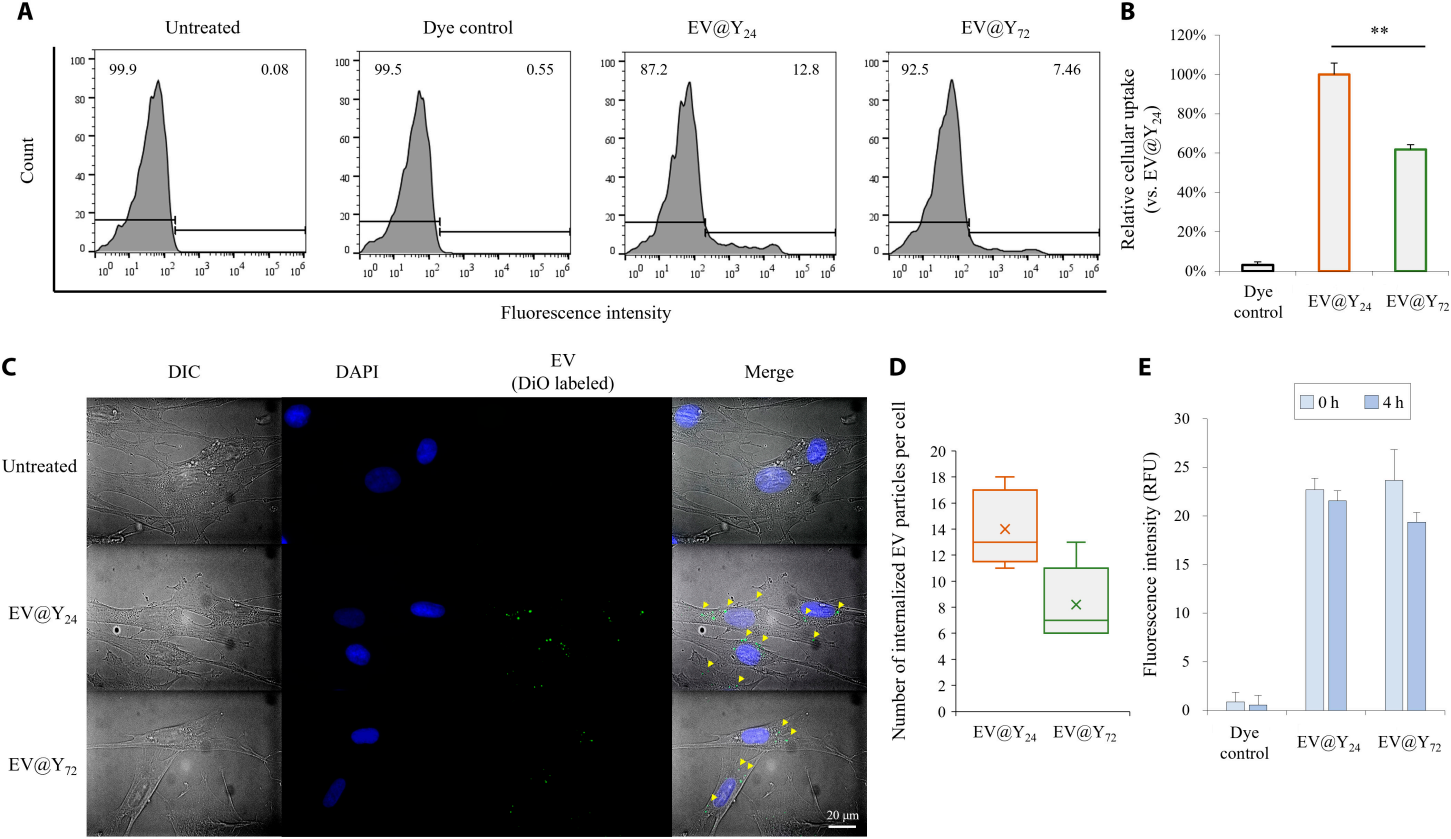
Fluorescence labeling and cellular uptake analysis of EV@Y_24_ and EV@Y_72_. DiO-labeled EVs were applied to Hs27 fibroblast cells at a concentration of 10^9^ particles/ml. After 4 h, cellular uptake was assessed by flow cytometry. (A) The cellular uptake of EV@Y_24_ and EV@Y_72_ is presented as histogram plots, and (B) cellular uptake is quantified as a percentage relative to that of the EV@Y_24_-treated group. (C) Fluorescence imaging of the uptake of DiO-labeled EVs (yellow arrows) by Hs27 cells after 4-h incubation. (D) The number of EV particles per cell was counted and is visualized as a box plot (*n* = 10). The white dashed lines indicate cell boundaries. (E) To assess dye stability, DiO-labeled EVs were incubated at 37 °C for 4 h, and their fluorescence intensity was measured and compared before and after incubation. Data are presented as mean ± SD (*n* = 3). **P < 0.01. DIC, differential interference contrast image; DAPI, 4′,6-diamidino-2-phenylindole; DiO, 3,3′-dioctadecyloxacarbocyanine perchlorate.

EVs can mediate communication between cells by delivering bioactive molecules and can enter target cells through fusion or endocytosis [23]. EVs that reach target cells can affect them by directly interacting with plasma membrane (PM) receptors, fusing with the membrane, or entering through various endocytosis pathways (phagocytosis, macropinocytosis, clathrin-mediated endocytosis, caveolin-mediated endocytosis, and clathrin/caveolin-independent or lipid raft-mediated endocytosis) [24]. In this study, we compared the endocytosis efficiency of each EV sample targeting fibroblasts. EVs were labeled with DiO lipophilic dye, commonly used to track liposomes or vesicles composed of lipid bilayers [17]. Hs27 cells were incubated with DiO-labeled EVs for 4 h, and cellular uptake was assessed by flow cytometry (Fig. 2A and B). The results showed that both EV@Y_24_ and EV@Y_72_ were successfully internalized by Hs27 cells, with EV@Y_24_ exhibiting 71% higher uptake efficiency than EV@Y_72_. No increase in fluorescence was observed in the dye control group, which underwent the same staining and removal procedures as the DiO-labeled EVs. Additionally, fluorescence microscopy analysis confirmed the localization of EVs (Fig. 2C and D). There was no significant change in the fluorescence intensity of DiO-labeled EVs at a concentration of 10^9^/ml before and after 4 h of incubation at 37 °C (Fig. 2E).

### Proteome analysis of EV@Y_24_ and EV@Y_72_

**Fig. 3.**
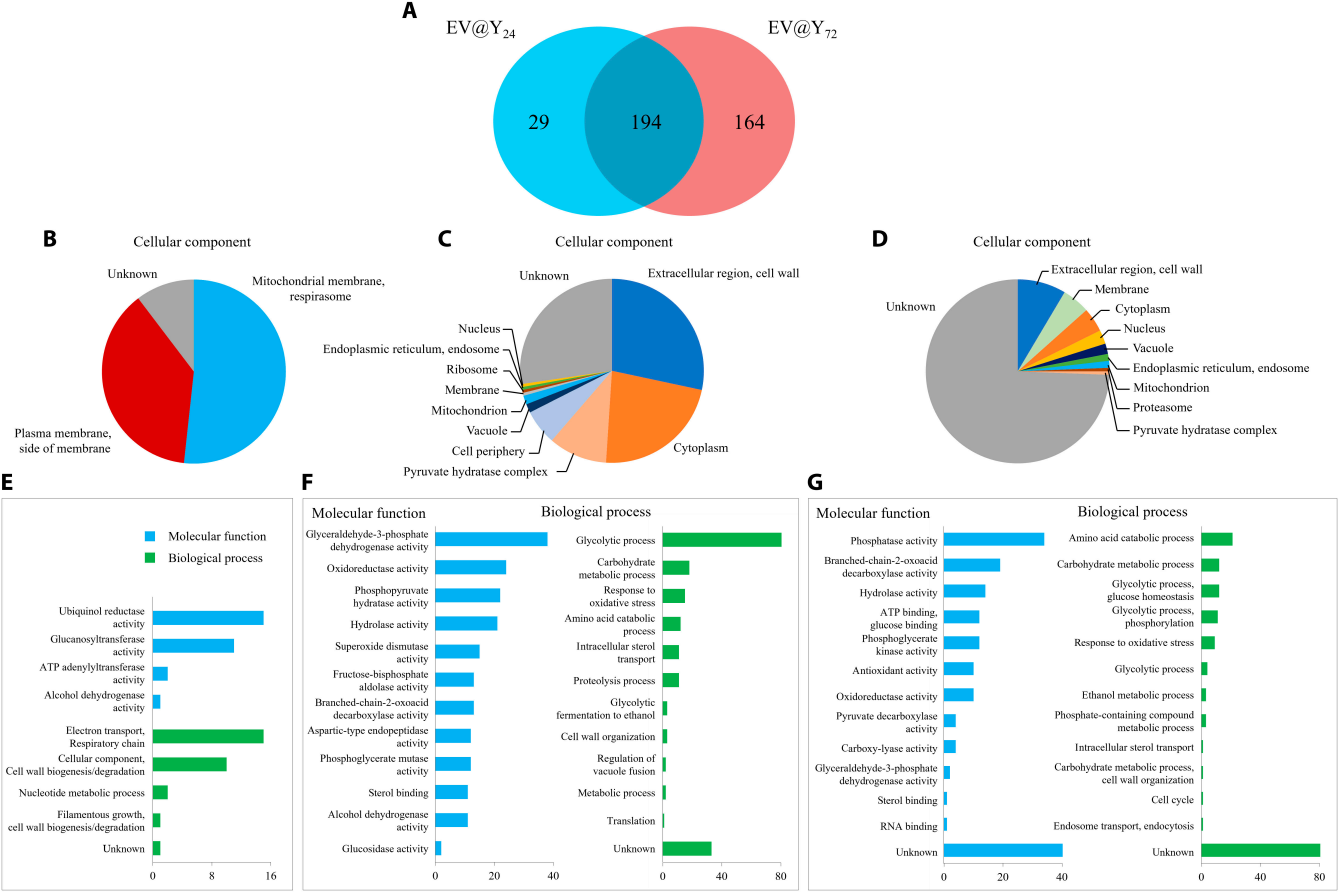
Protein qualitative analysis of EV@Y_24_ and EV@Y_72_. (A) Venn diagram of identified proteins in EV@Y_24_ and EV@Y_72_. (B and E) Gene Ontology (GO) analysis of 29 proteins present only in EV@Y_24_. (C and F) GO analysis of 194 commonly existing proteins. (D and G) GO analysis of 164 proteins present only in EV@Y_72_. ATP, adenosine triphosphate.

To compare and analyze the specific characteristics of proteins present in EV@Y_24_ and EV@Y_72_, proteome analysis using LC/MS was conducted. By protein identification, 223 proteins were identified in EV@Y_24_ and 358 proteins were identified in EV@Y_72_ (Fig. 3). The total data of identified proteins are provided in the Supplementary Materials (Tables S1 and S2). Figure 3A shows that 194 proteins are common to both EV samples, 29 proteins are present only in EV@Y_24_, and 164 proteins are present only in EV@Y_72_. Figure 3B and E show GO term analysis of 29 proteins that exist only in EV@Y_24_, Fig. 3C and F show the GO term analysis of the 194 proteins common to both EV samples, and Fig. 3D and G show the GO term analysis of the 164 proteins present only in EV@Y_72_. As a result, 15 cytochrome b-c1 complex subunit Rieske related proteins, a component of the ubiquinol-cytochrome c oxidoreductase related to mitochondrial electron transport, were identified. Also, 11 1,3-beta-glucanosyltransferases were identified. As glycosylphosphatidylinositol-anchored (GPI-anchored) proteins, glucanosyltransferases have been widely studied for their roles in ER- to Golgi vesicle-mediated transport and lipid raft-mediated endocytosis [25]. They may be one of the factors influencing the high cellular uptake of EV@Y_24_. Glucanosyltransferases may reside in the PM, but their complete inclusion as a component of EVs needs to be approached cautiously, as they have recently been reported to be covalently attached to cell wall glucans [26]. Additionally, as expected, EV@Y_72_ contained many proteins from various cell organelles.

**Fig. 4.**
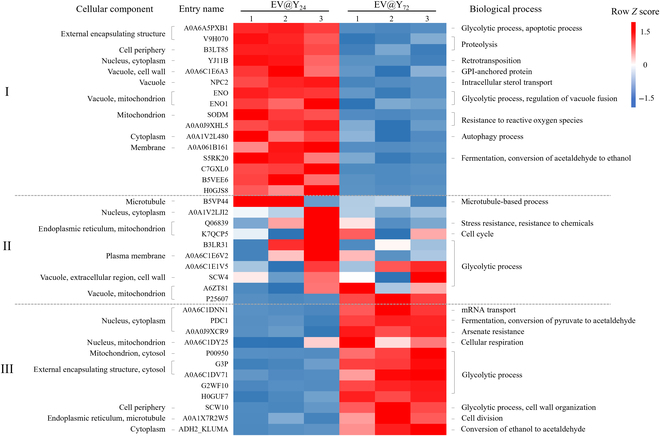
Protein relative analysis of EV@Y_24_ and EV@Y_72_. The heat map of the shared proteins among EV@Y_24_ and EV@Y_72_ was visualized, and GO analysis was performed. Zone I contains a collection of proteins enriched in EV@Y_24_ samples (|*Z* score| > 0.9). Zone II is a collection of proteins whose abundances do not differ significantly in the 2 samples. Zone III contains a collection of proteins enriched in EV@Y_72_ samples (|*Z* score| > 0.9). GPI, glycosylphosphatidylinositol; mRNA, messenger RNA.

Figure 4 shows comparative proteomic analysis. For comparative proteomic analysis, 38 proteins that were significantly common to the 2 samples were considered. Yeast prefers to utilize fermentable sugars like glucose or fructose over nonfermentable carbon sources such as glycerol or ethanol [27]. However, when glucose and other carbon sources are depleted, yeast switches to using alternative carbon sources, such as ethanol [28]. EV@Y_24_ contained numerous enzymes that reduce acetaldehyde to ethanol, whereas EV@Y_72_ contained many enzymes that convert ethanol to acetaldehyde. These findings suggest that yeast EVs maintain the metabolic state of their host cells. Additionally, EV@Y_24_ contains numerous GPI-anchored proteins, which may facilitate cell internalization.

### Transcriptome analysis of EV@Y_24_ and EV@Y_72_

**Fig. 5.**
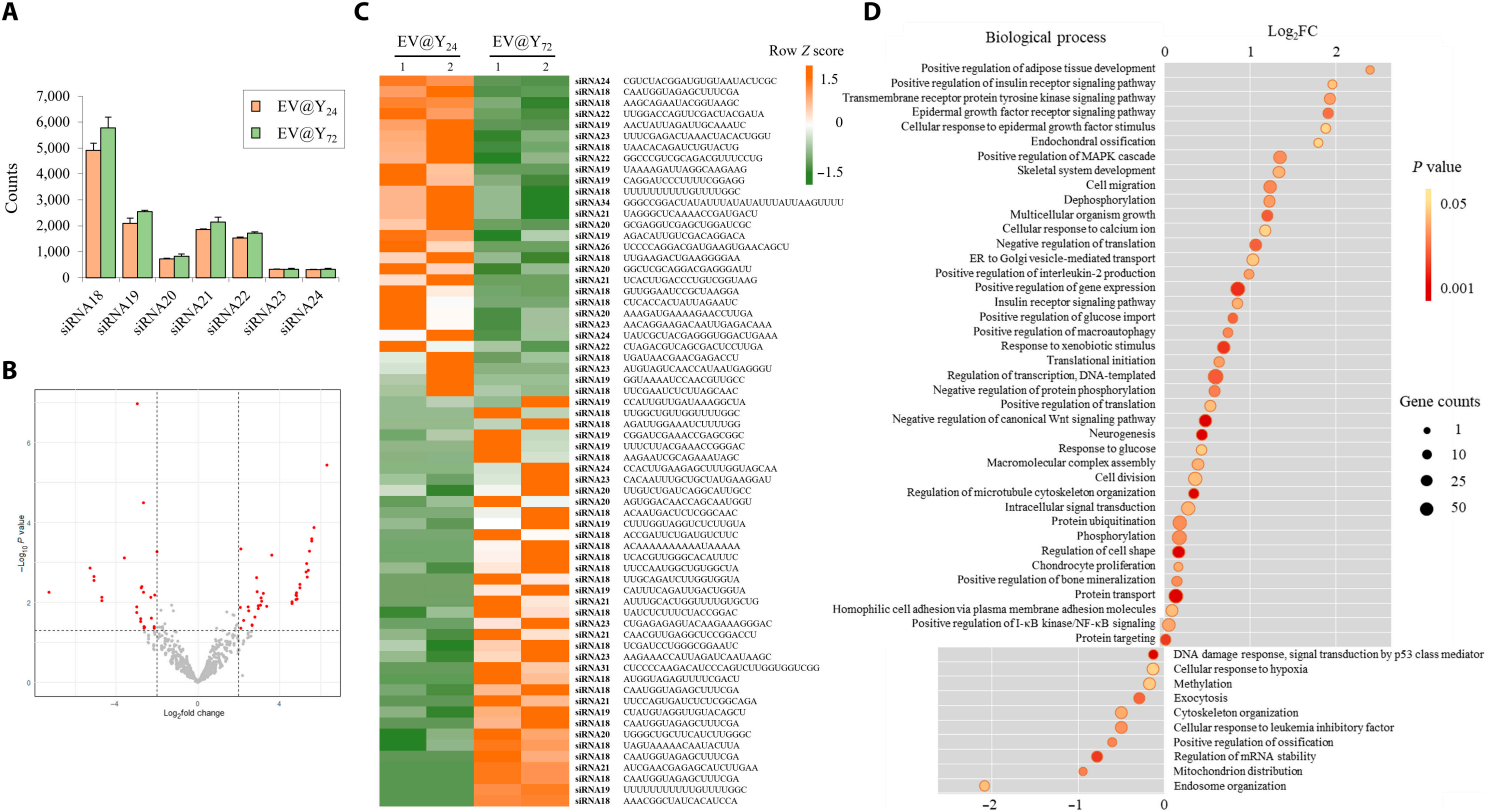
Small RNA sequencing in EV@Y_24_ and EV@Y_72_. (A) Small RNA length distribution of read counts from Illumina sequencing in EV samples. (B) A volcano plot was visualized, and 66 differentially expressed small RNAs (red spots) were selected based on a *P* value <0.05 and |log_2_(fold change)| > 2. (C) The heat map of the shared small RNAs among EV@Y_24_ and EV@Y_72_ is visualized. (D) The Kyoto Encyclopedia of Genes and Genomes (KEGG) analysis of the target genes of the identified small RNAs in EV@Y_24_ and EV@Y_72_. In the graph, a higher logFC indicates stronger suppression by EV@Y_72_, while a lower logFC suggests stronger suppression by EV@Y_24_. Circle size represents the number of mRNAs involved in each biological process targeted by microRNAs (miRNAs). Pathways with *P* values >0.05 were excluded. A lower *P* value indicates significant enrichment of the targeted genes in a biological process compared to random chance. Human mRNA target prediction of the identified yeast EV-derived miRNA sequences was performed using the psRNATarget tool. The GO terms and KEGG pathway of the target mRNA were categorized using DAVID Bioinformatics Resources. FC, fold change; MAPK, mitogen-activated protein kinase; NF-κB, nuclear factor-κB.

miRNAs are released in EVs, including exosomes and microvesicles. miRNAs are small noncoding RNAs (18 to 24 nucleotides [nt]) that can cause posttranscriptional gene repression by inhibiting the translation of target-specific mRNA or inducing mRNA degradation [29]. Figure S3 shows that most of the RNAs present in yeast-derived EV are small RNAs smaller than 25 nt. The EV samples were loaded onto an Illumina NextSeq 500 instrument, with a 75-cycle kit, to obtain single-end 75-nt reads. As a result, 1,146 different sequences of miRNAs were detected in EV@Y_24_, and 1,158 different sequences of miRNAs were detected in EV@Y_72_ (Table S3). In contrast to the proteome analysis, the sequences of identified miRNAs were not significantly different between the 2 groups, with 1,140 miRNAs common to both samples. In both groups, 99% of the read sequences were 18 to 24 nt, with the most abundant sequences being 18 nt in length (Fig. 5A). To identify biomarkers with high confidence in the 2 groups, a volcano plot was visualized and 66 differentially expressed small RNAs were selected based on a *P* value <0.05 and |log_2_(fold change)| > 2. A heat map of the 66 small RNAs was also visualized (Fig. 5B and C). To confirm the effect of the 2 groups of EVs on human cells, prediction of target genes and functional analysis was performed. Human mRNA target prediction of the identified yeast EV-derived miRNA sequences was performed using psRNATarget, and the biological pathways of the target mRNAs were categorized using DAVID Bioinformatics Resources (Table S4). Duplicate categories were removed from the dataset, and 50 biological pathways were diagrammed (Fig. 5D). Here, we present a biological pathway that can be inhibited by small RNAs present in EV@Y_24_ and EV@Y_72_. As a result, EV@Y_72_ is likely to be effective in suppressing obesity by targeting obesity-related processes better than EV@Y_24_, including positive regulation of adipose tissue development and positive regulation of the insulin receptor signaling pathway. There is also a possibility of influencing collagen synthesis by targeting processes related to epidermal growth factor, such as the epidermal growth factor receptor signaling pathway and cellular response to epidermal growth factor stimulus.

### EV@Y_24_ and EV@Y_72_ promote collagen synthesis in Hs27 fibroblast cells

**Fig. 6.**
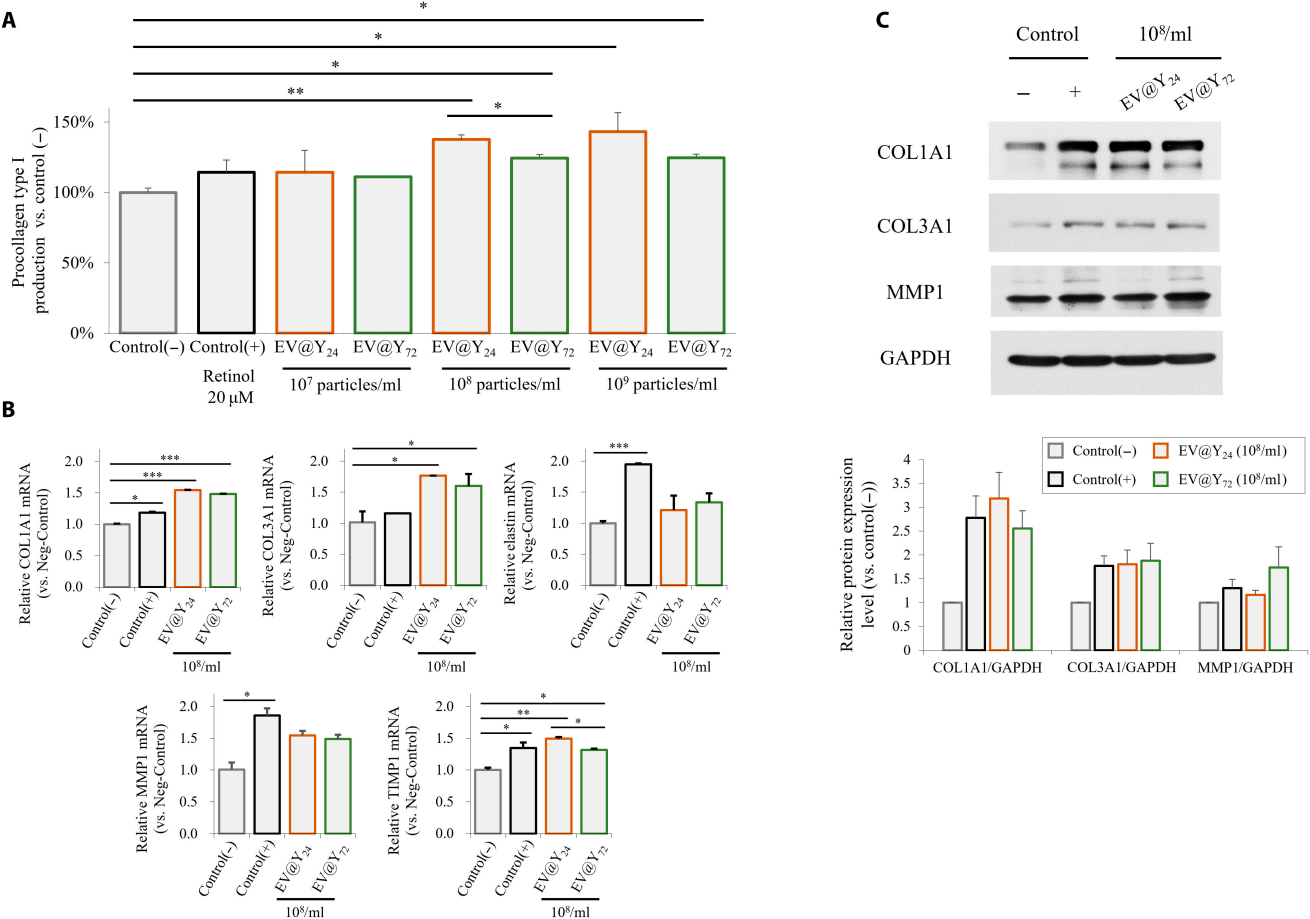
EV@Y_24_ and EV@Y_72_ promoted collagen synthesis in Hs27 fibroblast cells. Hs27 cells were grown until they reached 90% confluency and then switched to serum-free media containing retinol, EV@Y_24_, or EV@Y_72_. (A) After 24 h, the content of procollagen type 1 was confirmed in cultured medium using an enzyme-linked immunosorbent assay (ELISA) kit. (B) The expression of COL1A1, COL3A1, elastin, MMP1, and TIMP1 was evaluated by RT-PCR. (C) The expression of COL1A1, COL3A1, and MMP1 protein was evaluated by western blotting. Data are presented as mean ± SD (*n* = 3). *P < 0.05, **P < 0.01, and ***P < 0.001.

The potential use of EVs in wound treatment is gaining significant attention. Numerous studies have reported that EVs derived from various cells promote wound healing, reduce scar formation, and offer substantial advantages over traditional treatment methods [3]. EVs can accelerate wound healing by affecting the proliferation and migration of endothelial, epithelial, and dermal fibroblasts and increasing the production of collagen I and III in the early stages of wound healing [30]. Type I, II, and III collagen constitute 80% to 90% of the total collagen in the human body [31]. In the skin, type I collagen accounts for 80%, while type III collagen makes up 15% [32]. In this study, we evaluated the effect of EVs produced under different culture conditions on collagen synthesis. Hs27 fibroblasts were treated with EV@Y_24_ and EV@Y_72_ to evaluate EV-related functions in collagen synthesis using enzyme-linked immunosorbent assay (ELISA), RT-PCR, and western blotting. Figure 6A shows ELISA results analyzing collagen I secretion into the medium, confirming that yeast-derived EVs promoted the synthesis of collagen I and collagen III. The optimal efficiency was observed at a concentration of 10^8^ particles/ml, surpassing the effectiveness of 20 μM retinol, used as a positive control. At this concentration, EV@Y_24_ and EV@Y_72_ increased collagen synthesis by 38% and 24%, respectively, with EV@Y_24_ being more effective. Figure 6B and C, which show mRNA and protein analysis via RT-PCR and western blotting, reflected the same trend as the ELISA results. Overall, yeast-derived EVs promoted the synthesis of collagen I, collagen III, and MMP1. All EV samples showed no effect on Hs27 cell proliferation and exhibited no toxicity (Fig. S4).

## Discussion

In this study, we characterized EVs derived from *S. cerevisiae* cultured for 24 h (EV@Y_24_) and 72 h (EV@Y_72_), analyzing their properties, protein content, and potential effects on human cells. Our findings reveal that the culture period significantly influences the characteristics and composition of EVs. EV@Y_72_ showed a higher particle count but a smaller average particle size compared to EV@Y_24_. The prolonged culture period likely leads to increased EV secretion and particle degradation, resulting in smaller vesicles. These results are consistent with previous research showing that EVs can be degraded by degradative enzymes such as protease if the fermentation process or culture period is excessively long [33]. Additionally, when comparing the particle characteristics of EVs extracted from Y_24_ and Y_72_ using isolation buffer over the same period, the average particle size of both samples was similar at 160 nm. This finding supports the hypothesis that EVs may degrade and become smaller with prolonged culture periods.

As introduced, EVs are categorized into subgroups, such as exosomes, microvesicles, and apoptotic bodies, based on their origin [1,34]. In this study, we were the first to propose Pep12, a t-SNARE protein localized to late endosomes, as a specific marker for endosome-derived EVs. In Fig. 1G, EV@Y_24_ was enriched with the late-endosome protein Pep12, while EV@Y_72_ contained more proteins from various organelles, such as VMA2 and GAPDH. GAPDH, generally considered a housekeeping gene, functions in glycolysis and is found in the periplasm (anchored between the cell wall and PM) [35]. Previous studies have shown that GAPDH is tightly attached to the cell surface in *S. cerevisiae* cultured for more than 48 h and is located in outer membrane vesicles [36,37]. Increased GAPDH secretion may result from the depletion of GAPDH substrates in cells subjected to prolonged starvation [38]. Additionally, Vid (vacuole import and degradation) vesicles, which can be secreted into extracellular regions during glucose starvation, contain VMA2 [38,39]. These vesicles attach to endosomes and subsequently fuse with vacuoles, where they are degraded [40]. Similar in size and density to exosomes, Vid vesicles play a critical role in cellular processes under nutrient-deprived conditions. Therefore, the results in Fig. 1G indicate that EV@Y_72_ contains many outer membrane vesicles and includes proteins derived from various organelles such as the vacuole and Vid vesicles. Consistent with this, proteome analysis confirmed that EV@Y_72_ includes a diverse range of proteins from multiple cellular organelles. These findings suggest that EV@Y_24_ is more representative of endosome-derived exosomes, whereas EV@Y_72_ includes a broader range of EV subpopulations.

We subsequently analyzed the cellular uptake of EVs to identify functional differences between the 2 EV groups. Functional assays demonstrated that EV@Y_24_ is internalized by cells more efficiently than EV@Y_72_, potentially due to the presence of Pep12. There is a strong hypothesis that SNARE proteins mediate the fusion of MVBs with the PM in the final step of exosome secretion [24,41]. Similarly, SNARE proteins present in exosomes are likely to mediate the endocytosis of EVs [24]. From this perspective, the Pep12 SNARE protein in endosome-based vesicles, which is abundantly present in EV@Y_24_, may have a significant impact on cellular internalization. Additionally, in proteome analysis results, EV@Y_24_ contains numerous GPI-anchored proteins such as 1,3-beta-glucanosyltransferases and ZPS1 (accession: A0A6C1E6A3; Fig. 4), which may facilitate cell internalization. As GPI-anchored proteins, glucanosyltransferases have been widely studied for their roles in ER- to Golgi vesicle-mediated transport and lipid raft-mediated endocytosis [25]. It may be one of the factors influencing the high cellular uptake of EV@Y_24_. Glucanosyltransferases may reside in the PM, but their complete inclusion as a component of EVs needs to be approached cautiously, as they have recently been reported to be covalently attached to cell wall glucans [26]. However, the endocytosis mechanism of EVs has not been clearly elucidated and further studies are required.

We also observed that yeast-derived EVs enhanced the expression of type I collagen and MMP1 in fibroblasts. The dermal extracellular matrix (ECM) is mainly composed of type I collagen fibrils. These fibrils play essential roles in maintaining skin integrity and function. Fragmentation and disassembly of collagen fibrils are characteristic indicators of skin aging, leading to a decline in skin elasticity and resilience [42]. Previous studies have reported a significant increase in MMP1 in aged human skin dermal fibroblasts [42]. In contrast, recent studies have demonstrated a beneficial role for MMP1 in wound healing. The functions of MMPs are diverse and not solely involved in ECM turnover [43]. Indeed, many MMPs have been demonstrated to control the processes of cell migration and angiogenesis, and in various cell cultures, keratinocyte migration on type I collagen is dependent on MMP1 [44]. Additionally, the ability of certain MMPs to degrade the ECM and remove adhesion molecules such as cadherins and integrins makes them well suited to promote cell migration and inhibit scar formation during the healing process [45]. Therefore, *S. cerevisiae*-derived EVs, which can up-regulate collagen types I and III as well as MMP1, have significant potential to be developed as promising materials in regenerative medicine.

We aimed to identify miRNAs within EVs using transcriptome analysis and explore their potential roles in wound healing and cell proliferation. According to several studies, specific proteins or miRNAs present in EVs can induce wound healing or cell proliferation by regulating AKT and ERK signaling pathways [46,47]. In this study, several miRNAs identified were found to target mRNAs involved in the AKT and ERK pathways such as DUSP, PTPN, and SPRED1 (Table 2) [48–50]. These miRNAs may positively regulate AKT and ERK signaling, leading to increased collagen synthesis. Additionally, the transcriptome analysis results suggest that yeast-derived EVs may positively regulate the Wnt signaling pathway (Fig. 5D and Table 2). The Wnt/β-catenin pathway plays a crucial role in regulating cell development, maintenance, wound healing, and collagen synthesis [3]. Although many of these miRNAs are present in EV@Y_72_, EV@Y_24_ exhibited a superior collagen production effect. These findings suggest that endocytosis plays a significant role in determining the efficacy of EVs. The exact role of each component within an EV remains unknown. To elucidate the precise functional mechanisms, further research is needed to determine how yeast proteins affect human cells and to understand the effects of individual miRNAs.

**Table 2.** miRNAs in yeast-derived EVs target mRNAs related to the inhibition of AKT/ERK signaling pathways and the Wnt signaling pathway

Target mRNA description	Target mRNA abbreviation	miRNA sequence	Log_2_FC (EV@Y_24_ vs. EV@Y_72_)
Dephosphorylated by dual-specific phosphatases (DUSP) [48,50]	DUSP19	UUUCUUACGAAACCGGGAC	4.847
	DUSP6	UUUUUUUUUUUGUUUUGGC UUUUUUUUUUGUUUUGGC	0.557
Tyrosine-protein phosphatase nonreceptor type (PTPN) [48,49]	PTPN11	AUGUAGUCAACCAUAAUGAGGGU AUUUGCACUGGUUUUGUGCUG	−0.079
	PTPN14	UUUUUUUUUUGUUUUGGC UUUUUUUUUUUGUUUUGGC	0.557
Sprouty-related, EVH1 domain-containing protein (SPRED1) [48]	SPRED1	UAGUAAAAACAAUACUUA AAGAAACCAUUAGAUCAAUAAGC	2.980
Negative regulation of canonical Wnt signaling pathway [3] (Fig. 5D)	TLE4, AMER1, WWTR1, TCF7L2, NHERF1, HDAC1, LRP4, TMEM64, LIMD1, MCC, FOXO3, TMEM170B, SHISA6, LATS1, SFRP1, ZNRF3, SOX17, CDH2, ANKRD6, RBMS3, DACT1	24 miRNAs (Table S4)	0.481

ERK, extracellular signal-regulated kinase

Our study underscores the importance of culture duration in determining the properties and functional outcomes of yeast-derived EVs. In conclusion, EV@Y_24_ contained a higher proportion of endosome-derived vesicles compared to EV@Y_72_, leading to greater efficiency in cellular internalization and promotion of collagen synthesis. Therefore, EVs isolated from 24-h yeast cultures, which have not experienced energy depletion, may be especially effective for applications like drug delivery and wound healing. Additionally, proteome and transcriptome analyses identified distinct protein and miRNA profiles between EV@Y_24_ and EV@Y_72_. Notably, several miRNAs in both EV types target mRNAs involved in the AKT, ERK, and Wnt signaling pathways, which are known to regulate collagen synthesis. The differential effects on collagen production may be attributed to the efficiency of endocytosis and the specific protein and miRNA cargo of the EVs. This study has limitations, as it focused solely on controlling culture duration to examine the characteristics of EVs. To better understand how various factors in the culture conditions influence EV secretion, future research should include in-depth studies that manipulate the composition of the culture medium and explore a broader range of culture durations. Additionally, the study lacks mechanistic insights and functional analyses of individual EV components. Therefore, further research is needed to elucidate the precise mechanisms by which yeast proteins and miRNAs within EVs affect human cells, which will enhance our understanding of EV-mediated intercellular communication and therapeutic potential.

## Data Availability

The data that support the findings of this study are available from the corresponding authors upon reasonable request.
