# Supplementary material for: Impact of Culture Duration on the Properties and Functionality of Yeast-Derived Extracellular Vesicles
Source: Biomater Res. 2025 May 6;29:0201. doi: 10.34133/bmr.0201 (PMC12053258; doi:10.34133/bmr.0201)
Supplement: Supplementary 1 — Figs. S1 to S4 Tables S1 to S4 [file bmr.0201.f1.zip › Jeon_Suppl Tables_Biomaterials Research_re.docx]

**Table S1. Identified proteins in EV@Y24 and EV@Y72**

| **Proteins in EV@Y24** | | | | | | | | | |
| --- | --- | --- | --- | --- | --- | --- | --- | --- | --- |
| **Entry** | **Accession** | **Description** | **score** | **avgMass** | **Matched Products** | **matched Peptides** | **digestPeps** | **seq Cover(%)** | **Auto Curate** |
| N1P2H7_YEASC | N1P2H7 | Glyceraldehyde-3-phosphate dehydrogenase OS=Saccharomyces cerevisiae (strain CEN.PK113-7D) OX=889517 GN=CENPK1137D_3157 PE=3 SV=1 | 1278.6 | 35860.8 | 82 | 7 | 31 | 42.47 | Green |
| G2WES0_YEASK | G2WES0 | Glyceraldehyde-3-phosphate dehydrogenase OS=Saccharomyces cerevisiae (strain Kyokai no. 7 / NBRC 101557) OX=721032 GN=K7_TDH3 PE=3 SV=1 | 1278.6 | 35860.8 | 82 | 7 | 31 | 42.47 | Green |
| A0A6A5PYU8_YEASX | A0A6A5PYU8 | Glyceraldehyde-3-phosphate dehydrogenase OS=Saccharomyces cerevisiae OX=4932 GN=TDH3 PE=3 SV=1 | 1278.6 | 35860.8 | 82 | 7 | 31 | 42.47 | Green |
| G3P3_YEAST | P00359 | Glyceraldehyde-3-phosphate dehydrogenase 3 OS=Saccharomyces cerevisiae (strain ATCC 204508 / S288c) OX=559292 GN=TDH3 PE=1 SV=3 | 1278.6 | 35860.8 | 82 | 7 | 31 | 42.47 | Green |
| G2WFP7_YEASK | G2WFP7 | phosphopyruvate hydratase OS=Saccharomyces cerevisiae (strain Kyokai no. 7 / NBRC 101557) OX=721032 GN=K7_ENO2 PE=3 SV=1 | 1916.3 | 46971.3 | 108 | 11 | 34 | 41.88 | Green |
| B3LST6_YEAS1 | B3LST6 | phosphopyruvate hydratase OS=Saccharomyces cerevisiae (strain RM11-1a) OX=285006 GN=SCRG_04885 PE=3 SV=1 | 1916.3 | 46971.3 | 108 | 11 | 34 | 41.88 | Green |
| A6ZT81_YEAS7 | A6ZT81 | phosphopyruvate hydratase OS=Saccharomyces cerevisiae (strain YJM789) OX=307796 GN=ENO2 PE=3 SV=1 | 1916.3 | 46971.3 | 108 | 11 | 34 | 41.88 | Green |
| A0A6C1DSX5_SACPS | A0A6C1DSX5 | phosphopyruvate hydratase OS=Saccharomyces pastorianus OX=27292 GN=ENO2 PE=3 SV=1 | 1916.3 | 46971.3 | 108 | 11 | 34 | 41.88 | Green |
| A0A6A5PY35_YEASX | A0A6A5PY35 | phosphopyruvate hydratase OS=Saccharomyces cerevisiae OX=4932 GN=ENO2 PE=3 SV=1 | 1916.3 | 46971.3 | 108 | 11 | 34 | 41.88 | Green |
| ENO2_YEAST | P00925 | Enolase 2 OS=Saccharomyces cerevisiae (strain ATCC 204508 / S288c) OX=559292 GN=ENO2 PE=1 SV=2 | 1916.3 | 46971.3 | 108 | 11 | 34 | 41.88 | Green |
| H0GHK6_SACCK | H0GHK6 | phosphopyruvate hydratase OS=Saccharomyces cerevisiae x Saccharomyces kudriavzevii (strain VIN7) OX=1095631 GN=VIN7_2265 PE=3 SV=1 | 1723.6 | 46971.3 | 98 | 10 | 35 | 37.76 | Green |
| N1P5V1_YEASC | N1P5V1 | Phosphatidylglycerol/phosphatidylinositol transfer protein OS=Saccharomyces cerevisiae (strain CEN.PK113-7D) OX=889517 GN=CENPK1137D_3781 PE=3 SV=1 | 278.8 | 19291.0 | 16 | 4 | 12 | 36.99 | Green |
| G2WCB4_YEASK | G2WCB4 | Phosphatidylglycerol/phosphatidylinositol transfer protein OS=Saccharomyces cerevisiae (strain Kyokai no. 7 / NBRC 101557) OX=721032 GN=K7_NPC2 PE=3 SV=1 | 278.8 | 19261.0 | 16 | 4 | 12 | 36.99 | Green |
| A6ZXS8_YEAS7 | A6ZXS8 | Phosphatidylglycerol/phosphatidylinositol transfer protein OS=Saccharomyces cerevisiae (strain YJM789) OX=307796 GN=NPC2 PE=3 SV=1 | 278.8 | 19261.0 | 16 | 4 | 12 | 36.99 | Green |
| A0A8H8UMN4_YEASX | A0A8H8UMN4 | Phosphatidylglycerol/phosphatidylinositol transfer protein OS=Saccharomyces cerevisiae OX=4932 GN=NPC2 PE=3 SV=1 | 278.8 | 19291.0 | 16 | 4 | 12 | 36.99 | Green |
| NPC2_YEAST | Q12408 | Phosphatidylglycerol/phosphatidylinositol transfer protein OS=Saccharomyces cerevisiae (strain ATCC 204508 / S288c) OX=559292 GN=NPC2 PE=1 SV=1 | 278.8 | 19291.0 | 16 | 4 | 12 | 36.99 | Green |
| H0GW01_SACCK | H0GW01 | phosphopyruvate hydratase OS=Saccharomyces cerevisiae x Saccharomyces kudriavzevii (strain VIN7) OX=1095631 GN=VIN7_7633 PE=3 SV=1 | 1930.8 | 46811.0 | 113 | 10 | 32 | 36.84 | Green |
| H0GGT7_SACCK | H0GGT7 | Glyceraldehyde-3-phosphate dehydrogenase OS=Saccharomyces cerevisiae x Saccharomyces kudriavzevii (strain VIN7) OX=1095631 GN=VIN7_1968 PE=3 SV=1 | 1166.8 | 35847.7 | 70 | 6 | 31 | 36.14 | Green |
| C8Z985_YEAS8 | C8Z985 | Glyceraldehyde-3-phosphate dehydrogenase OS=Saccharomyces cerevisiae (strain Lalvin EC1118 / Prise de mousse) OX=643680 GN=EC1118_1G1_5204g PE=3 SV=1 | 1166.8 | 35847.7 | 70 | 6 | 31 | 36.14 | Green |
| B5VJD4_YEAS6 | B5VJD4 | Glyceraldehyde-3-phosphate dehydrogenase OS=Saccharomyces cerevisiae (strain AWRI1631) OX=545124 GN=AWRI1631_74130 PE=3 SV=1 | 1166.8 | 35847.7 | 70 | 6 | 31 | 36.14 | Green |
| B3LI45_YEAS1 | B3LI45 | Glyceraldehyde-3-phosphate dehydrogenase OS=Saccharomyces cerevisiae (strain RM11-1a) OX=285006 GN=SCRG_00830 PE=3 SV=1 | 1166.8 | 35847.7 | 70 | 6 | 31 | 36.14 | Green |
| A6ZUK2_YEAS7 | A6ZUK2 | Glyceraldehyde-3-phosphate dehydrogenase OS=Saccharomyces cerevisiae (strain YJM789) OX=307796 GN=TDH3 PE=3 SV=1 | 1166.8 | 35847.7 | 70 | 6 | 31 | 36.14 | Green |
| A0A6C1DS61_SACPS | A0A6C1DS61 | Glyceraldehyde-3-phosphate dehydrogenase OS=Saccharomyces pastorianus OX=27292 GN=TDH3 PE=3 SV=1 | 1166.8 | 35861.8 | 70 | 6 | 31 | 36.14 | Green |
| N1P439_YEASC | N1P439 | phosphopyruvate hydratase OS=Saccharomyces cerevisiae (strain CEN.PK113-7D) OX=889517 GN=CENPK1137D_3221 PE=3 SV=1 | 1654.8 | 46873.2 | 77 | 8 | 36 | 34.78 | Green |
| H0GGY4_SACCK | H0GGY4 | phosphopyruvate hydratase OS=Saccharomyces cerevisiae x Saccharomyces kudriavzevii (strain VIN7) OX=1095631 GN=VIN7_2021 PE=3 SV=1 | 1654.8 | 46859.2 | 77 | 8 | 36 | 34.78 | Green |
| G2WEY0_YEASK | G2WEY0 | phosphopyruvate hydratase OS=Saccharomyces cerevisiae (strain Kyokai no. 7 / NBRC 101557) OX=721032 GN=K7_ENO1 PE=3 SV=1 | 1654.8 | 46859.2 | 77 | 8 | 36 | 34.78 | Green |
| C8Z9E6_YEAS8 | C8Z9E6 | phosphopyruvate hydratase OS=Saccharomyces cerevisiae (strain Lalvin EC1118 / Prise de mousse) OX=643680 GN=EC1118_1G1_5908g PE=3 SV=1 | 1654.8 | 46859.2 | 77 | 8 | 36 | 34.78 | Green |
| B3LHY6_YEAS1 | B3LHY6 | phosphopyruvate hydratase OS=Saccharomyces cerevisiae (strain RM11-1a) OX=285006 GN=SCRG_00770 PE=3 SV=1 | 1654.8 | 46873.2 | 77 | 8 | 36 | 34.78 | Green |
| A6ZUR2_YEAS7 | A6ZUR2 | phosphopyruvate hydratase OS=Saccharomyces cerevisiae (strain YJM789) OX=307796 GN=ENO1 PE=3 SV=1 | 1654.8 | 46859.2 | 77 | 8 | 36 | 34.78 | Green |
| A0A8H8UKY7_YEASX | A0A8H8UKY7 | phosphopyruvate hydratase OS=Saccharomyces cerevisiae OX=4932 GN=ENO1 PE=3 SV=1 | 1654.8 | 46873.2 | 77 | 8 | 36 | 34.78 | Green |
| A0A6C1DT43_SACPS | A0A6C1DT43 | phosphopyruvate hydratase OS=Saccharomyces pastorianus OX=27292 GN=ENO1_1 PE=3 SV=1 | 1654.8 | 46859.2 | 77 | 8 | 36 | 34.78 | Green |
| ENO1_YEAST | P00924 | Enolase 1 OS=Saccharomyces cerevisiae (strain ATCC 204508 / S288c) OX=559292 GN=ENO1 PE=1 SV=3 | 1654.8 | 46873.2 | 77 | 8 | 36 | 34.78 | Green |
| S5S176_YEASX | S5S176 | Alcohol dehydrogenase OS=Saccharomyces cerevisiae OX=4932 GN=ADH1 PE=3 SV=1 | 429.9 | 37307.4 | 73 | 8 | 26 | 34.77 | Green |
| S5RZC2_YEASX | S5RZC2 | Alcohol dehydrogenase OS=Saccharomyces cerevisiae OX=4932 GN=ADH1 PE=1 SV=1 | 429.9 | 37295.4 | 73 | 8 | 26 | 34.77 | Green |
| S5RK20_YEASX | S5RK20 | Alcohol dehydrogenase OS=Saccharomyces cerevisiae OX=4932 GN=ADH1 PE=3 SV=1 | 429.9 | 37293.4 | 73 | 8 | 26 | 34.77 | Green |
| S5RCH3_YEASX | S5RCH3 | Alcohol dehydrogenase OS=Saccharomyces cerevisiae OX=4932 GN=ADH1 PE=3 SV=1 | 429.9 | 37294.4 | 73 | 8 | 26 | 34.77 | Green |
| M9VEX7_YEAS8 | M9VEX7 | Phenylacetaldehyde dehydrogenase OS=Saccharomyces cerevisiae (strain Lalvin EC1118 / Prise de mousse) OX=643680 PE=3 SV=1 | 429.9 | 37306.5 | 73 | 8 | 26 | 34.77 | Green |
| H0GN42_SACCK | H0GN42 | Adh1p OS=Saccharomyces cerevisiae x Saccharomyces kudriavzevii (strain VIN7) OX=1095631 GN=VIN7_4366 PE=3 SV=1 | 429.9 | 37208.3 | 73 | 8 | 25 | 34.77 | Green |
| G2WMF0_YEASK | G2WMF0 | K7_Adh1p OS=Saccharomyces cerevisiae (strain Kyokai no. 7 / NBRC 101557) OX=721032 GN=K7_ADH1 PE=3 SV=1 | 429.9 | 37295.4 | 73 | 8 | 26 | 34.77 | Green |
| C8ZHN0_YEAS8 | C8ZHN0 | Adh1p OS=Saccharomyces cerevisiae (strain Lalvin EC1118 / Prise de mousse) OX=643680 GN=EC1118_1O4_0859g PE=3 SV=1 | 429.9 | 37307.4 | 73 | 8 | 26 | 34.77 | Green |
| B6UQD0_SACPS | B6UQD0 | Alcohol dehydrogenase OS=Saccharomyces pastorianus OX=27292 GN=ADH1 PE=3 SV=1 | 429.9 | 37295.4 | 73 | 8 | 26 | 34.77 | Green |
| B5VRG9_YEAS6 | B5VRG9 | YOL086Cp-like protein OS=Saccharomyces cerevisiae (strain AWRI1631) OX=545124 GN=AWRI1631_150730 PE=3 SV=1 | 429.9 | 37295.4 | 73 | 8 | 26 | 34.77 | Green |
| B3LIX8_YEAS1 | B3LIX8 | Alcohol dehydrogenase OS=Saccharomyces cerevisiae (strain RM11-1a) OX=285006 GN=SCRG_01319 PE=3 SV=1 | 429.9 | 37307.4 | 73 | 8 | 26 | 34.77 | Green |
| A6ZNC5_YEAS7 | A6ZNC5 | Alcohol dehydrogenase OS=Saccharomyces cerevisiae (strain YJM789) OX=307796 GN=ADH1 PE=3 SV=1 | 429.9 | 37295.4 | 73 | 8 | 26 | 34.77 | Green |
| A0A8H4BVB5_YEASX | A0A8H4BVB5 | ADH1 isoform 1 OS=Saccharomyces cerevisiae OX=4932 GN=ADH1 PE=3 SV=1 | 429.9 | 37305.4 | 73 | 8 | 26 | 34.77 | Green |
| ADH1_YEAST | P00330 | Alcohol dehydrogenase 1 OS=Saccharomyces cerevisiae (strain ATCC 204508 / S288c) OX=559292 GN=ADH1 PE=1 SV=5 | 429.9 | 37305.4 | 73 | 8 | 26 | 34.77 | Green |
| N1P465_YEASC | N1P465 | Scw4p OS=Saccharomyces cerevisiae (strain CEN.PK113-7D) OX=889517 GN=CENPK1137D_3246 PE=3 SV=1 | 1990.5 | 40458.2 | 71 | 6 | 21 | 34.72 | Green |
| A0A8H8UL09_YEASX | A0A8H8UL09 | SCW4 isoform 1 OS=Saccharomyces cerevisiae OX=4932 GN=SCW4 PE=3 SV=1 | 1990.5 | 40458.2 | 71 | 6 | 21 | 34.72 | Green |
| SCW4_YEAST | P53334 | Probable family 17 glucosidase SCW4 OS=Saccharomyces cerevisiae (strain ATCC 204508 / S288c) OX=559292 GN=SCW4 PE=1 SV=1 | 1990.5 | 40458.2 | 73 | 6 | 21 | 34.72 | Green |
| B5VJJ6_YEAS6 | B5VJJ6 | phosphopyruvate hydratase OS=Saccharomyces cerevisiae (strain AWRI1631) OX=545124 GN=AWRI1631_74770 PE=3 SV=1 | 1654.8 | 41765.5 | 75 | 7 | 33 | 33.42 | Green |
| H0GK16_SACCK | H0GK16 | branched-chain-2-oxoacid decarboxylase OS=Saccharomyces cerevisiae x Saccharomyces kudriavzevii (strain VIN7) OX=1095631 GN=VIN7_3196 PE=3 SV=1 | 237.6 | 61723.6 | 72 | 11 | 35 | 33.21 | Green |
| B3LT15_YEAS1 | B3LT15 | branched-chain-2-oxoacid decarboxylase OS=Saccharomyces cerevisiae (strain RM11-1a) OX=285006 GN=SCRG_05030 PE=3 SV=1 | 237.6 | 61723.6 | 72 | 11 | 35 | 33.21 | Green |
| G2WIN6_YEASK | G2WIN6 | branched-chain-2-oxoacid decarboxylase OS=Saccharomyces cerevisiae (strain Kyokai no. 7 / NBRC 101557) OX=721032 GN=K7_PDC1 PE=3 SV=1 | 237.6 | 61737.6 | 72 | 11 | 35 | 33.21 | Green |
| A7A0U9_YEAS7 | A7A0U9 | branched-chain-2-oxoacid decarboxylase OS=Saccharomyces cerevisiae (strain YJM789) OX=307796 GN=PDC1 PE=3 SV=1 | 237.6 | 61723.6 | 72 | 11 | 35 | 33.21 | Green |
| A0A7I9FNH9_YEASX | A0A7I9FNH9 | branched-chain-2-oxoacid decarboxylase OS=Saccharomyces cerevisiae OX=4932 GN=PDC1 PE=3 SV=1 | 237.6 | 61723.6 | 72 | 11 | 35 | 33.21 | Green |
| A0A6C1DVV6_SACPS | A0A6C1DVV6 | branched-chain-2-oxoacid decarboxylase OS=Saccharomyces pastorianus OX=27292 GN=PDC1_1 PE=3 SV=1 | 237.6 | 61723.6 | 72 | 11 | 35 | 33.21 | Green |
| PDC1_YEAST | P06169 | Pyruvate decarboxylase isozyme 1 OS=Saccharomyces cerevisiae (strain ATCC 204508 / S288c) OX=559292 GN=PDC1 PE=1 SV=7 | 237.6 | 61723.6 | 72 | 11 | 35 | 33.21 | Green |
| C8ZD16_YEAS8 | C8ZD16 | branched-chain-2-oxoacid decarboxylase OS=Saccharomyces cerevisiae (strain Lalvin EC1118 / Prise de mousse) OX=643680 GN=EC1118_1L10_1178g PE=3 SV=1 | 231.6 | 61757.6 | 68 | 10 | 36 | 31.26 | Green |
| A0A3G3NDH9_YEASX | A0A3G3NDH9 | Alcohol dehydrogenase OS=Saccharomyces cerevisiae OX=4932 GN=ADH1 PE=3 SV=1 | 341.7 | 37278.4 | 63 | 7 | 26 | 31.03 | Green |
| G2WF02_YEASK | G2WF02 | K7_Scw4p OS=Saccharomyces cerevisiae (strain Kyokai no. 7 / NBRC 101557) OX=721032 GN=K7_SCW4 PE=3 SV=1 | 1610.7 | 40459.2 | 49 | 5 | 21 | 29.79 | Green |
| C7GNF6_YEAS2 | C7GNF6 | Scw4p OS=Saccharomyces cerevisiae (strain JAY291) OX=574961 GN=SCW4 PE=3 SV=1 | 1610.7 | 40459.2 | 49 | 5 | 21 | 29.79 | Green |
| A6ZUT5_YEAS7 | A6ZUT5 | Soluble cell wall protein OS=Saccharomyces cerevisiae (strain YJM789) OX=307796 GN=SCW4 PE=3 SV=1 | 1610.7 | 40459.2 | 49 | 5 | 21 | 29.79 | Green |
| N1P0G2_YEASC | N1P0G2 | branched-chain-2-oxoacid decarboxylase OS=Saccharomyces cerevisiae (strain CEN.PK113-7D) OX=889517 GN=CENPK1137D_891 PE=3 SV=1 | 237.6 | 61738.6 | 51 | 9 | 35 | 29.66 | Green |
| N1P004_YEASC | N1P004 | Glyceraldehyde-3-phosphate dehydrogenase OS=Saccharomyces cerevisiae (strain CEN.PK113-7D) OX=889517 GN=CENPK1137D_1304 PE=3 SV=1 | 888.0 | 35961.0 | 44 | 5 | 31 | 28.92 | Green |
| G2WH19_YEASK | G2WH19 | Glyceraldehyde-3-phosphate dehydrogenase OS=Saccharomyces cerevisiae (strain Kyokai no. 7 / NBRC 101557) OX=721032 GN=K7_TDH2 PE=3 SV=1 | 888.0 | 35961.0 | 44 | 5 | 31 | 28.92 | Green |
| C8ZBG4_YEAS8 | C8ZBG4 | Glyceraldehyde-3-phosphate dehydrogenase OS=Saccharomyces cerevisiae (strain Lalvin EC1118 / Prise de mousse) OX=643680 GN=EC1118_1J11_2685g PE=3 SV=1 | 888.0 | 35961.0 | 44 | 5 | 31 | 28.92 | Green |
| B3LQA8_YEAS1 | B3LQA8 | Glyceraldehyde-3-phosphate dehydrogenase OS=Saccharomyces cerevisiae (strain RM11-1a) OX=285006 GN=SCRG_03670 PE=3 SV=1 | 888.0 | 35961.0 | 44 | 5 | 31 | 28.92 | Green |
| A6ZPX6_YEAS7 | A6ZPX6 | Glyceraldehyde-3-phosphate dehydrogenase OS=Saccharomyces cerevisiae (strain YJM789) OX=307796 GN=TDH2 PE=3 SV=1 | 888.0 | 35961.0 | 44 | 5 | 31 | 28.92 | Green |
| A0A6A5PXB1_YEASX | A0A6A5PXB1 | Glyceraldehyde-3-phosphate dehydrogenase OS=Saccharomyces cerevisiae OX=4932 GN=TDH2 PE=3 SV=1 | 888.0 | 35961.0 | 44 | 5 | 31 | 28.92 | Green |
| G3P2_YEAST | P00358 | Glyceraldehyde-3-phosphate dehydrogenase 2 OS=Saccharomyces cerevisiae (strain ATCC 204508 / S288c) OX=559292 GN=TDH2 PE=1 SV=3 | 888.0 | 35961.0 | 44 | 5 | 31 | 28.92 | Green |
| H0GH04_SACCK | H0GH04 | Scw4p OS=Saccharomyces cerevisiae x Saccharomyces kudriavzevii (strain VIN7) OX=1095631 GN=VIN7_2044 PE=3 SV=1 | 1601.4 | 40445.2 | 43 | 4 | 21 | 27.72 | Green |
| C8Z9H2_YEAS8 | C8Z9H2 | Scw4p OS=Saccharomyces cerevisiae (strain Lalvin EC1118 / Prise de mousse) OX=643680 GN=EC1118_1G1_6194g PE=3 SV=1 | 1601.4 | 40445.2 | 43 | 4 | 21 | 27.72 | Green |
| B3LHW6_YEAS1 | B3LHW6 | Soluble cell wall protein OS=Saccharomyces cerevisiae (strain RM11-1a) OX=285006 GN=SCRG_00748 PE=3 SV=1 | 1601.4 | 40445.2 | 43 | 4 | 21 | 27.72 | Green |
| A0A6C1DSE2_SACPS | A0A6C1DSE2 | Glycoside hydrolase_ 17 OS=Saccharomyces pastorianus OX=27292 GN=SCW4_1 PE=3 SV=1 | 1601.4 | 40445.2 | 43 | 4 | 21 | 27.72 | Green |
| S4VPL7_YEASX | S4VPL7 | Superoxide dismutase OS=Saccharomyces cerevisiae OX=4932 GN=sod2 PE=3 SV=1 | 2403.0 | 25831.3 | 71 | 5 | 19 | 27.04 | Green |
| S4VIJ3_YEASX | S4VIJ3 | Superoxide dismutase OS=Saccharomyces cerevisiae var. ellipsoideus OX=1348153 GN=sod2 PE=3 SV=1 | 2403.0 | 25831.3 | 71 | 5 | 19 | 27.04 | Green |
| N1P9K8_YEASC | N1P9K8 | Superoxide dismutase OS=Saccharomyces cerevisiae (strain CEN.PK113-7D) OX=889517 GN=CENPK1137D_5400 PE=3 SV=1 | 2403.0 | 25831.3 | 71 | 5 | 19 | 27.04 | Green |
| H0GH32_SACCK | H0GH32 | Superoxide dismutase OS=Saccharomyces cerevisiae x Saccharomyces kudriavzevii (strain VIN7) OX=1095631 GN=VIN7_2106 PE=3 SV=1 | 2403.0 | 25831.3 | 71 | 5 | 19 | 27.04 | Green |
| G2WF76_YEASK | G2WF76 | Superoxide dismutase OS=Saccharomyces cerevisiae (strain Kyokai no. 7 / NBRC 101557) OX=721032 GN=K7_SOD2 PE=3 SV=1 | 2403.0 | 25831.3 | 71 | 5 | 19 | 27.04 | Green |
| C8ZA75_YEAS8 | C8ZA75 | Superoxide dismutase OS=Saccharomyces cerevisiae (strain Lalvin EC1118 / Prise de mousse) OX=643680 GN=EC1118_1H21_0716g PE=3 SV=1 | 2403.0 | 25831.3 | 71 | 5 | 19 | 27.04 | Green |
| C7GMQ3_YEAS2 | C7GMQ3 | Superoxide dismutase OS=Saccharomyces cerevisiae (strain JAY291) OX=574961 GN=SOD2 PE=3 SV=1 | 2403.0 | 25831.3 | 71 | 5 | 19 | 27.04 | Green |
| B5VJT4_YEAS6 | B5VJT4 | Superoxide dismutase OS=Saccharomyces cerevisiae (strain AWRI1631) OX=545124 GN=AWRI1631_80530 PE=3 SV=1 | 2403.0 | 25831.3 | 71 | 5 | 19 | 27.04 | Green |
| B3LSC9_YEAS1 | B3LSC9 | Superoxide dismutase OS=Saccharomyces cerevisiae (strain RM11-1a) OX=285006 GN=SCRG_04714 PE=3 SV=1 | 2403.0 | 25831.3 | 71 | 5 | 19 | 27.04 | Green |
| A6ZSR2_YEAS7 | A6ZSR2 | Superoxide dismutase OS=Saccharomyces cerevisiae (strain YJM789) OX=307796 GN=SOD2 PE=3 SV=1 | 2403.0 | 25831.3 | 71 | 5 | 19 | 27.04 | Green |
| A0A6C1DS42_SACPS | A0A6C1DS42 | Superoxide dismutase OS=Saccharomyces pastorianus OX=27292 GN=SOD2_1 PE=3 SV=1 | 2403.0 | 25831.3 | 71 | 5 | 19 | 27.04 | Green |
| SODM_YEAST | P00447 | Superoxide dismutase [Mn]_ mitochondrial OS=Saccharomyces cerevisiae (strain ATCC 204508 / S288c) OX=559292 GN=SOD2 PE=1 SV=1 | 2403.0 | 25831.3 | 71 | 5 | 19 | 27.04 | Green |
| H0GQJ2_SACCK | H0GQJ2 | Fructose-bisphosphate aldolase OS=Saccharomyces cerevisiae x Saccharomyces kudriavzevii (strain VIN7) OX=1095631 GN=VIN7_5279 PE=3 SV=1 | 204.3 | 37793.6 | 39 | 6 | 32 | 26.98 | Green |
| N1P7U8_YEASC | N1P7U8 | Fructose-bisphosphate aldolase OS=Saccharomyces cerevisiae (strain CEN.PK113-7D) OX=889517 GN=CENPK1137D_945 PE=3 SV=1 | 204.3 | 39906.0 | 39 | 6 | 33 | 25.63 | Green |
| H0GJ73_SACCK | H0GJ73 | Fructose-bisphosphate aldolase OS=Saccharomyces cerevisiae x Saccharomyces kudriavzevii (strain VIN7) OX=1095631 GN=VIN7_2949 PE=3 SV=1 | 204.3 | 39906.0 | 39 | 6 | 33 | 25.63 | Green |
| G2WHX2_YEASK | G2WHX2 | Fructose-bisphosphate aldolase OS=Saccharomyces cerevisiae (strain Kyokai no. 7 / NBRC 101557) OX=721032 GN=K7_FBA1 PE=3 SV=1 | 204.3 | 39891.9 | 39 | 6 | 33 | 25.63 | Green |
| C8ZCB2_YEAS8 | C8ZCB2 | Fructose-bisphosphate aldolase OS=Saccharomyces cerevisiae (strain Lalvin EC1118 / Prise de mousse) OX=643680 GN=EC1118_1K5_1860g PE=3 SV=1 | 204.3 | 39906.0 | 39 | 6 | 33 | 25.63 | Green |
| B3LR31_YEAS1 | B3LR31 | Fructose-bisphosphate aldolase OS=Saccharomyces cerevisiae (strain RM11-1a) OX=285006 GN=SCRG_03960 PE=3 SV=1 | 204.3 | 39893.0 | 39 | 6 | 33 | 25.63 | Green |
| A6ZZQ6_YEAS7 | A6ZZQ6 | Fructose-bisphosphate aldolase OS=Saccharomyces cerevisiae (strain YJM789) OX=307796 GN=FBA1 PE=3 SV=1 | 204.3 | 39920.0 | 39 | 6 | 33 | 25.63 | Green |
| A0A8H4FAG4_YEASX | A0A8H4FAG4 | Fructose-bisphosphate aldolase OS=Saccharomyces cerevisiae OX=4932 GN=FBA1 PE=3 SV=1 | 204.3 | 39906.0 | 39 | 6 | 33 | 25.63 | Green |
| A0A6C1DUM3_SACPS | A0A6C1DUM3 | Fructose-bisphosphate aldolase OS=Saccharomyces pastorianus OX=27292 GN=FBA1_1 PE=3 SV=1 | 204.3 | 39906.0 | 39 | 6 | 33 | 25.63 | Green |
| ALF_YEAST | P14540 | Fructose-bisphosphate aldolase OS=Saccharomyces cerevisiae (strain ATCC 204508 / S288c) OX=559292 GN=FBA1 PE=1 SV=3 | 204.3 | 39906.0 | 39 | 6 | 33 | 25.63 | Green |
| H0GVE8_SACCK | H0GVE8 | phosphopyruvate hydratase OS=Saccharomyces cerevisiae x Saccharomyces kudriavzevii (strain VIN7) OX=1095631 GN=VIN7_7387 PE=3 SV=1 | 1648.1 | 46886.3 | 73 | 6 | 35 | 24.94 | Green |
| H0GDT7_SACCK | H0GDT7 | Phosphatidylglycerol/phosphatidylinositol transfer protein OS=Saccharomyces cerevisiae x Saccharomyces kudriavzevii (strain VIN7) OX=1095631 GN=VIN7_0703 PE=3 SV=1 | 204.0 | 19246.9 | 9 | 2 | 12 | 24.86 | Green |
| C8Z4M3_YEAS8 | C8Z4M3 | Phosphatidylglycerol/phosphatidylinositol transfer protein OS=Saccharomyces cerevisiae (strain Lalvin EC1118 / Prise de mousse) OX=643680 GN=EC1118_1D0_1849g PE=3 SV=1 | 204.0 | 19246.9 | 9 | 2 | 12 | 24.86 | Green |
| C7GPW6_YEAS2 | C7GPW6 | Phosphatidylglycerol/phosphatidylinositol transfer protein OS=Saccharomyces cerevisiae (strain JAY291) OX=574961 GN=NPC2 PE=3 SV=1 | 204.0 | 19246.9 | 9 | 2 | 12 | 24.86 | Green |
| B5VFM1_YEAS6 | B5VFM1 | Phosphatidylglycerol/phosphatidylinositol transfer protein OS=Saccharomyces cerevisiae (strain AWRI1631) OX=545124 GN=AWRI1631_41920 PE=3 SV=1 | 204.0 | 19246.9 | 9 | 2 | 12 | 24.86 | Green |
| B3LGU5_YEAS1 | B3LGU5 | Phosphatidylglycerol/phosphatidylinositol transfer protein OS=Saccharomyces cerevisiae (strain RM11-1a) OX=285006 GN=SCRG_00546 PE=3 SV=1 | 204.0 | 19246.9 | 9 | 2 | 12 | 24.86 | Green |
| A0A6C1DN60_SACPS | A0A6C1DN60 | Phosphatidylglycerol/phosphatidylinositol transfer protein OS=Saccharomyces pastorianus OX=27292 GN=NPC2_1 PE=3 SV=1 | 204.0 | 19246.9 | 9 | 2 | 12 | 24.86 | Green |
| C7GU46_YEAS2 | C7GU46 | branched-chain-2-oxoacid decarboxylase OS=Saccharomyces cerevisiae (strain JAY291) OX=574961 GN=PDC1 PE=3 SV=1 | 101.2 | 46938.8 | 46 | 8 | 29 | 24.41 | Green |
| A0A6C1E9W5_SACPS | A0A6C1E9W5 | Glyceraldehyde-3-phosphate dehydrogenase OS=Saccharomyces pastorianus OX=27292 GN=TDH1_2 PE=3 SV=1 | 948.7 | 35765.0 | 28 | 4 | 32 | 23.49 | Green |
| A0A6C1EB88_SACPS | A0A6C1EB88 | Glyceraldehyde-3-phosphate dehydrogenase OS=Saccharomyces pastorianus OX=27292 GN=TDH2_3 PE=3 SV=1 | 861.4 | 35993.0 | 39 | 4 | 31 | 22.59 | Green |
| B5VJM2_YEAS6 | B5VJM2 | YGR279Cp-like protein (Fragment) OS=Saccharomyces cerevisiae (strain AWRI1631) OX=545124 GN=AWRI1631_75030 PE=4 SV=1 | 1505.0 | 28319.8 | 36 | 3 | 9 | 21.66 | Green |
| H0GY85_SACCK | H0GY85 | Yps3p OS=Saccharomyces cerevisiae x Saccharomyces kudriavzevii (strain VIN7) OX=1095631 GN=VIN7_8665 PE=3 SV=1 | 141.7 | 52765.1 | 32 | 7 | 26 | 21.62 | Green |
| A0A6C1DVM7_SACPS | A0A6C1DVM7 | Aspartyl protease OS=Saccharomyces pastorianus OX=27292 GN=YPS3_1 PE=3 SV=1 | 222.6 | 53738.2 | 50 | 6 | 35 | 21.04 | Green |
| A0A6C1E8Y3_SACPS | A0A6C1E8Y3 | Superoxide dismutase OS=Saccharomyces pastorianus OX=27292 GN=SOD2_2 PE=3 SV=1 | 1203.2 | 25969.5 | 46 | 3 | 19 | 21.03 | Green |
| H0GVM2_SACCK | H0GVM2 | Superoxide dismutase OS=Saccharomyces cerevisiae x Saccharomyces kudriavzevii (strain VIN7) OX=1095631 GN=VIN7_7477 PE=3 SV=1 | 873.5 | 25894.3 | 45 | 4 | 19 | 21.03 | Green |
| A0A6A5PSL3_YEASX | A0A6A5PSL3 | YPS3 isoform 1 OS=Saccharomyces cerevisiae OX=4932 GN=YPS3 PE=3 SV=1 | 222.6 | 54798.6 | 50 | 6 | 33 | 20.67 | Green |
| N1NY91_YEASC | N1NY91 | Yps3p OS=Saccharomyces cerevisiae (strain CEN.PK113-7D) OX=889517 GN=CENPK1137D_509 PE=3 SV=1 | 222.6 | 54798.6 | 50 | 6 | 33 | 20.67 | Green |
| G2WIW3_YEASK | G2WIW3 | K7_Yps3p OS=Saccharomyces cerevisiae (strain Kyokai no. 7 / NBRC 101557) OX=721032 GN=K7_YPS3 PE=3 SV=1 | 222.6 | 54798.6 | 50 | 6 | 33 | 20.67 | Green |
| C7GQD1_YEAS2 | C7GQD1 | Yps3p OS=Saccharomyces cerevisiae (strain JAY291) OX=574961 GN=YPS3 PE=3 SV=1 | 222.6 | 54757.5 | 50 | 6 | 33 | 20.67 | Green |
| A7A123_YEAS7 | A7A123 | Aspartic protease OS=Saccharomyces cerevisiae (strain YJM789) OX=307796 GN=YPS3 PE=3 SV=1 | 222.6 | 54798.6 | 50 | 6 | 33 | 20.67 | Green |
| YPS3_YEAST | Q12303 | Aspartic proteinase yapsin-3 OS=Saccharomyces cerevisiae (strain ATCC 204508 / S288c) OX=559292 GN=YPS3 PE=1 SV=1 | 222.6 | 54798.6 | 50 | 6 | 33 | 20.67 | Green |
| A0A6C1E8Q2_SACPS | A0A6C1E8Q2 | phosphopyruvate hydratase OS=Saccharomyces pastorianus OX=27292 GN=ENO1_2 PE=3 SV=1 | 404.3 | 46784.1 | 39 | 5 | 36 | 20.37 | Green |
| A0A6C1EGF6_SACPS | A0A6C1EGF6 | phosphopyruvate hydratase OS=Saccharomyces pastorianus OX=27292 GN=ENO1_3 PE=3 SV=1 | 402.3 | 46688.9 | 38 | 4 | 34 | 20.37 | Green |
| H0GXZ9_SACCK | H0GXZ9 | branched-chain-2-oxoacid decarboxylase OS=Saccharomyces cerevisiae x Saccharomyces kudriavzevii (strain VIN7) OX=1095631 GN=VIN7_8597 PE=3 SV=1 | 174.7 | 61612.4 | 55 | 7 | 34 | 19.72 | Green |
| A0A6C1EBJ7_SACPS | A0A6C1EBJ7 | branched-chain-2-oxoacid decarboxylase OS=Saccharomyces pastorianus OX=27292 GN=PDC1_2 PE=3 SV=1 | 118.0 | 61720.6 | 55 | 8 | 34 | 19.54 | Green |
| N1P8B2_YEASC | N1P8B2 | Phosphoglycerate mutase OS=Saccharomyces cerevisiae (strain CEN.PK113-7D) OX=889517 GN=CENPK1137D_1155 PE=3 SV=1 | 157.0 | 27608.6 | 31 | 5 | 21 | 19.43 | Green |
| H0GJ01_SACCK | H0GJ01 | Phosphoglycerate mutase OS=Saccharomyces cerevisiae x Saccharomyces kudriavzevii (strain VIN7) OX=1095631 GN=VIN7_2869 PE=3 SV=1 | 157.0 | 27608.6 | 31 | 5 | 21 | 19.43 | Green |
| G2WHN7_YEASK | G2WHN7 | Phosphoglycerate mutase OS=Saccharomyces cerevisiae (strain Kyokai no. 7 / NBRC 101557) OX=721032 GN=K7_GPM1 PE=3 SV=1 | 157.0 | 27608.6 | 31 | 5 | 21 | 19.43 | Green |
| C8ZC23_YEAS8 | C8ZC23 | Phosphoglycerate mutase OS=Saccharomyces cerevisiae (strain Lalvin EC1118 / Prise de mousse) OX=643680 GN=EC1118_1K5_0760g PE=3 SV=1 | 157.0 | 27608.6 | 31 | 5 | 21 | 19.43 | Green |
| C7GU53_YEAS2 | C7GU53 | Phosphoglycerate mutase OS=Saccharomyces cerevisiae (strain JAY291) OX=574961 GN=GPM1 PE=3 SV=1 | 157.0 | 27608.6 | 31 | 5 | 21 | 19.43 | Green |
| B3LQU9_YEAS1 | B3LQU9 | Phosphoglycerate mutase OS=Saccharomyces cerevisiae (strain RM11-1a) OX=285006 GN=SCRG_03874 PE=3 SV=1 | 157.0 | 27608.6 | 31 | 5 | 21 | 19.43 | Green |
| A6ZZH0_YEAS7 | A6ZZH0 | Phosphoglycerate mutase OS=Saccharomyces cerevisiae (strain YJM789) OX=307796 GN=GPM1 PE=3 SV=1 | 157.0 | 27608.6 | 31 | 5 | 21 | 19.43 | Green |
| A0A6C1DUX7_SACPS | A0A6C1DUX7 | Phosphoglycerate mutase OS=Saccharomyces pastorianus OX=27292 GN=GPM1_1 PE=3 SV=1 | 157.0 | 27608.6 | 31 | 5 | 21 | 19.43 | Green |
| A0A6A5PSG4_YEASX | A0A6A5PSG4 | Phosphoglycerate mutase OS=Saccharomyces cerevisiae OX=4932 GN=GPM1 PE=3 SV=1 | 157.0 | 27608.6 | 31 | 5 | 21 | 19.43 | Green |
| PMG1_YEAST | P00950 | Phosphoglycerate mutase 1 OS=Saccharomyces cerevisiae (strain ATCC 204508 / S288c) OX=559292 GN=GPM1 PE=1 SV=3 | 157.0 | 27608.6 | 31 | 5 | 21 | 19.43 | Green |
| G3P_EREGS | Q757I2 | Glyceraldehyde-3-phosphate dehydrogenase OS=Eremothecium gossypii (strain ATCC 10895 / CBS 109.51 / FGSC 9923 / NRRL Y-1056) OX=284811 GN=GPD PE=3 SV=2 | 1490.3 | 35633.6 | 41 | 4 | 27 | 19.34 | Green |
| H0GXI1_SACCK | H0GXI1 | Fructose-bisphosphate aldolase OS=Saccharomyces cerevisiae x Saccharomyces kudriavzevii (strain VIN7) OX=1095631 GN=VIN7_8353 PE=3 SV=1 | 155.4 | 39712.7 | 27 | 4 | 32 | 18.94 | Green |
| B5VM12_YEAS6 | B5VM12 | Phosphoglycerate mutase (Fragment) OS=Saccharomyces cerevisiae (strain AWRI1631) OX=545124 GN=AWRI1631_110760 PE=3 SV=1 | 157.0 | 28743.9 | 31 | 5 | 22 | 18.68 | Green |
| B7X717_SACPS | B7X717 | Glyceraldehyde-3-phosphate dehydrogenase OS=Saccharomyces pastorianus OX=27292 GN=YJR009C PE=3 SV=1 | 861.4 | 31134.3 | 32 | 3 | 28 | 17.99 | Green |
| B7X714_SACPS | B7X714 | Glyceraldehyde-3-phosphate dehydrogenase OS=Saccharomyces pastorianus OX=27292 GN=YJR009C PE=3 SV=1 | 861.4 | 31143.3 | 32 | 3 | 28 | 17.99 | Green |
| B7X711_SACPS | B7X711 | Glyceraldehyde-3-phosphate dehydrogenase OS=Saccharomyces pastorianus OX=27292 GN=YJR009C PE=3 SV=1 | 861.4 | 31088.3 | 32 | 3 | 28 | 17.99 | Green |
| C7GP14_YEAS2 | C7GP14 | Fructose-bisphosphate aldolase OS=Saccharomyces cerevisiae (strain JAY291) OX=574961 GN=FBA1 PE=3 SV=1 | 186.6 | 39905.0 | 37 | 5 | 33 | 16.99 | Green |
| H0GK82_SACCK | H0GK82 | Yps3p OS=Saccharomyces cerevisiae x Saccharomyces kudriavzevii (strain VIN7) OX=1095631 GN=VIN7_3267 PE=3 SV=1 | 215.0 | 54779.6 | 45 | 5 | 33 | 16.93 | Green |
| C8ZD91_YEAS8 | C8ZD91 | Yps3p OS=Saccharomyces cerevisiae (strain Lalvin EC1118 / Prise de mousse) OX=643680 GN=EC1118_1L10_2047g PE=3 SV=1 | 215.0 | 54793.6 | 45 | 5 | 33 | 16.93 | Green |
| B5VN56_YEAS6 | B5VN56 | YLR121Cp-like protein OS=Saccharomyces cerevisiae (strain AWRI1631) OX=545124 GN=AWRI1631_121690 PE=3 SV=1 | 215.0 | 54779.6 | 45 | 5 | 33 | 16.93 | Green |
| B3LT85_YEAS1 | B3LT85 | Aspartic proteinase yapsin-3 OS=Saccharomyces cerevisiae (strain RM11-1a) OX=285006 GN=SCRG_05103 PE=3 SV=1 | 215.0 | 54779.6 | 45 | 5 | 33 | 16.93 | Green |
| A0A5Q2WV56_YEASX | A0A5Q2WV56 | branched-chain-2-oxoacid decarboxylase OS=Saccharomyces cerevisiae OX=4932 GN=pdc PE=3 SV=1 | 123.9 | 61722.5 | 46 | 7 | 36 | 16.34 | Green |
| H0GWY1_SACCK | H0GWY1 | Glyceraldehyde-3-phosphate dehydrogenase OS=Saccharomyces cerevisiae x Saccharomyces kudriavzevii (strain VIN7) OX=1095631 GN=VIN7_8041 PE=3 SV=1 | 861.4 | 35898.0 | 32 | 3 | 30 | 15.66 | Green |
| H0GVA3_SACCK | H0GVA3 | Glyceraldehyde-3-phosphate dehydrogenase OS=Saccharomyces cerevisiae x Saccharomyces kudriavzevii (strain VIN7) OX=1095631 GN=VIN7_7332 PE=3 SV=1 | 861.4 | 35907.9 | 32 | 3 | 31 | 15.66 | Green |
| B7X708_SACPS | B7X708 | Glyceraldehyde-3-phosphate dehydrogenase OS=Saccharomyces pastorianus OX=27292 GN=YJR009C PE=3 SV=1 | 861.4 | 35936.0 | 32 | 3 | 31 | 15.66 | Green |
| A0A6C1E7B5_SACPS | A0A6C1E7B5 | Glyceraldehyde-3-phosphate dehydrogenase OS=Saccharomyces pastorianus OX=27292 GN=TDH2_2 PE=3 SV=1 | 861.4 | 35982.1 | 32 | 3 | 31 | 15.66 | Green |
| H0GXC9_SACCK | H0GXC9 | Phosphoglycerate mutase OS=Saccharomyces cerevisiae x Saccharomyces kudriavzevii (strain VIN7) OX=1095631 GN=VIN7_8276 PE=3 SV=1 | 136.1 | 27638.6 | 20 | 2 | 21 | 14.98 | Green |
| N1P635_YEASC | N1P635 | Scw10p OS=Saccharomyces cerevisiae (strain CEN.PK113-7D) OX=889517 GN=CENPK1137D_350 PE=3 SV=1 | 818.4 | 40754.3 | 40 | 4 | 17 | 14.65 | Green |
| H0GLJ6_SACCK | H0GLJ6 | Scw10p OS=Saccharomyces cerevisiae x Saccharomyces kudriavzevii (strain VIN7) OX=1095631 GN=VIN7_3939 PE=3 SV=1 | 818.4 | 40754.3 | 40 | 4 | 17 | 14.65 | Green |
| C8ZFH4_YEAS8 | C8ZFH4 | Scw10p OS=Saccharomyces cerevisiae (strain Lalvin EC1118 / Prise de mousse) OX=643680 GN=EC1118_1M3_5215g PE=3 SV=1 | 818.4 | 40724.3 | 40 | 4 | 17 | 14.65 | Green |
| B3LMJ3_YEAS1 | B3LMJ3 | Soluble cell wall protein OS=Saccharomyces cerevisiae (strain RM11-1a) OX=285006 GN=SCRG_02202 PE=3 SV=1 | 818.4 | 40754.3 | 40 | 4 | 17 | 14.65 | Green |
| A0A6A5PQ89_YEASX | A0A6A5PQ89 | SCW10 isoform 1 OS=Saccharomyces cerevisiae OX=4932 GN=SCW10 PE=3 SV=1 | 818.4 | 40754.3 | 40 | 4 | 17 | 14.65 | Green |
| G2WL14_YEASK | G2WL14 | K7_Scw10p OS=Saccharomyces cerevisiae (strain Kyokai no. 7 / NBRC 101557) OX=721032 GN=K7_SCW10 PE=3 SV=1 | 818.4 | 40784.4 | 40 | 4 | 17 | 14.65 | Green |
| C7GPV8_YEAS2 | C7GPV8 | Scw10p OS=Saccharomyces cerevisiae (strain JAY291) OX=574961 GN=SCW10 PE=3 SV=1 | 818.4 | 40754.3 | 40 | 4 | 17 | 14.65 | Green |
| A6ZN21_YEAS7 | A6ZN21 | Soluble cell wall protein OS=Saccharomyces cerevisiae (strain YJM789) OX=307796 GN=SCW10 PE=3 SV=1 | 818.4 | 40754.3 | 40 | 4 | 17 | 14.65 | Green |
| SCW10_YEAST | Q04951 | Probable family 17 glucosidase SCW10 OS=Saccharomyces cerevisiae (strain ATCC 204508 / S288c) OX=559292 GN=SCW10 PE=1 SV=1 | 818.4 | 40754.3 | 41 | 4 | 17 | 14.65 | Green |
| B5VLC4_YEAS6 | B5VLC4 | glyceraldehyde-3-phosphate dehydrogenase (phosphorylating) (Fragment) OS=Saccharomyces cerevisiae (strain AWRI1631) OX=545124 GN=AWRI1631_101500 PE=3 SV=1 | 834.5 | 27906.9 | 25 | 2 | 25 | 14.5 | Green |
| B5VQ72_YEAS6 | B5VQ72 | 1_3-beta-glucanosyltransferase OS=Saccharomyces cerevisiae (strain AWRI1631) OX=545124 GN=AWRI1631_134450 PE=3 SV=1 | 112.0 | 49056.2 | 13 | 4 | 26 | 14.47 | Green |
| H0H0S1_SACCK | H0H0S1 | Adh1p OS=Saccharomyces cerevisiae x Saccharomyces kudriavzevii (strain VIN7) OX=1095631 GN=VIN7_9785 PE=3 SV=1 | 126.4 | 37264.4 | 21 | 3 | 24 | 14.37 | Green |
| ADH2_KLUMA | Q9P4C2 | Alcohol dehydrogenase 2 OS=Kluyveromyces marxianus OX=4911 GN=ADH2 PE=3 SV=3 | 138.8 | 37481.5 | 22 | 4 | 27 | 13.22 | Green |
| N1P0S2_YEASC | N1P0S2 | 1_3-beta-glucanosyltransferase OS=Saccharomyces cerevisiae (strain CEN.PK113-7D) OX=889517 GN=CENPK1137D_352 PE=3 SV=1 | 112.0 | 60380.8 | 13 | 4 | 34 | 11.81 | Green |
| H0GLJ8_SACCK | H0GLJ8 | 1_3-beta-glucanosyltransferase OS=Saccharomyces cerevisiae x Saccharomyces kudriavzevii (strain VIN7) OX=1095631 GN=VIN7_3941 PE=3 SV=1 | 112.0 | 60263.7 | 13 | 4 | 34 | 11.81 | Green |
| G2WL16_YEASK | G2WL16 | 1_3-beta-glucanosyltransferase OS=Saccharomyces cerevisiae (strain Kyokai no. 7 / NBRC 101557) OX=721032 GN=K7_GAS1 PE=3 SV=1 | 112.0 | 60278.7 | 13 | 4 | 34 | 11.81 | Green |
| C8ZFH7_YEAS8 | C8ZFH7 | 1_3-beta-glucanosyltransferase OS=Saccharomyces cerevisiae (strain Lalvin EC1118 / Prise de mousse) OX=643680 GN=EC1118_1M3_5248g PE=3 SV=1 | 112.0 | 60350.8 | 13 | 4 | 34 | 11.81 | Green |
| C7GPV6_YEAS2 | C7GPV6 | 1_3-beta-glucanosyltransferase OS=Saccharomyces cerevisiae (strain JAY291) OX=574961 GN=GAS1 PE=3 SV=1 | 112.0 | 60350.8 | 13 | 4 | 34 | 11.81 | Green |
| B3LMJ5_YEAS1 | B3LMJ5 | 1_3-beta-glucanosyltransferase OS=Saccharomyces cerevisiae (strain RM11-1a) OX=285006 GN=SCRG_02204 PE=3 SV=1 | 112.0 | 60350.8 | 13 | 4 | 34 | 11.81 | Green |
| A6ZN24_YEAS7 | A6ZN24 | 1_3-beta-glucanosyltransferase OS=Saccharomyces cerevisiae (strain YJM789) OX=307796 GN=GAS1 PE=3 SV=1 | 112.0 | 60350.8 | 13 | 4 | 34 | 11.81 | Green |
| A0A8H4BYZ6_YEASX | A0A8H4BYZ6 | 1_3-beta-glucanosyltransferase OS=Saccharomyces cerevisiae OX=4932 GN=GAS1 PE=3 SV=1 | 112.0 | 60380.8 | 13 | 4 | 34 | 11.81 | Green |
| A0A6C1EEC1_SACPS | A0A6C1EEC1 | 1_3-beta-glucanosyltransferase OS=Saccharomyces pastorianus OX=27292 GN=GAS1 PE=3 SV=1 | 112.0 | 60350.8 | 13 | 4 | 34 | 11.81 | Green |
| GAS1_YEAST | P22146 | 1_3-beta-glucanosyltransferase GAS1 OS=Saccharomyces cerevisiae (strain ATCC 204508 / S288c) OX=559292 GN=GAS1 PE=1 SV=2 | 112.0 | 60380.8 | 13 | 4 | 34 | 11.81 | Green |
| N1NZW1_YEASC | N1NZW1 | Glyceraldehyde-3-phosphate dehydrogenase OS=Saccharomyces cerevisiae (strain CEN.PK113-7D) OX=889517 GN=CENPK1137D_1249 PE=3 SV=1 | 834.5 | 35864.0 | 25 | 2 | 32 | 11.45 | Green |
| H0GWU4_SACCK | H0GWU4 | Glyceraldehyde-3-phosphate dehydrogenase OS=Saccharomyces cerevisiae x Saccharomyces kudriavzevii (strain VIN7) OX=1095631 GN=VIN7_7993 PE=3 SV=1 | 834.5 | 35832.0 | 25 | 2 | 32 | 11.45 | Green |
| H0GID8_SACCK | H0GID8 | Glyceraldehyde-3-phosphate dehydrogenase OS=Saccharomyces cerevisiae x Saccharomyces kudriavzevii (strain VIN7) OX=1095631 GN=VIN7_2630 PE=3 SV=1 | 834.5 | 35806.0 | 25 | 2 | 32 | 11.45 | Green |
| G2WGW5_YEASK | G2WGW5 | Glyceraldehyde-3-phosphate dehydrogenase OS=Saccharomyces cerevisiae (strain Kyokai no. 7 / NBRC 101557) OX=721032 GN=K7_TDH1 PE=3 SV=1 | 834.5 | 35864.0 | 25 | 2 | 32 | 11.45 | Green |
| C8ZBG2_YEAS8 | C8ZBG2 | Glyceraldehyde-3-phosphate dehydrogenase OS=Saccharomyces cerevisiae (strain Lalvin EC1118 / Prise de mousse) OX=643680 GN=EC1118_1J11_2025g PE=3 SV=2 | 834.5 | 35806.0 | 25 | 2 | 32 | 11.45 | Green |
| B3LQ59_YEAS1 | B3LQ59 | Glyceraldehyde-3-phosphate dehydrogenase OS=Saccharomyces cerevisiae (strain RM11-1a) OX=285006 GN=SCRG_03618 PE=3 SV=1 | 834.5 | 35806.0 | 25 | 2 | 32 | 11.45 | Green |
| A6ZPS3_YEAS7 | A6ZPS3 | Glyceraldehyde-3-phosphate dehydrogenase OS=Saccharomyces cerevisiae (strain YJM789) OX=307796 GN=TDH1 PE=3 SV=1 | 834.5 | 35806.0 | 25 | 2 | 32 | 11.45 | Green |
| A0A8H4BYL0_YEASX | A0A8H4BYL0 | Glyceraldehyde-3-phosphate dehydrogenase OS=Saccharomyces cerevisiae OX=4932 GN=TDH1 PE=3 SV=1 | 834.5 | 35864.0 | 25 | 2 | 32 | 11.45 | Green |
| A0A6C1DV71_SACPS | A0A6C1DV71 | Glyceraldehyde-3-phosphate dehydrogenase OS=Saccharomyces pastorianus OX=27292 GN=TDH1_1 PE=3 SV=1 | 834.5 | 35806.0 | 25 | 2 | 32 | 11.45 | Green |
| G3P1_YEAST | P00360 | Glyceraldehyde-3-phosphate dehydrogenase 1 OS=Saccharomyces cerevisiae (strain ATCC 204508 / S288c) OX=559292 GN=TDH1 PE=1 SV=3 | 834.5 | 35864.0 | 25 | 2 | 32 | 11.45 | Green |
| ENO_EREGS | Q756H2 | Enolase OS=Eremothecium gossypii (strain ATCC 10895 / CBS 109.51 / FGSC 9923 / NRRL Y-1056) OX=284811 GN=ENO PE=3 SV=1 | 1701.2 | 46688.9 | 57 | 2 | 35 | 11.44 | Green |
| A0A6C1EBI4_SACPS | A0A6C1EBI4 | Fructose-bisphosphate aldolase OS=Saccharomyces pastorianus OX=27292 GN=FBA1_2 PE=3 SV=1 | 245.4 | 39648.8 | 31 | 3 | 31 | 11.14 | Green |
| H0GVG6_SACCK | H0GVG6 | Scw4p OS=Saccharomyces cerevisiae x Saccharomyces kudriavzevii (strain VIN7) OX=1095631 GN=VIN7_7410 PE=4 SV=1 | 1490.4 | 32790.9 | 33 | 2 | 12 | 10.44 | Green |
| A0A6C1EF25_SACPS | A0A6C1EF25 | Putative 17 glucosidase scw10 OS=Saccharomyces pastorianus OX=27292 GN=SCW10_2 PE=4 SV=1 | 805.9 | 47511.4 | 35 | 3 | 21 | 10.14 | Green |
| F8KAB8_SACBA | F8KAB8 | Alcohol dehydrogenase (Fragment) OS=Saccharomyces bayanus OX=4931 GN=adh1 PE=3 SV=1 | 98.5 | 36041.8 | 18 | 3 | 22 | 8.9 | Green |
| F8KAA6_SACUV | F8KAA6 | Alcohol dehydrogenase (Fragment) OS=Saccharomyces uvarum OX=230603 GN=adh1 PE=3 SV=1 | 98.5 | 36025.8 | 18 | 3 | 22 | 8.9 | Green |
| A0A0B4VIV5_YEASX | A0A0B4VIV5 | Glucose-repressible alcohol dehydrogenase II (Fragment) OS=Saccharomyces cerevisiae OX=4932 GN=ADH2 PE=3 SV=1 | 84.7 | 34917.6 | 20 | 3 | 25 | 8.84 | Green |
| A0A0B4VIU1_YEASX | A0A0B4VIU1 | Glucose-repressible alcohol dehydrogenase II (Fragment) OS=Saccharomyces cerevisiae OX=4932 GN=ADH2 PE=3 SV=1 | 84.7 | 34918.6 | 20 | 3 | 25 | 8.84 | Green |
| A0A0B4VIL5_YEASX | A0A0B4VIL5 | Glucose-repressible alcohol dehydrogenase II (Fragment) OS=Saccharomyces cerevisiae OX=4932 GN=ADH2 PE=3 SV=1 | 84.7 | 34902.6 | 20 | 3 | 26 | 8.84 | Green |
| A0A0B4VIL2_YEASX | A0A0B4VIL2 | Glucose-repressible alcohol dehydrogenase II (Fragment) OS=Saccharomyces cerevisiae OX=4932 GN=ADH2 PE=3 SV=1 | 84.7 | 34946.6 | 20 | 3 | 27 | 8.84 | Green |
| A0A0B4VK10_YEASX | A0A0B4VK10 | Glucose-repressible alcohol dehydrogenase II (Fragment) OS=Saccharomyces cerevisiae OX=4932 GN=ADH2 PE=3 SV=1 | 84.7 | 36123.0 | 20 | 3 | 26 | 8.58 | Green |
| A0A0B4VJK0_YEASX | A0A0B4VJK0 | Glucose-repressible alcohol dehydrogenase II (Fragment) OS=Saccharomyces cerevisiae OX=4932 GN=ADH2 PE=3 SV=1 | 84.7 | 36139.0 | 20 | 3 | 25 | 8.58 | Green |
| A0A0B4VIM0_YEASX | A0A0B4VIM0 | Glucose-repressible alcohol dehydrogenase II (Fragment) OS=Saccharomyces cerevisiae OX=4932 GN=ADH2 PE=3 SV=1 | 84.7 | 36122.0 | 20 | 3 | 26 | 8.58 | Green |
| Q6XQ69_SACPS | Q6XQ69 | Alcohol dehydrogenase 2 OS=Saccharomyces pastorianus OX=27292 GN=ADH2 PE=3 SV=1 | 84.7 | 37200.2 | 20 | 3 | 28 | 8.33 | Green |
| M9VDX4_YEASX | M9VDX4 | Phenylacetaldehyde dehydrogenase OS=Saccharomyces cerevisiae OX=4932 PE=3 SV=1 | 84.7 | 37200.2 | 20 | 3 | 28 | 8.33 | Green |
| H0GLJ4_SACCK | H0GLJ4 | Adh2p OS=Saccharomyces cerevisiae x Saccharomyces kudriavzevii (strain VIN7) OX=1095631 GN=VIN7_3937 PE=3 SV=1 | 84.7 | 37200.2 | 20 | 3 | 28 | 8.33 | Green |
| G2WL12_YEASK | G2WL12 | K7_Adh2p OS=Saccharomyces cerevisiae (strain Kyokai no. 7 / NBRC 101557) OX=721032 GN=K7_ADH2 PE=3 SV=1 | 84.7 | 37215.2 | 20 | 3 | 27 | 8.33 | Green |
| C8ZFH1_YEAS8 | C8ZFH1 | Adh2p OS=Saccharomyces cerevisiae (strain Lalvin EC1118 / Prise de mousse) OX=643680 GN=EC1118_1M3_5182g PE=3 SV=1 | 84.7 | 37200.2 | 20 | 3 | 28 | 8.33 | Green |
| B3LMJ1_YEAS1 | B3LMJ1 | Alcohol dehydrogenase II OS=Saccharomyces cerevisiae (strain RM11-1a) OX=285006 GN=SCRG_02200 PE=3 SV=1 | 84.7 | 37170.2 | 20 | 3 | 24 | 8.33 | Green |
| A0A3G3NDG3_YEASX | A0A3G3NDG3 | Alcohol dehydrogenase OS=Saccharomyces cerevisiae OX=4932 GN=ADH1 PE=3 SV=1 | 84.7 | 37244.3 | 20 | 3 | 29 | 8.33 | Green |
| A0A0B4VJL3_YEASX | A0A0B4VJL3 | ADH2 isoform 1 OS=Saccharomyces cerevisiae OX=4932 GN=ADH2 PE=3 SV=1 | 84.7 | 37188.2 | 20 | 3 | 27 | 8.33 | Green |
| ADH2_YEAST | P00331 | Alcohol dehydrogenase 2 OS=Saccharomyces cerevisiae (strain ATCC 204508 / S288c) OX=559292 GN=ADH2 PE=1 SV=3 | 84.7 | 37188.2 | 20 | 3 | 27 | 8.33 | Green |
| A0A6C1DY36_SACPS | A0A6C1DY36 | Putative 17 glucosidase scw10 OS=Saccharomyces pastorianus OX=27292 GN=SCW10_1 PE=3 SV=1 | 61.0 | 38194.7 | 16 | 2 | 16 | 8.24 | Green |
| X5CPF8_SACPS | X5CPF8 | Cytochrome b-c1 complex subunit Rieske_ mitochondrial (Fragment) OS=Saccharomyces pastorianus OX=27292 GN=RIP1 PE=3 SV=1 | 162.4 | 19345.0 | 9 | 1 | 13 | 6.82 | Green |
| A0A061BBA7_CYBFA | A0A061BBA7 | Superoxide dismutase OS=Cyberlindnera fabianii OX=36022 GN=BON22_4688 PE=3 SV=1 | 207.3 | 24273.2 | 13 | 2 | 13 | 6.48 | Green |
| W8P1Y9_9SACH | W8P1Y9 | Cytochrome b-c1 complex subunit Rieske_ mitochondrial (Fragment) OS=Saccharomyces cerevisiae x Saccharomyces eubayanus x Saccharomyces uvarum OX=1470621 GN=RIP1 PE=3 SV=1 | 162.4 | 20495.5 | 9 | 1 | 15 | 6.45 | Green |
| H0GEX1_SACCK | H0GEX1 | Cytochrome b-c1 complex subunit Rieske_ mitochondrial OS=Saccharomyces cerevisiae x Saccharomyces kudriavzevii (strain VIN7) OX=1095631 GN=VIN7_1266 PE=3 SV=1 | 171.1 | 22060.2 | 12 | 2 | 16 | 5.97 | Green |
| N1P7D2_YEASC | N1P7D2 | Cytochrome b-c1 complex subunit Rieske_ mitochondrial OS=Saccharomyces cerevisiae (strain CEN.PK113-7D) OX=889517 GN=CENPK1137D_3630 PE=3 SV=1 | 171.1 | 23650.1 | 12 | 2 | 16 | 5.58 | Green |
| G2WCK7_YEASK | G2WCK7 | Cytochrome b-c1 complex subunit Rieske_ mitochondrial OS=Saccharomyces cerevisiae (strain Kyokai no. 7 / NBRC 101557) OX=721032 GN=K7_RIP1 PE=3 SV=1 | 171.1 | 23636.1 | 12 | 2 | 16 | 5.58 | Green |
| C8Z6X3_YEAS8 | C8Z6X3 | Cytochrome b-c1 complex subunit Rieske_ mitochondrial OS=Saccharomyces cerevisiae (strain Lalvin EC1118 / Prise de mousse) OX=643680 GN=EC1118_1E8_0606g PE=3 SV=1 | 171.1 | 23636.1 | 12 | 2 | 16 | 5.58 | Green |
| C7GX18_YEAS2 | C7GX18 | Cytochrome b-c1 complex subunit Rieske_ mitochondrial OS=Saccharomyces cerevisiae (strain JAY291) OX=574961 GN=RIP1 PE=3 SV=1 | 171.1 | 23636.1 | 12 | 2 | 16 | 5.58 | Green |
| B5VHB1_YEAS6 | B5VHB1 | Cytochrome b-c1 complex subunit Rieske_ mitochondrial OS=Saccharomyces cerevisiae (strain AWRI1631) OX=545124 GN=AWRI1631_50460 PE=3 SV=1 | 171.1 | 23636.1 | 12 | 2 | 16 | 5.58 | Green |
| B3LRZ1_YEAS1 | B3LRZ1 | Cytochrome b-c1 complex subunit Rieske_ mitochondrial OS=Saccharomyces cerevisiae (strain RM11-1a) OX=285006 GN=SCRG_04444 PE=3 SV=1 | 171.1 | 23636.1 | 12 | 2 | 16 | 5.58 | Green |
| A6ZQS4_YEAS7 | A6ZQS4 | Cytochrome b-c1 complex subunit Rieske_ mitochondrial OS=Saccharomyces cerevisiae (strain YJM789) OX=307796 GN=RIP1 PE=3 SV=1 | 171.1 | 23636.1 | 12 | 2 | 16 | 5.58 | Green |
| A0A8H8ULJ0_YEASX | A0A8H8ULJ0 | Cytochrome b-c1 complex subunit Rieske_ mitochondrial OS=Saccharomyces cerevisiae OX=4932 GN=RIP1 PE=3 SV=1 | 171.1 | 23650.1 | 12 | 2 | 16 | 5.58 | Green |
| A0A6C1DQ93_SACPS | A0A6C1DQ93 | Cytochrome b-c1 complex subunit Rieske_ mitochondrial OS=Saccharomyces pastorianus OX=27292 GN=RIP1_1 PE=3 SV=1 | 171.1 | 23636.1 | 12 | 2 | 16 | 5.58 | Green |
| UCRI_YEAST | P08067 | Cytochrome b-c1 complex subunit Rieske_ mitochondrial OS=Saccharomyces cerevisiae (strain ATCC 204508 / S288c) OX=559292 GN=RIP1 PE=1 SV=1 | 171.1 | 23650.1 | 12 | 2 | 16 | 5.58 | Green |
| H0GTN9_SACCK | H0GTN9 | Cytochrome b-c1 complex subunit Rieske_ mitochondrial OS=Saccharomyces cerevisiae x Saccharomyces kudriavzevii (strain VIN7) OX=1095631 GN=VIN7_6628 PE=3 SV=1 | 162.4 | 23705.1 | 9 | 1 | 16 | 5.58 | Green |
| A0A6C1E7I5_SACPS | A0A6C1E7I5 | Cytochrome b-c1 complex subunit Rieske_ mitochondrial OS=Saccharomyces pastorianus OX=27292 GN=RIP1_2 PE=3 SV=1 | 162.4 | 23733.2 | 9 | 1 | 16 | 5.58 | Green |
| A0A1X7R3K1_9SACH | A0A1X7R3K1 | Similar to Saccharomyces cerevisiae YDR530C APA2 Diadenosine 5'_5''-P1_P4-tetraphosphate phosphorylase II (AP4A phosphorylase) OS=Kazachstania saulgeensis OX=1789683 GN=KASA_0N00275G PE=4 SV=1 | 79.0 | 36352.5 | 10 | 3 | 33 | 2.52 | Green |
| A0A8H2ZIS5_9SACH | A0A8H2ZIS5 | Similar to Saccharomyces cerevisiae YDR530C APA2 Diadenosine 5'_5''-P1_P4- tetraphosphate phosphorylase II (AP4A phosphorylase)_ involved in catabolism of bis(5'-nucleosidyl) tetraphosphates OS=Kazachstania barnettii OX=61262 GN=KABA2_02S17270 PE=4 SV=1 | 63.9 | 36171.1 | 6 | 1 | 27 | 2.52 | Green |
| Q6XQ70_SACPS | Q6XQ70 | Alcohol dehydrogenase 1 (Fragment) OS=Saccharomyces pastorianus OX=27292 GN=ADH1 PE=3 SV=2 | 61.4 | 32097.3 | 21 | 3 | 19 | 0 | Green |
| **Proteins in EV@Y72** | | | | | | | | | |
| **Entry** | **Accession** | **Description** | **score** | **avgMass** | **Matched Products** | **matched Peptides** | **digestPeps** | **seq Cover(%)** | **Auto Curate** |
| H0GHK6_SACCK | H0GHK6 | phosphopyruvate hydratase OS=Saccharomyces cerevisiae x Saccharomyces kudriavzevii (strain VIN7) OX=1095631 GN=VIN7_2265 PE=3 SV=1 | 6453.5 | 46971.3 | 298 | 24 | 35 | 62.93 | Green |
| N1P5V1_YEASC | N1P5V1 | Phosphatidylglycerol/phosphatidylinositol transfer protein OS=Saccharomyces cerevisiae (strain CEN.PK113-7D) OX=889517 GN=CENPK1137D_3781 PE=3 SV=1 | 598.2 | 19291.0 | 44 | 8 | 12 | 62.43 | Green |
| G2WCB4_YEASK | G2WCB4 | Phosphatidylglycerol/phosphatidylinositol transfer protein OS=Saccharomyces cerevisiae (strain Kyokai no. 7 / NBRC 101557) OX=721032 GN=K7_NPC2 PE=3 SV=1 | 598.2 | 19261.0 | 44 | 8 | 12 | 62.43 | Green |
| A6ZXS8_YEAS7 | A6ZXS8 | Phosphatidylglycerol/phosphatidylinositol transfer protein OS=Saccharomyces cerevisiae (strain YJM789) OX=307796 GN=NPC2 PE=3 SV=1 | 598.2 | 19261.0 | 44 | 8 | 12 | 62.43 | Green |
| A0A8H8UMN4_YEASX | A0A8H8UMN4 | Phosphatidylglycerol/phosphatidylinositol transfer protein OS=Saccharomyces cerevisiae OX=4932 GN=NPC2 PE=3 SV=1 | 598.2 | 19291.0 | 44 | 8 | 12 | 62.43 | Green |
| NPC2_YEAST | Q12408 | Phosphatidylglycerol/phosphatidylinositol transfer protein OS=Saccharomyces cerevisiae (strain ATCC 204508 / S288c) OX=559292 GN=NPC2 PE=1 SV=1 | 598.2 | 19291.0 | 46 | 8 | 12 | 62.43 | Green |
| G2WFP7_YEASK | G2WFP7 | phosphopyruvate hydratase OS=Saccharomyces cerevisiae (strain Kyokai no. 7 / NBRC 101557) OX=721032 GN=K7_ENO2 PE=3 SV=1 | 5307.7 | 46971.3 | 243 | 21 | 34 | 60.18 | Green |
| B3LST6_YEAS1 | B3LST6 | phosphopyruvate hydratase OS=Saccharomyces cerevisiae (strain RM11-1a) OX=285006 GN=SCRG_04885 PE=3 SV=1 | 5307.7 | 46971.3 | 243 | 21 | 34 | 60.18 | Green |
| A6ZT81_YEAS7 | A6ZT81 | phosphopyruvate hydratase OS=Saccharomyces cerevisiae (strain YJM789) OX=307796 GN=ENO2 PE=3 SV=1 | 5307.7 | 46971.3 | 243 | 21 | 34 | 60.18 | Green |
| A0A6C1DSX5_SACPS | A0A6C1DSX5 | phosphopyruvate hydratase OS=Saccharomyces pastorianus OX=27292 GN=ENO2 PE=3 SV=1 | 5307.7 | 46971.3 | 243 | 21 | 34 | 60.18 | Green |
| A0A6A5PY35_YEASX | A0A6A5PY35 | phosphopyruvate hydratase OS=Saccharomyces cerevisiae OX=4932 GN=ENO2 PE=3 SV=1 | 5307.7 | 46971.3 | 243 | 21 | 34 | 60.18 | Green |
| ENO2_YEAST | P00925 | Enolase 2 OS=Saccharomyces cerevisiae (strain ATCC 204508 / S288c) OX=559292 GN=ENO2 PE=1 SV=2 | 5307.7 | 46971.3 | 246 | 21 | 34 | 60.18 | Green |
| B5VJJ6_YEAS6 | B5VJJ6 | phosphopyruvate hydratase OS=Saccharomyces cerevisiae (strain AWRI1631) OX=545124 GN=AWRI1631_74770 PE=3 SV=1 | 6148.3 | 41765.5 | 237 | 18 | 33 | 60.15 | Green |
| N1P439_YEASC | N1P439 | phosphopyruvate hydratase OS=Saccharomyces cerevisiae (strain CEN.PK113-7D) OX=889517 GN=CENPK1137D_3221 PE=3 SV=1 | 6168.8 | 46873.2 | 241 | 19 | 36 | 56.52 | Green |
| H0GGY4_SACCK | H0GGY4 | phosphopyruvate hydratase OS=Saccharomyces cerevisiae x Saccharomyces kudriavzevii (strain VIN7) OX=1095631 GN=VIN7_2021 PE=3 SV=1 | 6168.8 | 46859.2 | 241 | 19 | 36 | 56.52 | Green |
| G2WEY0_YEASK | G2WEY0 | phosphopyruvate hydratase OS=Saccharomyces cerevisiae (strain Kyokai no. 7 / NBRC 101557) OX=721032 GN=K7_ENO1 PE=3 SV=1 | 6168.8 | 46859.2 | 241 | 19 | 36 | 56.52 | Green |
| C8Z9E6_YEAS8 | C8Z9E6 | phosphopyruvate hydratase OS=Saccharomyces cerevisiae (strain Lalvin EC1118 / Prise de mousse) OX=643680 GN=EC1118_1G1_5908g PE=3 SV=1 | 6168.8 | 46859.2 | 241 | 19 | 36 | 56.52 | Green |
| B3LHY6_YEAS1 | B3LHY6 | phosphopyruvate hydratase OS=Saccharomyces cerevisiae (strain RM11-1a) OX=285006 GN=SCRG_00770 PE=3 SV=1 | 6168.8 | 46873.2 | 241 | 19 | 36 | 56.52 | Green |
| A6ZUR2_YEAS7 | A6ZUR2 | phosphopyruvate hydratase OS=Saccharomyces cerevisiae (strain YJM789) OX=307796 GN=ENO1 PE=3 SV=1 | 6168.8 | 46859.2 | 241 | 19 | 36 | 56.52 | Green |
| A0A8H8UKY7_YEASX | A0A8H8UKY7 | phosphopyruvate hydratase OS=Saccharomyces cerevisiae OX=4932 GN=ENO1 PE=3 SV=1 | 6168.8 | 46873.2 | 241 | 19 | 36 | 56.52 | Green |
| A0A6C1DT43_SACPS | A0A6C1DT43 | phosphopyruvate hydratase OS=Saccharomyces pastorianus OX=27292 GN=ENO1_1 PE=3 SV=1 | 6168.8 | 46859.2 | 241 | 19 | 36 | 56.52 | Green |
| ENO1_YEAST | P00924 | Enolase 1 OS=Saccharomyces cerevisiae (strain ATCC 204508 / S288c) OX=559292 GN=ENO1 PE=1 SV=3 | 6168.8 | 46873.2 | 246 | 19 | 36 | 56.52 | Green |
| N1P2H7_YEASC | N1P2H7 | Glyceraldehyde-3-phosphate dehydrogenase OS=Saccharomyces cerevisiae (strain CEN.PK113-7D) OX=889517 GN=CENPK1137D_3157 PE=3 SV=1 | 2293.5 | 35860.8 | 161 | 14 | 31 | 54.52 | Green |
| G2WES0_YEASK | G2WES0 | Glyceraldehyde-3-phosphate dehydrogenase OS=Saccharomyces cerevisiae (strain Kyokai no. 7 / NBRC 101557) OX=721032 GN=K7_TDH3 PE=3 SV=1 | 2293.5 | 35860.8 | 161 | 14 | 31 | 54.52 | Green |
| A0A6A5PYU8_YEASX | A0A6A5PYU8 | Glyceraldehyde-3-phosphate dehydrogenase OS=Saccharomyces cerevisiae OX=4932 GN=TDH3 PE=3 SV=1 | 2293.5 | 35860.8 | 161 | 14 | 31 | 54.52 | Green |
| G3P3_YEAST | P00359 | Glyceraldehyde-3-phosphate dehydrogenase 3 OS=Saccharomyces cerevisiae (strain ATCC 204508 / S288c) OX=559292 GN=TDH3 PE=1 SV=3 | 2293.5 | 35860.8 | 161 | 14 | 31 | 54.52 | Green |
| H0GVE8_SACCK | H0GVE8 | phosphopyruvate hydratase OS=Saccharomyces cerevisiae x Saccharomyces kudriavzevii (strain VIN7) OX=1095631 GN=VIN7_7387 PE=3 SV=1 | 4921.7 | 46886.3 | 189 | 17 | 35 | 54.46 | Green |
| H0GW01_SACCK | H0GW01 | phosphopyruvate hydratase OS=Saccharomyces cerevisiae x Saccharomyces kudriavzevii (strain VIN7) OX=1095631 GN=VIN7_7633 PE=3 SV=1 | 6790.4 | 46811.0 | 280 | 20 | 32 | 51.95 | Green |
| H0GDT7_SACCK | H0GDT7 | Phosphatidylglycerol/phosphatidylinositol transfer protein OS=Saccharomyces cerevisiae x Saccharomyces kudriavzevii (strain VIN7) OX=1095631 GN=VIN7_0703 PE=3 SV=1 | 533.4 | 19246.9 | 37 | 7 | 12 | 50.29 | Green |
| C8Z4M3_YEAS8 | C8Z4M3 | Phosphatidylglycerol/phosphatidylinositol transfer protein OS=Saccharomyces cerevisiae (strain Lalvin EC1118 / Prise de mousse) OX=643680 GN=EC1118_1D0_1849g PE=3 SV=1 | 533.4 | 19246.9 | 37 | 7 | 12 | 50.29 | Green |
| C7GPW6_YEAS2 | C7GPW6 | Phosphatidylglycerol/phosphatidylinositol transfer protein OS=Saccharomyces cerevisiae (strain JAY291) OX=574961 GN=NPC2 PE=3 SV=1 | 533.4 | 19246.9 | 37 | 7 | 12 | 50.29 | Green |
| B5VFM1_YEAS6 | B5VFM1 | Phosphatidylglycerol/phosphatidylinositol transfer protein OS=Saccharomyces cerevisiae (strain AWRI1631) OX=545124 GN=AWRI1631_41920 PE=3 SV=1 | 533.4 | 19246.9 | 37 | 7 | 12 | 50.29 | Green |
| B3LGU5_YEAS1 | B3LGU5 | Phosphatidylglycerol/phosphatidylinositol transfer protein OS=Saccharomyces cerevisiae (strain RM11-1a) OX=285006 GN=SCRG_00546 PE=3 SV=1 | 533.4 | 19246.9 | 37 | 7 | 12 | 50.29 | Green |
| A0A6C1DN60_SACPS | A0A6C1DN60 | Phosphatidylglycerol/phosphatidylinositol transfer protein OS=Saccharomyces pastorianus OX=27292 GN=NPC2_1 PE=3 SV=1 | 533.4 | 19246.9 | 37 | 7 | 12 | 50.29 | Green |
| B5VI89_YEAS6 | B5VI89 | Phosphotransferase (Fragment) OS=Saccharomyces cerevisiae (strain AWRI1631) OX=545124 GN=AWRI1631_70090 PE=3 SV=1 | 703.7 | 38997.1 | 62 | 9 | 25 | 50 | Green |
| A0A6C1E8Q2_SACPS | A0A6C1E8Q2 | phosphopyruvate hydratase OS=Saccharomyces pastorianus OX=27292 GN=ENO1_2 PE=3 SV=1 | 2423.8 | 46784.1 | 114 | 14 | 36 | 49.43 | Green |
| H0GGT7_SACCK | H0GGT7 | Glyceraldehyde-3-phosphate dehydrogenase OS=Saccharomyces cerevisiae x Saccharomyces kudriavzevii (strain VIN7) OX=1095631 GN=VIN7_1968 PE=3 SV=1 | 2132.7 | 35847.7 | 125 | 11 | 31 | 48.19 | Green |
| C8Z985_YEAS8 | C8Z985 | Glyceraldehyde-3-phosphate dehydrogenase OS=Saccharomyces cerevisiae (strain Lalvin EC1118 / Prise de mousse) OX=643680 GN=EC1118_1G1_5204g PE=3 SV=1 | 2132.7 | 35847.7 | 125 | 11 | 31 | 48.19 | Green |
| B5VJD4_YEAS6 | B5VJD4 | Glyceraldehyde-3-phosphate dehydrogenase OS=Saccharomyces cerevisiae (strain AWRI1631) OX=545124 GN=AWRI1631_74130 PE=3 SV=1 | 2132.7 | 35847.7 | 125 | 11 | 31 | 48.19 | Green |
| B3LI45_YEAS1 | B3LI45 | Glyceraldehyde-3-phosphate dehydrogenase OS=Saccharomyces cerevisiae (strain RM11-1a) OX=285006 GN=SCRG_00830 PE=3 SV=1 | 2132.7 | 35847.7 | 125 | 11 | 31 | 48.19 | Green |
| A6ZUK2_YEAS7 | A6ZUK2 | Glyceraldehyde-3-phosphate dehydrogenase OS=Saccharomyces cerevisiae (strain YJM789) OX=307796 GN=TDH3 PE=3 SV=1 | 2132.7 | 35847.7 | 125 | 11 | 31 | 48.19 | Green |
| A0A6C1DS61_SACPS | A0A6C1DS61 | Glyceraldehyde-3-phosphate dehydrogenase OS=Saccharomyces pastorianus OX=27292 GN=TDH3 PE=3 SV=1 | 1853.4 | 35861.8 | 106 | 8 | 31 | 48.19 | Green |
| N1P4P1_YEASC | N1P4P1 | Phosphotransferase OS=Saccharomyces cerevisiae (strain CEN.PK113-7D) OX=889517 GN=CENPK1137D_3299 PE=3 SV=1 | 792.0 | 54170.7 | 93 | 14 | 40 | 48.15 | Green |
| G2WDK7_YEASK | G2WDK7 | Phosphotransferase OS=Saccharomyces cerevisiae (strain Kyokai no. 7 / NBRC 101557) OX=721032 GN=K7_HXK2 PE=3 SV=1 | 792.0 | 54170.7 | 93 | 14 | 40 | 48.15 | Green |
| C7GNZ9_YEAS2 | C7GNZ9 | Phosphotransferase OS=Saccharomyces cerevisiae (strain JAY291) OX=574961 GN=HXK2 PE=3 SV=1 | 792.0 | 54170.7 | 93 | 14 | 40 | 48.15 | Green |
| B3LHU5_YEAS1 | B3LHU5 | Phosphotransferase OS=Saccharomyces cerevisiae (strain RM11-1a) OX=285006 GN=SCRG_01237 PE=3 SV=1 | 792.0 | 54170.7 | 93 | 14 | 40 | 48.15 | Green |
| A6ZTT8_YEAS7 | A6ZTT8 | Phosphotransferase OS=Saccharomyces cerevisiae (strain YJM789) OX=307796 GN=HXK2 PE=3 SV=1 | 792.0 | 54170.7 | 93 | 14 | 40 | 48.15 | Green |
| A0A6A5PUV0_YEASX | A0A6A5PUV0 | Phosphotransferase OS=Saccharomyces cerevisiae OX=4932 GN=HXK2 PE=3 SV=1 | 792.0 | 54170.7 | 93 | 14 | 40 | 48.15 | Green |
| HXKB_YEAST | P04807 | Hexokinase-2 OS=Saccharomyces cerevisiae (strain ATCC 204508 / S288c) OX=559292 GN=HXK2 PE=1 SV=4 | 792.0 | 54170.7 | 93 | 14 | 40 | 48.15 | Green |
| N1P465_YEASC | N1P465 | Scw4p OS=Saccharomyces cerevisiae (strain CEN.PK113-7D) OX=889517 GN=CENPK1137D_3246 PE=3 SV=1 | 2630.3 | 40458.2 | 151 | 13 | 21 | 47.15 | Green |
| A0A8H8UL09_YEASX | A0A8H8UL09 | SCW4 isoform 1 OS=Saccharomyces cerevisiae OX=4932 GN=SCW4 PE=3 SV=1 | 2630.3 | 40458.2 | 151 | 13 | 21 | 47.15 | Green |
| SCW4_YEAST | P53334 | Probable family 17 glucosidase SCW4 OS=Saccharomyces cerevisiae (strain ATCC 204508 / S288c) OX=559292 GN=SCW4 PE=1 SV=1 | 2630.3 | 40458.2 | 151 | 13 | 21 | 47.15 | Green |
| S5S176_YEASX | S5S176 | Alcohol dehydrogenase OS=Saccharomyces cerevisiae OX=4932 GN=ADH1 PE=3 SV=1 | 566.1 | 37307.4 | 71 | 13 | 26 | 46.55 | Green |
| S5RZC2_YEASX | S5RZC2 | Alcohol dehydrogenase OS=Saccharomyces cerevisiae OX=4932 GN=ADH1 PE=1 SV=1 | 566.1 | 37295.4 | 71 | 13 | 26 | 46.55 | Green |
| S5RK20_YEASX | S5RK20 | Alcohol dehydrogenase OS=Saccharomyces cerevisiae OX=4932 GN=ADH1 PE=3 SV=1 | 566.1 | 37293.4 | 71 | 13 | 26 | 46.55 | Green |
| S5RCH3_YEASX | S5RCH3 | Alcohol dehydrogenase OS=Saccharomyces cerevisiae OX=4932 GN=ADH1 PE=3 SV=1 | 566.1 | 37294.4 | 71 | 13 | 26 | 46.55 | Green |
| H0GN42_SACCK | H0GN42 | Adh1p OS=Saccharomyces cerevisiae x Saccharomyces kudriavzevii (strain VIN7) OX=1095631 GN=VIN7_4366 PE=3 SV=1 | 566.1 | 37208.3 | 71 | 13 | 25 | 46.55 | Green |
| G2WMF0_YEASK | G2WMF0 | K7_Adh1p OS=Saccharomyces cerevisiae (strain Kyokai no. 7 / NBRC 101557) OX=721032 GN=K7_ADH1 PE=3 SV=1 | 566.1 | 37295.4 | 71 | 13 | 26 | 46.55 | Green |
| C8ZHN0_YEAS8 | C8ZHN0 | Adh1p OS=Saccharomyces cerevisiae (strain Lalvin EC1118 / Prise de mousse) OX=643680 GN=EC1118_1O4_0859g PE=3 SV=1 | 566.1 | 37307.4 | 71 | 13 | 26 | 46.55 | Green |
| B6UQD0_SACPS | B6UQD0 | Alcohol dehydrogenase OS=Saccharomyces pastorianus OX=27292 GN=ADH1 PE=3 SV=1 | 566.1 | 37295.4 | 71 | 13 | 26 | 46.55 | Green |
| B5VRG9_YEAS6 | B5VRG9 | YOL086Cp-like protein OS=Saccharomyces cerevisiae (strain AWRI1631) OX=545124 GN=AWRI1631_150730 PE=3 SV=1 | 566.1 | 37295.4 | 71 | 13 | 26 | 46.55 | Green |
| B3LIX8_YEAS1 | B3LIX8 | Alcohol dehydrogenase OS=Saccharomyces cerevisiae (strain RM11-1a) OX=285006 GN=SCRG_01319 PE=3 SV=1 | 566.1 | 37307.4 | 71 | 13 | 26 | 46.55 | Green |
| A6ZNC5_YEAS7 | A6ZNC5 | Alcohol dehydrogenase OS=Saccharomyces cerevisiae (strain YJM789) OX=307796 GN=ADH1 PE=3 SV=1 | 566.1 | 37295.4 | 71 | 13 | 26 | 46.55 | Green |
| A0A8H4BVB5_YEASX | A0A8H4BVB5 | ADH1 isoform 1 OS=Saccharomyces cerevisiae OX=4932 GN=ADH1 PE=3 SV=1 | 566.1 | 37305.4 | 71 | 13 | 26 | 46.55 | Green |
| ADH1_YEAST | P00330 | Alcohol dehydrogenase 1 OS=Saccharomyces cerevisiae (strain ATCC 204508 / S288c) OX=559292 GN=ADH1 PE=1 SV=5 | 566.1 | 37305.4 | 72 | 13 | 26 | 46.55 | Green |
| B5VP98_YEAS6 | B5VP98 | YML028Wp-like protein (Fragment) OS=Saccharomyces cerevisiae (strain AWRI1631) OX=545124 GN=AWRI1631_131090 PE=4 SV=1 | 233.1 | 13839.9 | 30 | 3 | 14 | 45.24 | Green |
| H0GFX8_SACCK | H0GFX8 | Phosphotransferase OS=Saccharomyces cerevisiae x Saccharomyces kudriavzevii (strain VIN7) OX=1095631 GN=VIN7_1585 PE=3 SV=1 | 771.3 | 54156.7 | 93 | 13 | 40 | 44.44 | Green |
| C8Z805_YEAS8 | C8Z805 | Phosphotransferase OS=Saccharomyces cerevisiae (strain Lalvin EC1118 / Prise de mousse) OX=643680 GN=EC1118_1G1_0144g PE=3 SV=1 | 771.3 | 54156.7 | 93 | 13 | 40 | 44.44 | Green |
| A0A3G3NDH9_YEASX | A0A3G3NDH9 | Alcohol dehydrogenase OS=Saccharomyces cerevisiae OX=4932 GN=ADH1 PE=3 SV=1 | 404.9 | 37278.4 | 61 | 12 | 26 | 42.82 | Green |
| M9VEX7_YEAS8 | M9VEX7 | Phenylacetaldehyde dehydrogenase OS=Saccharomyces cerevisiae (strain Lalvin EC1118 / Prise de mousse) OX=643680 PE=3 SV=1 | 556.7 | 37306.5 | 66 | 12 | 26 | 42.24 | Green |
| G2WF02_YEASK | G2WF02 | K7_Scw4p OS=Saccharomyces cerevisiae (strain Kyokai no. 7 / NBRC 101557) OX=721032 GN=K7_SCW4 PE=3 SV=1 | 2072.4 | 40459.2 | 125 | 12 | 21 | 42.23 | Green |
| C7GNF6_YEAS2 | C7GNF6 | Scw4p OS=Saccharomyces cerevisiae (strain JAY291) OX=574961 GN=SCW4 PE=3 SV=1 | 2072.4 | 40459.2 | 125 | 12 | 21 | 42.23 | Green |
| A6ZUT5_YEAS7 | A6ZUT5 | Soluble cell wall protein OS=Saccharomyces cerevisiae (strain YJM789) OX=307796 GN=SCW4 PE=3 SV=1 | 2072.4 | 40459.2 | 125 | 12 | 21 | 42.23 | Green |
| A0A6C1E673_SACPS | A0A6C1E673 | Phosphotransferase OS=Saccharomyces pastorianus OX=27292 GN=HXK2 PE=3 SV=1 | 702.2 | 54143.7 | 83 | 13 | 40 | 42.18 | Green |
| A0A6C1EGF6_SACPS | A0A6C1EGF6 | phosphopyruvate hydratase OS=Saccharomyces pastorianus OX=27292 GN=ENO1_3 PE=3 SV=1 | 1669.9 | 46688.9 | 108 | 12 | 34 | 41.42 | Green |
| H0GH04_SACCK | H0GH04 | Scw4p OS=Saccharomyces cerevisiae x Saccharomyces kudriavzevii (strain VIN7) OX=1095631 GN=VIN7_2044 PE=3 SV=1 | 2066.1 | 40445.2 | 111 | 10 | 21 | 40.16 | Green |
| C8Z9H2_YEAS8 | C8Z9H2 | Scw4p OS=Saccharomyces cerevisiae (strain Lalvin EC1118 / Prise de mousse) OX=643680 GN=EC1118_1G1_6194g PE=3 SV=1 | 2066.1 | 40445.2 | 111 | 10 | 21 | 40.16 | Green |
| B3LHW6_YEAS1 | B3LHW6 | Soluble cell wall protein OS=Saccharomyces cerevisiae (strain RM11-1a) OX=285006 GN=SCRG_00748 PE=3 SV=1 | 2066.1 | 40445.2 | 111 | 10 | 21 | 40.16 | Green |
| A0A6C1DSE2_SACPS | A0A6C1DSE2 | Glycoside hydrolase_ 17 OS=Saccharomyces pastorianus OX=27292 GN=SCW4_1 PE=3 SV=1 | 2066.1 | 40445.2 | 111 | 10 | 21 | 40.16 | Green |
| N1P8B2_YEASC | N1P8B2 | Phosphoglycerate mutase OS=Saccharomyces cerevisiae (strain CEN.PK113-7D) OX=889517 GN=CENPK1137D_1155 PE=3 SV=1 | 551.2 | 27608.6 | 46 | 5 | 21 | 39.27 | Green |
| H0GJ01_SACCK | H0GJ01 | Phosphoglycerate mutase OS=Saccharomyces cerevisiae x Saccharomyces kudriavzevii (strain VIN7) OX=1095631 GN=VIN7_2869 PE=3 SV=1 | 551.2 | 27608.6 | 46 | 5 | 21 | 39.27 | Green |
| G2WHN7_YEASK | G2WHN7 | Phosphoglycerate mutase OS=Saccharomyces cerevisiae (strain Kyokai no. 7 / NBRC 101557) OX=721032 GN=K7_GPM1 PE=3 SV=1 | 551.2 | 27608.6 | 46 | 5 | 21 | 39.27 | Green |
| C8ZC23_YEAS8 | C8ZC23 | Phosphoglycerate mutase OS=Saccharomyces cerevisiae (strain Lalvin EC1118 / Prise de mousse) OX=643680 GN=EC1118_1K5_0760g PE=3 SV=1 | 551.2 | 27608.6 | 46 | 5 | 21 | 39.27 | Green |
| C7GU53_YEAS2 | C7GU53 | Phosphoglycerate mutase OS=Saccharomyces cerevisiae (strain JAY291) OX=574961 GN=GPM1 PE=3 SV=1 | 551.2 | 27608.6 | 46 | 5 | 21 | 39.27 | Green |
| B3LQU9_YEAS1 | B3LQU9 | Phosphoglycerate mutase OS=Saccharomyces cerevisiae (strain RM11-1a) OX=285006 GN=SCRG_03874 PE=3 SV=1 | 551.2 | 27608.6 | 46 | 5 | 21 | 39.27 | Green |
| A6ZZH0_YEAS7 | A6ZZH0 | Phosphoglycerate mutase OS=Saccharomyces cerevisiae (strain YJM789) OX=307796 GN=GPM1 PE=3 SV=1 | 551.2 | 27608.6 | 46 | 5 | 21 | 39.27 | Green |
| A0A6C1DUX7_SACPS | A0A6C1DUX7 | Phosphoglycerate mutase OS=Saccharomyces pastorianus OX=27292 GN=GPM1_1 PE=3 SV=1 | 551.2 | 27608.6 | 46 | 5 | 21 | 39.27 | Green |
| A0A6A5PSG4_YEASX | A0A6A5PSG4 | Phosphoglycerate mutase OS=Saccharomyces cerevisiae OX=4932 GN=GPM1 PE=3 SV=1 | 551.2 | 27608.6 | 46 | 5 | 21 | 39.27 | Green |
| PMG1_YEAST | P00950 | Phosphoglycerate mutase 1 OS=Saccharomyces cerevisiae (strain ATCC 204508 / S288c) OX=559292 GN=GPM1 PE=1 SV=3 | 551.2 | 27608.6 | 46 | 5 | 21 | 39.27 | Green |
| N1P004_YEASC | N1P004 | Glyceraldehyde-3-phosphate dehydrogenase OS=Saccharomyces cerevisiae (strain CEN.PK113-7D) OX=889517 GN=CENPK1137D_1304 PE=3 SV=1 | 1367.6 | 35961.0 | 99 | 11 | 31 | 38.55 | Green |
| G2WH19_YEASK | G2WH19 | Glyceraldehyde-3-phosphate dehydrogenase OS=Saccharomyces cerevisiae (strain Kyokai no. 7 / NBRC 101557) OX=721032 GN=K7_TDH2 PE=3 SV=1 | 1367.6 | 35961.0 | 99 | 11 | 31 | 38.55 | Green |
| C8ZBG4_YEAS8 | C8ZBG4 | Glyceraldehyde-3-phosphate dehydrogenase OS=Saccharomyces cerevisiae (strain Lalvin EC1118 / Prise de mousse) OX=643680 GN=EC1118_1J11_2685g PE=3 SV=1 | 1367.6 | 35961.0 | 99 | 11 | 31 | 38.55 | Green |
| B3LQA8_YEAS1 | B3LQA8 | Glyceraldehyde-3-phosphate dehydrogenase OS=Saccharomyces cerevisiae (strain RM11-1a) OX=285006 GN=SCRG_03670 PE=3 SV=1 | 1367.6 | 35961.0 | 99 | 11 | 31 | 38.55 | Green |
| A6ZPX6_YEAS7 | A6ZPX6 | Glyceraldehyde-3-phosphate dehydrogenase OS=Saccharomyces cerevisiae (strain YJM789) OX=307796 GN=TDH2 PE=3 SV=1 | 1367.6 | 35961.0 | 99 | 11 | 31 | 38.55 | Green |
| A0A6A5PXB1_YEASX | A0A6A5PXB1 | Glyceraldehyde-3-phosphate dehydrogenase OS=Saccharomyces cerevisiae OX=4932 GN=TDH2 PE=3 SV=1 | 1367.6 | 35961.0 | 99 | 11 | 31 | 38.55 | Green |
| G3P2_YEAST | P00358 | Glyceraldehyde-3-phosphate dehydrogenase 2 OS=Saccharomyces cerevisiae (strain ATCC 204508 / S288c) OX=559292 GN=TDH2 PE=1 SV=3 | 1367.6 | 35961.0 | 99 | 11 | 31 | 38.55 | Green |
| B5VM12_YEAS6 | B5VM12 | Phosphoglycerate mutase (Fragment) OS=Saccharomyces cerevisiae (strain AWRI1631) OX=545124 GN=AWRI1631_110760 PE=3 SV=1 | 551.2 | 28743.9 | 46 | 5 | 22 | 37.74 | Green |
| B3LUR9_YEAS1 | B3LUR9 | Acid phosphatase OS=Saccharomyces cerevisiae (strain RM11-1a) OX=285006 GN=SCRG_05608 PE=4 SV=1 | 280.2 | 8467.7 | 9 | 1 | 6 | 35.9 | Green |
| N1P635_YEASC | N1P635 | Scw10p OS=Saccharomyces cerevisiae (strain CEN.PK113-7D) OX=889517 GN=CENPK1137D_350 PE=3 SV=1 | 839.1 | 40754.3 | 70 | 9 | 17 | 34.7 | Green |
| H0GLJ6_SACCK | H0GLJ6 | Scw10p OS=Saccharomyces cerevisiae x Saccharomyces kudriavzevii (strain VIN7) OX=1095631 GN=VIN7_3939 PE=3 SV=1 | 839.1 | 40754.3 | 70 | 9 | 17 | 34.7 | Green |
| C8ZFH4_YEAS8 | C8ZFH4 | Scw10p OS=Saccharomyces cerevisiae (strain Lalvin EC1118 / Prise de mousse) OX=643680 GN=EC1118_1M3_5215g PE=3 SV=1 | 839.1 | 40724.3 | 70 | 9 | 17 | 34.7 | Green |
| B3LMJ3_YEAS1 | B3LMJ3 | Soluble cell wall protein OS=Saccharomyces cerevisiae (strain RM11-1a) OX=285006 GN=SCRG_02202 PE=3 SV=1 | 839.1 | 40754.3 | 70 | 9 | 17 | 34.7 | Green |
| A0A6A5PQ89_YEASX | A0A6A5PQ89 | SCW10 isoform 1 OS=Saccharomyces cerevisiae OX=4932 GN=SCW10 PE=3 SV=1 | 839.1 | 40754.3 | 70 | 9 | 17 | 34.7 | Green |
| G2WL14_YEASK | G2WL14 | K7_Scw10p OS=Saccharomyces cerevisiae (strain Kyokai no. 7 / NBRC 101557) OX=721032 GN=K7_SCW10 PE=3 SV=1 | 839.1 | 40784.4 | 70 | 9 | 17 | 34.7 | Green |
| C7GPV8_YEAS2 | C7GPV8 | Scw10p OS=Saccharomyces cerevisiae (strain JAY291) OX=574961 GN=SCW10 PE=3 SV=1 | 839.1 | 40754.3 | 70 | 9 | 17 | 34.7 | Green |
| A6ZN21_YEAS7 | A6ZN21 | Soluble cell wall protein OS=Saccharomyces cerevisiae (strain YJM789) OX=307796 GN=SCW10 PE=3 SV=1 | 839.1 | 40754.3 | 70 | 9 | 17 | 34.7 | Green |
| SCW10_YEAST | Q04951 | Probable family 17 glucosidase SCW10 OS=Saccharomyces cerevisiae (strain ATCC 204508 / S288c) OX=559292 GN=SCW10 PE=1 SV=1 | 839.1 | 40754.3 | 70 | 9 | 17 | 34.7 | Green |
| H0H2T9_SACCK | H0H2T9 | Pho12p OS=Saccharomyces cerevisiae x Saccharomyces kudriavzevii (strain VIN7) OX=1095631 GN=VIN7_8176 PE=3 SV=1 | 447.5 | 26144.2 | 28 | 3 | 17 | 34.62 | Green |
| A0A1X7R1L9_9SACH | A0A1X7R1L9 | Similar to Saccharomyces cerevisiae YBR173C UMP1 Short-lived chaperone required for correct maturation of the 20S proteasome OS=Kazachstania saulgeensis OX=1789683 GN=KASA_0O01320G PE=4 SV=1 | 248.2 | 16846.7 | 17 | 3 | 19 | 34.23 | Green |
| H0GK16_SACCK | H0GK16 | branched-chain-2-oxoacid decarboxylase OS=Saccharomyces cerevisiae x Saccharomyces kudriavzevii (strain VIN7) OX=1095631 GN=VIN7_3196 PE=3 SV=1 | 381.5 | 61723.6 | 113 | 16 | 35 | 33.93 | Green |
| B3LT15_YEAS1 | B3LT15 | branched-chain-2-oxoacid decarboxylase OS=Saccharomyces cerevisiae (strain RM11-1a) OX=285006 GN=SCRG_05030 PE=3 SV=1 | 381.5 | 61723.6 | 113 | 16 | 35 | 33.93 | Green |
| G2WIN6_YEASK | G2WIN6 | branched-chain-2-oxoacid decarboxylase OS=Saccharomyces cerevisiae (strain Kyokai no. 7 / NBRC 101557) OX=721032 GN=K7_PDC1 PE=3 SV=1 | 381.5 | 61737.6 | 113 | 16 | 35 | 33.93 | Green |
| A7A0U9_YEAS7 | A7A0U9 | branched-chain-2-oxoacid decarboxylase OS=Saccharomyces cerevisiae (strain YJM789) OX=307796 GN=PDC1 PE=3 SV=1 | 381.5 | 61723.6 | 113 | 16 | 35 | 33.93 | Green |
| A0A7I9FNH9_YEASX | A0A7I9FNH9 | branched-chain-2-oxoacid decarboxylase OS=Saccharomyces cerevisiae OX=4932 GN=PDC1 PE=3 SV=1 | 381.5 | 61723.6 | 113 | 16 | 35 | 33.93 | Green |
| A0A6C1DVV6_SACPS | A0A6C1DVV6 | branched-chain-2-oxoacid decarboxylase OS=Saccharomyces pastorianus OX=27292 GN=PDC1_1 PE=3 SV=1 | 381.5 | 61723.6 | 113 | 16 | 35 | 33.93 | Green |
| PDC1_YEAST | P06169 | Pyruvate decarboxylase isozyme 1 OS=Saccharomyces cerevisiae (strain ATCC 204508 / S288c) OX=559292 GN=PDC1 PE=1 SV=7 | 381.5 | 61723.6 | 113 | 16 | 35 | 33.93 | Green |
| A0A0B4VK19_YEASX | A0A0B4VK19 | Glucose-repressible alcohol dehydrogenase II (Fragment) OS=Saccharomyces cerevisiae OX=4932 GN=ADH2 PE=4 SV=1 | 239.6 | 10014.5 | 13 | 2 | 9 | 32.97 | Green |
| A0A0B4VK15_YEASX | A0A0B4VK15 | Glucose-repressible alcohol dehydrogenase II (Fragment) OS=Saccharomyces cerevisiae OX=4932 GN=ADH2 PE=4 SV=1 | 239.6 | 9980.5 | 13 | 2 | 9 | 32.97 | Green |
| A0A0B4VIW6_YEASX | A0A0B4VIW6 | Glucose-repressible alcohol dehydrogenase II (Fragment) OS=Saccharomyces cerevisiae OX=4932 GN=ADH2 PE=4 SV=1 | 239.6 | 9954.5 | 13 | 2 | 9 | 32.97 | Green |
| A0A0B4VIM8_YEASX | A0A0B4VIM8 | Glucose-repressible alcohol dehydrogenase II (Fragment) OS=Saccharomyces cerevisiae OX=4932 GN=ADH2 PE=4 SV=1 | 239.6 | 9981.5 | 13 | 2 | 9 | 32.97 | Green |
| A7A095_YEAS7 | A7A095 | acid phosphatase (Fragment) OS=Saccharomyces cerevisiae (strain YJM789) OX=307796 GN=PHO11 PE=3 SV=1 | 575.4 | 52330.0 | 33 | 6 | 35 | 32.9 | Green |
| A7A091_YEAS7 | A7A091 | Acid phosphatase (Fragment) OS=Saccharomyces cerevisiae (strain YJM789) OX=307796 GN=SCY_0002 PE=3 SV=1 | 536.0 | 38862.2 | 44 | 5 | 28 | 32.85 | Green |
| H0GQP2_SACCK | H0GQP2 | acid phosphatase OS=Saccharomyces cerevisiae x Saccharomyces kudriavzevii (strain VIN7) OX=1095631 GN=VIN7_5330 PE=3 SV=1 | 575.4 | 53271.1 | 33 | 6 | 35 | 32.33 | Green |
| B5VKE4_YEAS6 | B5VKE4 | acid phosphatase OS=Saccharomyces cerevisiae (strain AWRI1631) OX=545124 GN=AWRI1631_82680 PE=3 SV=1 | 575.4 | 53271.1 | 33 | 6 | 35 | 32.33 | Green |
| A6ZSK2_YEAS7 | A6ZSK2 | acid phosphatase OS=Saccharomyces cerevisiae (strain YJM789) OX=307796 GN=PHO12 PE=3 SV=1 | 575.4 | 53271.1 | 33 | 6 | 36 | 32.33 | Green |
| A0A8H4C1F2_YEASX | A0A8H4C1F2 | acid phosphatase OS=Saccharomyces cerevisiae OX=4932 GN=PHO11 PE=3 SV=1 | 575.4 | 53271.1 | 33 | 6 | 36 | 32.33 | Green |
| A0A6C1DRP2_SACPS | A0A6C1DRP2 | acid phosphatase OS=Saccharomyces pastorianus OX=27292 GN=PHO11_1 PE=3 SV=1 | 575.4 | 53271.1 | 33 | 6 | 36 | 32.33 | Green |
| PPAB_YEAST | P35842 | Acid phosphatase PHO11 OS=Saccharomyces cerevisiae (strain ATCC 204508 / S288c) OX=559292 GN=PHO11 PE=1 SV=1 | 575.4 | 53271.1 | 33 | 6 | 36 | 32.33 | Green |
| A0A0B4VIU7_YEASX | A0A0B4VIU7 | Glucose-repressible alcohol dehydrogenase II (Fragment) OS=Saccharomyces cerevisiae OX=4932 GN=ADH2 PE=4 SV=1 | 239.6 | 10294.9 | 13 | 2 | 9 | 32.26 | Green |
| C8ZD16_YEAS8 | C8ZD16 | branched-chain-2-oxoacid decarboxylase OS=Saccharomyces cerevisiae (strain Lalvin EC1118 / Prise de mousse) OX=643680 GN=EC1118_1L10_1178g PE=3 SV=1 | 372.4 | 61757.6 | 113 | 15 | 36 | 31.97 | Green |
| B3LSX7_YEAS1 | B3LSX7 | Acid phosphatase OS=Saccharomyces cerevisiae (strain RM11-1a) OX=285006 GN=SCRG_04930 PE=3 SV=1 | 447.5 | 28424.7 | 28 | 3 | 19 | 31.89 | Green |
| Q756U7_EREGS | Q756U7 | AER155Cp OS=Eremothecium gossypii (strain ATCC 10895 / CBS 109.51 / FGSC 9923 / NRRL Y-1056) OX=284811 GN=AGOS_AER155C PE=4 SV=1 | 187.4 | 40102.8 | 39 | 6 | 32 | 31.87 | Green |
| S4VPL7_YEASX | S4VPL7 | Superoxide dismutase OS=Saccharomyces cerevisiae OX=4932 GN=sod2 PE=3 SV=1 | 680.4 | 25831.3 | 54 | 7 | 19 | 31.76 | Green |
| S4VIJ3_YEASX | S4VIJ3 | Superoxide dismutase OS=Saccharomyces cerevisiae var. ellipsoideus OX=1348153 GN=sod2 PE=3 SV=1 | 680.4 | 25831.3 | 54 | 7 | 19 | 31.76 | Green |
| N1P9K8_YEASC | N1P9K8 | Superoxide dismutase OS=Saccharomyces cerevisiae (strain CEN.PK113-7D) OX=889517 GN=CENPK1137D_5400 PE=3 SV=1 | 680.4 | 25831.3 | 54 | 7 | 19 | 31.76 | Green |
| H0GH32_SACCK | H0GH32 | Superoxide dismutase OS=Saccharomyces cerevisiae x Saccharomyces kudriavzevii (strain VIN7) OX=1095631 GN=VIN7_2106 PE=3 SV=1 | 680.4 | 25831.3 | 54 | 7 | 19 | 31.76 | Green |
| G2WF76_YEASK | G2WF76 | Superoxide dismutase OS=Saccharomyces cerevisiae (strain Kyokai no. 7 / NBRC 101557) OX=721032 GN=K7_SOD2 PE=3 SV=1 | 680.4 | 25831.3 | 54 | 7 | 19 | 31.76 | Green |
| C8ZA75_YEAS8 | C8ZA75 | Superoxide dismutase OS=Saccharomyces cerevisiae (strain Lalvin EC1118 / Prise de mousse) OX=643680 GN=EC1118_1H21_0716g PE=3 SV=1 | 680.4 | 25831.3 | 54 | 7 | 19 | 31.76 | Green |
| C7GMQ3_YEAS2 | C7GMQ3 | Superoxide dismutase OS=Saccharomyces cerevisiae (strain JAY291) OX=574961 GN=SOD2 PE=3 SV=1 | 680.4 | 25831.3 | 54 | 7 | 19 | 31.76 | Green |
| B5VJT4_YEAS6 | B5VJT4 | Superoxide dismutase OS=Saccharomyces cerevisiae (strain AWRI1631) OX=545124 GN=AWRI1631_80530 PE=3 SV=1 | 680.4 | 25831.3 | 54 | 7 | 19 | 31.76 | Green |
| B3LSC9_YEAS1 | B3LSC9 | Superoxide dismutase OS=Saccharomyces cerevisiae (strain RM11-1a) OX=285006 GN=SCRG_04714 PE=3 SV=1 | 680.4 | 25831.3 | 54 | 7 | 19 | 31.76 | Green |
| A6ZSR2_YEAS7 | A6ZSR2 | Superoxide dismutase OS=Saccharomyces cerevisiae (strain YJM789) OX=307796 GN=SOD2 PE=3 SV=1 | 680.4 | 25831.3 | 54 | 7 | 19 | 31.76 | Green |
| A0A6C1DS42_SACPS | A0A6C1DS42 | Superoxide dismutase OS=Saccharomyces pastorianus OX=27292 GN=SOD2_1 PE=3 SV=1 | 680.4 | 25831.3 | 54 | 7 | 19 | 31.76 | Green |
| SODM_YEAST | P00447 | Superoxide dismutase [Mn]_ mitochondrial OS=Saccharomyces cerevisiae (strain ATCC 204508 / S288c) OX=559292 GN=SOD2 PE=1 SV=1 | 680.4 | 25831.3 | 54 | 7 | 19 | 31.76 | Green |
| A0A6C1DY36_SACPS | A0A6C1DY36 | Putative 17 glucosidase scw10 OS=Saccharomyces pastorianus OX=27292 GN=SCW10_1 PE=3 SV=1 | 303.9 | 38194.7 | 44 | 8 | 16 | 31.59 | Green |
| A0A0B4VJK5_YEASX | A0A0B4VJK5 | Glucose-repressible alcohol dehydrogenase II (Fragment) OS=Saccharomyces cerevisiae OX=4932 GN=ADH2 PE=4 SV=1 | 239.6 | 10522.2 | 13 | 2 | 9 | 31.58 | Green |
| B5VJM2_YEAS6 | B5VJM2 | YGR279Cp-like protein (Fragment) OS=Saccharomyces cerevisiae (strain AWRI1631) OX=545124 GN=AWRI1631_75030 PE=4 SV=1 | 1717.9 | 28319.8 | 58 | 4 | 9 | 29.6 | Green |
| B7X711_SACPS | B7X711 | Glyceraldehyde-3-phosphate dehydrogenase OS=Saccharomyces pastorianus OX=27292 GN=YJR009C PE=3 SV=1 | 1186.5 | 31088.3 | 59 | 7 | 28 | 29.41 | Green |
| A0A6C1EB88_SACPS | A0A6C1EB88 | Glyceraldehyde-3-phosphate dehydrogenase OS=Saccharomyces pastorianus OX=27292 GN=TDH2_3 PE=3 SV=1 | 1183.8 | 35993.0 | 57 | 7 | 31 | 29.22 | Green |
| N1NY02_YEASC | N1NY02 | Tsa1p OS=Saccharomyces cerevisiae (strain CEN.PK113-7D) OX=889517 GN=CENPK1137D_424 PE=3 SV=1 | 233.1 | 21703.8 | 30 | 3 | 21 | 29.08 | Green |
| G2WK24_YEASK | G2WK24 | K7_Tsa1p OS=Saccharomyces cerevisiae (strain Kyokai no. 7 / NBRC 101557) OX=721032 GN=K7_TSA1 PE=3 SV=1 | 233.1 | 21703.8 | 30 | 3 | 21 | 29.08 | Green |
| C8ZEH6_YEAS8 | C8ZEH6 | Tsa1p OS=Saccharomyces cerevisiae (strain Lalvin EC1118 / Prise de mousse) OX=643680 GN=EC1118_1M3_1222g PE=3 SV=1 | 233.1 | 21703.8 | 30 | 3 | 21 | 29.08 | Green |
| C7GT58_YEAS2 | C7GT58 | Tsa1p OS=Saccharomyces cerevisiae (strain JAY291) OX=574961 GN=TSA1 PE=3 SV=1 | 233.1 | 21788.9 | 30 | 3 | 20 | 29.08 | Green |
| B3LLM4_YEAS1 | B3LLM4 | Peroxiredoxin TSA1 OS=Saccharomyces cerevisiae (strain RM11-1a) OX=285006 GN=SCRG_01867 PE=3 SV=1 | 233.1 | 21703.8 | 30 | 3 | 21 | 29.08 | Green |
| A6ZM34_YEAS7 | A6ZM34 | CTPxI OS=Saccharomyces cerevisiae (strain YJM789) OX=307796 GN=TSA1 PE=3 SV=1 | 233.1 | 21703.8 | 30 | 3 | 21 | 29.08 | Green |
| A0A6C1DXZ1_SACPS | A0A6C1DXZ1 | CTPxI OS=Saccharomyces pastorianus OX=27292 GN=TSA1_1 PE=3 SV=1 | 233.1 | 21703.8 | 30 | 3 | 21 | 29.08 | Green |
| A0A6A5PQ45_YEASX | A0A6A5PQ45 | TSA1 isoform 1 OS=Saccharomyces cerevisiae OX=4932 GN=TSA1 PE=3 SV=1 | 233.1 | 21703.8 | 30 | 3 | 21 | 29.08 | Green |
| TSA1_YEAST | P34760 | Peroxiredoxin TSA1 OS=Saccharomyces cerevisiae (strain ATCC 204508 / S288c) OX=559292 GN=TSA1 PE=1 SV=3 | 233.1 | 21703.8 | 30 | 3 | 21 | 29.08 | Green |
| N1P2L2_YEASC | N1P2L2 | acid phosphatase OS=Saccharomyces cerevisiae (strain CEN.PK113-7D) OX=889517 GN=CENPK1137D_5368 PE=3 SV=1 | 702.2 | 53214.0 | 44 | 6 | 35 | 28.91 | Green |
| H0GQM4_SACCK | H0GQM4 | acid phosphatase OS=Saccharomyces cerevisiae x Saccharomyces kudriavzevii (strain VIN7) OX=1095631 GN=VIN7_5311 PE=3 SV=1 | 702.2 | 53266.1 | 44 | 6 | 35 | 28.91 | Green |
| C8Z7M5_YEAS8 | C8Z7M5 | acid phosphatase OS=Saccharomyces cerevisiae (strain Lalvin EC1118 / Prise de mousse) OX=643680 GN=EC1118_1F14_0188g PE=3 SV=1 | 702.2 | 53155.0 | 44 | 6 | 35 | 28.91 | Green |
| C7GJR1_YEAS2 | C7GJR1 | acid phosphatase OS=Saccharomyces cerevisiae (strain JAY291) OX=574961 GN=PHO11 PE=3 SV=1 | 702.2 | 53213.0 | 44 | 6 | 35 | 28.91 | Green |
| B3LUF9_YEAS1 | B3LUF9 | acid phosphatase OS=Saccharomyces cerevisiae (strain RM11-1a) OX=285006 GN=SCRG_05494 PE=3 SV=1 | 702.2 | 53213.0 | 44 | 6 | 35 | 28.91 | Green |
| A0A8H4C1M3_YEASX | A0A8H4C1M3 | acid phosphatase OS=Saccharomyces cerevisiae OX=4932 GN=PHO12 PE=3 SV=1 | 702.2 | 53213.0 | 44 | 6 | 35 | 28.91 | Green |
| PPAC_YEAST | P38693 | Acid phosphatase PHO12 OS=Saccharomyces cerevisiae (strain ATCC 204508 / S288c) OX=559292 GN=PHO12 PE=1 SV=1 | 702.2 | 53213.0 | 44 | 6 | 35 | 28.91 | Green |
| A0A5Q2WV56_YEASX | A0A5Q2WV56 | branched-chain-2-oxoacid decarboxylase OS=Saccharomyces cerevisiae OX=4932 GN=pdc PE=3 SV=1 | 278.4 | 61722.5 | 82 | 11 | 36 | 28.06 | Green |
| A0A6C1E8Y3_SACPS | A0A6C1E8Y3 | Superoxide dismutase OS=Saccharomyces pastorianus OX=27292 GN=SOD2_2 PE=3 SV=1 | 580.1 | 25969.5 | 46 | 6 | 19 | 27.9 | Green |
| N1P0G2_YEASC | N1P0G2 | branched-chain-2-oxoacid decarboxylase OS=Saccharomyces cerevisiae (strain CEN.PK113-7D) OX=889517 GN=CENPK1137D_891 PE=3 SV=1 | 335.0 | 61738.6 | 77 | 13 | 35 | 27.71 | Green |
| H0H2T8_SACCK | H0H2T8 | Pho11p OS=Saccharomyces cerevisiae x Saccharomyces kudriavzevii (strain VIN7) OX=1095631 GN=VIN7_8175 PE=4 SV=1 | 189.3 | 13717.1 | 11 | 2 | 8 | 27.59 | Green |
| B7X717_SACPS | B7X717 | Glyceraldehyde-3-phosphate dehydrogenase OS=Saccharomyces pastorianus OX=27292 GN=YJR009C PE=3 SV=1 | 1163.6 | 31134.3 | 53 | 6 | 28 | 25.95 | Green |
| B7X714_SACPS | B7X714 | Glyceraldehyde-3-phosphate dehydrogenase OS=Saccharomyces pastorianus OX=27292 GN=YJR009C PE=3 SV=1 | 1163.6 | 31143.3 | 53 | 6 | 28 | 25.95 | Green |
| H0GVM2_SACCK | H0GVM2 | Superoxide dismutase OS=Saccharomyces cerevisiae x Saccharomyces kudriavzevii (strain VIN7) OX=1095631 GN=VIN7_7477 PE=3 SV=1 | 579.1 | 25894.3 | 46 | 6 | 19 | 25.75 | Green |
| H0GWY1_SACCK | H0GWY1 | Glyceraldehyde-3-phosphate dehydrogenase OS=Saccharomyces cerevisiae x Saccharomyces kudriavzevii (strain VIN7) OX=1095631 GN=VIN7_8041 PE=3 SV=1 | 1186.5 | 35898.0 | 59 | 7 | 30 | 25.6 | Green |
| H0GVA3_SACCK | H0GVA3 | Glyceraldehyde-3-phosphate dehydrogenase OS=Saccharomyces cerevisiae x Saccharomyces kudriavzevii (strain VIN7) OX=1095631 GN=VIN7_7332 PE=3 SV=1 | 1186.5 | 35907.9 | 59 | 7 | 31 | 25.6 | Green |
| B7X708_SACPS | B7X708 | Glyceraldehyde-3-phosphate dehydrogenase OS=Saccharomyces pastorianus OX=27292 GN=YJR009C PE=3 SV=1 | 1186.5 | 35936.0 | 59 | 7 | 31 | 25.6 | Green |
| B5VJM6_YEAS6 | B5VJM6 | YGR282Cp-like protein (Fragment) OS=Saccharomyces cerevisiae (strain AWRI1631) OX=545124 GN=AWRI1631_75070 PE=3 SV=1 | 186.2 | 28359.3 | 23 | 3 | 11 | 24.71 | Green |
| H0H0S1_SACCK | H0H0S1 | Adh1p OS=Saccharomyces cerevisiae x Saccharomyces kudriavzevii (strain VIN7) OX=1095631 GN=VIN7_9785 PE=3 SV=1 | 245.1 | 37264.4 | 47 | 9 | 24 | 24.43 | Green |
| H0GXZ9_SACCK | H0GXZ9 | branched-chain-2-oxoacid decarboxylase OS=Saccharomyces cerevisiae x Saccharomyces kudriavzevii (strain VIN7) OX=1095631 GN=VIN7_8597 PE=3 SV=1 | 294.4 | 61612.4 | 96 | 14 | 34 | 24.33 | Green |
| H0GQL5_SACCK | H0GQL5 | acid phosphatase OS=Saccharomyces cerevisiae x Saccharomyces kudriavzevii (strain VIN7) OX=1095631 GN=VIN7_5302 PE=3 SV=1 | 536.0 | 53102.9 | 44 | 5 | 34 | 24.2 | Green |
| A0A0B4VIW5_YEASX | A0A0B4VIW5 | Glucose-repressible alcohol dehydrogenase II (Fragment) OS=Saccharomyces cerevisiae OX=4932 GN=ADH2 PE=4 SV=1 | 208.0 | 9523.0 | 10 | 1 | 6 | 24.14 | Green |
| N1P832_YEASC | N1P832 | Phosphoglycerate kinase OS=Saccharomyces cerevisiae (strain CEN.PK113-7D) OX=889517 GN=CENPK1137D_4509 PE=3 SV=1 | 459.5 | 44795.5 | 47 | 7 | 44 | 23.8 | Green |
| H0GD00_SACCK | H0GD00 | Phosphoglycerate kinase OS=Saccharomyces cerevisiae x Saccharomyces kudriavzevii (strain VIN7) OX=1095631 GN=VIN7_0465 PE=3 SV=1 | 459.5 | 44809.5 | 47 | 7 | 44 | 23.8 | Green |
| G2WA29_YEASK | G2WA29 | Phosphoglycerate kinase OS=Saccharomyces cerevisiae (strain Kyokai no. 7 / NBRC 101557) OX=721032 GN=K7_PGK1 PE=3 SV=1 | 459.5 | 44795.5 | 47 | 7 | 44 | 23.8 | Green |
| C8Z499_YEAS8 | C8Z499 | Phosphoglycerate kinase OS=Saccharomyces cerevisiae (strain Lalvin EC1118 / Prise de mousse) OX=643680 GN=EC1118_1C17_0859g PE=3 SV=2 | 459.5 | 44795.5 | 47 | 7 | 44 | 23.8 | Green |
| C7GQM4_YEAS2 | C7GQM4 | Phosphoglycerate kinase OS=Saccharomyces cerevisiae (strain JAY291) OX=574961 GN=PGK1 PE=3 SV=1 | 459.5 | 44795.5 | 47 | 7 | 44 | 23.8 | Green |
| B3LU83_YEAS1 | B3LU83 | Phosphoglycerate kinase OS=Saccharomyces cerevisiae (strain RM11-1a) OX=285006 GN=SCRG_05411 PE=3 SV=1 | 459.5 | 44795.5 | 47 | 7 | 44 | 23.8 | Green |
| A6ZTJ3_YEAS7 | A6ZTJ3 | Phosphoglycerate kinase OS=Saccharomyces cerevisiae (strain YJM789) OX=307796 GN=PGK1 PE=3 SV=1 | 459.5 | 44795.5 | 47 | 7 | 44 | 23.8 | Green |
| A0A6A5Q0F0_YEASX | A0A6A5Q0F0 | Phosphoglycerate kinase OS=Saccharomyces cerevisiae OX=4932 GN=PGK1 PE=3 SV=1 | 459.5 | 44795.5 | 47 | 7 | 44 | 23.8 | Green |
| PGK_YEAST | P00560 | Phosphoglycerate kinase OS=Saccharomyces cerevisiae (strain ATCC 204508 / S288c) OX=559292 GN=PGK1 PE=1 SV=2 | 459.5 | 44795.5 | 47 | 7 | 44 | 23.8 | Green |
| A6ZSK5_YEAS7 | A6ZSK5 | acid phosphatase OS=Saccharomyces cerevisiae (strain YJM789) OX=307796 GN=PHO12 PE=3 SV=1 | 617.8 | 53197.0 | 36 | 5 | 35 | 23.55 | Green |
| B5VLC4_YEAS6 | B5VLC4 | glyceraldehyde-3-phosphate dehydrogenase (phosphorylating) (Fragment) OS=Saccharomyces cerevisiae (strain AWRI1631) OX=545124 GN=AWRI1631_101500 PE=3 SV=1 | 841.3 | 27906.9 | 38 | 5 | 25 | 23.28 | Green |
| A0A0B4VJK7_YEASX | A0A0B4VJK7 | Glucose-repressible alcohol dehydrogenase II (Fragment) OS=Saccharomyces cerevisiae OX=4932 GN=ADH2 PE=4 SV=1 | 208.0 | 9982.5 | 10 | 1 | 8 | 23.08 | Green |
| B5VEW0_YEAS6 | B5VEW0 | Phosphoglycerate kinase OS=Saccharomyces cerevisiae (strain AWRI1631) OX=545124 GN=AWRI1631_30700 PE=3 SV=1 | 371.9 | 15363.6 | 18 | 2 | 15 | 22.82 | Green |
| A0A6C1E7B5_SACPS | A0A6C1E7B5 | Glyceraldehyde-3-phosphate dehydrogenase OS=Saccharomyces pastorianus OX=27292 GN=TDH2_2 PE=3 SV=1 | 1163.6 | 35982.1 | 53 | 6 | 31 | 22.59 | Green |
| C7GU46_YEAS2 | C7GU46 | branched-chain-2-oxoacid decarboxylase OS=Saccharomyces cerevisiae (strain JAY291) OX=574961 GN=PDC1 PE=3 SV=1 | 220.1 | 46938.8 | 78 | 11 | 29 | 22.07 | Green |
| G3P_EREGS | Q757I2 | Glyceraldehyde-3-phosphate dehydrogenase OS=Eremothecium gossypii (strain ATCC 10895 / CBS 109.51 / FGSC 9923 / NRRL Y-1056) OX=284811 GN=GPD PE=3 SV=2 | 1247.5 | 35633.6 | 79 | 7 | 27 | 21.45 | Green |
| A0A6C1EF25_SACPS | A0A6C1EF25 | Putative 17 glucosidase scw10 OS=Saccharomyces pastorianus OX=27292 GN=SCW10_2 PE=4 SV=1 | 700.9 | 47511.4 | 49 | 5 | 21 | 20.95 | Green |
| A0A6C1DVM7_SACPS | A0A6C1DVM7 | Aspartyl protease OS=Saccharomyces pastorianus OX=27292 GN=YPS3_1 PE=3 SV=1 | 191.0 | 53738.2 | 38 | 6 | 35 | 20.84 | Green |
| N1P4I5_YEASC | N1P4I5 | Bgl2p OS=Saccharomyces cerevisiae (strain CEN.PK113-7D) OX=889517 GN=CENPK1137D_3249 PE=3 SV=1 | 186.2 | 34346.8 | 23 | 3 | 16 | 20.77 | Green |
| H0GH08_SACCK | H0GH08 | Bgl2p OS=Saccharomyces cerevisiae x Saccharomyces kudriavzevii (strain VIN7) OX=1095631 GN=VIN7_2048 PE=3 SV=1 | 186.2 | 34346.8 | 23 | 3 | 16 | 20.77 | Green |
| G2WF05_YEASK | G2WF05 | K7_Bgl2p OS=Saccharomyces cerevisiae (strain Kyokai no. 7 / NBRC 101557) OX=721032 GN=K7_BGL2 PE=3 SV=1 | 186.2 | 34346.8 | 23 | 3 | 16 | 20.77 | Green |
| F8KAD8_YEASX | F8KAD8 | Endo-beta-1_3-glucanase OS=Saccharomyces cerevisiae OX=4932 GN=BGL2 PE=3 SV=1 | 186.2 | 34360.8 | 23 | 3 | 16 | 20.77 | Green |
| C8Z9H5_YEAS8 | C8Z9H5 | Bgl2p OS=Saccharomyces cerevisiae (strain Lalvin EC1118 / Prise de mousse) OX=643680 GN=EC1118_1G1_6227g PE=3 SV=1 | 186.2 | 34346.8 | 23 | 3 | 16 | 20.77 | Green |
| C7GNF3_YEAS2 | C7GNF3 | Bgl2p OS=Saccharomyces cerevisiae (strain JAY291) OX=574961 GN=BGL2 PE=3 SV=1 | 186.2 | 34346.8 | 23 | 3 | 16 | 20.77 | Green |
| B3LHW3_YEAS1 | B3LHW3 | Cell wall endo-beta-1_3-glucanase OS=Saccharomyces cerevisiae (strain RM11-1a) OX=285006 GN=SCRG_00745 PE=3 SV=1 | 186.2 | 34346.8 | 23 | 3 | 16 | 20.77 | Green |
| A6ZUT8_YEAS7 | A6ZUT8 | Cell wall endo-beta-1_3-glucanase OS=Saccharomyces cerevisiae (strain YJM789) OX=307796 GN=BGL2 PE=3 SV=1 | 186.2 | 34346.8 | 23 | 3 | 16 | 20.77 | Green |
| A0A6A5PVP7_YEASX | A0A6A5PVP7 | BGL2 isoform 1 OS=Saccharomyces cerevisiae OX=4932 GN=BGL2 PE=3 SV=1 | 186.2 | 34346.8 | 23 | 3 | 16 | 20.77 | Green |
| F8KAD4_SACPS | F8KAD4 | Endo-beta-1_3-glucanase OS=Saccharomyces pastorianus OX=27292 GN=bgl2 PE=3 SV=1 | 186.2 | 34346.8 | 23 | 3 | 16 | 20.77 | Green |
| BGL2_YEAST | P15703 | Glucan 1_3-beta-glucosidase OS=Saccharomyces cerevisiae (strain ATCC 204508 / S288c) OX=559292 GN=BGL2 PE=1 SV=1 | 186.2 | 34346.8 | 23 | 3 | 16 | 20.77 | Green |
| A0A6A5PSL3_YEASX | A0A6A5PSL3 | YPS3 isoform 1 OS=Saccharomyces cerevisiae OX=4932 GN=YPS3 PE=3 SV=1 | 191.0 | 54798.6 | 38 | 6 | 33 | 20.47 | Green |
| N1NY91_YEASC | N1NY91 | Yps3p OS=Saccharomyces cerevisiae (strain CEN.PK113-7D) OX=889517 GN=CENPK1137D_509 PE=3 SV=1 | 191.0 | 54798.6 | 38 | 6 | 33 | 20.47 | Green |
| G2WIW3_YEASK | G2WIW3 | K7_Yps3p OS=Saccharomyces cerevisiae (strain Kyokai no. 7 / NBRC 101557) OX=721032 GN=K7_YPS3 PE=3 SV=1 | 191.0 | 54798.6 | 38 | 6 | 33 | 20.47 | Green |
| C7GQD1_YEAS2 | C7GQD1 | Yps3p OS=Saccharomyces cerevisiae (strain JAY291) OX=574961 GN=YPS3 PE=3 SV=1 | 191.0 | 54757.5 | 38 | 6 | 33 | 20.47 | Green |
| A7A123_YEAS7 | A7A123 | Aspartic protease OS=Saccharomyces cerevisiae (strain YJM789) OX=307796 GN=YPS3 PE=3 SV=1 | 191.0 | 54798.6 | 38 | 6 | 33 | 20.47 | Green |
| YPS3_YEAST | Q12303 | Aspartic proteinase yapsin-3 OS=Saccharomyces cerevisiae (strain ATCC 204508 / S288c) OX=559292 GN=YPS3 PE=1 SV=1 | 191.0 | 54798.6 | 38 | 6 | 33 | 20.47 | Green |
| H0GUF7_SACCK | H0GUF7 | Phosphotransferase OS=Saccharomyces cerevisiae x Saccharomyces kudriavzevii (strain VIN7) OX=1095631 GN=VIN7_6946 PE=3 SV=1 | 204.1 | 54170.6 | 40 | 8 | 41 | 19.96 | Green |
| A0A6C1EBJ7_SACPS | A0A6C1EBJ7 | branched-chain-2-oxoacid decarboxylase OS=Saccharomyces pastorianus OX=27292 GN=PDC1_2 PE=3 SV=1 | 197.6 | 61720.6 | 74 | 10 | 34 | 19.54 | Green |
| H0GRW0_SACCK | H0GRW0 | Phosphoglycerate kinase OS=Saccharomyces cerevisiae x Saccharomyces kudriavzevii (strain VIN7) OX=1095631 GN=VIN7_5856 PE=3 SV=1 | 439.3 | 44756.5 | 41 | 6 | 43 | 19.47 | Green |
| A0A6C1E663_SACPS | A0A6C1E663 | acid phosphatase OS=Saccharomyces pastorianus OX=27292 GN=PHO11_2 PE=3 SV=1 | 340.6 | 51901.7 | 22 | 4 | 31 | 18.9 | Green |
| H0GQM1_SACCK | H0GQM1 | acid phosphatase OS=Saccharomyces cerevisiae x Saccharomyces kudriavzevii (strain VIN7) OX=1095631 GN=VIN7_5308 PE=3 SV=1 | 255.9 | 52023.5 | 35 | 4 | 34 | 18.81 | Green |
| F8KAB8_SACBA | F8KAB8 | Alcohol dehydrogenase (Fragment) OS=Saccharomyces bayanus OX=4931 GN=adh1 PE=3 SV=1 | 134.9 | 36041.8 | 22 | 4 | 22 | 18.69 | Green |
| F8KAA6_SACUV | F8KAA6 | Alcohol dehydrogenase (Fragment) OS=Saccharomyces uvarum OX=230603 GN=adh1 PE=3 SV=1 | 134.9 | 36025.8 | 22 | 4 | 22 | 18.69 | Green |
| N1NZW1_YEASC | N1NZW1 | Glyceraldehyde-3-phosphate dehydrogenase OS=Saccharomyces cerevisiae (strain CEN.PK113-7D) OX=889517 GN=CENPK1137D_1249 PE=3 SV=1 | 841.3 | 35864.0 | 38 | 5 | 32 | 18.37 | Green |
| H0GWU4_SACCK | H0GWU4 | Glyceraldehyde-3-phosphate dehydrogenase OS=Saccharomyces cerevisiae x Saccharomyces kudriavzevii (strain VIN7) OX=1095631 GN=VIN7_7993 PE=3 SV=1 | 841.3 | 35832.0 | 38 | 5 | 32 | 18.37 | Green |
| H0GID8_SACCK | H0GID8 | Glyceraldehyde-3-phosphate dehydrogenase OS=Saccharomyces cerevisiae x Saccharomyces kudriavzevii (strain VIN7) OX=1095631 GN=VIN7_2630 PE=3 SV=1 | 841.3 | 35806.0 | 38 | 5 | 32 | 18.37 | Green |
| G2WGW5_YEASK | G2WGW5 | Glyceraldehyde-3-phosphate dehydrogenase OS=Saccharomyces cerevisiae (strain Kyokai no. 7 / NBRC 101557) OX=721032 GN=K7_TDH1 PE=3 SV=1 | 841.3 | 35864.0 | 38 | 5 | 32 | 18.37 | Green |
| C8ZBG2_YEAS8 | C8ZBG2 | Glyceraldehyde-3-phosphate dehydrogenase OS=Saccharomyces cerevisiae (strain Lalvin EC1118 / Prise de mousse) OX=643680 GN=EC1118_1J11_2025g PE=3 SV=2 | 841.3 | 35806.0 | 38 | 5 | 32 | 18.37 | Green |
| B3LQ59_YEAS1 | B3LQ59 | Glyceraldehyde-3-phosphate dehydrogenase OS=Saccharomyces cerevisiae (strain RM11-1a) OX=285006 GN=SCRG_03618 PE=3 SV=1 | 841.3 | 35806.0 | 38 | 5 | 32 | 18.37 | Green |
| A6ZPS3_YEAS7 | A6ZPS3 | Glyceraldehyde-3-phosphate dehydrogenase OS=Saccharomyces cerevisiae (strain YJM789) OX=307796 GN=TDH1 PE=3 SV=1 | 841.3 | 35806.0 | 38 | 5 | 32 | 18.37 | Green |
| A0A8H4BYL0_YEASX | A0A8H4BYL0 | Glyceraldehyde-3-phosphate dehydrogenase OS=Saccharomyces cerevisiae OX=4932 GN=TDH1 PE=3 SV=1 | 841.3 | 35864.0 | 38 | 5 | 32 | 18.37 | Green |
| A0A6C1E9W5_SACPS | A0A6C1E9W5 | Glyceraldehyde-3-phosphate dehydrogenase OS=Saccharomyces pastorianus OX=27292 GN=TDH1_2 PE=3 SV=1 | 841.3 | 35765.0 | 38 | 5 | 32 | 18.37 | Green |
| A0A6C1DV71_SACPS | A0A6C1DV71 | Glyceraldehyde-3-phosphate dehydrogenase OS=Saccharomyces pastorianus OX=27292 GN=TDH1_1 PE=3 SV=1 | 841.3 | 35806.0 | 38 | 5 | 32 | 18.37 | Green |
| G3P1_YEAST | P00360 | Glyceraldehyde-3-phosphate dehydrogenase 1 OS=Saccharomyces cerevisiae (strain ATCC 204508 / S288c) OX=559292 GN=TDH1 PE=1 SV=3 | 841.3 | 35864.0 | 38 | 5 | 32 | 18.37 | Green |
| K4GVE0_9SACH | K4GVE0 | Glycosylphosphatidylinositol (Fragment) OS=Saccharomyces cerevisiae x Saccharomyces kudriavzevii x Saccharomyces bayanus OX=1219479 GN=EGT2 PE=4 SV=1 | 441.2 | 12225.6 | 12 | 1 | 6 | 18.18 | Green |
| K4GVD7_9SACH | K4GVD7 | Glycosylphosphatidylinositol (Fragment) OS=Saccharomyces cerevisiae x Saccharomyces kudriavzevii OX=332112 GN=EGT2 PE=4 SV=1 | 441.2 | 12225.6 | 12 | 1 | 6 | 18.18 | Green |
| K4GUJ1_9SACH | K4GUJ1 | Glycosylphosphatidylinositol (Fragment) OS=Saccharomyces cerevisiae x Saccharomyces kudriavzevii OX=332112 GN=EGT2 PE=4 SV=1 | 441.2 | 12097.3 | 12 | 1 | 6 | 18.18 | Green |
| K4GUI9_SACKU | K4GUI9 | Glycosylphosphatidylinositol (Fragment) OS=Saccharomyces kudriavzevii OX=114524 GN=EGT2 PE=4 SV=1 | 441.2 | 12204.5 | 12 | 1 | 6 | 18.18 | Green |
| K4GT63_9SACH | K4GT63 | Glycosylphosphatidylinositol (Fragment) OS=Saccharomyces cerevisiae x Saccharomyces kudriavzevii OX=332112 GN=EGT2 PE=4 SV=1 | 441.2 | 12146.4 | 12 | 1 | 6 | 18.18 | Green |
| K4GSW5_9SACH | K4GSW5 | Glycosylphosphatidylinositol (Fragment) OS=Saccharomyces cerevisiae x Saccharomyces kudriavzevii OX=332112 GN=EGT2 PE=4 SV=1 | 441.2 | 12128.4 | 12 | 1 | 6 | 18.18 | Green |
| K4GSV8_SACKU | K4GSV8 | Glycosylphosphatidylinositol (Fragment) OS=Saccharomyces kudriavzevii OX=114524 GN=EGT2 PE=4 SV=1 | 441.2 | 12146.4 | 12 | 1 | 6 | 18.18 | Green |
| K4GSR4_9SACH | K4GSR4 | Glycosylphosphatidylinositol (Fragment) OS=Saccharomyces cerevisiae x Saccharomyces kudriavzevii x Saccharomyces bayanus OX=1219479 GN=EGT2 PE=4 SV=1 | 441.2 | 12128.4 | 12 | 1 | 6 | 18.18 | Green |
| K4GSQ8_9SACH | K4GSQ8 | Glycosylphosphatidylinositol (Fragment) OS=Saccharomyces cerevisiae x Saccharomyces kudriavzevii OX=332112 GN=EGT2 PE=4 SV=1 | 441.2 | 12230.5 | 12 | 1 | 8 | 18.18 | Green |
| I6QI47_YEASX | I6QI47 | Glycosylphosphatidylinositol-anchored cell wall endoglucanase (Fragment) OS=Saccharomyces cerevisiae OX=4932 GN=EGT2 PE=4 SV=1 | 441.2 | 12211.6 | 12 | 1 | 6 | 18.18 | Green |
| I6QI38_YEASX | I6QI38 | Glycosylphosphatidylinositol-anchored cell wall endoglucanase (Fragment) OS=Saccharomyces cerevisiae OX=4932 GN=EGT2 PE=4 SV=1 | 441.2 | 12170.4 | 12 | 1 | 6 | 18.18 | Green |
| I6QH62_YEASX | I6QH62 | Glycosylphosphatidylinositol-anchored cell wall endoglucanase (Fragment) OS=Saccharomyces cerevisiae OX=4932 GN=EGT2 PE=4 SV=1 | 441.2 | 12182.5 | 12 | 1 | 6 | 18.18 | Green |
| I6QCU4_YEASX | I6QCU4 | Glycosylphosphatidylinositol-anchored cell wall endoglucanase (Fragment) OS=Saccharomyces cerevisiae OX=4932 GN=EGT2 PE=4 SV=1 | 441.2 | 12199.5 | 12 | 1 | 6 | 18.18 | Green |
| K4GUI8_9SACH | K4GUI8 | Glycosylphosphatidylinositol (Fragment) OS=Saccharomyces cerevisiae x Saccharomyces kudriavzevii x Saccharomyces bayanus OX=1219479 GN=EGT2 PE=4 SV=1 | 441.2 | 12282.7 | 12 | 1 | 6 | 18.02 | Green |
| G2WEH2_YEASK | G2WEH2 | K7_Pdc6ap OS=Saccharomyces cerevisiae (strain Kyokai no. 7 / NBRC 101557) OX=721032 GN=K7_PDC6a PE=3 SV=1 | 134.0 | 11242.9 | 8 | 1 | 6 | 17.92 | Green |
| A0A1S5VBC7_YEASX | A0A1S5VBC7 | Egt2 (Fragment) OS=Saccharomyces cerevisiae OX=4932 GN=EGT2 PE=4 SV=1 | 441.2 | 12415.7 | 12 | 1 | 6 | 17.86 | Green |
| A0A1S5VBC4_YEASX | A0A1S5VBC4 | Egt2 (Fragment) OS=Saccharomyces cerevisiae OX=4932 GN=EGT2 PE=4 SV=1 | 441.2 | 12472.8 | 12 | 1 | 6 | 17.86 | Green |
| A0A1S5VBB5_YEASX | A0A1S5VBB5 | Egt2 (Fragment) OS=Saccharomyces cerevisiae OX=4932 GN=EGT2 PE=4 SV=1 | 441.2 | 12441.8 | 12 | 1 | 6 | 17.86 | Green |
| A0A1S5VBA4_YEASX | A0A1S5VBA4 | Egt2 (Fragment) OS=Saccharomyces cerevisiae OX=4932 GN=EGT2 PE=4 SV=1 | 441.2 | 12427.8 | 12 | 1 | 6 | 17.86 | Green |
| A0A1S5VBA3_YEASX | A0A1S5VBA3 | Egt2 (Fragment) OS=Saccharomyces cerevisiae OX=4932 GN=EGT2 PE=4 SV=1 | 441.2 | 12398.7 | 12 | 1 | 6 | 17.86 | Green |
| A0A1S5VB96_YEASX | A0A1S5VB96 | Egt2 (Fragment) OS=Saccharomyces cerevisiae OX=4932 GN=EGT2 PE=4 SV=1 | 441.2 | 12388.6 | 12 | 1 | 6 | 17.86 | Green |
| A0A1S5VB93_YEASX | A0A1S5VB93 | Egt2 (Fragment) OS=Saccharomyces cerevisiae OX=4932 GN=EGT2 PE=4 SV=1 | 441.2 | 12425.7 | 12 | 1 | 6 | 17.86 | Green |
| A0A1S5VB78_YEASX | A0A1S5VB78 | Egt2 (Fragment) OS=Saccharomyces cerevisiae OX=4932 GN=EGT2 PE=4 SV=1 | 441.2 | 12427.8 | 12 | 1 | 6 | 17.86 | Green |
| A0A1S5VB61_YEASX | A0A1S5VB61 | Egt2 (Fragment) OS=Saccharomyces cerevisiae OX=4932 GN=EGT2 PE=4 SV=1 | 441.2 | 12370.6 | 12 | 1 | 6 | 17.86 | Green |
| A0A1S5VB53_YEASX | A0A1S5VB53 | Egt2 (Fragment) OS=Saccharomyces cerevisiae OX=4932 GN=EGT2 PE=4 SV=1 | 441.2 | 12428.7 | 12 | 1 | 6 | 17.86 | Green |
| A0A6C1EAE6_SACPS | A0A6C1EAE6 | Phosphoglycerate mutase OS=Saccharomyces pastorianus OX=27292 GN=GPM1_2 PE=3 SV=1 | 205.1 | 27468.4 | 6 | 1 | 22 | 17.41 | Green |
| H0GQJ2_SACCK | H0GQJ2 | Fructose-bisphosphate aldolase OS=Saccharomyces cerevisiae x Saccharomyces kudriavzevii (strain VIN7) OX=1095631 GN=VIN7_5279 PE=3 SV=1 | 196.4 | 37793.6 | 28 | 4 | 32 | 17.01 | Green |
| H0GK82_SACCK | H0GK82 | Yps3p OS=Saccharomyces cerevisiae x Saccharomyces kudriavzevii (strain VIN7) OX=1095631 GN=VIN7_3267 PE=3 SV=1 | 156.2 | 54779.6 | 31 | 5 | 33 | 16.73 | Green |
| C8ZD91_YEAS8 | C8ZD91 | Yps3p OS=Saccharomyces cerevisiae (strain Lalvin EC1118 / Prise de mousse) OX=643680 GN=EC1118_1L10_2047g PE=3 SV=1 | 156.2 | 54793.6 | 31 | 5 | 33 | 16.73 | Green |
| B5VN56_YEAS6 | B5VN56 | YLR121Cp-like protein OS=Saccharomyces cerevisiae (strain AWRI1631) OX=545124 GN=AWRI1631_121690 PE=3 SV=1 | 156.2 | 54779.6 | 31 | 5 | 33 | 16.73 | Green |
| B3LT85_YEAS1 | B3LT85 | Aspartic proteinase yapsin-3 OS=Saccharomyces cerevisiae (strain RM11-1a) OX=285006 GN=SCRG_05103 PE=3 SV=1 | 156.2 | 54779.6 | 31 | 5 | 33 | 16.73 | Green |
| ENO_EREGS | Q756H2 | Enolase OS=Eremothecium gossypii (strain ATCC 10895 / CBS 109.51 / FGSC 9923 / NRRL Y-1056) OX=284811 GN=ENO PE=3 SV=1 | 3976.1 | 46688.9 | 98 | 6 | 35 | 16.25 | Green |
| N1P7U8_YEASC | N1P7U8 | Fructose-bisphosphate aldolase OS=Saccharomyces cerevisiae (strain CEN.PK113-7D) OX=889517 GN=CENPK1137D_945 PE=3 SV=1 | 196.4 | 39906.0 | 28 | 4 | 33 | 16.16 | Green |
| H0GJ73_SACCK | H0GJ73 | Fructose-bisphosphate aldolase OS=Saccharomyces cerevisiae x Saccharomyces kudriavzevii (strain VIN7) OX=1095631 GN=VIN7_2949 PE=3 SV=1 | 196.4 | 39906.0 | 28 | 4 | 33 | 16.16 | Green |
| G2WHX2_YEASK | G2WHX2 | Fructose-bisphosphate aldolase OS=Saccharomyces cerevisiae (strain Kyokai no. 7 / NBRC 101557) OX=721032 GN=K7_FBA1 PE=3 SV=1 | 196.4 | 39891.9 | 28 | 4 | 33 | 16.16 | Green |
| C8ZCB2_YEAS8 | C8ZCB2 | Fructose-bisphosphate aldolase OS=Saccharomyces cerevisiae (strain Lalvin EC1118 / Prise de mousse) OX=643680 GN=EC1118_1K5_1860g PE=3 SV=1 | 196.4 | 39906.0 | 28 | 4 | 33 | 16.16 | Green |
| B3LR31_YEAS1 | B3LR31 | Fructose-bisphosphate aldolase OS=Saccharomyces cerevisiae (strain RM11-1a) OX=285006 GN=SCRG_03960 PE=3 SV=1 | 196.4 | 39893.0 | 28 | 4 | 33 | 16.16 | Green |
| A6ZZQ6_YEAS7 | A6ZZQ6 | Fructose-bisphosphate aldolase OS=Saccharomyces cerevisiae (strain YJM789) OX=307796 GN=FBA1 PE=3 SV=1 | 196.4 | 39920.0 | 28 | 4 | 33 | 16.16 | Green |
| A0A8H4FAG4_YEASX | A0A8H4FAG4 | Fructose-bisphosphate aldolase OS=Saccharomyces cerevisiae OX=4932 GN=FBA1 PE=3 SV=1 | 196.4 | 39906.0 | 28 | 4 | 33 | 16.16 | Green |
| A0A6C1DUM3_SACPS | A0A6C1DUM3 | Fructose-bisphosphate aldolase OS=Saccharomyces pastorianus OX=27292 GN=FBA1_1 PE=3 SV=1 | 196.4 | 39906.0 | 28 | 4 | 33 | 16.16 | Green |
| ALF_YEAST | P14540 | Fructose-bisphosphate aldolase OS=Saccharomyces cerevisiae (strain ATCC 204508 / S288c) OX=559292 GN=FBA1 PE=1 SV=3 | 196.4 | 39906.0 | 28 | 4 | 33 | 16.16 | Green |
| H0GXC9_SACCK | H0GXC9 | Phosphoglycerate mutase OS=Saccharomyces cerevisiae x Saccharomyces kudriavzevii (strain VIN7) OX=1095631 GN=VIN7_8276 PE=3 SV=1 | 410.0 | 27638.6 | 24 | 2 | 21 | 14.98 | Green |
| Q6XQ69_SACPS | Q6XQ69 | Alcohol dehydrogenase 2 OS=Saccharomyces pastorianus OX=27292 GN=ADH2 PE=3 SV=1 | 276.0 | 37200.2 | 29 | 5 | 28 | 12.93 | Green |
| M9VDX4_YEASX | M9VDX4 | Phenylacetaldehyde dehydrogenase OS=Saccharomyces cerevisiae OX=4932 PE=3 SV=1 | 276.0 | 37200.2 | 29 | 5 | 28 | 12.93 | Green |
| H0GLJ4_SACCK | H0GLJ4 | Adh2p OS=Saccharomyces cerevisiae x Saccharomyces kudriavzevii (strain VIN7) OX=1095631 GN=VIN7_3937 PE=3 SV=1 | 276.0 | 37200.2 | 29 | 5 | 28 | 12.93 | Green |
| C8ZFH1_YEAS8 | C8ZFH1 | Adh2p OS=Saccharomyces cerevisiae (strain Lalvin EC1118 / Prise de mousse) OX=643680 GN=EC1118_1M3_5182g PE=3 SV=1 | 276.0 | 37200.2 | 29 | 5 | 28 | 12.93 | Green |
| B3LMJ1_YEAS1 | B3LMJ1 | Alcohol dehydrogenase II OS=Saccharomyces cerevisiae (strain RM11-1a) OX=285006 GN=SCRG_02200 PE=3 SV=1 | 276.0 | 37170.2 | 29 | 5 | 24 | 12.93 | Green |
| A0A0B4VIL5_YEASX | A0A0B4VIL5 | Glucose-repressible alcohol dehydrogenase II (Fragment) OS=Saccharomyces cerevisiae OX=4932 GN=ADH2 PE=3 SV=1 | 263.1 | 34902.6 | 24 | 4 | 26 | 11.59 | Green |
| B2G464_ZYGRO | B2G464 | NADPH dehydrogenase 2 and NADPH dehydrogenase 3 OS=Zygosaccharomyces rouxii OX=4956 GN=Zr_OYE2 and Zr_OYE3 PE=4 SV=1 | 165.7 | 37065.4 | 10 | 1 | 19 | 11.28 | Green |
| A0A0B4VK10_YEASX | A0A0B4VK10 | Glucose-repressible alcohol dehydrogenase II (Fragment) OS=Saccharomyces cerevisiae OX=4932 GN=ADH2 PE=3 SV=1 | 263.1 | 36123.0 | 24 | 4 | 26 | 11.24 | Green |
| A0A0B4VIM0_YEASX | A0A0B4VIM0 | Glucose-repressible alcohol dehydrogenase II (Fragment) OS=Saccharomyces cerevisiae OX=4932 GN=ADH2 PE=3 SV=1 | 263.1 | 36122.0 | 24 | 4 | 26 | 11.24 | Green |
| H0GXI1_SACCK | H0GXI1 | Fructose-bisphosphate aldolase OS=Saccharomyces cerevisiae x Saccharomyces kudriavzevii (strain VIN7) OX=1095631 GN=VIN7_8353 PE=3 SV=1 | 171.2 | 39712.7 | 21 | 5 | 32 | 11.14 | Green |
| C7GP14_YEAS2 | C7GP14 | Fructose-bisphosphate aldolase OS=Saccharomyces cerevisiae (strain JAY291) OX=574961 GN=FBA1 PE=3 SV=1 | 171.2 | 39905.0 | 22 | 6 | 33 | 11.14 | Green |
| A0A6C1EBI4_SACPS | A0A6C1EBI4 | Fructose-bisphosphate aldolase OS=Saccharomyces pastorianus OX=27292 GN=FBA1_2 PE=3 SV=1 | 162.3 | 39648.8 | 18 | 4 | 31 | 11.14 | Green |
| H0GY85_SACCK | H0GY85 | Yps3p OS=Saccharomyces cerevisiae x Saccharomyces kudriavzevii (strain VIN7) OX=1095631 GN=VIN7_8665 PE=3 SV=1 | 589.8 | 52765.1 | 39 | 3 | 26 | 11.11 | Green |
| H0GVG6_SACCK | H0GVG6 | Scw4p OS=Saccharomyces cerevisiae x Saccharomyces kudriavzevii (strain VIN7) OX=1095631 GN=VIN7_7410 PE=4 SV=1 | 1700.7 | 32790.9 | 43 | 3 | 12 | 11.08 | Green |
| G2WFX0_YEASK | G2WFX0 | acid phosphatase OS=Saccharomyces cerevisiae (strain Kyokai no. 7 / NBRC 101557) OX=721032 GN=K7_PHO11 PE=3 SV=1 | 189.3 | 53123.9 | 15 | 3 | 31 | 10.49 | Green |
| G2WL12_YEASK | G2WL12 | K7_Adh2p OS=Saccharomyces cerevisiae (strain Kyokai no. 7 / NBRC 101557) OX=721032 GN=K7_ADH2 PE=3 SV=1 | 244.4 | 37215.2 | 26 | 4 | 27 | 10.34 | Green |
| A0A3G3NDG3_YEASX | A0A3G3NDG3 | Alcohol dehydrogenase OS=Saccharomyces cerevisiae OX=4932 GN=ADH1 PE=3 SV=1 | 244.4 | 37244.3 | 26 | 4 | 29 | 10.34 | Green |
| A0A0B4VJL3_YEASX | A0A0B4VJL3 | ADH2 isoform 1 OS=Saccharomyces cerevisiae OX=4932 GN=ADH2 PE=3 SV=1 | 244.4 | 37188.2 | 26 | 4 | 27 | 10.34 | Green |
| ADH2_YEAST | P00331 | Alcohol dehydrogenase 2 OS=Saccharomyces cerevisiae (strain ATCC 204508 / S288c) OX=559292 GN=ADH2 PE=1 SV=3 | 244.4 | 37188.2 | 26 | 4 | 27 | 10.34 | Green |
| B5VN62_YEAS6 | B5VN62 | YLR134Wp-like protein (Fragment) OS=Saccharomyces cerevisiae (strain AWRI1631) OX=545124 GN=AWRI1631_121820 PE=3 SV=1 | 134.0 | 21258.2 | 8 | 1 | 9 | 9.74 | Green |
| A0A0B4VK24_YEASX | A0A0B4VK24 | Glucose-repressible alcohol dehydrogenase II (Fragment) OS=Saccharomyces cerevisiae OX=4932 GN=ADH2 PE=3 SV=1 | 248.4 | 33903.5 | 19 | 3 | 23 | 9.4 | Green |
| ADH2_KLUMA | Q9P4C2 | Alcohol dehydrogenase 2 OS=Kluyveromyces marxianus OX=4911 GN=ADH2 PE=3 SV=3 | 241.8 | 37481.5 | 24 | 3 | 27 | 9.2 | Green |
| A0A0B4VIV5_YEASX | A0A0B4VIV5 | Glucose-repressible alcohol dehydrogenase II (Fragment) OS=Saccharomyces cerevisiae OX=4932 GN=ADH2 PE=3 SV=1 | 231.5 | 34917.6 | 21 | 3 | 25 | 8.84 | Green |
| A0A0B4VIU1_YEASX | A0A0B4VIU1 | Glucose-repressible alcohol dehydrogenase II (Fragment) OS=Saccharomyces cerevisiae OX=4932 GN=ADH2 PE=3 SV=1 | 231.5 | 34918.6 | 21 | 3 | 25 | 8.84 | Green |
| A0A0B4VIL2_YEASX | A0A0B4VIL2 | Glucose-repressible alcohol dehydrogenase II (Fragment) OS=Saccharomyces cerevisiae OX=4932 GN=ADH2 PE=3 SV=1 | 231.5 | 34946.6 | 21 | 3 | 27 | 8.84 | Green |
| A0A0B4VJK0_YEASX | A0A0B4VJK0 | Glucose-repressible alcohol dehydrogenase II (Fragment) OS=Saccharomyces cerevisiae OX=4932 GN=ADH2 PE=3 SV=1 | 231.5 | 36139.0 | 21 | 3 | 25 | 8.58 | Green |
| A0A6C1E788_SACPS | A0A6C1E788 | Nuclear localization sequence binding protein OS=Saccharomyces pastorianus OX=27292 GN=NSR1 PE=4 SV=1 | 155.1 | 41475.3 | 9 | 1 | 36 | 7.79 | Green |
| N1P8D9_YEASC | N1P8D9 | acid phosphatase OS=Saccharomyces cerevisiae (strain CEN.PK113-7D) OX=889517 GN=CENPK1137D_4639 PE=3 SV=1 | 165.1 | 53212.9 | 16 | 3 | 35 | 6.85 | Green |
| G2W9A0_YEASK | G2W9A0 | acid phosphatase OS=Saccharomyces cerevisiae (strain Kyokai no. 7 / NBRC 101557) OX=721032 GN=K7_PHO5 PE=3 SV=1 | 165.1 | 53471.0 | 20 | 4 | 32 | 6.85 | Green |
| D3UEI8_YEAS8 | D3UEI8 | acid phosphatase OS=Saccharomyces cerevisiae (strain Lalvin EC1118 / Prise de mousse) OX=643680 GN=EC1118_1B15_2377g PE=3 SV=1 | 165.1 | 53428.9 | 20 | 4 | 32 | 6.85 | Green |
| A0A8H4F6L0_YEASX | A0A8H4F6L0 | acid phosphatase OS=Saccharomyces cerevisiae OX=4932 GN=PHO5 PE=3 SV=1 | 165.1 | 53428.9 | 20 | 4 | 32 | 6.85 | Green |
| A0A6C1DMH2_SACPS | A0A6C1DMH2 | acid phosphatase OS=Saccharomyces pastorianus OX=27292 GN=PHO5 PE=3 SV=1 | 165.1 | 53471.0 | 20 | 4 | 32 | 6.85 | Green |
| PPA5_YEAST | P00635 | Repressible acid phosphatase OS=Saccharomyces cerevisiae (strain ATCC 204508 / S288c) OX=559292 GN=PHO5 PE=1 SV=2 | 165.1 | 53428.9 | 20 | 4 | 32 | 6.85 | Green |
| H0GCF4_SACCK | H0GCF4 | acid phosphatase OS=Saccharomyces cerevisiae x Saccharomyces kudriavzevii (strain VIN7) OX=1095631 GN=VIN7_0254 PE=3 SV=1 | 158.7 | 53477.0 | 18 | 3 | 30 | 6.85 | Green |
| H0GCF3_SACCK | H0GCF3 | acid phosphatase OS=Saccharomyces cerevisiae x Saccharomyces kudriavzevii (strain VIN7) OX=1095631 GN=VIN7_0253 PE=3 SV=1 | 158.7 | 53275.0 | 14 | 2 | 33 | 6.85 | Green |
| D3UEI7_YEAS8 | D3UEI7 | acid phosphatase OS=Saccharomyces cerevisiae (strain Lalvin EC1118 / Prise de mousse) OX=643680 GN=EC1118_1B15_2366g PE=3 SV=1 | 158.7 | 53275.0 | 14 | 2 | 33 | 6.85 | Green |
| B3LN59_YEAS1 | B3LN59 | acid phosphatase OS=Saccharomyces cerevisiae (strain RM11-1a) OX=285006 GN=SCRG_02872 PE=3 SV=1 | 158.7 | 53275.9 | 14 | 2 | 34 | 6.85 | Green |
| B3LN58_YEAS1 | B3LN58 | acid phosphatase OS=Saccharomyces cerevisiae (strain RM11-1a) OX=285006 GN=SCRG_02871 PE=3 SV=1 | 158.7 | 53477.0 | 18 | 3 | 30 | 6.85 | Green |
| A6ZL40_YEAS7 | A6ZL40 | acid phosphatase OS=Saccharomyces cerevisiae (strain YJM789) OX=307796 GN=PHO5 PE=3 SV=1 | 158.7 | 53477.0 | 18 | 3 | 30 | 6.85 | Green |
| A6ZL39_YEAS7 | A6ZL39 | acid phosphatase OS=Saccharomyces cerevisiae (strain YJM789) OX=307796 GN=PHO3 PE=3 SV=1 | 158.7 | 53230.8 | 14 | 2 | 32 | 6.85 | Green |
| A0A8H4BUB0_YEASX | A0A8H4BUB0 | acid phosphatase OS=Saccharomyces cerevisiae OX=4932 GN=PHO3 PE=3 SV=1 | 158.7 | 53232.9 | 14 | 2 | 33 | 6.85 | Green |
| PPA3_YEAST | P24031 | Constitutive acid phosphatase OS=Saccharomyces cerevisiae (strain ATCC 204508 / S288c) OX=559292 GN=PHO3 PE=1 SV=2 | 158.7 | 53232.9 | 14 | 2 | 33 | 6.85 | Green |
| H0GSF7_SACCK | H0GSF7 | Phosphatidylglycerol/phosphatidylinositol transfer protein OS=Saccharomyces cerevisiae x Saccharomyces kudriavzevii (strain VIN7) OX=1095631 GN=VIN7_6098 PE=3 SV=1 | 91.7 | 19182.9 | 13 | 3 | 11 | 6.36 | Green |
| A0A1V2LI93_PICKU | A0A1V2LI93 | glyceraldehyde-3-phosphate dehydrogenase (phosphorylating) OS=Pichia kudriavzevii OX=4909 GN=BOH78_4052 PE=3 SV=1 | 141.3 | 27341.1 | 13 | 1 | 27 | 5.93 | Green |
| A0A061B1C1_CYBFA | A0A061B1C1 | phosphopyruvate hydratase OS=Cyberlindnera fabianii OX=36022 GN=BON22_4808 PE=3 SV=1 | 88.4 | 46793.1 | 22 | 4 | 35 | 5.73 | Green |
| A0A1X7R416_9SACH | A0A1X7R416 | Similar to Saccharomyces cerevisiae YGR279C SCW4 Cell wall protein with similarity to glucanases OS=Kazachstania saulgeensis OX=1789683 GN=KASA_0N03905G PE=3 SV=1 | 128.2 | 37260.9 | 13 | 1 | 17 | 4.52 | Green |
| A0A061B2A1_CYBFA | A0A061B2A1 | Glyceraldehyde-3-phosphate dehydrogenase OS=Cyberlindnera fabianii OX=36022 GN=BON22_5334 PE=3 SV=1 | 141.3 | 36156.3 | 13 | 1 | 31 | 4.5 | Green |
| A0A6C1DYU4_SACPS | A0A6C1DYU4 | Glycosylphosphatidylinositol (GPI)-anchored cell wall endoglucanase OS=Saccharomyces pastorianus OX=27292 GN=EGT2 PE=4 SV=1 | 460.5 | 104855.9 | 21 | 3 | 30 | 4.29 | Green |
| A0A061BBA7_CYBFA | A0A061BBA7 | Superoxide dismutase OS=Cyberlindnera fabianii OX=36022 GN=BON22_4688 PE=3 SV=1 | 100.3 | 24273.2 | 8 | 1 | 13 | 4.17 | Green |
| N1NYC3_YEASC | N1NYC3 | Egt2p OS=Saccharomyces cerevisiae (strain CEN.PK113-7D) OX=889517 GN=CENPK1137D_2763 PE=4 SV=1 | 460.5 | 108810.3 | 21 | 3 | 31 | 4.13 | Green |
| G2WLE4_YEASK | G2WLE4 | K7_Egt2p OS=Saccharomyces cerevisiae (strain Kyokai no. 7 / NBRC 101557) OX=721032 GN=K7_EGT2 PE=4 SV=1 | 460.5 | 108751.2 | 21 | 3 | 31 | 4.13 | Green |
| A0A8H4BXH9_YEASX | A0A8H4BXH9 | EGT2 isoform 1 OS=Saccharomyces cerevisiae OX=4932 GN=EGT2 PE=4 SV=1 | 460.5 | 108810.3 | 21 | 3 | 31 | 4.13 | Green |
| EGT2_YEAST | P42835 | Protein EGT2 OS=Saccharomyces cerevisiae (strain ATCC 204508 / S288c) OX=559292 GN=EGT2 PE=2 SV=2 | 460.5 | 108810.3 | 21 | 3 | 31 | 4.13 | Green |
| A6ZSF5_YEAS7 | A6ZSF5 | Glycosylphosphatidylinositol (GPI)-anchored cell wall endoglucanase OS=Saccharomyces cerevisiae (strain YJM789) OX=307796 GN=EGT2 PE=4 SV=1 | 460.5 | 112607.4 | 21 | 3 | 32 | 3.99 | Green |
| B5VJ31_YEAS6 | B5VJ31 | branched-chain-2-oxoacid decarboxylase (Fragment) OS=Saccharomyces cerevisiae (strain AWRI1631) OX=545124 GN=AWRI1631_73100 PE=3 SV=1 | 134.0 | 53904.7 | 8 | 1 | 31 | 3.85 | Green |
| H0H1K2_SACCK | H0H1K2 | Scd5p OS=Saccharomyces cerevisiae x Saccharomyces kudriavzevii (strain VIN7) OX=1095631 GN=VIN7_10144 PE=4 SV=1 | 103.5 | 97295.6 | 28 | 4 | 38 | 3.42 | Green |
| N1P9V9_YEASC | N1P9V9 | branched-chain-2-oxoacid decarboxylase OS=Saccharomyces cerevisiae (strain CEN.PK113-7D) OX=889517 GN=CENPK1137D_3053 PE=3 SV=1 | 137.5 | 61590.5 | 9 | 2 | 35 | 3.37 | Green |
| H0GY93_SACCK | H0GY93 | branched-chain-2-oxoacid decarboxylase OS=Saccharomyces cerevisiae x Saccharomyces kudriavzevii (strain VIN7) OX=1095631 GN=VIN7_8674 PE=3 SV=1 | 137.5 | 62195.2 | 9 | 2 | 34 | 3.37 | Green |
| H0GK91_SACCK | H0GK91 | branched-chain-2-oxoacid decarboxylase OS=Saccharomyces cerevisiae x Saccharomyces kudriavzevii (strain VIN7) OX=1095631 GN=VIN7_3276 PE=3 SV=1 | 137.5 | 62140.3 | 9 | 2 | 35 | 3.37 | Green |
| H0GGK8_SACCK | H0GGK8 | branched-chain-2-oxoacid decarboxylase OS=Saccharomyces cerevisiae x Saccharomyces kudriavzevii (strain VIN7) OX=1095631 GN=VIN7_1877 PE=3 SV=1 | 137.5 | 61621.5 | 9 | 2 | 35 | 3.37 | Green |
| C8ZDA3_YEAS8 | C8ZDA3 | branched-chain-2-oxoacid decarboxylase OS=Saccharomyces cerevisiae (strain Lalvin EC1118 / Prise de mousse) OX=643680 GN=EC1118_1L10_2190g PE=3 SV=1 | 137.5 | 62140.3 | 9 | 2 | 35 | 3.37 | Green |
| C8Z8Y1_YEAS8 | C8Z8Y1 | branched-chain-2-oxoacid decarboxylase OS=Saccharomyces cerevisiae (strain Lalvin EC1118 / Prise de mousse) OX=643680 GN=EC1118_1G1_3983g PE=3 SV=1 | 137.5 | 61621.5 | 9 | 2 | 35 | 3.37 | Green |
| B3LT95_YEAS1 | B3LT95 | branched-chain-2-oxoacid decarboxylase OS=Saccharomyces cerevisiae (strain RM11-1a) OX=285006 GN=SCRG_05113 PE=3 SV=1 | 137.5 | 62170.3 | 9 | 2 | 35 | 3.37 | Green |
| B3LID9_YEAS1 | B3LID9 | branched-chain-2-oxoacid decarboxylase OS=Saccharomyces cerevisiae (strain RM11-1a) OX=285006 GN=SCRG_00930 PE=3 SV=1 | 137.5 | 61621.5 | 9 | 2 | 35 | 3.37 | Green |
| G2WIX3_YEASK | G2WIX3 | branched-chain-2-oxoacid decarboxylase OS=Saccharomyces cerevisiae (strain Kyokai no. 7 / NBRC 101557) OX=721032 GN=K7_PDC5 PE=3 SV=1 | 137.5 | 62131.3 | 9 | 2 | 35 | 3.37 | Green |
| A7A133_YEAS7 | A7A133 | branched-chain-2-oxoacid decarboxylase OS=Saccharomyces cerevisiae (strain YJM789) OX=307796 GN=PDC5 PE=3 SV=1 | 137.5 | 62131.3 | 9 | 2 | 35 | 3.37 | Green |
| A0A8H4F8E8_YEASX | A0A8H4F8E8 | branched-chain-2-oxoacid decarboxylase OS=Saccharomyces cerevisiae OX=4932 GN=PDC5 PE=3 SV=1 | 137.5 | 62140.3 | 9 | 2 | 35 | 3.37 | Green |
| A0A6C1EC67_SACPS | A0A6C1EC67 | branched-chain-2-oxoacid decarboxylase OS=Saccharomyces pastorianus OX=27292 GN=PDC5_2 PE=3 SV=1 | 137.5 | 62006.1 | 9 | 2 | 33 | 3.37 | Green |
| A0A6C1E8A3_SACPS | A0A6C1E8A3 | branched-chain-2-oxoacid decarboxylase OS=Saccharomyces pastorianus OX=27292 GN=PDC6_2 PE=3 SV=1 | 137.5 | 61786.7 | 9 | 2 | 40 | 3.37 | Green |
| A0A6C1DWX6_SACPS | A0A6C1DWX6 | branched-chain-2-oxoacid decarboxylase OS=Saccharomyces pastorianus OX=27292 GN=PDC5_1 PE=3 SV=1 | 137.5 | 62131.3 | 9 | 2 | 35 | 3.37 | Green |
| A0A6C1DSN7_SACPS | A0A6C1DSN7 | branched-chain-2-oxoacid decarboxylase OS=Saccharomyces pastorianus OX=27292 GN=PDC6_1 PE=3 SV=1 | 137.5 | 61665.5 | 9 | 2 | 35 | 3.37 | Green |
| PDC5_YEAST | P16467 | Pyruvate decarboxylase isozyme 2 OS=Saccharomyces cerevisiae (strain ATCC 204508 / S288c) OX=559292 GN=PDC5 PE=1 SV=4 | 137.5 | 62140.3 | 9 | 2 | 35 | 3.37 | Green |
| C7GWX6_YEAS2 | C7GWX6 | branched-chain-2-oxoacid decarboxylase OS=Saccharomyces cerevisiae (strain JAY291) OX=574961 GN=PDC6 PE=3 SV=1 | 134.0 | 61637.5 | 8 | 1 | 35 | 3.37 | Green |
| A6ZV69_YEAS7 | A6ZV69 | branched-chain-2-oxoacid decarboxylase OS=Saccharomyces cerevisiae (strain YJM789) OX=307796 GN=PDC6 PE=3 SV=1 | 134.0 | 61637.5 | 8 | 1 | 35 | 3.37 | Green |
| A0A8H4C1R4_YEASX | A0A8H4C1R4 | branched-chain-2-oxoacid decarboxylase OS=Saccharomyces cerevisiae OX=4932 GN=PDC6 PE=3 SV=1 | 134.0 | 61637.5 | 8 | 1 | 35 | 3.37 | Green |
| PDC6_YEAST | P26263 | Pyruvate decarboxylase isozyme 3 OS=Saccharomyces cerevisiae (strain ATCC 204508 / S288c) OX=559292 GN=PDC6 PE=1 SV=3 | 134.0 | 61637.5 | 8 | 1 | 35 | 3.37 | Green |
| Q75CQ3_EREGS | Q75CQ3 | ACL134Cp OS=Eremothecium gossypii (strain ATCC 10895 / CBS 109.51 / FGSC 9923 / NRRL Y-1056) OX=284811 GN=AGOS_ACL134C PE=3 SV=1 | 137.5 | 64473.6 | 9 | 2 | 35 | 3.24 | Green |
| H0GM17_SACCK | H0GM17 | Egt2p OS=Saccharomyces cerevisiae x Saccharomyces kudriavzevii (strain VIN7) OX=1095631 GN=VIN7_3960 PE=4 SV=1 | 441.2 | 104774.8 | 12 | 1 | 30 | 1.99 | Green |
| B5VQ94_YEAS6 | B5VQ94 | YNL327Wp-like protein (Fragment) OS=Saccharomyces cerevisiae (strain AWRI1631) OX=545124 GN=AWRI1631_140090 PE=4 SV=1 | 441.2 | 104658.3 | 12 | 1 | 27 | 1.99 | Green |
| C8ZGJ8_YEAS8 | C8ZGJ8 | Egt2p OS=Saccharomyces cerevisiae (strain Lalvin EC1118 / Prise de mousse) OX=643680 GN=EC1118_1N9_0056g PE=4 SV=1 | 441.2 | 108585.0 | 12 | 1 | 31 | 1.92 | Green |
| C7GWS2_YEAS2 | C7GWS2 | Egt2p OS=Saccharomyces cerevisiae (strain JAY291) OX=574961 GN=EGT2 PE=4 SV=1 | 441.2 | 108675.1 | 12 | 1 | 31 | 1.92 | Green |
| B3LPF9_YEAS1 | B3LPF9 | Protein EGT2 OS=Saccharomyces cerevisiae (strain RM11-1a) OX=285006 GN=SCRG_03449 PE=4 SV=1 | 441.2 | 108675.1 | 12 | 1 | 31 | 1.92 | Green |
| H0GZT7_SACCK | H0GZT7 | Egt2p OS=Saccharomyces cerevisiae x Saccharomyces kudriavzevii (strain VIN7) OX=1095631 GN=VIN7_9377 PE=4 SV=1 | 441.2 | 112034.4 | 12 | 1 | 32 | 1.88 | Green |

**Table S2. Comparative proteomic analysis of 38 proteins that were significantly common to the two samples were considered**

| **Accession** | **Peptide count** | **Unique peptides** | **Confidence score** | **Anova (p)** | **Description** | **EV@Y24** | | | **EV@Y72** | | |
| --- | --- | --- | --- | --- | --- | --- | --- | --- | --- | --- | --- |
|  |  |  |  |  |  | **1** | **2** | **3** | **1** | **2** | **3** |
| P53334 | 14 | 14 | 122.58 | 0.00437 | Probable family 17 glucosidase SCW4 OS=Saccharomyces cerevisiae (strain ATCC 204508 / S288c) OX=559292 GN=SCW4 PE=1 SV=1 | 416618.96 | 423606.29 | 401330.79 | 570360.50 | 552393.20 | 567782.90 |
| Q04951 | 14 | 14 | 110.41 | 0.02249 | Probable family 17 glucosidase SCW10 OS=Saccharomyces cerevisiae (strain ATCC 204508 / S288c) OX=559292 GN=SCW10 PE=1 SV=1 | 255989.25 | 234540.27 | 241708.13 | 192967.73 | 174085.96 | 181698.93 |
| P00447 | 9 | 9 | 75.80 | 0.13347 | Superoxide dismutase [Mn], mitochondrial OS=Saccharomyces cerevisiae (strain ATCC 204508 / S288c) OX=559292 GN=SOD2 PE=1 SV=1 | 510294.21 | 667102.50 | 860751.07 | 626189.08 | 481627.35 | 510691.76 |
| S5RK20 | 10 | 7 | 59.90 | 0.00002 | Alcohol dehydrogenase OS=Saccharomyces cerevisiae OX=4932 GN=ADH1 PE=3 SV=1 | 109432.10 | 105798.58 | 115834.77 | 67872.94 | 78717.23 | 77189.40 |
| P00924 | 30 | 6 | 268.73 | 0.00000 | Enolase 1 OS=Saccharomyces cerevisiae (strain ATCC 204508 / S288c) OX=559292 GN=ENO1 PE=1 SV=3 | 29141.74 | 32692.83 | 38888.20 | 96412.57 | 90179.08 | 96866.15 |
| P06169 | 6 | 6 | 56.27 | 0.02321 | Pyruvate decarboxylase isozyme 1 OS=Saccharomyces cerevisiae (strain ATCC 204508 / S288c) OX=559292 GN=PDC1 PE=1 SV=7 | 96007.34 | 95713.15 | 95321.74 | 112069.35 | 116273.78 | 113536.91 |
| B3LR31 | 6 | 5 | 42.67 | 0.00000 | Fructose-bisphosphate aldolase OS=Saccharomyces cerevisiae (strain RM11-1a) OX=285006 GN=SCRG_03960 PE=3 SV=1 | 138035.93 | 135560.73 | 134363.51 | 78298.81 | 71207.19 | 76760.99 |
| Q12408 | 5 | 5 | 22.21 | 0.00354 | Phosphatidylglycerol/phosphatidylinositol transfer protein OS=Saccharomyces cerevisiae (strain ATCC 204508 / S288c) OX=559292 GN=NPC2 PE=1 SV=1 | 22982.74 | 24648.99 | 22698.02 | 31336.87 | 37503.26 | 33042.97 |
| A0A6A5PXB1 | 11 | 4 | 89.49 | 0.02843 | Glyceraldehyde-3-phosphate dehydrogenase OS=Saccharomyces cerevisiae OX=4932 GN=TDH2 PE=3 SV=1 | 19062.93 | 18445.50 | 24929.18 | 28422.06 | 20945.88 | 24015.02 |
| H0GUF7 | 4 | 4 | 30.58 | 0.00000 | Phosphotransferase OS=Saccharomyces cerevisiae x Saccharomyces kudriavzevii (strain VIN7) OX=1095631 GN=VIN7_6946 PE=3 SV=1 | 16554.72 | 17660.07 | 22414.43 | 62748.53 | 63380.10 | 68417.03 |
| A0A1V2LJI2 | 4 | 4 | 25.08 | 0.00000 | Transcriptional activator HAP2 OS=Pichia kudriavzevii OX=4909 GN=BOH78_3540 PE=3 SV=1 | 16117.60 | 16844.71 | 17082.31 | 8542.00 | 8760.51 | 8938.56 |
| H0GK82 | 4 | 4 | 39.69 | 0.14336 | Yps3p OS=Saccharomyces cerevisiae x Saccharomyces kudriavzevii (strain VIN7) OX=1095631 GN=VIN7_3267 PE=3 SV=1 | 103721.57 | 96001.84 | 108330.05 | 102891.59 | 90779.36 | 115698.83 |
| A0A1V2L480 | 3 | 3 | 17.60 | 0.00000 | Ubiquitin-binding protein CUE5 OS=Cyberlindnera fabianii OX=36022 GN=BON22_3650 PE=4 SV=1 | 37459.23 | 39109.37 | 31244.36 | 127507.97 | 135378.01 | 134128.55 |
| B5VP44 | 3 | 3 | 18.98 | 0.00000 | YML085Cp-like protein OS=Saccharomyces cerevisiae (strain AWRI1631) OX=545124 GN=AWRI1631_130500 PE=3 SV=1 | 126013.34 | 128115.75 | 122821.55 | 59329.36 | 57963.08 | 59741.75 |
| B3LQU9 | 3 | 3 | 28.45 | 0.42844 | Phosphoglycerate mutase OS=Saccharomyces cerevisiae (strain RM11-1a) OX=285006 GN=SCRG_03874 PE=3 SV=1 | 100093.92 | 96539.72 | 105030.02 | 102842.87 | 96701.68 | 101735.18 |
| Q756H2 | 13 | 2 | 119.13 | 0.00000 | Enolase OS=Eremothecium gossypii (strain ATCC 10895 / CBS 109.51 / FGSC 9923 / NRRL Y-1056) OX=284811 GN=ENO PE=3 SV=1 | 592.39 | 834.91 | 926.29 | 6472.36 | 8517.57 | 9536.31 |
| G2WF10 | 3 | 2 | 13.46 | 0.00001 | K7_Ygr287c-1p OS=Saccharomyces cerevisiae (strain Kyokai no. 7 / NBRC 101557) OX=721032 GN=K7_YGR287C-1 PE=4 SV=1 | 160133.62 | 182740.26 | 188923.41 | 95580.95 | 94910.76 | 96742.01 |
| B5VEE6 | 3 | 2 | 23.97 | 0.00012 | Uncharacterized protein OS=Saccharomyces cerevisiae (strain AWRI1631) OX=545124 GN=AWRI1631_22720 PE=4 SV=1 | 105975.57 | 102388.09 | 94379.59 | 25499.40 | 28623.49 | 41330.43 |
| A0A6C1DNN1 | 2 | 2 | 15.32 | 0.00000 | FG-nucleoporin asm4 OS=Saccharomyces pastorianus OX=27292 GN=ASM4_1 PE=4 SV=1 | 286.72 | 271.71 | 241.68 | 3420.73 | 3816.66 | 4699.41 |
| C7GXL0 | 2 | 2 | 9.91 | 0.00001 | YLR407W-like protein OS=Saccharomyces cerevisiae (strain JAY291) OX=574961 GN=C1Q_05244 PE=4 SV=1 | 48856.64 | 48973.31 | 45917.48 | 17855.19 | 22658.66 | 17575.14 |
| A0A6C1E1V5 | 2 | 2 | 8.91 | 0.00001 | Oligo-1,6-glucosidase ima2 OS=Saccharomyces pastorianus OX=27292 GN=IMA2_4 PE=4 SV=1 | 82053.30 | 76786.90 | 75050.53 | 43227.60 | 42862.44 | 44007.33 |
| H0GJS8 | 2 | 2 | 10.00 | 0.00389 | PH domain-containing protein OS=Saccharomyces cerevisiae x Saccharomyces kudriavzevii (strain VIN7) OX=1095631 GN=VIN7_3101 PE=4 SV=1 | 7292.34 | 7589.25 | 7048.19 | 5610.63 | 5231.51 | 5818.60 |
| A0A0J9XHL5 | 2 | 2 | 9.24 | 0.13381 | Similar to Saccharomyces cerevisiae YIL010W DOT5 Nuclear thiol peroxidase which functions as an alkyl-hydroperoxide reductase during post-diauxic growth OS=Geotrichum candidum OX=1173061 GN=BN980_GECA15s01209g PE=4 SV=1 | 12517.94 | 12722.39 | 12938.26 | 9860.27 | 8788.49 | 9629.61 |
| K7QCP5 | 2 | 2 | 8.08 | 0.93954 | Adenylate cyclase OS=Saccharomyces cerevisiae OX=4932 GN=CYR1 PE=3 SV=1 | 7196.07 | 9335.49 | 11823.36 | 9396.38 | 7404.17 | 8124.12 |
| A6ZT81 | 30 | 1 | 269.96 | 0.00010 | phosphopyruvate hydratase OS=Saccharomyces cerevisiae (strain YJM789) OX=307796 GN=ENO2 PE=3 SV=1 | 21332.54 | 20931.72 | 22885.35 | 46348.75 | 54363.76 | 47603.13 |
| C8ZBG2 | 5 | 1 | 40.43 | 0.00000 | Glyceraldehyde-3-phosphate dehydrogenase OS=Saccharomyces cerevisiae (strain Lalvin EC1118 / Prise de mousse) OX=643680 GN=EC1118_1J11_2025g PE=3 SV=2 | 7873.20 | 7345.91 | 9568.40 | 3978.91 | 3861.52 | 3976.79 |
| Q757I2 | 5 | 1 | 55.89 | 0.00204 | Glyceraldehyde-3-phosphate dehydrogenase OS=Eremothecium gossypii (strain ATCC 10895 / CBS 109.51 / FGSC 9923 / NRRL Y-1056) OX=284811 GN=GPD PE=3 SV=2 | 6650.76 | 6284.00 | 10159.73 | 13322.75 | 9991.35 | 11126.72 |
| Q9P4C2 | 4 | 1 | 21.68 | 0.95830 | Alcohol dehydrogenase 2 OS=Kluyveromyces marxianus OX=4911 GN=ADH2 PE=3 SV=3 | 1552.25 | 1408.03 | 1720.06 | 1549.04 | 1705.03 | 1730.82 |
| N1P295 | 2 | 1 | 10.60 | 0.00000 | PX domain-containing protein OS=Saccharomyces cerevisiae (strain CEN.PK113-7D) OX=889517 GN=CENPK1137D_1792 PE=4 SV=1 | 4435.12 | 6507.21 | 5574.74 | 71441.98 | 77526.38 | 79137.92 |
| P47098 | 2 | 1 | 12.00 | 0.00000 | Transposon Ty1-JR1 Gag-Pol polyprotein OS=Saccharomyces cerevisiae (strain ATCC 204508 / S288c) OX=559292 GN=TY1B-JR1 PE=3 SV=3 | 47209.75 | 53228.69 | 44564.55 | 24726.46 | 23873.14 | 24331.06 |
| V9H070 | 2 | 1 | 8.78 | 0.19668 | LAP16 OS=Saccharomyces cerevisiae OX=4932 GN=LAP16 PE=4 SV=1 | 958.34 | 930.15 | 837.45 | 566.75 | 488.12 | 510.10 |
| A0A0J9XCR9 | 1 | 1 | 4.44 | 0.00000 | Similar to Saccharomyces cerevisiae YPR200C ARR2 Arsenate reductase required for arsenate resistance OS=Geotrichum candidum OX=1173061 GN=BN980_GECA10s02672g PE=4 SV=1 | 983.35 | 681.42 | 852.79 | 10999.20 | 13023.91 | 11298.88 |
| A0A1X7R2W5 | 1 | 1 | 4.55 | 0.00000 | Similar to Saccharomyces cerevisiae YNL188W KAR1 Essential protein involved in karyogamy during mating and in spindle pole body duplication during mitosis OS=Kazachstania saulgeensis OX=1789683 GN=KASA_0O03872G PE=4 SV=1 | 176.17 | 96.40 | 117.58 | 1288.50 | 1855.53 | 1932.32 |
| A0A6C1DY25 | 1 | 1 | 4.57 | 0.00000 | Respiration factor rsf1 OS=Saccharomyces pastorianus OX=27292 GN=RSF1_1 PE=4 SV=1 | 2937.88 | 2901.47 | 2476.60 | 590.30 | 597.18 | 426.55 |
| C8Z4I8 | 1 | 1 | 5.15 | 0.00001 | EC1118_1C17_1915p OS=Saccharomyces cerevisiae (strain Lalvin EC1118 / Prise de mousse) OX=643680 GN=EC1118_1C17_1915g PE=4 SV=1 | 80521.57 | 81287.92 | 85230.16 | 55536.29 | 55446.26 | 56157.85 |
| A0A6C1E6V2 | 1 | 1 | 4.88 | 0.03210 | Alpha-glucosidase mal32 OS=Saccharomyces pastorianus OX=27292 GN=MAL32_2 PE=4 SV=1 | 7999.86 | 7232.00 | 13381.26 | 7150.99 | 6406.41 | 6911.11 |
| A0A6C1E8Z4 | 1 | 1 | 5.12 | 0.12672 | Zinc-and pH-regulated surface protein OS=Saccharomyces pastorianus OX=27292 GN=ZPS1_4 PE=4 SV=1 | 10442.07 | 12917.14 | 13657.61 | 10514.31 | 11783.13 | 11071.37 |
| A0A061B161 | 1 | 1 | 4.77 | 0.44748 | CYFA0S07e01354g1_1 OS=Cyberlindnera fabianii OX=36022 GN=BON22_3653 PE=3 SV=1 | 1240.25 | 1276.07 | 481.19 | 624.84 | 646.57 | 401.82 |

**Table S3. Identified RNAs in EV@Y24 and EV@Y72**

| **Type** | **Locus** | **logFC** | **logCPM** | **LR** | **PValue** | **EV@Y24** | | **EV@Y72** | |
| --- | --- | --- | --- | --- | --- | --- | --- | --- | --- |
|  |  |  |  |  |  | **1** | **2** | **1** | **2** |
| miRNA | NC_001142.9..120731..120807 | 0.730 | 10.745 | 2.168 | 0.141 | 870.00 | 1472.76 | 1682.76 | 2197.01 |
| miRNA | NC_001134.8..649089..649174 | 1.033 | 8.671 | 1.328 | 0.249 | 158.18 | 163.64 | 400.66 | 283.49 |
| miRNA | NC_001224.1..49091..49129 | 0.417 | 11.020 | 0.681 | 0.409 | 2214.53 | 1063.66 | 2564.20 | 1842.66 |
| miRNA | NC_001144.5..313299..313358 | 0.480 | 7.984 | 0.114 | 0.735 | 158.18 | 0.00 | 240.39 | 0.00 |
| miRNA | NC_001224.1..47721..47759 | 0.115 | 9.360 | 0.030 | 0.861 | 553.63 | 409.10 | 320.53 | 708.71 |
| siRNA18 | NC_001139.9..878734..878853 | -2.976 | 10.844 | 28.294 | 0.000 | 2768.17 | 3272.79 | 320.53 | 425.23 |
| siRNA18 | NC_001145.3..419615..419732 | 5.681 | 8.662 | 14.584 | 0.000 | 0.00 | 0.00 | 480.79 | 496.10 |
| siRNA18 | NC_001143.9..302880..302997 | 5.570 | 8.593 | 13.383 | 0.000 | 0.00 | 0.00 | 480.79 | 425.23 |
| siRNA18 | NC_001145.3..320159..320276 | 5.576 | 8.596 | 13.161 | 0.000 | 0.00 | 0.00 | 560.92 | 354.36 |
| siRNA18 | NC_001139.9..287376..287493 | 5.451 | 8.522 | 12.066 | 0.001 | 0.00 | 0.00 | 480.79 | 354.36 |
| siRNA18 | NC_001136.10..450346..450465 | -2.013 | 9.851 | 11.967 | 0.001 | 1265.45 | 1227.30 | 400.66 | 212.61 |
| siRNA18 | NC_001145.3..379329..379446 | 3.608 | 8.908 | 11.616 | 0.001 | 79.09 | 0.00 | 480.79 | 708.71 |
| siRNA18 | NC_001134.8..88209..88327 | -3.602 | 8.814 | 11.320 | 0.001 | 474.54 | 654.56 | 0.00 | 70.87 |
| siRNA18 | NC_001137.3..86582..86699 | 5.407 | 8.506 | 9.987 | 0.002 | 0.00 | 0.00 | 80.13 | 708.71 |
| siRNA18 | NC_001134.8..348486..348604 | 5.287 | 8.433 | 9.825 | 0.002 | 0.00 | 0.00 | 160.26 | 566.97 |
| siRNA18 | NC_001135.5..146190..146307 | 5.356 | 8.455 | 9.302 | 0.002 | 0.00 | 0.00 | 721.18 | 70.87 |
| siRNA18 | NC_001141.2..49056..49174 | 2.862 | 9.032 | 9.236 | 0.002 | 79.09 | 81.82 | 881.45 | 425.23 |
| siRNA18 | NC_001139.9..998912..999024 | -5.087 | 8.286 | 8.910 | 0.003 | 474.54 | 163.64 | 0.00 | 0.00 |
| siRNA18 | NC_001148.4..56195..56312 | 4.991 | 8.271 | 8.548 | 0.003 | 0.00 | 0.00 | 240.39 | 354.36 |
| siRNA18 | NC_001134.8..774617..774734 | -2.747 | 8.879 | 8.265 | 0.004 | 474.54 | 654.56 | 160.26 | 0.00 |
| siRNA18 | NC_001136.10..452861..452973 | 4.980 | 8.268 | 8.158 | 0.004 | 0.00 | 0.00 | 160.26 | 425.23 |
| siRNA18 | NC_001148.4..35639..35756 | 4.831 | 8.186 | 7.376 | 0.007 | 0.00 | 0.00 | 400.66 | 141.74 |
| siRNA18 | NC_001140.6..421173..421291 | 2.939 | 8.515 | 6.021 | 0.014 | 79.09 | 0.00 | 240.39 | 496.10 |
| siRNA18 | NC_001147.6..838048..838166 | -1.808 | 9.199 | 5.829 | 0.016 | 553.63 | 818.20 | 240.39 | 141.74 |
| siRNA18 | NC_001136.10..1205569..1205687 | -1.196 | 10.440 | 5.783 | 0.016 | 1581.81 | 1881.85 | 641.05 | 850.46 |
| siRNA18 | NC_001140.6..124764..124880 | 2.493 | 8.806 | 5.778 | 0.016 | 79.09 | 81.82 | 801.31 | 212.61 |
| siRNA18 | NC_001142.9..663823..664008 | 1.414 | 9.701 | 5.676 | 0.017 | 316.36 | 409.10 | 1121.84 | 850.46 |
| siRNA18 | NC_001146.8..276709..276826 | -2.987 | 8.458 | 5.642 | 0.018 | 158.18 | 572.74 | 0.00 | 70.87 |
| siRNA18 | NC_001135.5..90894..91020 | 1.777 | 9.265 | 5.318 | 0.021 | 395.45 | 0.00 | 560.92 | 850.46 |
| siRNA18 | NC_001145.3..677196..677313 | -2.803 | 8.377 | 4.986 | 0.026 | 237.27 | 409.10 | 80.13 | 0.00 |
| siRNA18 | NC_001147.6..533261..533379 | 2.227 | 8.661 | 4.836 | 0.028 | 158.18 | 0.00 | 320.53 | 496.10 |
| siRNA18 | NC_001136.10..887312..887434 | -1.628 | 9.097 | 4.486 | 0.034 | 632.72 | 572.74 | 240.39 | 141.74 |
| siRNA18 | NC_001137.3..144758..144875 | 2.637 | 8.363 | 4.410 | 0.036 | 0.00 | 81.82 | 320.53 | 283.49 |
| siRNA18 | NC_001145.3..93778..93962 | 1.874 | 9.090 | 4.334 | 0.037 | 316.36 | 0.00 | 961.58 | 283.49 |
| siRNA18 | NC_001224.1..33322..33440 | 2.646 | 8.366 | 4.328 | 0.037 | 0.00 | 81.82 | 400.66 | 212.61 |
| siRNA18 | NC_001144.5..628418..628545 | -2.135 | 10.438 | 4.225 | 0.040 | 474.54 | 3600.07 | 560.92 | 354.36 |
| siRNA18 | NC_001139.9..255885..256002 | -2.126 | 8.533 | 4.053 | 0.044 | 474.54 | 245.46 | 80.13 | 70.87 |
| siRNA18 | NC_001139.9..830221..830341 | 2.087 | 8.591 | 4.028 | 0.045 | 79.09 | 81.82 | 240.39 | 496.10 |
| siRNA18 | NC_001140.6..207702..207820 | 1.835 | 8.804 | 3.844 | 0.050 | 79.09 | 163.64 | 641.05 | 283.49 |
| siRNA18 | NC_001137.3..427275..427428 | 1.230 | 9.594 | 3.837 | 0.050 | 158.18 | 572.74 | 801.31 | 921.33 |
| siRNA18 | NC_001139.9..1009412..1009527 | 2.609 | 8.353 | 3.754 | 0.053 | 79.09 | 0.00 | 80.13 | 496.10 |
| siRNA18 | NC_001139.9..13554..13671 | 2.603 | 8.353 | 3.737 | 0.053 | 0.00 | 81.82 | 80.13 | 496.10 |
| siRNA18 | NC_001136.10..845871..845987 | 2.460 | 8.277 | 3.602 | 0.058 | 79.09 | 0.00 | 320.53 | 212.61 |
| siRNA18 | NC_001224.1..28217..28426 | 1.521 | 9.121 | 3.569 | 0.059 | 237.27 | 163.64 | 320.53 | 850.46 |
| siRNA18 | NC_001144.5..419474..419591 | -2.120 | 8.532 | 3.450 | 0.063 | 632.72 | 81.82 | 80.13 | 70.87 |
| siRNA18 | NC_001144.5..570104..570224 | -1.763 | 8.675 | 3.371 | 0.066 | 316.36 | 490.92 | 0.00 | 212.61 |
| siRNA18 | NC_001147.6..103447..103564 | 1.694 | 8.730 | 3.353 | 0.067 | 158.18 | 81.82 | 320.53 | 496.10 |
| siRNA18 | NC_001136.10..254577..254695 | 1.958 | 8.524 | 3.351 | 0.067 | 158.18 | 0.00 | 400.66 | 283.49 |
| siRNA18 | NC_001224.1..31012..31112 | -2.412 | 8.189 | 3.287 | 0.070 | 316.36 | 163.64 | 0.00 | 70.87 |
| siRNA18 | NC_001146.8..443041..443159 | 1.702 | 8.732 | 3.265 | 0.071 | 237.27 | 0.00 | 400.66 | 425.23 |
| siRNA18 | NC_001136.10..296029..296146 | 2.482 | 8.284 | 3.204 | 0.073 | 0.00 | 81.82 | 480.79 | 70.87 |
| siRNA18 | NC_001139.9..952697..952814 | -1.978 | 8.453 | 3.181 | 0.075 | 237.27 | 409.10 | 0.00 | 141.74 |
| siRNA18 | NC_001140.6..254943..255060 | 1.932 | 8.516 | 3.117 | 0.077 | 79.09 | 81.82 | 160.26 | 496.10 |
| siRNA18 | NC_001144.5..187629..187747 | -1.950 | 8.458 | 3.092 | 0.079 | 395.45 | 245.46 | 160.26 | 0.00 |
| siRNA18 | NC_001134.8..526376..526495 | -1.502 | 8.807 | 2.995 | 0.084 | 474.54 | 409.10 | 80.13 | 212.61 |
| siRNA18 | NC_001224.1..27876..28010 | -0.977 | 9.777 | 2.838 | 0.092 | 711.81 | 1227.30 | 320.53 | 637.84 |
| siRNA18 | NC_001147.6..825956..826074 | 1.431 | 8.857 | 2.837 | 0.092 | 158.18 | 163.64 | 400.66 | 496.10 |
| siRNA18 | NC_001136.10..1008265..1008388 | 0.896 | 9.920 | 2.836 | 0.092 | 632.72 | 490.92 | 1201.97 | 921.33 |
| siRNA18 | NC_001145.3..447214..447333 | -1.491 | 8.814 | 2.823 | 0.093 | 316.36 | 572.74 | 240.39 | 70.87 |
| siRNA18 | NC_001147.6..992376..992494 | 1.356 | 9.031 | 2.816 | 0.093 | 316.36 | 81.82 | 641.05 | 425.23 |
| siRNA18 | NC_001139.9..369569..369687 | 1.039 | 9.487 | 2.741 | 0.098 | 316.36 | 409.10 | 881.45 | 637.84 |
| siRNA18 | NC_001147.6..815147..815264 | 1.517 | 8.907 | 2.674 | 0.102 | 79.09 | 245.46 | 160.26 | 779.58 |
| siRNA18 | NC_001144.5..565029..565145 | -2.405 | 8.196 | 2.655 | 0.103 | 0.00 | 490.92 | 80.13 | 0.00 |
| siRNA18 | NC_001139.9..715700..715818 | 1.779 | 8.443 | 2.641 | 0.104 | 79.09 | 81.82 | 240.39 | 354.36 |
| siRNA18 | NC_001139.9..423127..423245 | 1.577 | 8.670 | 2.639 | 0.104 | 237.27 | 0.00 | 480.79 | 283.49 |
| siRNA18 | NC_001145.3..67399..67511 | 2.211 | 8.177 | 2.546 | 0.111 | 0.00 | 81.82 | 80.13 | 354.36 |
| siRNA18 | NC_001136.10..371725..371899 | 1.187 | 9.130 | 2.517 | 0.113 | 158.18 | 327.28 | 480.79 | 637.84 |
| siRNA18 | NC_001145.3..808232..808349 | 1.421 | 8.851 | 2.504 | 0.114 | 237.27 | 81.82 | 240.39 | 637.84 |
| siRNA18 | NC_001135.5..132184..132381 | 1.059 | 9.368 | 2.484 | 0.115 | 316.36 | 327.28 | 881.45 | 496.10 |
| siRNA18 | NC_001136.10..1373450..1373553 | -2.145 | 8.093 | 2.483 | 0.115 | 237.27 | 163.64 | 80.13 | 0.00 |
| siRNA18 | NC_001144.5..605997..606118 | 1.548 | 8.659 | 2.459 | 0.117 | 158.18 | 81.82 | 160.26 | 566.97 |
| siRNA18 | NC_001144.5..505670..505878 | -1.074 | 9.288 | 2.448 | 0.118 | 474.54 | 818.20 | 320.53 | 283.49 |
| siRNA18 | NC_001144.5..265228..265347 | -1.151 | 9.200 | 2.371 | 0.124 | 632.72 | 572.74 | 480.79 | 70.87 |
| siRNA18 | NC_001139.9..739506..739704 | -0.888 | 9.644 | 2.355 | 0.125 | 949.09 | 736.38 | 480.79 | 425.23 |
| siRNA18 | NC_001146.8..401829..401946 | -1.762 | 8.375 | 2.343 | 0.126 | 316.36 | 245.46 | 160.26 | 0.00 |
| siRNA18 | NC_001134.8..159955..160074 | 0.716 | 13.445 | 2.336 | 0.126 | 6089.97 | 10554.75 | 13301.81 | 14032.53 |
| siRNA18 | NC_001224.1..78857..78987 | 0.837 | 9.801 | 2.273 | 0.132 | 395.45 | 654.56 | 1041.71 | 850.46 |
| siRNA18 | NC_001137.3..107264..107378 | 1.309 | 8.799 | 2.243 | 0.134 | 79.09 | 245.46 | 400.66 | 425.23 |
| siRNA18 | NC_001148.4..391762..391880 | 1.306 | 8.795 | 2.241 | 0.134 | 158.18 | 163.64 | 320.53 | 496.10 |
| siRNA18 | NC_001145.3..786016..786133 | 0.907 | 9.742 | 2.207 | 0.137 | 632.72 | 327.28 | 1282.10 | 566.97 |
| siRNA18 | NC_001139.9..384736..384854 | 1.417 | 8.596 | 2.193 | 0.139 | 158.18 | 81.82 | 320.53 | 354.36 |
| siRNA18 | NC_001137.3..126615..126732 | 1.329 | 8.807 | 2.127 | 0.145 | 158.18 | 163.64 | 641.05 | 212.61 |
| siRNA18 | NC_001134.8..318751..318868 | -1.194 | 8.869 | 2.111 | 0.146 | 474.54 | 409.10 | 160.26 | 212.61 |
| siRNA18 | NC_001145.3..164775..164892 | -1.431 | 8.534 | 2.106 | 0.147 | 316.36 | 327.28 | 160.26 | 70.87 |
| siRNA18 | NC_001147.6..364480..364596 | 1.984 | 8.082 | 2.096 | 0.148 | 79.09 | 0.00 | 160.26 | 212.61 |
| siRNA18 | NC_001145.3..693859..693969 | -2.152 | 8.095 | 2.090 | 0.148 | 0.00 | 409.10 | 80.13 | 0.00 |
| siRNA18 | NC_001148.4..525800..525917 | 1.980 | 8.083 | 2.086 | 0.149 | 0.00 | 81.82 | 160.26 | 212.61 |
| siRNA18 | NC_001136.10..1038498..1038615 | -1.307 | 8.937 | 2.056 | 0.152 | 158.18 | 818.20 | 320.53 | 70.87 |
| siRNA18 | NC_001136.10..100336..100455 | -0.747 | 11.831 | 2.025 | 0.155 | 4429.07 | 4336.45 | 1282.10 | 3897.92 |
| siRNA18 | NC_001136.10..451456..451573 | 2.011 | 8.089 | 2.009 | 0.156 | 79.09 | 0.00 | 320.53 | 70.87 |
| siRNA18 | NC_001134.8..304496..304614 | 0.779 | 9.767 | 1.963 | 0.161 | 632.72 | 409.10 | 961.58 | 850.46 |
| siRNA18 | NC_001145.3..770897..771017 | -1.183 | 8.876 | 1.897 | 0.168 | 316.36 | 572.74 | 320.53 | 70.87 |
| siRNA18 | NC_001141.2..159555..159670 | -1.065 | 8.987 | 1.858 | 0.173 | 395.45 | 572.74 | 240.39 | 212.61 |
| siRNA18 | NC_001142.9..253360..253482 | 0.985 | 9.188 | 1.790 | 0.181 | 158.18 | 409.10 | 721.18 | 425.23 |
| siRNA18 | NC_001139.9..929777..929878 | -1.576 | 8.281 | 1.772 | 0.183 | 237.27 | 245.46 | 0.00 | 141.74 |
| siRNA18 | NC_001145.3..762439..762549 | -1.581 | 8.282 | 1.727 | 0.189 | 158.18 | 327.28 | 0.00 | 141.74 |
| siRNA18 | NC_001146.8..406839..406952 | -1.581 | 8.282 | 1.727 | 0.189 | 158.18 | 327.28 | 0.00 | 141.74 |
| siRNA18 | NC_001140.6..328362..328485 | -1.209 | 8.681 | 1.709 | 0.191 | 237.27 | 490.92 | 240.39 | 70.87 |
| siRNA18 | NC_001148.4..592460..592578 | 0.987 | 9.027 | 1.611 | 0.204 | 316.36 | 163.64 | 400.66 | 566.97 |
| siRNA18 | NC_001141.2..277132..277430 | 0.757 | 9.564 | 1.590 | 0.207 | 395.45 | 490.92 | 881.45 | 637.84 |
| siRNA18 | NC_001141.2..214221..214351 | -1.059 | 8.806 | 1.576 | 0.209 | 474.54 | 327.28 | 160.26 | 212.61 |
| siRNA18 | NC_001136.10..197652..197769 | -1.205 | 8.675 | 1.506 | 0.220 | 632.72 | 81.82 | 160.26 | 141.74 |
| siRNA18 | NC_001139.9..477795..477902 | 1.267 | 8.527 | 1.489 | 0.222 | 237.27 | 0.00 | 400.66 | 212.61 |
| siRNA18 | NC_001134.8..309551..309671 | -1.057 | 8.811 | 1.478 | 0.224 | 237.27 | 572.74 | 240.39 | 141.74 |
| siRNA18 | NC_001147.6..449212..449329 | -1.252 | 8.452 | 1.476 | 0.224 | 316.36 | 245.46 | 80.13 | 141.74 |
| siRNA18 | NC_001148.4..331479..331598 | 0.571 | 11.177 | 1.383 | 0.240 | 1344.54 | 2127.31 | 2243.68 | 2905.73 |
| siRNA18 | NC_001144.5..136515..136634 | 1.376 | 8.274 | 1.373 | 0.241 | 79.09 | 81.82 | 160.26 | 283.49 |
| siRNA18 | NC_001146.8..18404..18521 | 1.376 | 8.274 | 1.373 | 0.241 | 79.09 | 81.82 | 160.26 | 283.49 |
| siRNA18 | NC_001146.8..24174..24293 | 0.995 | 9.038 | 1.358 | 0.244 | 0.00 | 490.92 | 641.05 | 354.36 |
| siRNA18 | NC_001143.9..316908..317027 | 1.028 | 8.668 | 1.328 | 0.249 | 158.18 | 163.64 | 320.53 | 354.36 |
| siRNA18 | NC_001145.3..171094..171208 | -1.231 | 8.459 | 1.319 | 0.251 | 237.27 | 327.28 | 240.39 | 0.00 |
| siRNA18 | NC_001142.9..94170..94287 | 0.640 | 10.092 | 1.297 | 0.255 | 316.36 | 1145.48 | 1442.37 | 850.46 |
| siRNA18 | NC_001137.3..131066..131184 | 0.562 | 9.975 | 1.217 | 0.270 | 632.72 | 736.38 | 961.58 | 1063.07 |
| siRNA18 | NC_001144.5..612858..612971 | -1.312 | 8.191 | 1.190 | 0.275 | 158.18 | 245.46 | 80.13 | 70.87 |
| siRNA18 | NC_001147.6..903091..903208 | -1.309 | 8.190 | 1.188 | 0.276 | 237.27 | 163.64 | 80.13 | 70.87 |
| siRNA18 | NC_001134.8..689314..689431 | -0.641 | 9.602 | 1.175 | 0.278 | 711.81 | 818.20 | 320.53 | 637.84 |
| siRNA18 | NC_001148.4..839567..839691 | -0.773 | 9.146 | 1.151 | 0.283 | 474.54 | 572.74 | 400.66 | 212.61 |
| siRNA18 | NC_001224.1..7317..7434 | 1.011 | 8.663 | 1.140 | 0.286 | 79.09 | 245.46 | 160.26 | 496.10 |
| siRNA18 | NC_001146.8..443394..443561 | -0.785 | 9.142 | 1.118 | 0.290 | 316.36 | 736.38 | 240.39 | 354.36 |
| siRNA18 | NC_001147.6..574745..574855 | -1.334 | 8.188 | 1.097 | 0.295 | 79.09 | 327.28 | 0.00 | 141.74 |
| siRNA18 | NC_001146.8..204586..204687 | -1.293 | 8.193 | 1.095 | 0.295 | 237.27 | 163.64 | 160.26 | 0.00 |
| siRNA18 | NC_001141.2..259280..259390 | -1.323 | 8.186 | 1.084 | 0.298 | 316.36 | 81.82 | 0.00 | 141.74 |
| siRNA18 | NC_001145.3..542441..542559 | -0.658 | 9.541 | 1.073 | 0.300 | 632.72 | 818.20 | 721.18 | 212.61 |
| siRNA18 | NC_001141.2..248421..248533 | 1.344 | 8.268 | 1.069 | 0.301 | 79.09 | 81.82 | 0.00 | 425.23 |
| siRNA18 | NC_001142.9..255992..256110 | 1.065 | 8.446 | 1.058 | 0.304 | 158.18 | 81.82 | 240.39 | 283.49 |
| siRNA18 | NC_001148.4..908151..908269 | -0.513 | 11.035 | 1.039 | 0.308 | 2609.99 | 1963.67 | 2163.55 | 1063.07 |
| siRNA18 | NC_001224.1..36489..36594 | -0.899 | 8.748 | 1.036 | 0.309 | 316.36 | 409.10 | 320.53 | 70.87 |
| siRNA18 | NC_001144.5..940711..940828 | 1.005 | 8.658 | 1.033 | 0.310 | 237.27 | 81.82 | 80.13 | 566.97 |
| siRNA18 | NC_001224.1..77620..77737 | -1.231 | 8.462 | 1.012 | 0.314 | 0.00 | 572.74 | 240.39 | 0.00 |
| siRNA18 | NC_001139.9..827390..827507 | -0.917 | 8.737 | 0.999 | 0.318 | 553.63 | 163.64 | 80.13 | 283.49 |
| siRNA18 | NC_001134.8..75115..75259 | 0.602 | 9.484 | 0.981 | 0.322 | 474.54 | 409.10 | 641.05 | 708.71 |
| siRNA18 | NC_001136.10..730223..730340 | 0.606 | 9.486 | 0.972 | 0.324 | 553.63 | 327.28 | 721.18 | 637.84 |
| siRNA18 | NC_001137.3..335668..335786 | 0.701 | 9.186 | 0.972 | 0.324 | 395.45 | 245.46 | 641.05 | 425.23 |
| siRNA18 | NC_001133.9..87265..87399 | 0.469 | 10.683 | 0.966 | 0.326 | 1344.54 | 1145.48 | 1682.76 | 1771.78 |
| siRNA18 | NC_001136.10..754983..755104 | -0.819 | 8.864 | 0.964 | 0.326 | 395.45 | 409.10 | 80.13 | 354.36 |
| siRNA18 | NC_001224.1..61866..62087 | 0.459 | 11.541 | 0.933 | 0.334 | 2135.44 | 2618.23 | 4006.57 | 2551.37 |
| siRNA18 | NC_001145.3..720571..720689 | 0.510 | 11.639 | 0.914 | 0.339 | 2214.53 | 2781.87 | 1843.02 | 5244.48 |
| siRNA18 | NC_001139.9..373605..373722 | -1.042 | 8.370 | 0.913 | 0.339 | 158.18 | 327.28 | 80.13 | 141.74 |
| siRNA18 | NC_001134.8..680744..680857 | 1.140 | 8.187 | 0.888 | 0.346 | 79.09 | 81.82 | 240.39 | 141.74 |
| siRNA18 | NC_001144.5..983054..983161 | 1.140 | 8.187 | 0.888 | 0.346 | 79.09 | 81.82 | 240.39 | 141.74 |
| siRNA18 | NC_001136.10..1008914..1009029 | 1.130 | 8.183 | 0.879 | 0.349 | 79.09 | 81.82 | 160.26 | 212.61 |
| siRNA18 | NC_001137.3..74534..74651 | -1.058 | 8.367 | 0.870 | 0.351 | 158.18 | 327.28 | 0.00 | 212.61 |
| siRNA18 | NC_001133.9..190425..190571 | -0.745 | 9.368 | 0.852 | 0.356 | 1186.36 | 81.82 | 400.66 | 354.36 |
| siRNA18 | NC_001140.6..476876..476996 | 0.867 | 13.040 | 0.850 | 0.356 | 2135.44 | 9572.91 | 11939.58 | 9425.89 |
| siRNA18 | NC_001144.5..254429..254616 | -0.442 | 10.777 | 0.848 | 0.357 | 2135.44 | 1554.58 | 1442.37 | 1275.68 |
| siRNA18 | NC_001145.3..15907..16097 | 0.455 | 10.181 | 0.844 | 0.358 | 632.72 | 1063.66 | 1041.71 | 1275.68 |
| siRNA18 | NC_001136.10..868319..868439 | 0.881 | 8.606 | 0.834 | 0.361 | 158.18 | 163.64 | 480.79 | 141.74 |
| siRNA18 | NC_001136.10..1508372..1508490 | -0.871 | 8.529 | 0.809 | 0.368 | 237.27 | 327.28 | 80.13 | 212.61 |
| siRNA18 | NC_001139.9..89205..89322 | -0.797 | 8.866 | 0.782 | 0.377 | 711.81 | 81.82 | 240.39 | 212.61 |
| siRNA18 | NC_001140.6..104841..105028 | -0.419 | 11.116 | 0.750 | 0.387 | 2926.35 | 1800.04 | 1762.89 | 1771.78 |
| siRNA18 | NC_001144.5..1033551..1033663 | 1.186 | 8.192 | 0.742 | 0.389 | 158.18 | 0.00 | 400.66 | 0.00 |
| siRNA18 | NC_001146.8..273347..273578 | 0.463 | 10.240 | 0.736 | 0.391 | 870.00 | 900.02 | 641.05 | 1771.78 |
| siRNA18 | NC_001136.10..665438..665553 | -1.015 | 8.377 | 0.734 | 0.391 | 79.09 | 409.10 | 240.39 | 0.00 |
| siRNA18 | NC_001136.10..645137..645255 | 0.434 | 10.231 | 0.727 | 0.394 | 553.63 | 1227.30 | 1121.84 | 1275.68 |
| siRNA18 | NC_001148.4..775736..775853 | -0.749 | 8.675 | 0.722 | 0.395 | 237.27 | 409.10 | 160.26 | 212.61 |
| siRNA18 | NC_001139.9..1019782..1019898 | -1.001 | 8.374 | 0.719 | 0.396 | 395.45 | 81.82 | 240.39 | 0.00 |
| siRNA18 | NC_001148.4..910345..910463 | -0.771 | 8.876 | 0.707 | 0.400 | 553.63 | 245.46 | 480.79 | 0.00 |
| siRNA18 | NC_001148.4..578932..579050 | -0.757 | 8.672 | 0.704 | 0.401 | 237.27 | 409.10 | 80.13 | 283.49 |
| siRNA18 | NC_001145.3..856423..856540 | -0.897 | 8.527 | 0.685 | 0.408 | 79.09 | 490.92 | 0.00 | 283.49 |
| siRNA18 | NC_001134.8..350796..350924 | 0.402 | 10.877 | 0.666 | 0.414 | 1898.17 | 1063.66 | 2163.55 | 1771.78 |
| siRNA18 | NC_001137.3..288395..288511 | 1.087 | 8.178 | 0.645 | 0.422 | 0.00 | 163.64 | 0.00 | 354.36 |
| siRNA18 | NC_001139.9..740714..740831 | 0.720 | 8.910 | 0.641 | 0.423 | 158.18 | 327.28 | 80.13 | 708.71 |
| siRNA18 | NC_001147.6..594358..594475 | -0.657 | 8.807 | 0.635 | 0.426 | 395.45 | 327.28 | 240.39 | 212.61 |
| siRNA18 | NC_001143.9..458592..458710 | 0.736 | 8.737 | 0.631 | 0.427 | 395.45 | 0.00 | 400.66 | 283.49 |
| siRNA18 | NC_001139.9..707079..707196 | -0.662 | 8.803 | 0.614 | 0.433 | 474.54 | 245.46 | 160.26 | 283.49 |
| siRNA18 | NC_001138.5..211603..211720 | 0.578 | 9.130 | 0.612 | 0.434 | 237.27 | 409.10 | 320.53 | 637.84 |
| siRNA18 | NC_001139.9..1057728..1057923 | -0.340 | 12.695 | 0.596 | 0.440 | 6959.96 | 7527.42 | 5769.46 | 5669.71 |
| siRNA18 | NC_001142.9..23338..23455 | 0.828 | 8.587 | 0.593 | 0.441 | 237.27 | 81.82 | 0.00 | 566.97 |
| siRNA18 | NC_001134.8..392897..393015 | -0.662 | 8.809 | 0.578 | 0.447 | 158.18 | 572.74 | 240.39 | 212.61 |
| siRNA18 | NC_001137.3..321982..322100 | -0.596 | 8.927 | 0.574 | 0.449 | 316.36 | 490.92 | 240.39 | 283.49 |
| siRNA18 | NC_001134.8..439881..439998 | -0.983 | 8.092 | 0.570 | 0.450 | 237.27 | 81.82 | 160.26 | 0.00 |
| siRNA18 | NC_001135.5..261944..262062 | -0.670 | 8.799 | 0.556 | 0.456 | 553.63 | 163.64 | 80.13 | 354.36 |
| siRNA18 | NC_001145.3..178690..178804 | -0.655 | 8.812 | 0.550 | 0.458 | 158.18 | 572.74 | 320.53 | 141.74 |
| siRNA18 | NC_001142.9..243738..243855 | 0.467 | 9.782 | 0.543 | 0.461 | 316.36 | 900.02 | 1282.10 | 425.23 |
| siRNA18 | NC_001144.5..108847..108965 | -0.645 | 8.808 | 0.540 | 0.463 | 553.63 | 163.64 | 320.53 | 141.74 |
| siRNA18 | NC_001137.3..250255..250373 | -0.360 | 10.495 | 0.536 | 0.464 | 1107.27 | 1800.04 | 1121.84 | 1133.94 |
| siRNA18 | NC_001146.8..544829..544950 | -0.380 | 9.869 | 0.524 | 0.469 | 949.09 | 818.20 | 721.18 | 637.84 |
| siRNA18 | NC_001147.6..770826..770946 | 0.683 | 8.528 | 0.506 | 0.477 | 158.18 | 163.64 | 320.53 | 212.61 |
| siRNA18 | NC_001137.3..158683..158800 | -0.703 | 8.801 | 0.498 | 0.480 | 79.09 | 654.56 | 0.00 | 425.23 |
| siRNA18 | NC_001146.8..172004..172127 | 0.406 | 9.664 | 0.494 | 0.482 | 632.72 | 490.92 | 560.92 | 921.33 |
| siRNA18 | NC_001147.6..39112..39228 | -0.786 | 8.280 | 0.493 | 0.482 | 237.27 | 163.64 | 80.13 | 141.74 |
| siRNA18 | NC_001134.8..760768..760887 | 0.678 | 8.524 | 0.488 | 0.485 | 237.27 | 81.82 | 240.39 | 283.49 |
| siRNA18 | NC_001147.6..281273..281391 | 0.681 | 8.529 | 0.487 | 0.485 | 79.09 | 245.46 | 320.53 | 212.61 |
| siRNA18 | NC_001140.6..57959..58064 | 0.674 | 8.526 | 0.481 | 0.488 | 79.09 | 245.46 | 240.39 | 283.49 |
| siRNA18 | NC_001146.8..386057..386174 | 0.674 | 8.526 | 0.481 | 0.488 | 79.09 | 245.46 | 240.39 | 283.49 |
| siRNA18 | NC_001224.1..75832..75950 | 0.691 | 8.526 | 0.458 | 0.498 | 316.36 | 0.00 | 320.53 | 212.61 |
| siRNA18 | NC_001134.8..581787..581909 | -0.460 | 9.240 | 0.458 | 0.499 | 553.63 | 490.92 | 480.79 | 283.49 |
| siRNA18 | NC_001143.9..382763..382916 | 0.301 | 13.520 | 0.455 | 0.500 | 9807.22 | 10963.85 | 12901.15 | 12685.97 |
| siRNA18 | NC_001145.3..288106..288217 | 0.665 | 8.523 | 0.452 | 0.501 | 79.09 | 245.46 | 160.26 | 354.36 |
| siRNA18 | NC_001144.5..735041..735172 | 0.336 | 10.178 | 0.438 | 0.508 | 711.81 | 1063.66 | 801.31 | 1417.43 |
| siRNA18 | NC_001134.8..633525..633645 | 0.404 | 9.488 | 0.427 | 0.514 | 632.72 | 327.28 | 721.18 | 566.97 |
| siRNA18 | NC_001148.4..869443..869555 | 0.835 | 8.085 | 0.424 | 0.515 | 158.18 | 0.00 | 160.26 | 141.74 |
| siRNA18 | NC_001139.9..251166..251275 | 0.818 | 8.083 | 0.416 | 0.519 | 79.09 | 81.82 | 80.13 | 212.61 |
| siRNA18 | NC_001143.9..292797..292914 | 0.818 | 8.083 | 0.416 | 0.519 | 79.09 | 81.82 | 80.13 | 212.61 |
| siRNA18 | NC_001134.8..130892..131006 | -0.757 | 8.287 | 0.414 | 0.520 | 237.27 | 163.64 | 240.39 | 0.00 |
| siRNA18 | NC_001136.10..1255938..1256055 | 0.296 | 11.707 | 0.414 | 0.520 | 2451.80 | 3272.79 | 3846.31 | 3189.21 |
| siRNA18 | NC_001144.5..448634..448752 | 0.314 | 10.111 | 0.414 | 0.520 | 711.81 | 981.84 | 1041.71 | 1063.07 |
| siRNA18 | NC_001136.10..848864..848978 | 0.841 | 8.090 | 0.411 | 0.522 | 0.00 | 163.64 | 240.39 | 70.87 |
| siRNA18 | NC_001136.10..349700..349817 | 0.702 | 8.536 | 0.409 | 0.522 | 0.00 | 327.28 | 480.79 | 70.87 |
| siRNA18 | NC_001144.5..780640..780760 | 0.560 | 8.671 | 0.409 | 0.523 | 158.18 | 245.46 | 320.53 | 283.49 |
| siRNA18 | NC_001136.10..965901..966018 | -0.655 | 8.449 | 0.409 | 0.523 | 316.36 | 163.64 | 80.13 | 212.61 |
| siRNA18 | NC_001147.6..1031909..1032024 | -0.655 | 8.449 | 0.409 | 0.523 | 316.36 | 163.64 | 80.13 | 212.61 |
| siRNA18 | NC_001135.5..175232..175400 | 0.325 | 10.055 | 0.406 | 0.524 | 632.72 | 981.84 | 801.31 | 1204.81 |
| siRNA18 | NC_001134.8..598118..598235 | 0.821 | 8.082 | 0.399 | 0.528 | 158.18 | 0.00 | 80.13 | 212.61 |
| siRNA18 | NC_001142.9..232724..232841 | 0.821 | 8.082 | 0.399 | 0.528 | 158.18 | 0.00 | 80.13 | 212.61 |
| siRNA18 | NC_001224.1..59564..59732 | -0.480 | 9.147 | 0.398 | 0.528 | 632.72 | 327.28 | 560.92 | 141.74 |
| siRNA18 | NC_001145.3..98337..98454 | 0.554 | 8.669 | 0.397 | 0.529 | 158.18 | 245.46 | 240.39 | 354.36 |
| siRNA18 | NC_001145.3..643533..643650 | -0.800 | 8.275 | 0.386 | 0.534 | 395.45 | 0.00 | 0.00 | 212.61 |
| siRNA18 | NC_001144.5..497876..497993 | 0.270 | 12.499 | 0.377 | 0.539 | 5219.97 | 4991.01 | 6009.85 | 6307.55 |
| siRNA18 | NC_001148.4..827957..828074 | -0.304 | 10.242 | 0.377 | 0.539 | 949.09 | 1390.94 | 1121.84 | 779.58 |
| siRNA18 | NC_001136.10..198317..198481 | 0.278 | 15.971 | 0.371 | 0.542 | 55046.96 | 60792.09 | 66829.58 | 73635.34 |
| siRNA18 | NC_001135.5..285277..285394 | -0.564 | 8.604 | 0.370 | 0.543 | 158.18 | 409.10 | 160.26 | 212.61 |
| siRNA18 | NC_001141.2..297127..297244 | 0.567 | 8.669 | 0.361 | 0.548 | 395.45 | 0.00 | 320.53 | 283.49 |
| siRNA18 | NC_001146.8..403682..403799 | -0.641 | 8.457 | 0.359 | 0.549 | 79.09 | 409.10 | 240.39 | 70.87 |
| siRNA18 | NC_001144.5..457044..457241 | 0.274 | 12.100 | 0.353 | 0.552 | 3005.44 | 4663.73 | 4086.70 | 5173.61 |
| siRNA18 | NC_001144.5..811938..812060 | -0.352 | 9.706 | 0.347 | 0.556 | 474.54 | 1063.66 | 400.66 | 779.58 |
| siRNA18 | NC_001148.4..125411..125530 | -0.588 | 8.598 | 0.346 | 0.556 | 158.18 | 409.10 | 0.00 | 354.36 |
| siRNA18 | NC_001146.8..230524..230711 | -0.501 | 8.740 | 0.341 | 0.559 | 237.27 | 409.10 | 160.26 | 283.49 |
| siRNA18 | NC_001147.6..944011..944129 | -0.476 | 9.153 | 0.335 | 0.562 | 237.27 | 736.38 | 641.05 | 70.87 |
| siRNA18 | NC_001147.6..54585..54701 | 0.636 | 8.517 | 0.334 | 0.563 | 79.09 | 245.46 | 0.00 | 496.10 |
| siRNA18 | NC_001144.5..466181..466378 | 0.263 | 12.079 | 0.333 | 0.564 | 3005.44 | 4581.91 | 4567.49 | 4535.77 |
| siRNA18 | NC_001146.8..448027..448134 | 0.789 | 8.080 | 0.331 | 0.565 | 0.00 | 163.64 | 0.00 | 283.49 |
| siRNA18 | NC_001145.3..126946..127064 | 0.494 | 8.809 | 0.310 | 0.578 | 316.36 | 163.64 | 560.92 | 141.74 |
| siRNA18 | NC_001141.2..278734..278852 | 0.467 | 8.797 | 0.305 | 0.581 | 316.36 | 163.64 | 240.39 | 425.23 |
| siRNA18 | NC_001142.9..518415..518587 | 0.244 | 13.440 | 0.291 | 0.590 | 8541.77 | 11536.59 | 12580.63 | 11197.68 |
| siRNA18 | NC_001140.6..158166..158286 | -0.460 | 8.865 | 0.288 | 0.591 | 158.18 | 572.74 | 160.26 | 354.36 |
| siRNA18 | NC_001144.5..159675..159792 | 0.369 | 9.276 | 0.269 | 0.604 | 158.18 | 654.56 | 400.66 | 637.84 |
| siRNA18 | NC_001142.9..364600..364719 | -0.403 | 8.982 | 0.267 | 0.605 | 553.63 | 245.46 | 320.53 | 283.49 |
| siRNA18 | NC_001139.9..668867..668984 | 0.645 | 8.287 | 0.266 | 0.606 | 158.18 | 81.82 | 400.66 | 0.00 |
| siRNA18 | NC_001136.10..1115494..1115615 | 0.404 | 8.919 | 0.256 | 0.613 | 395.45 | 163.64 | 320.53 | 425.23 |
| siRNA18 | NC_001142.9..698973..699188 | 0.239 | 10.687 | 0.253 | 0.615 | 1265.45 | 1472.76 | 1602.63 | 1630.04 |
| siRNA18 | NC_001139.9..520276..520423 | 0.309 | 9.444 | 0.248 | 0.619 | 553.63 | 409.10 | 480.79 | 708.71 |
| siRNA18 | NC_001144.5..454210..454485 | 0.225 | 13.255 | 0.246 | 0.620 | 7434.50 | 10309.29 | 11538.92 | 9213.28 |
| siRNA18 | NC_001139.9..107509..107632 | -0.345 | 9.192 | 0.245 | 0.621 | 553.63 | 409.10 | 480.79 | 283.49 |
| siRNA18 | NC_001136.10..504545..504666 | -0.329 | 9.280 | 0.235 | 0.628 | 711.81 | 327.28 | 400.66 | 425.23 |
| siRNA18 | NC_001144.5..456708..456974 | 0.213 | 12.767 | 0.233 | 0.629 | 6485.42 | 6136.48 | 7692.61 | 6945.39 |
| siRNA18 | NC_001147.6..960981..961099 | 0.393 | 8.919 | 0.232 | 0.630 | 158.18 | 409.10 | 240.39 | 496.10 |
| siRNA18 | NC_001139.9..176616..176770 | -0.341 | 9.195 | 0.231 | 0.631 | 474.54 | 490.92 | 560.92 | 212.61 |
| siRNA18 | NC_001138.5..90700..90820 | 0.327 | 9.355 | 0.225 | 0.635 | 474.54 | 409.10 | 240.39 | 850.46 |
| siRNA18 | NC_001224.1..40311..40484 | -0.331 | 9.287 | 0.220 | 0.639 | 237.27 | 818.20 | 480.79 | 354.36 |
| siRNA18 | NC_001134.8..393084..393205 | 0.348 | 9.030 | 0.214 | 0.644 | 316.36 | 327.28 | 320.53 | 496.10 |
| siRNA18 | NC_001147.6..171417..171527 | 0.487 | 8.455 | 0.211 | 0.646 | 158.18 | 163.64 | 400.66 | 70.87 |
| siRNA18 | NC_001136.10..714171..714284 | 0.473 | 8.453 | 0.209 | 0.648 | 79.09 | 245.46 | 320.53 | 141.74 |
| siRNA18 | NC_001144.5..812183..812300 | 0.463 | 8.450 | 0.207 | 0.649 | 79.09 | 245.46 | 240.39 | 212.61 |
| siRNA18 | NC_001141.2..422137..422459 | -0.282 | 9.535 | 0.205 | 0.651 | 395.45 | 900.02 | 641.05 | 425.23 |
| siRNA18 | NC_001144.5..325503..325622 | -0.370 | 9.086 | 0.203 | 0.653 | 790.90 | 81.82 | 320.53 | 354.36 |
| siRNA18 | NC_001146.8..580904..581021 | -0.223 | 10.034 | 0.202 | 0.653 | 949.09 | 981.84 | 721.18 | 921.33 |
| siRNA18 | NC_001134.8..406365..406482 | -0.439 | 8.969 | 0.196 | 0.658 | 711.81 | 81.82 | 0.00 | 566.97 |
| siRNA18 | NC_001224.1..36124..36355 | 0.194 | 13.422 | 0.191 | 0.662 | 9965.40 | 10227.47 | 11619.05 | 11481.16 |
| siRNA18 | NC_001144.5..374350..374468 | 0.315 | 9.141 | 0.187 | 0.665 | 237.27 | 490.92 | 560.92 | 354.36 |
| siRNA18 | NC_001144.5..749357..749563 | -0.261 | 9.733 | 0.184 | 0.668 | 1107.27 | 409.10 | 400.66 | 850.46 |
| siRNA18 | NC_001148.4..814801..814918 | -0.482 | 8.187 | 0.172 | 0.679 | 158.18 | 163.64 | 80.13 | 141.74 |
| siRNA18 | NC_001139.9..695525..695642 | 0.372 | 8.599 | 0.169 | 0.681 | 237.27 | 163.64 | 240.39 | 283.49 |
| siRNA18 | NC_001140.6..462812..462929 | 0.390 | 8.605 | 0.166 | 0.683 | 316.36 | 81.82 | 400.66 | 141.74 |
| siRNA18 | NC_001134.8..178325..178441 | -0.479 | 8.186 | 0.164 | 0.686 | 237.27 | 81.82 | 80.13 | 141.74 |
| siRNA18 | NC_001137.3..66697..66807 | -0.470 | 8.190 | 0.163 | 0.687 | 158.18 | 163.64 | 160.26 | 70.87 |
| siRNA18 | NC_001137.3..158090..158195 | -0.470 | 8.190 | 0.163 | 0.687 | 158.18 | 163.64 | 160.26 | 70.87 |
| siRNA18 | NC_001148.4..376919..377045 | 0.374 | 8.599 | 0.162 | 0.687 | 316.36 | 81.82 | 240.39 | 283.49 |
| siRNA18 | NC_001136.10..1384471..1384588 | 0.332 | 9.030 | 0.160 | 0.689 | 79.09 | 572.74 | 240.39 | 566.97 |
| siRNA18 | NC_001146.8..586093..586210 | 0.383 | 8.607 | 0.160 | 0.689 | 79.09 | 327.28 | 400.66 | 141.74 |
| siRNA18 | NC_001147.6..127824..127943 | 0.223 | 9.940 | 0.159 | 0.690 | 870.00 | 654.56 | 480.79 | 1275.68 |
| siRNA18 | NC_001134.8..642978..643095 | 0.365 | 8.596 | 0.157 | 0.692 | 237.27 | 163.64 | 160.26 | 354.36 |
| siRNA18 | NC_001137.3..532058..532175 | 0.386 | 8.601 | 0.154 | 0.695 | 395.45 | 0.00 | 320.53 | 212.61 |
| siRNA18 | NC_001146.8..252556..252679 | 0.281 | 9.774 | 0.150 | 0.698 | 79.09 | 1227.30 | 801.31 | 779.58 |
| siRNA18 | NC_001134.8..492172..492296 | 0.248 | 9.324 | 0.148 | 0.700 | 474.54 | 409.10 | 560.92 | 496.10 |
| siRNA18 | NC_001143.9..558966..559083 | 0.250 | 9.327 | 0.148 | 0.701 | 395.45 | 490.92 | 641.05 | 425.23 |
| siRNA18 | NC_001135.5..168285..168403 | 0.191 | 10.498 | 0.147 | 0.701 | 1265.45 | 1145.48 | 961.58 | 1771.78 |
| siRNA18 | NC_001144.5..463347..463598 | 0.170 | 13.368 | 0.146 | 0.702 | 10360.85 | 9245.63 | 11218.39 | 10843.32 |
| siRNA18 | NC_001133.9..138118..138236 | -0.407 | 8.366 | 0.145 | 0.704 | 158.18 | 245.46 | 80.13 | 212.61 |
| siRNA18 | NC_001145.3..405229..405356 | 0.182 | 10.579 | 0.144 | 0.704 | 1265.45 | 1309.12 | 1282.10 | 1630.04 |
| siRNA18 | NC_001136.10..399874..399992 | -0.405 | 8.366 | 0.143 | 0.705 | 237.27 | 163.64 | 80.13 | 212.61 |
| siRNA18 | NC_001136.10..520941..521059 | -0.189 | 10.366 | 0.140 | 0.708 | 1660.90 | 818.20 | 1041.71 | 1133.94 |
| siRNA18 | NC_001148.4..499096..499198 | -0.394 | 8.369 | 0.140 | 0.709 | 237.27 | 163.64 | 160.26 | 141.74 |
| siRNA18 | NC_001148.4..762685..762802 | 0.243 | 9.323 | 0.137 | 0.711 | 316.36 | 572.74 | 480.79 | 566.97 |
| siRNA18 | NC_001148.4..370993..371109 | -0.431 | 8.364 | 0.137 | 0.711 | 79.09 | 327.28 | 0.00 | 283.49 |
| siRNA18 | NC_001147.6..357414..357530 | -0.452 | 8.193 | 0.136 | 0.712 | 158.18 | 163.64 | 240.39 | 0.00 |
| siRNA18 | NC_001136.10..401553..401670 | 0.368 | 8.595 | 0.136 | 0.713 | 395.45 | 0.00 | 160.26 | 354.36 |
| siRNA18 | NC_001148.4..360498..360615 | -0.355 | 8.525 | 0.135 | 0.713 | 237.27 | 245.46 | 80.13 | 283.49 |
| siRNA18 | NC_001139.9..609629..609746 | 0.314 | 8.742 | 0.135 | 0.713 | 237.27 | 245.46 | 400.66 | 212.61 |
| siRNA18 | NC_001148.4..150171..150288 | -0.400 | 8.370 | 0.135 | 0.713 | 79.09 | 327.28 | 160.26 | 141.74 |
| siRNA18 | NC_001135.5..281828..281944 | -0.454 | 8.194 | 0.132 | 0.716 | 79.09 | 245.46 | 240.39 | 0.00 |
| siRNA18 | NC_001134.8..150101..150218 | -0.419 | 8.362 | 0.131 | 0.718 | 316.36 | 81.82 | 0.00 | 283.49 |
| siRNA18 | NC_001137.3..56896..57010 | -0.419 | 8.362 | 0.131 | 0.718 | 316.36 | 81.82 | 0.00 | 283.49 |
| siRNA18 | NC_001144.5..309388..309506 | 0.172 | 11.192 | 0.130 | 0.718 | 1819.08 | 2290.95 | 1923.15 | 2693.11 |
| siRNA18 | NC_001147.6..334585..334702 | -0.374 | 8.523 | 0.130 | 0.718 | 158.18 | 327.28 | 0.00 | 354.36 |
| siRNA18 | NC_001224.1..60520..60732 | 0.228 | 9.412 | 0.129 | 0.719 | 553.63 | 409.10 | 721.18 | 425.23 |
| siRNA18 | NC_001133.9..142400..142517 | -0.313 | 8.672 | 0.129 | 0.720 | 237.27 | 327.28 | 160.26 | 283.49 |
| siRNA18 | NC_001143.9..249048..249165 | -0.364 | 8.527 | 0.128 | 0.720 | 79.09 | 409.10 | 80.13 | 283.49 |
| siRNA18 | NC_001137.3..485370..485487 | -0.446 | 8.192 | 0.128 | 0.720 | 237.27 | 81.82 | 240.39 | 0.00 |
| siRNA18 | NC_001135.5..284664..284829 | 0.191 | 9.866 | 0.128 | 0.721 | 553.63 | 900.02 | 881.45 | 779.58 |
| siRNA18 | NC_001140.6..120586..120753 | -0.173 | 10.859 | 0.126 | 0.723 | 1977.26 | 1636.40 | 1201.97 | 1984.40 |
| siRNA18 | NC_001147.6..155496..155617 | -0.299 | 8.677 | 0.116 | 0.733 | 316.36 | 245.46 | 320.53 | 141.74 |
| siRNA18 | NC_001144.5..463006..463328 | 0.166 | 10.691 | 0.116 | 0.733 | 1107.27 | 1718.22 | 1682.76 | 1488.30 |
| siRNA18 | NC_001139.9..458885..459003 | -0.270 | 8.921 | 0.114 | 0.736 | 316.36 | 409.10 | 160.26 | 425.23 |
| siRNA18 | NC_001142.9..284213..284330 | -0.333 | 8.530 | 0.113 | 0.736 | 395.45 | 81.82 | 240.39 | 141.74 |
| siRNA18 | NC_001139.9..813876..813993 | -0.331 | 8.535 | 0.113 | 0.737 | 158.18 | 327.28 | 320.53 | 70.87 |
| siRNA18 | NC_001134.8..550043..550165 | -0.285 | 8.800 | 0.110 | 0.741 | 474.54 | 163.64 | 160.26 | 354.36 |
| siRNA18 | NC_001134.8..660403..660525 | -0.237 | 9.135 | 0.108 | 0.742 | 395.45 | 490.92 | 240.39 | 496.10 |
| siRNA18 | NC_001136.10..419558..419676 | -0.302 | 8.917 | 0.106 | 0.745 | 158.18 | 572.74 | 0.00 | 566.97 |
| siRNA18 | NC_001139.9..932588..932707 | -0.192 | 9.492 | 0.103 | 0.749 | 553.63 | 654.56 | 560.92 | 496.10 |
| siRNA18 | NC_001148.4..435864..435981 | -0.272 | 8.805 | 0.102 | 0.749 | 474.54 | 163.64 | 320.53 | 212.61 |
| siRNA18 | NC_001139.9..979010..979135 | 0.260 | 8.867 | 0.100 | 0.752 | 158.18 | 409.10 | 400.66 | 283.49 |
| siRNA18 | NC_001136.10..802714..802833 | -0.150 | 10.212 | 0.098 | 0.754 | 1107.27 | 1063.66 | 961.58 | 992.20 |
| siRNA18 | NC_001143.9..493535..493653 | -0.266 | 8.918 | 0.092 | 0.761 | 632.72 | 81.82 | 160.26 | 425.23 |
| siRNA18 | NC_001134.8..78264..78381 | -0.186 | 9.493 | 0.089 | 0.765 | 790.90 | 409.10 | 641.05 | 425.23 |
| siRNA18 | NC_001140.6..134290..134408 | -0.162 | 10.118 | 0.082 | 0.775 | 395.45 | 1636.40 | 881.45 | 921.33 |
| siRNA18 | NC_001144.5..461858..462195 | 0.121 | 13.325 | 0.074 | 0.786 | 9965.40 | 9409.27 | 9936.29 | 11126.80 |
| siRNA18 | NC_001143.9..155659..155782 | 0.210 | 8.975 | 0.074 | 0.786 | 316.36 | 327.28 | 240.39 | 496.10 |
| siRNA18 | NC_001136.10..662419..662534 | 0.334 | 8.193 | 0.069 | 0.793 | 158.18 | 81.82 | 320.53 | 0.00 |
| siRNA18 | NC_001136.10..1329744..1329881 | 0.165 | 9.186 | 0.057 | 0.812 | 474.54 | 327.28 | 480.79 | 425.23 |
| siRNA18 | NC_001224.1..58803..59068 | 0.099 | 13.561 | 0.048 | 0.826 | 12654.47 | 10391.11 | 13061.42 | 11622.90 |
| siRNA18 | NC_001147.6..780421..780527 | 0.265 | 8.181 | 0.045 | 0.832 | 79.09 | 163.64 | 0.00 | 283.49 |
| siRNA18 | NC_001144.5..872933..873050 | 0.237 | 8.371 | 0.045 | 0.832 | 237.27 | 81.82 | 320.53 | 70.87 |
| siRNA18 | NC_001145.3..750927..751044 | 0.219 | 8.369 | 0.043 | 0.836 | 158.18 | 163.64 | 240.39 | 141.74 |
| siRNA18 | NC_001137.3..35490..35607 | 0.229 | 8.367 | 0.041 | 0.840 | 316.36 | 0.00 | 240.39 | 141.74 |
| siRNA18 | NC_001145.3..761922..762039 | 0.213 | 8.365 | 0.039 | 0.843 | 237.27 | 81.82 | 160.26 | 212.61 |
| siRNA18 | NC_001137.3..510713..510899 | 0.121 | 9.366 | 0.036 | 0.849 | 395.45 | 572.74 | 480.79 | 566.97 |
| siRNA18 | NC_001138.5..93467..93575 | 0.201 | 8.362 | 0.034 | 0.855 | 237.27 | 81.82 | 80.13 | 283.49 |
| siRNA18 | NC_001134.8..270527..270648 | -0.091 | 9.869 | 0.030 | 0.863 | 870.00 | 736.38 | 881.45 | 637.84 |
| siRNA18 | NC_001147.6..219773..219912 | -0.115 | 9.272 | 0.026 | 0.873 | 395.45 | 572.74 | 160.26 | 708.71 |
| siRNA18 | NC_001147.6..328572..328687 | 0.152 | 8.525 | 0.025 | 0.873 | 158.18 | 245.46 | 160.26 | 283.49 |
| siRNA18 | NC_001137.3..67472..67590 | 0.156 | 8.524 | 0.025 | 0.873 | 316.36 | 81.82 | 160.26 | 283.49 |
| siRNA18 | NC_001136.10..313413..313565 | 0.082 | 9.602 | 0.020 | 0.886 | 553.63 | 654.56 | 641.05 | 637.84 |
| siRNA18 | NC_001134.8..516127..516242 | 0.122 | 8.676 | 0.018 | 0.894 | 79.09 | 409.10 | 320.53 | 212.61 |
| siRNA18 | NC_001139.9..488611..488759 | -0.100 | 8.982 | 0.016 | 0.901 | 158.18 | 572.74 | 320.53 | 354.36 |
| siRNA18 | NC_001144.5..453888..454192 | -0.058 | 10.343 | 0.013 | 0.908 | 870.00 | 1472.76 | 881.45 | 1346.56 |
| siRNA18 | NC_001144.5..465435..465714 | 0.051 | 15.221 | 0.013 | 0.911 | 34008.90 | 40746.25 | 40386.22 | 37065.72 |
| siRNA18 | NC_001134.8..650940..651056 | -0.090 | 8.742 | 0.011 | 0.915 | 237.27 | 327.28 | 320.53 | 212.61 |
| siRNA18 | NC_001144.5..262389..262503 | -0.118 | 8.442 | 0.011 | 0.916 | 316.36 | 81.82 | 0.00 | 354.36 |
| siRNA18 | NC_001144.5..456298..456577 | 0.048 | 15.153 | 0.011 | 0.917 | 31715.27 | 39682.59 | 35738.60 | 38057.92 |
| siRNA18 | NC_001134.8..89395..89511 | -0.125 | 8.275 | 0.011 | 0.918 | 79.09 | 245.46 | 0.00 | 283.49 |
| siRNA18 | NC_001136.10..334934..335051 | -0.096 | 8.605 | 0.010 | 0.919 | 79.09 | 409.10 | 240.39 | 212.61 |
| siRNA18 | NC_001136.10..639262..639371 | -0.096 | 8.605 | 0.010 | 0.919 | 79.09 | 409.10 | 240.39 | 212.61 |
| siRNA18 | NC_001147.6..487717..487834 | -0.090 | 8.602 | 0.010 | 0.921 | 316.36 | 163.64 | 240.39 | 212.61 |
| siRNA18 | NC_001147.6..439749..439872 | -0.047 | 10.589 | 0.010 | 0.922 | 1502.72 | 1309.12 | 1602.63 | 1133.94 |
| siRNA18 | NC_001148.4..811162..811282 | -0.100 | 8.451 | 0.009 | 0.923 | 79.09 | 327.28 | 160.26 | 212.61 |
| siRNA18 | NC_001147.6..495092..495211 | -0.096 | 8.450 | 0.009 | 0.923 | 158.18 | 245.46 | 160.26 | 212.61 |
| siRNA18 | NC_001145.3..783248..783365 | -0.085 | 8.606 | 0.009 | 0.925 | 237.27 | 245.46 | 320.53 | 141.74 |
| siRNA18 | NC_001136.10..635250..635480 | 0.048 | 9.864 | 0.008 | 0.927 | 870.00 | 654.56 | 801.31 | 779.58 |
| siRNA18 | NC_001134.8..456655..456772 | -0.101 | 8.278 | 0.008 | 0.928 | 158.18 | 163.64 | 80.13 | 212.61 |
| siRNA18 | NC_001147.6..874631..874744 | -0.096 | 8.087 | 0.006 | 0.938 | 79.09 | 163.64 | 80.13 | 141.74 |
| siRNA18 | NC_001148.4..820768..820885 | -0.093 | 8.086 | 0.006 | 0.940 | 158.18 | 81.82 | 80.13 | 141.74 |
| siRNA18 | NC_001145.3..160312..160429 | -0.074 | 8.455 | 0.005 | 0.942 | 237.27 | 163.64 | 320.53 | 70.87 |
| siRNA18 | NC_001135.5..246912..247029 | -0.080 | 8.285 | 0.005 | 0.944 | 79.09 | 245.46 | 240.39 | 70.87 |
| siRNA18 | NC_001134.8..639333..639438 | -0.089 | 8.085 | 0.005 | 0.945 | 237.27 | 0.00 | 80.13 | 141.74 |
| siRNA18 | NC_001143.9..414664..414778 | -0.086 | 8.091 | 0.004 | 0.947 | 0.00 | 245.46 | 160.26 | 70.87 |
| siRNA18 | NC_001144.5..491784..491901 | -0.080 | 8.089 | 0.004 | 0.948 | 158.18 | 81.82 | 160.26 | 70.87 |
| siRNA18 | NC_001144.5..465845..466175 | -0.024 | 12.650 | 0.003 | 0.957 | 5536.33 | 7118.32 | 6490.64 | 5953.19 |
| siRNA18 | NC_001143.9..490963..491081 | 0.024 | 9.234 | 0.001 | 0.972 | 316.36 | 572.74 | 400.66 | 496.10 |
| siRNA18 | NC_001145.3..499938..500055 | -0.018 | 10.644 | 0.001 | 0.972 | 949.09 | 1963.67 | 1602.63 | 1275.68 |
| siRNA18 | NC_001136.10..753984..754091 | -0.038 | 8.456 | 0.001 | 0.974 | 395.45 | 0.00 | 400.66 | 0.00 |
| siRNA18 | NC_001136.10..778559..778677 | 0.010 | 10.285 | 0.000 | 0.983 | 870.00 | 1309.12 | 1282.10 | 921.33 |
| siRNA18 | NC_001224.1..6506..6631 | 0.004 | 12.017 | 0.000 | 0.994 | 3717.25 | 4254.63 | 4166.83 | 3827.05 |
| siRNA18 | NC_001148.4..911310..911434 | 0.002 | 9.485 | 0.000 | 0.997 | 790.90 | 327.28 | 480.79 | 637.84 |
| siRNA19 | NC_001140.6..508748..508872 | 6.291 | 9.072 | 21.468 | 0.000 | 0.00 | 0.00 | 721.18 | 779.58 |
| siRNA19 | NC_001139.9..447283..447387 | -5.090 | 8.287 | 9.336 | 0.002 | 395.45 | 245.46 | 0.00 | 0.00 |
| siRNA19 | NC_001148.4..338802..338920 | 2.881 | 9.041 | 7.735 | 0.005 | 0.00 | 163.64 | 1121.84 | 212.61 |
| siRNA19 | NC_001224.1..64386..64504 | -2.649 | 8.813 | 7.714 | 0.005 | 474.54 | 572.74 | 80.13 | 70.87 |
| siRNA19 | NC_001138.5..180960..181084 | -7.289 | 9.804 | 7.703 | 0.006 | 158.18 | 2863.69 | 0.00 | 0.00 |
| siRNA19 | NC_001144.5..381195..381312 | 3.191 | 8.656 | 7.596 | 0.006 | 79.09 | 0.00 | 240.39 | 637.84 |
| siRNA19 | NC_001145.3..364803..364922 | -2.317 | 8.991 | 7.192 | 0.007 | 711.81 | 490.92 | 160.26 | 70.87 |
| siRNA19 | NC_001224.1..44879..44994 | 4.847 | 8.190 | 6.978 | 0.008 | 0.00 | 0.00 | 480.79 | 70.87 |
| siRNA19 | NC_001141.2..231366..231484 | 2.947 | 8.518 | 6.286 | 0.012 | 79.09 | 0.00 | 320.53 | 425.23 |
| siRNA19 | NC_001145.3..356992..357110 | 3.363 | 8.776 | 6.247 | 0.012 | 0.00 | 81.82 | 0.00 | 992.20 |
| siRNA19 | NC_001142.9..679469..679576 | 2.456 | 8.789 | 6.178 | 0.013 | 79.09 | 81.82 | 320.53 | 637.84 |
| siRNA19 | NC_001145.3..504929..505048 | 1.933 | 8.853 | 4.439 | 0.035 | 237.27 | 0.00 | 400.66 | 566.97 |
| siRNA19 | NC_001140.6..156259..156452 | 1.874 | 9.090 | 4.334 | 0.037 | 316.36 | 0.00 | 961.58 | 283.49 |
| siRNA19 | NC_001136.10..756572..756692 | -1.727 | 8.935 | 4.217 | 0.040 | 474.54 | 572.74 | 240.39 | 70.87 |
| siRNA19 | NC_001133.9..170173..170285 | -2.138 | 8.531 | 4.100 | 0.043 | 395.45 | 327.28 | 0.00 | 141.74 |
| siRNA19 | NC_001142.9..520134..520300 | -1.077 | 9.750 | 3.655 | 0.056 | 949.09 | 981.84 | 560.92 | 354.36 |
| siRNA19 | NC_001136.10..1297262..1297381 | 1.441 | 9.078 | 3.436 | 0.064 | 237.27 | 163.64 | 480.79 | 637.84 |
| siRNA19 | NC_001140.6..65503..65616 | 1.705 | 8.735 | 3.432 | 0.064 | 158.18 | 81.82 | 480.79 | 354.36 |
| siRNA19 | NC_001143.9..595902..596009 | -2.414 | 8.190 | 3.378 | 0.066 | 237.27 | 245.46 | 0.00 | 70.87 |
| siRNA19 | NC_001139.9..205555..205674 | 1.715 | 8.738 | 3.164 | 0.075 | 237.27 | 0.00 | 560.92 | 283.49 |
| siRNA19 | NC_001139.9..656210..656330 | -1.604 | 8.879 | 3.082 | 0.079 | 316.36 | 654.56 | 320.53 | 0.00 |
| siRNA19 | NC_001142.9..517767..517885 | -2.424 | 8.192 | 3.066 | 0.080 | 79.09 | 409.10 | 0.00 | 70.87 |
| siRNA19 | NC_001144.5..953182..953294 | -2.410 | 8.189 | 3.050 | 0.081 | 395.45 | 81.82 | 0.00 | 70.87 |
| siRNA19 | NC_001148.4..606275..606396 | 1.017 | 9.807 | 2.865 | 0.091 | 316.36 | 654.56 | 1362.23 | 637.84 |
| siRNA19 | NC_001134.8..331240..331343 | 2.241 | 8.183 | 2.855 | 0.091 | 79.09 | 0.00 | 240.39 | 212.61 |
| siRNA19 | NC_001147.6..613106..613226 | -1.603 | 8.606 | 2.841 | 0.092 | 395.45 | 327.28 | 80.13 | 141.74 |
| siRNA19 | NC_001136.10..1461675..1461793 | 1.570 | 8.667 | 2.685 | 0.101 | 237.27 | 0.00 | 400.66 | 354.36 |
| siRNA19 | NC_001144.5..51593..51717 | 0.909 | 9.929 | 2.500 | 0.114 | 553.63 | 572.74 | 1522.50 | 637.84 |
| siRNA19 | NC_001146.8..154326..154444 | 1.769 | 8.440 | 2.487 | 0.115 | 79.09 | 81.82 | 160.26 | 425.23 |
| siRNA19 | NC_001146.8..603499..603620 | 0.992 | 9.685 | 2.327 | 0.127 | 553.63 | 327.28 | 400.66 | 1346.56 |
| siRNA19 | NC_001139.9..513793..513911 | -1.787 | 8.368 | 2.321 | 0.128 | 395.45 | 163.64 | 0.00 | 141.74 |
| siRNA19 | NC_001147.6..320935..321047 | -1.631 | 8.606 | 2.249 | 0.134 | 79.09 | 654.56 | 0.00 | 212.61 |
| siRNA19 | NC_001143.9..113179..113296 | 1.991 | 8.087 | 2.094 | 0.148 | 0.00 | 81.82 | 240.39 | 141.74 |
| siRNA19 | NC_001147.6..178751..178871 | 0.884 | 9.519 | 2.093 | 0.148 | 395.45 | 409.10 | 641.05 | 850.46 |
| siRNA19 | NC_001138.5..165715..165820 | -2.129 | 8.091 | 2.064 | 0.151 | 395.45 | 0.00 | 80.13 | 0.00 |
| siRNA19 | NC_001144.5..445961..446068 | -1.202 | 8.867 | 2.056 | 0.152 | 395.45 | 490.92 | 80.13 | 283.49 |
| siRNA19 | NC_001144.5..909482..909599 | -1.448 | 8.528 | 1.993 | 0.158 | 395.45 | 245.46 | 0.00 | 212.61 |
| siRNA19 | NC_001136.10..1329315..1329420 | -1.435 | 8.530 | 1.973 | 0.160 | 474.54 | 163.64 | 80.13 | 141.74 |
| siRNA19 | NC_001136.10..1238971..1239088 | 1.970 | 8.079 | 1.967 | 0.161 | 79.09 | 0.00 | 80.13 | 283.49 |
| siRNA19 | NC_001136.10..1265099..1265217 | 1.615 | 8.369 | 1.924 | 0.165 | 79.09 | 81.82 | 400.66 | 141.74 |
| siRNA19 | NC_001139.9..375556..375670 | -1.548 | 8.287 | 1.698 | 0.193 | 237.27 | 245.46 | 160.26 | 0.00 |
| siRNA19 | NC_001139.9..989604..989722 | -1.588 | 8.283 | 1.608 | 0.205 | 79.09 | 409.10 | 0.00 | 141.74 |
| siRNA19 | NC_001141.2..168789..168902 | 1.246 | 8.522 | 1.558 | 0.212 | 79.09 | 163.64 | 240.39 | 354.36 |
| siRNA19 | NC_001135.5..535..653 | 1.258 | 8.524 | 1.508 | 0.219 | 237.27 | 0.00 | 320.53 | 283.49 |
| siRNA19 | NC_001136.10..181721..181844 | -0.546 | 13.049 | 1.501 | 0.221 | 10439.94 | 9327.45 | 7372.09 | 6165.81 |
| siRNA19 | NC_001147.6..1054911..1055029 | 1.250 | 8.526 | 1.484 | 0.223 | 0.00 | 245.46 | 320.53 | 283.49 |
| siRNA19 | NC_001142.9..108101..108219 | 1.240 | 8.518 | 1.466 | 0.226 | 158.18 | 81.82 | 160.26 | 425.23 |
| siRNA19 | NC_001147.6..828422..828718 | 0.544 | 12.675 | 1.461 | 0.227 | 4824.52 | 5563.74 | 6490.64 | 8646.31 |
| siRNA19 | NC_001136.10..239486..239604 | -1.250 | 8.451 | 1.412 | 0.235 | 395.45 | 163.64 | 80.13 | 141.74 |
| siRNA19 | NC_001136.10..552388..552508 | 0.876 | 9.133 | 1.407 | 0.236 | 158.18 | 409.10 | 480.79 | 566.97 |
| siRNA19 | NC_001144.5..1027554..1027674 | 1.286 | 8.534 | 1.398 | 0.237 | 158.18 | 81.82 | 560.92 | 70.87 |
| siRNA19 | NC_001148.4..626924..627040 | -1.086 | 8.803 | 1.378 | 0.240 | 237.27 | 572.74 | 0.00 | 354.36 |
| siRNA19 | NC_001134.8..552238..552355 | 1.379 | 8.273 | 1.310 | 0.252 | 158.18 | 0.00 | 160.26 | 283.49 |
| siRNA19 | NC_001145.3..753389..753763 | -0.546 | 10.875 | 1.278 | 0.258 | 2214.53 | 1881.85 | 1682.76 | 1133.94 |
| siRNA19 | NC_001137.3..90704..90821 | 1.408 | 8.285 | 1.236 | 0.266 | 0.00 | 163.64 | 400.66 | 70.87 |
| siRNA19 | NC_001144.5..197797..197915 | -1.043 | 8.807 | 1.230 | 0.267 | 711.81 | 81.82 | 240.39 | 141.74 |
| siRNA19 | NC_001148.4..719242..719359 | -1.309 | 8.190 | 1.188 | 0.276 | 237.27 | 163.64 | 80.13 | 70.87 |
| siRNA19 | NC_001135.5..49524..49652 | -0.570 | 9.956 | 1.178 | 0.278 | 790.90 | 1227.30 | 560.92 | 779.58 |
| siRNA19 | NC_001137.3..3408..3675 | 0.480 | 15.540 | 1.107 | 0.293 | 38833.41 | 40500.79 | 52085.40 | 58539.74 |
| siRNA19 | NC_001147.6..70241..70359 | -1.297 | 8.194 | 1.097 | 0.295 | 158.18 | 245.46 | 160.26 | 0.00 |
| siRNA19 | NC_001135.5..195078..195255 | 0.874 | 8.801 | 1.078 | 0.299 | 237.27 | 163.64 | 400.66 | 354.36 |
| siRNA19 | NC_001144.5..815390..815519 | -0.646 | 9.604 | 1.076 | 0.300 | 474.54 | 1063.66 | 320.53 | 637.84 |
| siRNA19 | NC_001134.8..585729..585922 | -0.516 | 10.428 | 1.067 | 0.302 | 1660.90 | 1227.30 | 721.18 | 1275.68 |
| siRNA19 | NC_001141.2..365489..365660 | 0.869 | 8.798 | 1.058 | 0.304 | 237.27 | 163.64 | 320.53 | 425.23 |
| siRNA19 | NC_001139.9..857338..857457 | 0.805 | 9.230 | 1.053 | 0.305 | 632.72 | 0.00 | 641.05 | 496.10 |
| siRNA19 | NC_001137.3..350040..350157 | 0.868 | 8.799 | 1.052 | 0.305 | 158.18 | 245.46 | 320.53 | 425.23 |
| siRNA19 | NC_001137.3..403830..403948 | 1.063 | 8.446 | 1.052 | 0.305 | 79.09 | 163.64 | 240.39 | 283.49 |
| siRNA19 | NC_001148.4..141638..141759 | 1.057 | 8.442 | 1.004 | 0.316 | 158.18 | 81.82 | 160.26 | 354.36 |
| siRNA19 | NC_001144.5..203291..203412 | 1.054 | 8.443 | 0.997 | 0.318 | 79.09 | 163.64 | 160.26 | 354.36 |
| siRNA19 | NC_001136.10..619928..620046 | 0.865 | 8.799 | 0.997 | 0.318 | 79.09 | 327.28 | 320.53 | 425.23 |
| siRNA19 | NC_001146.8..511535..511652 | -1.039 | 8.369 | 0.933 | 0.334 | 237.27 | 245.46 | 80.13 | 141.74 |
| siRNA19 | NC_001139.9..108820..108921 | -1.298 | 8.196 | 0.902 | 0.342 | 0.00 | 409.10 | 160.26 | 0.00 |
| siRNA19 | NC_001145.3..64679..64790 | -1.298 | 8.196 | 0.902 | 0.342 | 0.00 | 409.10 | 160.26 | 0.00 |
| siRNA19 | NC_001143.9..605629..605747 | 1.140 | 8.187 | 0.888 | 0.346 | 79.09 | 81.82 | 240.39 | 141.74 |
| siRNA19 | NC_001140.6..299820..299932 | -1.025 | 8.372 | 0.884 | 0.347 | 316.36 | 163.64 | 160.26 | 70.87 |
| siRNA19 | NC_001136.10..912634..912752 | 0.875 | 8.602 | 0.854 | 0.355 | 237.27 | 81.82 | 400.66 | 212.61 |
| siRNA19 | NC_001136.10..938099..938223 | -0.464 | 10.044 | 0.840 | 0.359 | 1028.18 | 1063.66 | 961.58 | 566.97 |
| siRNA19 | NC_001139.9..802918..803036 | 0.857 | 8.598 | 0.832 | 0.362 | 79.09 | 245.46 | 240.39 | 354.36 |
| siRNA19 | NC_001148.4..19720..19837 | 1.125 | 8.184 | 0.825 | 0.364 | 0.00 | 163.64 | 160.26 | 212.61 |
| siRNA19 | NC_001140.6..130115..130215 | 1.117 | 8.180 | 0.812 | 0.368 | 79.09 | 81.82 | 80.13 | 283.49 |
| siRNA19 | NC_001147.6..413745..413863 | -0.871 | 8.529 | 0.809 | 0.368 | 237.27 | 327.28 | 80.13 | 212.61 |
| siRNA19 | NC_001133.9..181175..181294 | 0.872 | 8.598 | 0.799 | 0.371 | 316.36 | 0.00 | 320.53 | 283.49 |
| siRNA19 | NC_001145.3..702399..702611 | -0.671 | 9.090 | 0.798 | 0.372 | 316.36 | 654.56 | 240.39 | 354.36 |
| siRNA19 | NC_001224.1..84134..84309 | 0.420 | 11.153 | 0.771 | 0.380 | 1739.99 | 1881.85 | 2884.73 | 1984.40 |
| siRNA19 | NC_001146.8..732547..732651 | -1.035 | 8.375 | 0.730 | 0.393 | 0.00 | 490.92 | 160.26 | 70.87 |
| siRNA19 | NC_001148.4..247874..247992 | 0.729 | 8.739 | 0.716 | 0.397 | 237.27 | 163.64 | 400.66 | 283.49 |
| siRNA19 | NC_001139.9..105553..105680 | 0.681 | 9.041 | 0.711 | 0.399 | 237.27 | 327.28 | 721.18 | 212.61 |
| siRNA19 | NC_001137.3..256645..256762 | -0.866 | 8.526 | 0.708 | 0.400 | 474.54 | 81.82 | 80.13 | 212.61 |
| siRNA19 | NC_001135.5..149920..150037 | 1.097 | 8.177 | 0.696 | 0.404 | 79.09 | 81.82 | 0.00 | 354.36 |
| siRNA19 | NC_001140.6..512995..513115 | 0.716 | 8.734 | 0.679 | 0.410 | 158.18 | 245.46 | 240.39 | 425.23 |
| siRNA19 | NC_001148.4..509789..509911 | 0.450 | 9.767 | 0.674 | 0.412 | 711.81 | 490.92 | 801.31 | 850.46 |
| siRNA19 | NC_001145.3..860532..860651 | 0.720 | 8.737 | 0.669 | 0.413 | 79.09 | 327.28 | 320.53 | 354.36 |
| siRNA19 | NC_001139.9..136466..136614 | -0.663 | 8.804 | 0.636 | 0.425 | 395.45 | 327.28 | 160.26 | 283.49 |
| siRNA19 | NC_001137.3..31462..31572 | -1.026 | 8.087 | 0.622 | 0.430 | 79.09 | 245.46 | 0.00 | 141.74 |
| siRNA19 | NC_001134.8..449178..449297 | 0.621 | 8.863 | 0.592 | 0.442 | 237.27 | 245.46 | 400.66 | 354.36 |
| siRNA19 | NC_001136.10..1200019..1200131 | -0.983 | 8.092 | 0.570 | 0.450 | 237.27 | 81.82 | 160.26 | 0.00 |
| siRNA19 | NC_001137.3..100133..100250 | -1.038 | 8.088 | 0.566 | 0.452 | 0.00 | 327.28 | 0.00 | 141.74 |
| siRNA19 | NC_001139.9..1051048..1051153 | -0.996 | 8.088 | 0.555 | 0.456 | 316.36 | 0.00 | 80.13 | 70.87 |
| siRNA19 | NC_001144.5..795733..795852 | -0.586 | 8.927 | 0.530 | 0.466 | 553.63 | 245.46 | 320.53 | 212.61 |
| siRNA19 | NC_001136.10..1517493..1517611 | 0.676 | 8.525 | 0.500 | 0.479 | 158.18 | 163.64 | 240.39 | 283.49 |
| siRNA19 | NC_001146.8..309923..310041 | 0.676 | 8.525 | 0.500 | 0.479 | 158.18 | 163.64 | 240.39 | 283.49 |
| siRNA19 | NC_001139.9..556350..556464 | -0.537 | 9.037 | 0.481 | 0.488 | 632.72 | 245.46 | 320.53 | 283.49 |
| siRNA19 | NC_001147.6..246676..246793 | -0.774 | 8.284 | 0.477 | 0.490 | 237.27 | 163.64 | 160.26 | 70.87 |
| siRNA19 | NC_001147.6..217281..217399 | 0.690 | 8.532 | 0.476 | 0.490 | 79.09 | 245.46 | 400.66 | 141.74 |
| siRNA19 | NC_001142.9..177534..177641 | 0.830 | 8.086 | 0.441 | 0.507 | 79.09 | 81.82 | 160.26 | 141.74 |
| siRNA19 | NC_001144.5..200431..200554 | 0.396 | 9.739 | 0.424 | 0.515 | 949.09 | 245.46 | 961.58 | 637.84 |
| siRNA19 | NC_001147.6..560787..560904 | 0.818 | 8.083 | 0.416 | 0.519 | 79.09 | 81.82 | 80.13 | 212.61 |
| siRNA19 | NC_001136.10..1210303..1210421 | -0.660 | 8.450 | 0.413 | 0.520 | 158.18 | 327.28 | 80.13 | 212.61 |
| siRNA19 | NC_001147.6..200566..200676 | 0.865 | 8.093 | 0.408 | 0.523 | 79.09 | 81.82 | 320.53 | 0.00 |
| siRNA19 | NC_001148.4..281280..281395 | 0.865 | 8.093 | 0.408 | 0.523 | 79.09 | 81.82 | 320.53 | 0.00 |
| siRNA19 | NC_001147.6..813023..813142 | 0.492 | 9.090 | 0.402 | 0.526 | 474.54 | 163.64 | 641.05 | 283.49 |
| siRNA19 | NC_001144.5..737155..737594 | -0.286 | 11.880 | 0.390 | 0.532 | 4429.07 | 3518.25 | 3766.18 | 2763.98 |
| siRNA19 | NC_001147.6..608697..608816 | 0.574 | 8.677 | 0.390 | 0.532 | 158.18 | 245.46 | 480.79 | 141.74 |
| siRNA19 | NC_001146.8..624933..625049 | -0.562 | 8.603 | 0.386 | 0.535 | 237.27 | 327.28 | 160.26 | 212.61 |
| siRNA19 | NC_001136.10..593772..593891 | -0.653 | 8.454 | 0.386 | 0.535 | 79.09 | 409.10 | 160.26 | 141.74 |
| siRNA19 | NC_001147.6..175540..175650 | 0.862 | 8.094 | 0.382 | 0.536 | 0.00 | 163.64 | 320.53 | 0.00 |
| siRNA19 | NC_001146.8..709042..709160 | -0.555 | 8.606 | 0.375 | 0.540 | 237.27 | 327.28 | 240.39 | 141.74 |
| siRNA19 | NC_001146.8..261688..261807 | 0.382 | 9.279 | 0.336 | 0.562 | 395.45 | 409.10 | 560.92 | 496.10 |
| siRNA19 | NC_001142.9..276437..276555 | -0.651 | 8.447 | 0.330 | 0.566 | 474.54 | 0.00 | 80.13 | 212.61 |
| siRNA19 | NC_001135.5..121454..121670 | -0.275 | 10.068 | 0.306 | 0.580 | 870.00 | 1145.48 | 961.58 | 708.71 |
| siRNA19 | NC_001140.6..8921..9039 | -0.610 | 8.457 | 0.295 | 0.587 | 395.45 | 81.82 | 320.53 | 0.00 |
| siRNA19 | NC_001224.1..32183..32307 | 0.452 | 9.320 | 0.286 | 0.593 | 0.00 | 818.20 | 400.66 | 708.71 |
| siRNA19 | NC_001139.9..477473..477590 | 0.489 | 8.811 | 0.286 | 0.593 | 79.09 | 409.10 | 560.92 | 141.74 |
| siRNA19 | NC_001146.8..163638..163755 | 0.596 | 8.278 | 0.282 | 0.595 | 79.09 | 163.64 | 160.26 | 212.61 |
| siRNA19 | NC_001224.1..56762..56881 | 0.799 | 9.333 | 0.281 | 0.596 | 711.81 | 0.00 | 1121.84 | 141.74 |
| siRNA19 | NC_001147.6..811227..811347 | -0.445 | 8.871 | 0.277 | 0.599 | 158.18 | 572.74 | 320.53 | 212.61 |
| siRNA19 | NC_001139.9..847070..847184 | 0.632 | 8.283 | 0.268 | 0.604 | 237.27 | 0.00 | 320.53 | 70.87 |
| siRNA19 | NC_001137.3..492311..492429 | -0.403 | 8.982 | 0.267 | 0.605 | 553.63 | 245.46 | 320.53 | 283.49 |
| siRNA19 | NC_001141.2..350264..350386 | -0.354 | 9.187 | 0.264 | 0.607 | 474.54 | 490.92 | 320.53 | 425.23 |
| siRNA19 | NC_001137.3..140165..140277 | 0.603 | 8.276 | 0.264 | 0.608 | 237.27 | 0.00 | 160.26 | 212.61 |
| siRNA19 | NC_001224.1..19042..19157 | 0.603 | 8.282 | 0.261 | 0.609 | 0.00 | 245.46 | 240.39 | 141.74 |
| siRNA19 | NC_001145.3..249890..250003 | 0.587 | 8.274 | 0.259 | 0.611 | 158.18 | 81.82 | 80.13 | 283.49 |
| siRNA19 | NC_001147.6..552536..552689 | -0.395 | 9.085 | 0.253 | 0.615 | 237.27 | 654.56 | 160.26 | 496.10 |
| siRNA19 | NC_001136.10..536206..536391 | -0.369 | 9.094 | 0.248 | 0.619 | 395.45 | 490.92 | 480.79 | 212.61 |
| siRNA19 | NC_001134.8..661010..661174 | -0.323 | 9.286 | 0.233 | 0.629 | 553.63 | 490.92 | 560.92 | 283.49 |
| siRNA19 | NC_001146.8..536479..536593 | 0.569 | 8.271 | 0.215 | 0.643 | 158.18 | 81.82 | 0.00 | 354.36 |
| siRNA19 | NC_001142.9..701195..701313 | 0.463 | 8.450 | 0.207 | 0.649 | 79.09 | 245.46 | 240.39 | 212.61 |
| siRNA19 | NC_001146.8..244553..244670 | 0.485 | 8.456 | 0.203 | 0.652 | 79.09 | 245.46 | 400.66 | 70.87 |
| siRNA19 | NC_001144.5..512008..512125 | 0.454 | 8.447 | 0.196 | 0.658 | 79.09 | 245.46 | 160.26 | 283.49 |
| siRNA19 | NC_001148.4..22814..22932 | 0.312 | 9.138 | 0.193 | 0.660 | 316.36 | 409.10 | 480.79 | 425.23 |
| siRNA19 | NC_001141.2..186233..186349 | 0.460 | 8.451 | 0.184 | 0.668 | 0.00 | 327.28 | 240.39 | 212.61 |
| siRNA19 | NC_001143.9..179650..179750 | 0.449 | 8.442 | 0.179 | 0.672 | 237.27 | 81.82 | 80.13 | 354.36 |
| siRNA19 | NC_001147.6..340258..340376 | 0.304 | 9.132 | 0.178 | 0.673 | 395.45 | 327.28 | 320.53 | 566.97 |
| siRNA19 | NC_001136.10..992782..992916 | 2.177 | 11.835 | 0.178 | 0.674 | 0.00 | 2536.41 | 11538.92 | 0.00 |
| siRNA19 | NC_001134.8..422900..423018 | 0.442 | 8.444 | 0.173 | 0.677 | 79.09 | 245.46 | 80.13 | 354.36 |
| siRNA19 | NC_001144.5..720973..721082 | -0.499 | 8.184 | 0.168 | 0.682 | 158.18 | 163.64 | 0.00 | 212.61 |
| siRNA19 | NC_001145.3..24794..24909 | 0.370 | 8.600 | 0.167 | 0.683 | 158.18 | 245.46 | 240.39 | 283.49 |
| siRNA19 | NC_001145.3..73495..73624 | 0.249 | 9.593 | 0.167 | 0.683 | 474.54 | 654.56 | 400.66 | 921.33 |
| siRNA19 | NC_001148.4..246201..246318 | -0.476 | 8.185 | 0.144 | 0.705 | 316.36 | 0.00 | 80.13 | 141.74 |
| siRNA19 | NC_001147.6..95704..95821 | -0.397 | 8.370 | 0.141 | 0.707 | 158.18 | 245.46 | 160.26 | 141.74 |
| siRNA19 | NC_001141.2..176570..176687 | 0.310 | 8.738 | 0.133 | 0.716 | 316.36 | 163.64 | 320.53 | 283.49 |
| siRNA19 | NC_001148.4..180025..180143 | 0.237 | 9.589 | 0.132 | 0.716 | 474.54 | 654.56 | 240.39 | 1063.07 |
| siRNA19 | NC_001142.9..549313..549429 | -0.421 | 8.368 | 0.128 | 0.721 | 0.00 | 409.10 | 80.13 | 212.61 |
| siRNA19 | NC_001143.9..431823..432029 | -0.172 | 10.494 | 0.120 | 0.729 | 1660.90 | 1063.66 | 1442.37 | 992.20 |
| siRNA19 | NC_001134.8..270200..270326 | 0.269 | 8.869 | 0.107 | 0.743 | 316.36 | 245.46 | 480.79 | 212.61 |
| siRNA19 | NC_001224.1..43943..44071 | -0.206 | 9.409 | 0.104 | 0.747 | 395.45 | 736.38 | 400.66 | 566.97 |
| siRNA19 | NC_001148.4..297529..297669 | 0.183 | 9.567 | 0.098 | 0.754 | 553.63 | 572.74 | 721.18 | 566.97 |
| siRNA19 | NC_001145.3..703252..703372 | -0.294 | 8.807 | 0.095 | 0.758 | 0.00 | 654.56 | 240.39 | 283.49 |
| siRNA19 | NC_001145.3..25747..25889 | 0.253 | 8.859 | 0.094 | 0.759 | 395.45 | 163.64 | 240.39 | 425.23 |
| siRNA19 | NC_001136.10..2375..2489 | -0.369 | 8.378 | 0.088 | 0.767 | 0.00 | 409.10 | 320.53 | 0.00 |
| siRNA19 | NC_001135.5..282885..283010 | 0.146 | 10.516 | 0.081 | 0.775 | 1581.81 | 900.02 | 961.58 | 1771.78 |
| siRNA19 | NC_001142.9..269135..269254 | -0.216 | 9.041 | 0.068 | 0.795 | 632.72 | 163.64 | 560.92 | 141.74 |
| siRNA19 | NC_001144.5..502925..503043 | 0.300 | 8.186 | 0.066 | 0.797 | 158.18 | 81.82 | 160.26 | 141.74 |
| siRNA19 | NC_001145.3..801067..801167 | 0.300 | 8.186 | 0.066 | 0.797 | 158.18 | 81.82 | 160.26 | 141.74 |
| siRNA19 | NC_001144.5..456117..456276 | 0.110 | 13.385 | 0.056 | 0.813 | 9569.95 | 10718.39 | 13622.34 | 8291.95 |
| siRNA19 | NC_001142.9..113392..113509 | 0.290 | 8.182 | 0.055 | 0.815 | 237.27 | 0.00 | 80.13 | 212.61 |
| siRNA19 | NC_001134.8..197544..197749 | 0.346 | 14.674 | 0.047 | 0.828 | 43816.11 | 1963.67 | 28847.30 | 29340.74 |
| siRNA19 | NC_001144.5..465253..465413 | 0.085 | 13.388 | 0.036 | 0.850 | 10281.76 | 10227.47 | 12340.23 | 9425.89 |
| siRNA19 | NC_001145.3..25045..25163 | 0.186 | 8.533 | 0.033 | 0.856 | 316.36 | 81.82 | 400.66 | 70.87 |
| siRNA19 | NC_001134.8..517966..518083 | 0.173 | 8.530 | 0.031 | 0.861 | 316.36 | 81.82 | 320.53 | 141.74 |
| siRNA19 | NC_001136.10..95389..95495 | 0.186 | 8.364 | 0.026 | 0.873 | 0.00 | 327.28 | 80.13 | 283.49 |
| siRNA19 | NC_001137.3..225154..225274 | 0.152 | 8.525 | 0.025 | 0.873 | 158.18 | 245.46 | 160.26 | 283.49 |
| siRNA19 | NC_001142.9..372156..372274 | -0.094 | 9.452 | 0.024 | 0.877 | 553.63 | 572.74 | 560.92 | 496.10 |
| siRNA19 | NC_001147.6..780048..780170 | 0.127 | 8.673 | 0.021 | 0.885 | 316.36 | 163.64 | 320.53 | 212.61 |
| siRNA19 | NC_001136.10..630419..630538 | -0.103 | 8.979 | 0.017 | 0.895 | 237.27 | 490.92 | 240.39 | 425.23 |
| siRNA19 | NC_001139.9..449897..450016 | -0.095 | 8.980 | 0.016 | 0.901 | 395.45 | 327.28 | 320.53 | 354.36 |
| siRNA19 | NC_001136.10..814948..815069 | 0.107 | 8.805 | 0.015 | 0.903 | 474.54 | 81.82 | 400.66 | 212.61 |
| siRNA19 | NC_001147.6..546032..546155 | -0.093 | 8.979 | 0.015 | 0.904 | 474.54 | 245.46 | 320.53 | 354.36 |
| siRNA19 | NC_001135.5..103011..103130 | 0.099 | 8.807 | 0.014 | 0.905 | 237.27 | 327.28 | 400.66 | 212.61 |
| siRNA19 | NC_001136.10..896699..896813 | -0.110 | 8.598 | 0.013 | 0.908 | 158.18 | 327.28 | 80.13 | 354.36 |
| siRNA19 | NC_001139.9..672548..672672 | 0.065 | 9.667 | 0.013 | 0.909 | 632.72 | 654.56 | 480.79 | 850.46 |
| siRNA19 | NC_001147.6..710201..710318 | -0.137 | 8.276 | 0.011 | 0.916 | 0.00 | 327.28 | 0.00 | 283.49 |
| siRNA19 | NC_001142.9..365362..365480 | -0.094 | 8.604 | 0.011 | 0.918 | 158.18 | 327.28 | 240.39 | 212.61 |
| siRNA19 | NC_001145.3..77193..77315 | -0.092 | 8.603 | 0.011 | 0.918 | 237.27 | 245.46 | 240.39 | 212.61 |
| siRNA19 | NC_001136.10..345719..345837 | -0.090 | 8.602 | 0.010 | 0.921 | 316.36 | 163.64 | 240.39 | 212.61 |
| siRNA19 | NC_001136.10..61925..62036 | -0.094 | 8.449 | 0.009 | 0.925 | 237.27 | 163.64 | 160.26 | 212.61 |
| siRNA19 | NC_001134.8..574042..574160 | -0.082 | 8.605 | 0.008 | 0.928 | 316.36 | 163.64 | 320.53 | 141.74 |
| siRNA19 | NC_001142.9..57564..57681 | -0.085 | 8.452 | 0.007 | 0.932 | 237.27 | 163.64 | 240.39 | 141.74 |
| siRNA19 | NC_001139.9..366328..366443 | -0.110 | 8.083 | 0.007 | 0.932 | 158.18 | 81.82 | 0.00 | 212.61 |
| siRNA19 | NC_001148.4..601941..602059 | -0.093 | 8.454 | 0.007 | 0.932 | 0.00 | 409.10 | 240.39 | 141.74 |
| siRNA19 | NC_001145.3..888768..888885 | -0.090 | 8.281 | 0.007 | 0.935 | 158.18 | 163.64 | 160.26 | 141.74 |
| siRNA19 | NC_001140.6..307141..307256 | -0.077 | 8.456 | 0.006 | 0.940 | 158.18 | 245.46 | 320.53 | 70.87 |
| siRNA19 | NC_001133.9..116563..116670 | -0.074 | 8.455 | 0.005 | 0.942 | 237.27 | 163.64 | 320.53 | 70.87 |
| siRNA19 | NC_001143.9..65106..65213 | -0.089 | 8.085 | 0.005 | 0.945 | 237.27 | 0.00 | 80.13 | 141.74 |
| siRNA19 | NC_001145.3..183523..183637 | -0.089 | 8.085 | 0.005 | 0.945 | 237.27 | 0.00 | 80.13 | 141.74 |
| siRNA19 | NC_001136.10..1251488..1251606 | -0.065 | 8.607 | 0.004 | 0.947 | 395.45 | 81.82 | 400.66 | 70.87 |
| siRNA19 | NC_001140.6..28936..29054 | -0.073 | 8.283 | 0.004 | 0.948 | 237.27 | 81.82 | 240.39 | 70.87 |
| siRNA19 | NC_001139.9..752484..752587 | -0.073 | 8.088 | 0.003 | 0.955 | 237.27 | 0.00 | 160.26 | 70.87 |
| siRNA19 | NC_001137.3..139870..139991 | 0.040 | 9.137 | 0.003 | 0.955 | 474.54 | 327.28 | 400.66 | 425.23 |
| siRNA19 | NC_001146.8..337254..337641 | 0.025 | 10.585 | 0.003 | 0.958 | 1186.36 | 1554.58 | 1442.37 | 1346.56 |
| siRNA19 | NC_001134.8..569958..570069 | -0.060 | 8.093 | 0.002 | 0.962 | 158.18 | 81.82 | 240.39 | 0.00 |
| siRNA19 | NC_001136.10..103568..103687 | 0.018 | 9.226 | 0.001 | 0.980 | 632.72 | 245.46 | 240.39 | 637.84 |
| siRNA20 | NC_001143.9..432533..432657 | 2.105 | 9.855 | 12.272 | 0.000 | 158.18 | 409.10 | 1362.23 | 1133.94 |
| siRNA20 | NC_001145.3..539770..539897 | -2.758 | 8.871 | 8.146 | 0.004 | 711.81 | 409.10 | 0.00 | 141.74 |
| siRNA20 | NC_001139.9..1016696..1016815 | 3.103 | 8.603 | 6.779 | 0.009 | 0.00 | 81.82 | 641.05 | 212.61 |
| siRNA20 | NC_001140.6..71633..71752 | 2.072 | 9.213 | 6.145 | 0.013 | 316.36 | 0.00 | 400.66 | 992.20 |
| siRNA20 | NC_001146.8..282649..282828 | -1.816 | 9.192 | 5.864 | 0.015 | 711.81 | 654.56 | 80.13 | 283.49 |
| siRNA20 | NC_001142.9..538507..538626 | 1.705 | 9.227 | 5.409 | 0.020 | 158.18 | 245.46 | 721.18 | 637.84 |
| siRNA20 | NC_001136.10..760717..760836 | -2.279 | 8.610 | 5.065 | 0.024 | 316.36 | 490.92 | 80.13 | 70.87 |
| siRNA20 | NC_001148.4..174121..174254 | -1.825 | 9.186 | 4.175 | 0.041 | 1107.27 | 245.46 | 0.00 | 354.36 |
| siRNA20 | NC_001147.6..211796..211915 | -1.736 | 8.934 | 4.053 | 0.044 | 316.36 | 736.38 | 160.26 | 141.74 |
| siRNA20 | NC_001143.9..285995..286122 | 0.925 | 12.371 | 4.048 | 0.044 | 3400.89 | 3681.89 | 5368.80 | 8079.34 |
| siRNA20 | NC_001136.10..936700..936819 | 1.820 | 8.795 | 4.041 | 0.044 | 158.18 | 81.82 | 400.66 | 496.10 |
| siRNA20 | NC_001148.4..286223..286340 | -2.625 | 8.283 | 4.030 | 0.045 | 395.45 | 163.64 | 0.00 | 70.87 |
| siRNA20 | NC_001136.10..576467..576583 | -2.619 | 8.290 | 3.670 | 0.055 | 79.09 | 490.92 | 80.13 | 0.00 |
| siRNA20 | NC_001138.5..201825..201936 | -2.110 | 8.536 | 3.611 | 0.057 | 553.63 | 163.64 | 160.26 | 0.00 |
| siRNA20 | NC_001142.9..529276..529395 | 2.592 | 8.349 | 3.293 | 0.070 | 79.09 | 0.00 | 0.00 | 566.97 |
| siRNA20 | NC_001138.5..60921..61045 | -1.866 | 8.745 | 3.255 | 0.071 | 790.90 | 81.82 | 160.26 | 70.87 |
| siRNA20 | NC_001146.8..576673..576780 | 1.970 | 8.532 | 3.040 | 0.081 | 0.00 | 163.64 | 560.92 | 141.74 |
| siRNA20 | NC_001148.4..442670..442791 | 1.130 | 9.406 | 3.000 | 0.083 | 237.27 | 409.10 | 801.31 | 637.84 |
| siRNA20 | NC_001148.4..445301..445424 | -1.420 | 8.993 | 2.961 | 0.085 | 395.45 | 654.56 | 320.53 | 70.87 |
| siRNA20 | NC_001148.4..829677..829803 | -1.253 | 9.248 | 2.863 | 0.091 | 395.45 | 900.02 | 400.66 | 141.74 |
| siRNA20 | NC_001148.4..628561..628661 | 1.561 | 8.665 | 2.791 | 0.095 | 79.09 | 163.64 | 320.53 | 425.23 |
| siRNA20 | NC_001140.6..294900..295039 | -1.161 | 9.193 | 2.533 | 0.111 | 790.90 | 409.10 | 320.53 | 212.61 |
| siRNA20 | NC_001138.5..129440..129564 | 1.115 | 9.392 | 2.503 | 0.114 | 474.54 | 163.64 | 400.66 | 992.20 |
| siRNA20 | NC_001138.5..215832..215968 | 0.943 | 9.656 | 2.278 | 0.131 | 632.72 | 245.46 | 560.92 | 1133.94 |
| siRNA20 | NC_001143.9..451167..451271 | 1.417 | 8.596 | 2.193 | 0.139 | 158.18 | 81.82 | 320.53 | 354.36 |
| siRNA20 | NC_001144.5..44477..44588 | 1.996 | 8.086 | 2.104 | 0.147 | 79.09 | 0.00 | 240.39 | 141.74 |
| siRNA20 | NC_001142.9..288821..288971 | -0.723 | 10.389 | 2.095 | 0.148 | 1660.90 | 1309.12 | 641.05 | 1133.94 |
| siRNA20 | NC_001143.9..435975..436093 | 1.175 | 8.732 | 1.741 | 0.187 | 237.27 | 81.82 | 320.53 | 425.23 |
| siRNA20 | NC_001147.6..306376..306491 | 1.951 | 8.076 | 1.729 | 0.189 | 79.09 | 0.00 | 0.00 | 354.36 |
| siRNA20 | NC_001135.5..251292..251410 | -0.970 | 9.089 | 1.685 | 0.194 | 553.63 | 490.92 | 160.26 | 354.36 |
| siRNA20 | NC_001143.9..244288..244407 | -1.083 | 8.987 | 1.430 | 0.232 | 79.09 | 900.02 | 160.26 | 283.49 |
| siRNA20 | NC_001137.3..329665..329791 | -0.556 | 10.143 | 1.339 | 0.247 | 1186.36 | 1145.48 | 801.31 | 779.58 |
| siRNA20 | NC_001136.10..806708..806821 | 1.402 | 8.280 | 1.332 | 0.248 | 158.18 | 0.00 | 320.53 | 141.74 |
| siRNA20 | NC_001134.8..652558..652675 | 1.033 | 8.671 | 1.328 | 0.249 | 158.18 | 163.64 | 400.66 | 283.49 |
| siRNA20 | NC_001148.4..138827..138953 | 0.656 | 9.702 | 1.294 | 0.255 | 711.81 | 327.28 | 881.45 | 779.58 |
| siRNA20 | NC_001144.5..43638..43757 | -0.721 | 9.451 | 1.272 | 0.259 | 553.63 | 818.20 | 240.39 | 566.97 |
| siRNA20 | NC_001143.9..360146..360262 | 1.408 | 8.285 | 1.236 | 0.266 | 0.00 | 163.64 | 400.66 | 70.87 |
| siRNA20 | NC_001143.9..130505..130628 | -0.781 | 9.140 | 1.191 | 0.275 | 553.63 | 490.92 | 240.39 | 354.36 |
| siRNA20 | NC_001145.3..546586..546708 | -0.777 | 9.143 | 1.189 | 0.275 | 474.54 | 572.74 | 320.53 | 283.49 |
| siRNA20 | NC_001134.8..764479..764590 | 1.356 | 8.272 | 1.175 | 0.278 | 0.00 | 163.64 | 80.13 | 354.36 |
| siRNA20 | NC_001138.5..62634..62761 | 0.576 | 9.831 | 1.143 | 0.285 | 711.81 | 490.92 | 961.58 | 850.46 |
| siRNA20 | NC_001139.9..753629..753746 | -1.055 | 8.609 | 1.112 | 0.292 | 79.09 | 572.74 | 160.26 | 141.74 |
| siRNA20 | NC_001144.5..284585..284693 | -1.305 | 8.189 | 1.109 | 0.292 | 316.36 | 81.82 | 80.13 | 70.87 |
| siRNA20 | NC_001146.8..718993..719112 | -1.323 | 8.186 | 1.084 | 0.298 | 316.36 | 81.82 | 0.00 | 141.74 |
| siRNA20 | NC_001139.9..212576..212764 | 0.582 | 9.663 | 1.012 | 0.314 | 632.72 | 409.10 | 641.05 | 921.33 |
| siRNA20 | NC_001134.8..184924..185043 | -0.526 | 9.869 | 1.007 | 0.316 | 949.09 | 900.02 | 641.05 | 637.84 |
| siRNA20 | NC_001145.3..799779..799898 | -0.906 | 8.739 | 0.925 | 0.336 | 632.72 | 81.82 | 160.26 | 212.61 |
| siRNA20 | NC_001148.4..898451..898570 | -0.912 | 8.748 | 0.920 | 0.338 | 79.09 | 654.56 | 240.39 | 141.74 |
| siRNA20 | NC_001148.4..249218..249443 | 0.690 | 9.178 | 0.895 | 0.344 | 474.54 | 163.64 | 400.66 | 637.84 |
| siRNA20 | NC_001146.8..617043..617162 | 1.092 | 8.457 | 0.892 | 0.345 | 0.00 | 245.46 | 480.79 | 70.87 |
| siRNA20 | NC_001144.5..1044102..1044219 | 1.140 | 8.187 | 0.888 | 0.346 | 79.09 | 81.82 | 240.39 | 141.74 |
| siRNA20 | NC_001146.8..534861..534978 | 1.146 | 8.186 | 0.850 | 0.357 | 158.18 | 0.00 | 240.39 | 141.74 |
| siRNA20 | NC_001137.3..148709..148816 | 1.137 | 8.187 | 0.836 | 0.361 | 0.00 | 163.64 | 240.39 | 141.74 |
| siRNA20 | NC_001148.4..185915..186026 | 1.137 | 8.187 | 0.836 | 0.361 | 0.00 | 163.64 | 240.39 | 141.74 |
| siRNA20 | NC_001139.9..453317..453437 | -0.733 | 8.979 | 0.821 | 0.365 | 632.72 | 245.46 | 160.26 | 354.36 |
| siRNA20 | NC_001147.6..324755..324874 | 1.117 | 8.180 | 0.812 | 0.368 | 79.09 | 81.82 | 80.13 | 283.49 |
| siRNA20 | NC_001139.9..105380..105484 | -0.869 | 8.528 | 0.807 | 0.369 | 316.36 | 245.46 | 80.13 | 212.61 |
| siRNA20 | NC_001144.5..783745..783864 | 0.434 | 10.706 | 0.792 | 0.373 | 1502.72 | 1063.66 | 2003.28 | 1488.30 |
| siRNA20 | NC_001139.9..525420..525535 | -0.865 | 8.533 | 0.781 | 0.377 | 158.18 | 409.10 | 160.26 | 141.74 |
| siRNA20 | NC_001140.6..173608..173709 | 1.120 | 8.179 | 0.776 | 0.379 | 158.18 | 0.00 | 80.13 | 283.49 |
| siRNA20 | NC_001146.8..94144..94260 | -0.859 | 8.530 | 0.775 | 0.379 | 395.45 | 163.64 | 160.26 | 141.74 |
| siRNA20 | NC_001143.9..505462..505581 | -0.691 | 9.086 | 0.709 | 0.400 | 237.27 | 736.38 | 80.13 | 496.10 |
| siRNA20 | NC_001133.9..44017..44209 | -0.405 | 10.062 | 0.667 | 0.414 | 1028.18 | 1063.66 | 641.05 | 921.33 |
| siRNA20 | NC_001133.9..33713..33827 | 1.100 | 8.176 | 0.662 | 0.416 | 158.18 | 0.00 | 0.00 | 354.36 |
| siRNA20 | NC_001147.6..708164..708281 | -1.005 | 8.090 | 0.651 | 0.420 | 158.18 | 163.64 | 80.13 | 70.87 |
| siRNA20 | NC_001136.10..32764..32881 | 0.862 | 8.369 | 0.616 | 0.433 | 79.09 | 163.64 | 320.53 | 141.74 |
| siRNA20 | NC_001145.3..174962..175081 | 0.525 | 9.231 | 0.577 | 0.447 | 237.27 | 490.92 | 480.79 | 566.97 |
| siRNA20 | NC_001136.10..96679..96794 | -0.983 | 8.092 | 0.570 | 0.450 | 237.27 | 81.82 | 160.26 | 0.00 |
| siRNA20 | NC_001142.9..102497..102609 | 0.860 | 8.370 | 0.564 | 0.453 | 0.00 | 245.46 | 320.53 | 141.74 |
| siRNA20 | NC_001134.8..236419..236652 | 0.475 | 9.319 | 0.533 | 0.465 | 395.45 | 409.10 | 480.79 | 637.84 |
| siRNA20 | NC_001136.10..39817..39937 | 0.676 | 8.525 | 0.500 | 0.479 | 158.18 | 163.64 | 240.39 | 283.49 |
| siRNA20 | NC_001136.10..1509382..1509494 | 0.676 | 8.525 | 0.500 | 0.479 | 158.18 | 163.64 | 240.39 | 283.49 |
| siRNA20 | NC_001142.9..165004..165111 | -0.786 | 8.280 | 0.493 | 0.482 | 237.27 | 163.64 | 80.13 | 141.74 |
| siRNA20 | NC_001139.9..909028..909147 | 0.702 | 8.529 | 0.450 | 0.502 | 316.36 | 0.00 | 400.66 | 141.74 |
| siRNA20 | NC_001142.9..526563..526666 | 0.702 | 8.529 | 0.450 | 0.502 | 316.36 | 0.00 | 400.66 | 141.74 |
| siRNA20 | NC_001136.10..1499123..1499245 | -0.453 | 9.242 | 0.415 | 0.519 | 632.72 | 409.10 | 560.92 | 212.61 |
| siRNA20 | NC_001138.5..128219..128338 | 0.660 | 8.518 | 0.411 | 0.521 | 237.27 | 81.82 | 80.13 | 425.23 |
| siRNA20 | NC_001148.4..863548..863649 | -0.824 | 8.280 | 0.406 | 0.524 | 0.00 | 409.10 | 0.00 | 212.61 |
| siRNA20 | NC_001148.4..383934..384045 | 0.812 | 8.084 | 0.389 | 0.533 | 0.00 | 163.64 | 80.13 | 212.61 |
| siRNA20 | NC_001143.9..540566..540686 | -0.669 | 8.445 | 0.387 | 0.534 | 316.36 | 163.64 | 0.00 | 283.49 |
| siRNA20 | NC_001140.6..304848..304969 | -0.558 | 8.602 | 0.365 | 0.546 | 395.45 | 163.64 | 160.26 | 212.61 |
| siRNA20 | NC_001148.4..792111..792230 | -0.491 | 8.741 | 0.335 | 0.563 | 395.45 | 245.46 | 240.39 | 212.61 |
| siRNA20 | NC_001146.8..66183..66300 | -0.556 | 8.601 | 0.333 | 0.564 | 474.54 | 81.82 | 160.26 | 212.61 |
| siRNA20 | NC_001137.3..514324..514444 | 0.279 | 10.032 | 0.316 | 0.574 | 870.00 | 736.38 | 961.58 | 992.20 |
| siRNA20 | NC_001224.1..35544..35667 | 0.475 | 8.800 | 0.303 | 0.582 | 395.45 | 81.82 | 320.53 | 354.36 |
| siRNA20 | NC_001143.9..42092..42211 | -0.481 | 8.743 | 0.295 | 0.587 | 474.54 | 163.64 | 320.53 | 141.74 |
| siRNA20 | NC_001143.9..409450..409569 | 0.599 | 8.277 | 0.286 | 0.593 | 158.18 | 81.82 | 160.26 | 212.61 |
| siRNA20 | NC_001141.2..151869..151988 | 0.620 | 8.284 | 0.281 | 0.596 | 79.09 | 163.64 | 320.53 | 70.87 |
| siRNA20 | NC_001137.3..334202..334320 | -0.548 | 8.612 | 0.265 | 0.606 | 0.00 | 572.74 | 320.53 | 70.87 |
| siRNA20 | NC_001134.8..15959..16071 | 0.583 | 8.275 | 0.255 | 0.613 | 79.09 | 163.64 | 80.13 | 283.49 |
| siRNA20 | NC_001140.6..85860..85981 | 0.261 | 9.900 | 0.235 | 0.628 | 632.72 | 818.20 | 1121.84 | 637.84 |
| siRNA20 | NC_001140.6..161811..161930 | -0.361 | 9.097 | 0.223 | 0.637 | 395.45 | 490.92 | 560.92 | 141.74 |
| siRNA20 | NC_001139.9..57865..58139 | -0.220 | 10.087 | 0.198 | 0.656 | 870.00 | 1145.48 | 721.18 | 992.20 |
| siRNA20 | NC_001134.8..164055..164191 | -0.335 | 9.199 | 0.197 | 0.658 | 316.36 | 654.56 | 641.05 | 141.74 |
| siRNA20 | NC_001134.8..666337..666441 | 0.380 | 8.912 | 0.192 | 0.661 | 316.36 | 245.46 | 80.13 | 637.84 |
| siRNA20 | NC_001134.8..246029..246147 | 0.378 | 8.602 | 0.173 | 0.677 | 237.27 | 163.64 | 320.53 | 212.61 |
| siRNA20 | NC_001147.6..830581..830700 | -0.470 | 8.190 | 0.163 | 0.687 | 158.18 | 163.64 | 160.26 | 70.87 |
| siRNA20 | NC_001135.5..284069..284190 | 0.374 | 8.599 | 0.162 | 0.687 | 316.36 | 81.82 | 240.39 | 283.49 |
| siRNA20 | NC_001148.4..794891..795053 | 0.191 | 10.774 | 0.161 | 0.688 | 1502.72 | 1472.76 | 1923.15 | 1488.30 |
| siRNA20 | NC_001137.3..153647..153764 | -0.473 | 8.191 | 0.158 | 0.691 | 79.09 | 245.46 | 160.26 | 70.87 |
| siRNA20 | NC_001140.6..521265..521380 | -0.473 | 8.191 | 0.158 | 0.691 | 79.09 | 245.46 | 160.26 | 70.87 |
| siRNA20 | NC_001146.8..664287..664402 | -0.466 | 8.189 | 0.154 | 0.695 | 237.27 | 81.82 | 160.26 | 70.87 |
| siRNA20 | NC_001139.9..439291..439483 | 0.194 | 9.867 | 0.135 | 0.713 | 711.81 | 736.38 | 961.58 | 708.71 |
| siRNA20 | NC_001148.4..839412..839528 | -0.354 | 8.524 | 0.131 | 0.717 | 316.36 | 163.64 | 80.13 | 283.49 |
| siRNA20 | NC_001146.8..272242..272353 | -0.391 | 8.368 | 0.130 | 0.719 | 316.36 | 81.82 | 160.26 | 141.74 |
| siRNA20 | NC_001136.10..57117..57237 | 0.303 | 8.736 | 0.128 | 0.720 | 237.27 | 245.46 | 240.39 | 354.36 |
| siRNA20 | NC_001135.5..121986..122163 | 0.176 | 10.612 | 0.127 | 0.722 | 1660.90 | 981.84 | 1282.10 | 1700.91 |
| siRNA20 | NC_001136.10..973263..973382 | -0.288 | 8.801 | 0.119 | 0.730 | 316.36 | 327.28 | 160.26 | 354.36 |
| siRNA20 | NC_001136.10..309135..309247 | -0.299 | 8.677 | 0.116 | 0.733 | 316.36 | 245.46 | 320.53 | 141.74 |
| siRNA20 | NC_001144.5..833821..833940 | -0.295 | 8.798 | 0.115 | 0.735 | 395.45 | 245.46 | 80.13 | 425.23 |
| siRNA20 | NC_001141.2..110105..110224 | -0.326 | 8.533 | 0.110 | 0.740 | 316.36 | 163.64 | 320.53 | 70.87 |
| siRNA20 | NC_001139.9..210983..211170 | -0.194 | 9.489 | 0.105 | 0.746 | 632.72 | 572.74 | 480.79 | 566.97 |
| siRNA20 | NC_001147.6..753577..753699 | 0.257 | 8.863 | 0.103 | 0.748 | 316.36 | 245.46 | 320.53 | 354.36 |
| siRNA20 | NC_001137.3..516085..516197 | 0.260 | 8.867 | 0.100 | 0.752 | 158.18 | 409.10 | 400.66 | 283.49 |
| siRNA20 | NC_001145.3..127737..127855 | -0.292 | 8.681 | 0.098 | 0.754 | 158.18 | 409.10 | 400.66 | 70.87 |
| siRNA20 | NC_001147.6..551863..551981 | -0.327 | 8.529 | 0.096 | 0.757 | 474.54 | 0.00 | 240.39 | 141.74 |
| siRNA20 | NC_001145.3..905136..905255 | -0.256 | 8.812 | 0.084 | 0.772 | 395.45 | 245.46 | 480.79 | 70.87 |
| siRNA20 | NC_001148.4..729095..729212 | 0.297 | 8.187 | 0.065 | 0.799 | 79.09 | 163.64 | 160.26 | 141.74 |
| siRNA20 | NC_001140.6..247875..247994 | 0.182 | 9.084 | 0.062 | 0.804 | 316.36 | 409.10 | 320.53 | 496.10 |
| siRNA20 | NC_001140.6..444655..444773 | 0.288 | 8.183 | 0.059 | 0.808 | 158.18 | 81.82 | 80.13 | 212.61 |
| siRNA20 | NC_001143.9..191425..191544 | 0.292 | 8.188 | 0.057 | 0.811 | 0.00 | 245.46 | 160.26 | 141.74 |
| siRNA20 | NC_001148.4..289781..289893 | 0.292 | 8.188 | 0.057 | 0.811 | 0.00 | 245.46 | 160.26 | 141.74 |
| siRNA20 | NC_001145.3..282562..282674 | 0.276 | 8.185 | 0.050 | 0.823 | 0.00 | 245.46 | 80.13 | 212.61 |
| siRNA20 | NC_001136.10..814138..814257 | 0.237 | 8.371 | 0.045 | 0.832 | 237.27 | 81.82 | 320.53 | 70.87 |
| siRNA20 | NC_001136.10..465632..465749 | 0.230 | 8.373 | 0.042 | 0.837 | 79.09 | 245.46 | 320.53 | 70.87 |
| siRNA20 | NC_001148.4..458393..458504 | 0.253 | 8.181 | 0.038 | 0.846 | 0.00 | 245.46 | 0.00 | 283.49 |
| siRNA20 | NC_001136.10..227955..228251 | -0.086 | 11.765 | 0.036 | 0.850 | 2926.35 | 3927.35 | 3044.99 | 3401.83 |
| siRNA20 | NC_001134.8..240187..240310 | 0.107 | 9.450 | 0.031 | 0.860 | 474.54 | 572.74 | 560.92 | 566.97 |
| siRNA20 | NC_001142.9..429935..430046 | 0.169 | 8.531 | 0.031 | 0.860 | 237.27 | 163.64 | 320.53 | 141.74 |
| siRNA20 | NC_001136.10..443136..443258 | -0.106 | 9.359 | 0.024 | 0.877 | 711.81 | 327.28 | 240.39 | 708.71 |
| siRNA20 | NC_001145.3..627421..627547 | 0.070 | 10.604 | 0.021 | 0.885 | 1502.72 | 1227.30 | 1602.63 | 1275.68 |
| siRNA20 | NC_001147.6..1018197..1018316 | 0.118 | 8.672 | 0.018 | 0.893 | 158.18 | 327.28 | 240.39 | 283.49 |
| siRNA20 | NC_001139.9..867742..867866 | -0.098 | 8.740 | 0.013 | 0.910 | 158.18 | 409.10 | 240.39 | 283.49 |
| siRNA20 | NC_001137.3..114552..114674 | 0.089 | 8.801 | 0.012 | 0.914 | 316.36 | 245.46 | 240.39 | 354.36 |
| siRNA20 | NC_001137.3..319918..320037 | -0.086 | 8.607 | 0.009 | 0.925 | 158.18 | 327.28 | 320.53 | 141.74 |
| siRNA20 | NC_001145.3..871011..871129 | -0.085 | 8.606 | 0.009 | 0.925 | 237.27 | 245.46 | 320.53 | 141.74 |
| siRNA20 | NC_001141.2..82970..83089 | -0.101 | 8.278 | 0.008 | 0.928 | 158.18 | 163.64 | 80.13 | 212.61 |
| siRNA20 | NC_001140.6..189548..189666 | -0.099 | 8.277 | 0.007 | 0.931 | 237.27 | 81.82 | 80.13 | 212.61 |
| siRNA20 | NC_001140.6..60347..60458 | -0.109 | 8.082 | 0.006 | 0.936 | 237.27 | 0.00 | 0.00 | 212.61 |
| siRNA20 | NC_001134.8..415226..415329 | -0.096 | 8.087 | 0.006 | 0.938 | 79.09 | 163.64 | 80.13 | 141.74 |
| siRNA20 | NC_001141.2..179854..179955 | -0.096 | 8.087 | 0.006 | 0.938 | 79.09 | 163.64 | 80.13 | 141.74 |
| siRNA20 | NC_001142.9..575503..575612 | -0.087 | 8.280 | 0.006 | 0.938 | 237.27 | 81.82 | 160.26 | 141.74 |
| siRNA20 | NC_001145.3..752820..752927 | -0.080 | 8.089 | 0.004 | 0.948 | 158.18 | 81.82 | 160.26 | 70.87 |
| siRNA20 | NC_001137.3..401591..401747 | 0.059 | 8.792 | 0.004 | 0.949 | 316.36 | 245.46 | 0.00 | 566.97 |
| siRNA20 | NC_001145.3..123564..123782 | 0.029 | 11.664 | 0.004 | 0.951 | 2847.26 | 3272.79 | 2323.81 | 3897.92 |
| siRNA20 | NC_001134.8..80263..80377 | -0.061 | 8.288 | 0.003 | 0.959 | 79.09 | 245.46 | 320.53 | 0.00 |
| siRNA21 | NC_001139.9..250941..251060 | -5.261 | 8.377 | 10.254 | 0.001 | 237.27 | 490.92 | 0.00 | 0.00 |
| siRNA21 | NC_001138.5..69579..69691 | 4.819 | 8.183 | 7.637 | 0.006 | 0.00 | 0.00 | 320.53 | 212.61 |
| siRNA21 | NC_001147.6..481522..481640 | 3.097 | 8.599 | 7.123 | 0.008 | 79.09 | 0.00 | 560.92 | 283.49 |
| siRNA21 | NC_001134.8..800434..800553 | 4.600 | 8.083 | 6.690 | 0.010 | 0.00 | 0.00 | 240.39 | 212.61 |
| siRNA21 | NC_001147.6..826114..826233 | 4.612 | 8.086 | 6.558 | 0.010 | 0.00 | 0.00 | 320.53 | 141.74 |
| siRNA21 | NC_001137.3..456457..456576 | -2.615 | 8.288 | 4.168 | 0.041 | 237.27 | 327.28 | 80.13 | 0.00 |
| siRNA21 | NC_001142.9..347792..347965 | -1.620 | 9.094 | 3.697 | 0.055 | 949.09 | 245.46 | 240.39 | 141.74 |
| siRNA21 | NC_001137.3..158886..159094 | -1.532 | 9.039 | 3.518 | 0.061 | 790.90 | 327.28 | 160.26 | 212.61 |
| siRNA21 | NC_001142.9..398906..399008 | 2.467 | 8.281 | 3.438 | 0.064 | 0.00 | 81.82 | 400.66 | 141.74 |
| siRNA21 | NC_001143.9..35415..35535 | 1.799 | 8.786 | 3.363 | 0.067 | 158.18 | 81.82 | 160.26 | 708.71 |
| siRNA21 | NC_001139.9..451924..452026 | 2.420 | 8.268 | 3.124 | 0.077 | 0.00 | 81.82 | 80.13 | 425.23 |
| siRNA21 | NC_001141.2..72234..72357 | 0.860 | 10.207 | 2.982 | 0.084 | 790.90 | 654.56 | 1522.50 | 1133.94 |
| siRNA21 | NC_001134.8..82432..82556 | 1.424 | 8.855 | 2.655 | 0.103 | 79.09 | 245.46 | 320.53 | 566.97 |
| siRNA21 | NC_001142.9..639663..639779 | -2.164 | 8.090 | 2.525 | 0.112 | 158.18 | 245.46 | 0.00 | 70.87 |
| siRNA21 | NC_001142.9..293778..293900 | -1.368 | 8.742 | 2.407 | 0.121 | 395.45 | 409.10 | 80.13 | 212.61 |
| siRNA21 | NC_001144.5..180819..180930 | -2.159 | 8.088 | 2.382 | 0.123 | 316.36 | 81.82 | 0.00 | 70.87 |
| siRNA21 | NC_001148.4..130504..130614 | -1.758 | 8.374 | 2.230 | 0.135 | 395.45 | 163.64 | 160.26 | 0.00 |
| siRNA21 | NC_001136.10..1476441..1476558 | -1.785 | 8.375 | 2.201 | 0.138 | 79.09 | 490.92 | 80.13 | 70.87 |
| siRNA21 | NC_001133.9..69194..69347 | 0.896 | 9.634 | 2.187 | 0.139 | 632.72 | 245.46 | 961.58 | 708.71 |
| siRNA21 | NC_001142.9..113058..113170 | 1.980 | 8.083 | 2.086 | 0.149 | 0.00 | 81.82 | 160.26 | 212.61 |
| siRNA21 | NC_001148.4..265267..265387 | -1.435 | 8.530 | 1.973 | 0.160 | 474.54 | 163.64 | 80.13 | 141.74 |
| siRNA21 | NC_001144.5..680814..680945 | 0.613 | 13.571 | 1.868 | 0.172 | 9411.76 | 9572.91 | 13381.94 | 15662.57 |
| siRNA21 | NC_001140.6..287749..287866 | -1.220 | 8.674 | 1.838 | 0.175 | 395.45 | 327.28 | 80.13 | 212.61 |
| siRNA21 | NC_001133.9..199861..199984 | -1.171 | 8.880 | 1.602 | 0.206 | 237.27 | 654.56 | 400.66 | 0.00 |
| siRNA21 | NC_001134.8..234986..235101 | 1.267 | 8.527 | 1.489 | 0.222 | 237.27 | 0.00 | 400.66 | 212.61 |
| siRNA21 | NC_001147.6..883545..883658 | -1.047 | 8.813 | 1.474 | 0.225 | 395.45 | 409.10 | 320.53 | 70.87 |
| siRNA21 | NC_001147.6..620979..621178 | -0.815 | 9.288 | 1.398 | 0.237 | 395.45 | 818.20 | 400.66 | 283.49 |
| siRNA21 | NC_001137.3..286530..286650 | 0.876 | 9.128 | 1.378 | 0.240 | 395.45 | 163.64 | 400.66 | 637.84 |
| siRNA21 | NC_001144.5..727683..727797 | -1.229 | 8.458 | 1.316 | 0.251 | 316.36 | 245.46 | 240.39 | 0.00 |
| siRNA21 | NC_001147.6..377048..377167 | -1.247 | 8.450 | 1.291 | 0.256 | 474.54 | 81.82 | 80.13 | 141.74 |
| siRNA21 | NC_001136.10..1490952..1491072 | -1.070 | 8.601 | 1.196 | 0.274 | 237.27 | 409.10 | 0.00 | 283.49 |
| siRNA21 | NC_001148.4..31722..31841 | 1.071 | 8.449 | 1.060 | 0.303 | 79.09 | 163.64 | 320.53 | 212.61 |
| siRNA21 | NC_001139.9..116399..116517 | 1.065 | 8.446 | 1.058 | 0.304 | 158.18 | 81.82 | 240.39 | 283.49 |
| siRNA21 | NC_001142.9..643550..643670 | 1.057 | 8.442 | 1.004 | 0.316 | 158.18 | 81.82 | 160.26 | 354.36 |
| siRNA21 | NC_001148.4..94956..95066 | -1.300 | 8.188 | 0.971 | 0.324 | 395.45 | 0.00 | 80.13 | 70.87 |
| siRNA21 | NC_001136.10..301367..301520 | -0.628 | 9.409 | 0.966 | 0.326 | 790.90 | 490.92 | 320.53 | 496.10 |
| siRNA21 | NC_001136.10..1006364..1006491 | -0.555 | 9.571 | 0.896 | 0.344 | 711.81 | 736.38 | 560.92 | 425.23 |
| siRNA21 | NC_001137.3..329958..330063 | 1.130 | 8.183 | 0.879 | 0.349 | 79.09 | 81.82 | 160.26 | 212.61 |
| siRNA21 | NC_001137.3..197205..197327 | -0.790 | 8.874 | 0.862 | 0.353 | 474.54 | 327.28 | 400.66 | 70.87 |
| siRNA21 | NC_001142.9..624181..624301 | 0.750 | 8.922 | 0.825 | 0.364 | 79.09 | 409.10 | 400.66 | 425.23 |
| siRNA21 | NC_001140.6..430118..430304 | 0.619 | 9.272 | 0.822 | 0.365 | 316.36 | 409.10 | 400.66 | 708.71 |
| siRNA21 | NC_001144.5..205393..205513 | -0.869 | 8.528 | 0.807 | 0.369 | 316.36 | 245.46 | 80.13 | 212.61 |
| siRNA21 | NC_001146.8..117333..117501 | 0.668 | 9.036 | 0.766 | 0.382 | 237.27 | 327.28 | 560.92 | 354.36 |
| siRNA21 | NC_001143.9..441003..441123 | 0.745 | 9.216 | 0.742 | 0.389 | 158.18 | 490.92 | 80.13 | 992.20 |
| siRNA21 | NC_001139.9..1064729..1064896 | -0.532 | 9.374 | 0.686 | 0.408 | 474.54 | 736.38 | 480.79 | 354.36 |
| siRNA21 | NC_001139.9..58244..58364 | 0.718 | 8.733 | 0.683 | 0.408 | 237.27 | 163.64 | 240.39 | 425.23 |
| siRNA21 | NC_001136.10..711854..711968 | -1.021 | 8.086 | 0.639 | 0.424 | 158.18 | 163.64 | 0.00 | 141.74 |
| siRNA21 | NC_001145.3..10108..10228 | -0.665 | 8.805 | 0.638 | 0.424 | 316.36 | 409.10 | 160.26 | 283.49 |
| siRNA21 | NC_001147.6..58795..58905 | -1.009 | 8.091 | 0.631 | 0.427 | 79.09 | 245.46 | 80.13 | 70.87 |
| siRNA21 | NC_001142.9..621121..621226 | 0.866 | 8.369 | 0.621 | 0.431 | 158.18 | 81.82 | 320.53 | 141.74 |
| siRNA21 | NC_001148.4..718289..718406 | 0.862 | 8.369 | 0.616 | 0.433 | 79.09 | 163.64 | 320.53 | 141.74 |
| siRNA21 | NC_001137.3..547114..547242 | 0.372 | 10.142 | 0.553 | 0.457 | 711.81 | 981.84 | 1362.23 | 850.46 |
| siRNA21 | NC_001139.9..896749..896872 | 0.480 | 9.325 | 0.514 | 0.473 | 237.27 | 572.74 | 641.05 | 496.10 |
| siRNA21 | NC_001139.9..227820..228022 | -0.357 | 10.978 | 0.495 | 0.482 | 1581.81 | 2618.23 | 1121.84 | 2126.14 |
| siRNA21 | NC_001134.8..235260..235461 | 0.440 | 9.410 | 0.488 | 0.485 | 474.54 | 409.10 | 721.18 | 496.10 |
| siRNA21 | NC_001144.5..753417..753532 | -0.783 | 8.280 | 0.460 | 0.498 | 316.36 | 81.82 | 80.13 | 141.74 |
| siRNA21 | NC_001140.6..339510..339630 | 0.354 | 10.010 | 0.446 | 0.504 | 474.54 | 1063.66 | 1121.84 | 850.46 |
| siRNA21 | NC_001145.3..613043..613158 | 0.835 | 8.085 | 0.424 | 0.515 | 158.18 | 0.00 | 160.26 | 141.74 |
| siRNA21 | NC_001134.8..607015..607139 | 0.377 | 9.729 | 0.422 | 0.516 | 790.90 | 409.10 | 560.92 | 992.20 |
| siRNA21 | NC_001134.8..391826..391941 | -0.655 | 8.449 | 0.409 | 0.523 | 316.36 | 163.64 | 80.13 | 212.61 |
| siRNA21 | NC_001141.2..367220..367324 | 0.556 | 8.668 | 0.400 | 0.527 | 237.27 | 163.64 | 240.39 | 354.36 |
| siRNA21 | NC_001138.5..188012..188125 | -0.634 | 8.455 | 0.381 | 0.537 | 316.36 | 163.64 | 240.39 | 70.87 |
| siRNA21 | NC_001134.8..193994..194115 | -0.399 | 9.416 | 0.368 | 0.544 | 790.90 | 409.10 | 641.05 | 283.49 |
| siRNA21 | NC_001147.6..918948..919114 | 0.323 | 9.929 | 0.360 | 0.549 | 632.72 | 818.20 | 1201.97 | 637.84 |
| siRNA21 | NC_001142.9..99566..99683 | 0.554 | 8.673 | 0.342 | 0.559 | 0.00 | 409.10 | 320.53 | 283.49 |
| siRNA21 | NC_001133.9..141094..141467 | -0.248 | 12.432 | 0.315 | 0.575 | 5536.33 | 6136.48 | 4647.62 | 5173.61 |
| siRNA21 | NC_001143.9..384451..384573 | -0.392 | 9.085 | 0.263 | 0.608 | 316.36 | 572.74 | 160.26 | 496.10 |
| siRNA21 | NC_001143.9..155809..155929 | 0.426 | 8.932 | 0.244 | 0.621 | 158.18 | 409.10 | 641.05 | 141.74 |
| siRNA21 | NC_001143.9..243740..243856 | 0.575 | 8.276 | 0.227 | 0.634 | 0.00 | 245.46 | 80.13 | 283.49 |
| siRNA21 | NC_001139.9..474160..474287 | 0.388 | 8.915 | 0.219 | 0.640 | 237.27 | 327.28 | 160.26 | 566.97 |
| siRNA21 | NC_001142.9..424860..424980 | 0.569 | 8.271 | 0.215 | 0.643 | 158.18 | 81.82 | 0.00 | 354.36 |
| siRNA21 | NC_001136.10..315681..315789 | 0.469 | 8.448 | 0.212 | 0.645 | 237.27 | 81.82 | 240.39 | 212.61 |
| siRNA21 | NC_001139.9..274976..275085 | -0.482 | 8.187 | 0.172 | 0.679 | 158.18 | 163.64 | 80.13 | 141.74 |
| siRNA21 | NC_001148.4..376513..376625 | -0.482 | 8.187 | 0.172 | 0.679 | 158.18 | 163.64 | 80.13 | 141.74 |
| siRNA21 | NC_001136.10..71539..71652 | -0.486 | 8.188 | 0.167 | 0.682 | 79.09 | 245.46 | 80.13 | 141.74 |
| siRNA21 | NC_001139.9..1026560..1026675 | 0.370 | 8.600 | 0.167 | 0.683 | 158.18 | 245.46 | 240.39 | 283.49 |
| siRNA21 | NC_001146.8..284027..284155 | 0.381 | 8.602 | 0.167 | 0.683 | 316.36 | 81.82 | 320.53 | 212.61 |
| siRNA21 | NC_001142.9..147131..147235 | -0.479 | 8.186 | 0.164 | 0.686 | 237.27 | 81.82 | 80.13 | 141.74 |
| siRNA21 | NC_001147.6..1016632..1016749 | -0.517 | 8.185 | 0.153 | 0.696 | 0.00 | 327.28 | 0.00 | 212.61 |
| siRNA21 | NC_001136.10..1444969..1445186 | 0.193 | 10.169 | 0.148 | 0.701 | 790.90 | 1063.66 | 1362.23 | 779.58 |
| siRNA21 | NC_001139.9..128118..128238 | 0.263 | 9.229 | 0.138 | 0.711 | 237.27 | 572.74 | 320.53 | 637.84 |
| siRNA21 | NC_001145.3..318946..319064 | -0.452 | 8.193 | 0.136 | 0.712 | 158.18 | 163.64 | 240.39 | 0.00 |
| siRNA21 | NC_001143.9..575200..575317 | -0.347 | 8.528 | 0.135 | 0.713 | 237.27 | 245.46 | 160.26 | 212.61 |
| siRNA21 | NC_001144.5..399567..399687 | -0.358 | 8.526 | 0.134 | 0.714 | 158.18 | 327.28 | 80.13 | 283.49 |
| siRNA21 | NC_001143.9..76121..76233 | -0.339 | 8.531 | 0.129 | 0.719 | 237.27 | 245.46 | 240.39 | 141.74 |
| siRNA21 | NC_001143.9..75727..75847 | -0.337 | 8.530 | 0.124 | 0.724 | 316.36 | 163.64 | 240.39 | 141.74 |
| siRNA21 | NC_001224.1..73414..73534 | 0.338 | 8.742 | 0.120 | 0.729 | 474.54 | 0.00 | 480.79 | 141.74 |
| siRNA21 | NC_001145.3..464058..464252 | -0.262 | 8.923 | 0.113 | 0.737 | 395.45 | 327.28 | 240.39 | 354.36 |
| siRNA21 | NC_001140.6..125515..125635 | -0.252 | 8.928 | 0.103 | 0.749 | 395.45 | 327.28 | 400.66 | 212.61 |
| siRNA21 | NC_001140.6..282689..282810 | 0.256 | 8.864 | 0.102 | 0.749 | 237.27 | 327.28 | 320.53 | 354.36 |
| siRNA21 | NC_001148.4..207928..208068 | -0.244 | 9.030 | 0.085 | 0.771 | 711.81 | 81.82 | 240.39 | 425.23 |
| siRNA21 | NC_001145.3..290995..291111 | -0.242 | 8.926 | 0.079 | 0.779 | 632.72 | 81.82 | 400.66 | 212.61 |
| siRNA21 | NC_001143.9..185463..185584 | -0.212 | 9.144 | 0.079 | 0.779 | 632.72 | 245.46 | 560.92 | 212.61 |
| siRNA21 | NC_001136.10..325959..326073 | 0.300 | 8.186 | 0.066 | 0.797 | 158.18 | 81.82 | 160.26 | 141.74 |
| siRNA21 | NC_001146.8..384593..384704 | 0.305 | 8.185 | 0.062 | 0.803 | 237.27 | 0.00 | 160.26 | 141.74 |
| siRNA21 | NC_001138.5..128676..128784 | 0.288 | 8.183 | 0.059 | 0.808 | 158.18 | 81.82 | 80.13 | 212.61 |
| siRNA21 | NC_001147.6..1001735..1001853 | 0.284 | 8.184 | 0.058 | 0.810 | 79.09 | 163.64 | 80.13 | 212.61 |
| siRNA21 | NC_001146.8..518496..518829 | -0.108 | 10.363 | 0.047 | 0.828 | 1028.18 | 1390.94 | 801.31 | 1417.43 |
| siRNA21 | NC_001134.8..294328..294445 | 0.232 | 8.372 | 0.045 | 0.833 | 158.18 | 163.64 | 320.53 | 70.87 |
| siRNA21 | NC_001146.8..27049..27168 | 0.248 | 8.376 | 0.044 | 0.835 | 79.09 | 245.46 | 400.66 | 0.00 |
| siRNA21 | NC_001143.9..74581..74750 | 0.093 | 16.596 | 0.042 | 0.838 | 91982.20 | 99492.85 | 100003.97 | 104251.77 |
| siRNA21 | NC_001143.9..178838..178955 | 0.210 | 8.366 | 0.040 | 0.842 | 158.18 | 163.64 | 160.26 | 212.61 |
| siRNA21 | NC_001139.9..414073..414183 | 0.207 | 8.367 | 0.037 | 0.847 | 79.09 | 245.46 | 160.26 | 212.61 |
| siRNA21 | NC_001146.8..650657..650776 | 0.201 | 8.362 | 0.034 | 0.855 | 237.27 | 81.82 | 80.13 | 283.49 |
| siRNA21 | NC_001138.5..232310..232427 | 0.181 | 8.534 | 0.033 | 0.857 | 237.27 | 163.64 | 400.66 | 70.87 |
| siRNA21 | NC_001141.2..420045..420165 | 0.181 | 8.534 | 0.033 | 0.857 | 237.27 | 163.64 | 400.66 | 70.87 |
| siRNA21 | NC_001147.6..938168..938286 | 0.161 | 8.528 | 0.029 | 0.864 | 237.27 | 163.64 | 240.39 | 212.61 |
| siRNA21 | NC_001139.9..479526..479720 | 0.072 | 10.786 | 0.023 | 0.880 | 1423.63 | 1718.22 | 1522.50 | 1771.78 |
| siRNA21 | NC_001135.5..283506..283630 | -0.106 | 8.975 | 0.018 | 0.893 | 395.45 | 327.28 | 160.26 | 496.10 |
| siRNA21 | NC_001148.4..577996..578118 | 0.113 | 8.668 | 0.017 | 0.897 | 237.27 | 245.46 | 160.26 | 354.36 |
| siRNA21 | NC_001135.5..263994..264114 | -0.093 | 8.866 | 0.014 | 0.907 | 316.36 | 327.28 | 320.53 | 283.49 |
| siRNA21 | NC_001145.3..819179..819343 | 0.095 | 8.804 | 0.013 | 0.908 | 316.36 | 245.46 | 320.53 | 283.49 |
| siRNA21 | NC_001135.5..26594..26714 | -0.093 | 8.738 | 0.012 | 0.914 | 395.45 | 163.64 | 240.39 | 283.49 |
| siRNA21 | NC_001144.5..501321..501439 | -0.106 | 8.278 | 0.009 | 0.927 | 79.09 | 245.46 | 80.13 | 212.61 |
| siRNA21 | NC_001146.8..48423..48536 | -0.088 | 8.453 | 0.008 | 0.930 | 158.18 | 245.46 | 240.39 | 141.74 |
| siRNA21 | NC_001145.3..133985..134101 | -0.093 | 8.282 | 0.007 | 0.934 | 79.09 | 245.46 | 160.26 | 141.74 |
| siRNA21 | NC_001140.6..316654..316771 | -0.090 | 8.281 | 0.007 | 0.935 | 158.18 | 163.64 | 160.26 | 141.74 |
| siRNA21 | NC_001145.3..699891..699992 | -0.086 | 8.091 | 0.004 | 0.947 | 0.00 | 245.46 | 160.26 | 70.87 |
| siRNA21 | NC_001136.10..93294..93408 | -0.073 | 8.283 | 0.004 | 0.948 | 237.27 | 81.82 | 240.39 | 70.87 |
| siRNA21 | NC_001139.9..674887..675009 | 0.043 | 9.140 | 0.004 | 0.951 | 395.45 | 409.10 | 480.79 | 354.36 |
| siRNA21 | NC_001139.9..761560..761684 | 0.039 | 9.027 | 0.002 | 0.960 | 395.45 | 327.28 | 160.26 | 566.97 |
| siRNA21 | NC_001138.5..141261..141363 | -0.060 | 8.093 | 0.002 | 0.962 | 158.18 | 81.82 | 240.39 | 0.00 |
| siRNA21 | NC_001145.3..431165..431275 | -0.060 | 8.093 | 0.002 | 0.962 | 158.18 | 81.82 | 240.39 | 0.00 |
| siRNA21 | NC_001144.5..50416..50538 | 0.031 | 9.136 | 0.002 | 0.966 | 237.27 | 572.74 | 320.53 | 496.10 |
| siRNA21 | NC_001140.6..226786..226908 | 0.028 | 9.231 | 0.002 | 0.967 | 632.72 | 245.46 | 400.66 | 496.10 |
| siRNA21 | NC_001134.8..775411..775533 | 0.008 | 9.408 | 0.000 | 0.990 | 474.54 | 572.74 | 480.79 | 566.97 |
| siRNA22 | NC_001144.5..978537..978785 | -2.665 | 9.756 | 17.287 | 0.000 | 1344.54 | 1145.48 | 240.39 | 141.74 |
| siRNA22 | NC_001224.1..23001..23125 | -1.286 | 10.227 | 6.366 | 0.012 | 1423.63 | 1554.58 | 801.31 | 425.23 |
| siRNA22 | NC_001143.9..65755..65856 | -2.794 | 8.374 | 4.716 | 0.030 | 474.54 | 163.64 | 80.13 | 0.00 |
| siRNA22 | NC_001139.9..987172..987293 | -2.629 | 8.285 | 4.219 | 0.040 | 237.27 | 327.28 | 0.00 | 70.87 |
| siRNA22 | NC_001143.9..174692..174829 | -1.729 | 8.936 | 3.913 | 0.048 | 316.36 | 736.38 | 240.39 | 70.87 |
| siRNA22 | NC_001139.9..397171..397277 | -2.148 | 8.534 | 3.726 | 0.054 | 158.18 | 572.74 | 0.00 | 141.74 |
| siRNA22 | NC_001141.2..231550..231663 | -2.136 | 8.529 | 3.714 | 0.054 | 553.63 | 163.64 | 0.00 | 141.74 |
| siRNA22 | NC_001140.6..294780..294897 | -1.893 | 8.740 | 3.511 | 0.061 | 711.81 | 163.64 | 0.00 | 212.61 |
| siRNA22 | NC_001135.5..14582..14701 | -1.973 | 8.452 | 3.177 | 0.075 | 395.45 | 245.46 | 0.00 | 141.74 |
| siRNA22 | NC_001144.5..819248..819366 | 2.426 | 8.267 | 3.139 | 0.076 | 79.09 | 0.00 | 80.13 | 425.23 |
| siRNA22 | NC_001144.5..311024..311131 | 2.420 | 8.268 | 3.124 | 0.077 | 0.00 | 81.82 | 80.13 | 425.23 |
| siRNA22 | NC_001142.9..446401..446523 | -1.494 | 8.809 | 2.940 | 0.086 | 553.63 | 327.28 | 160.26 | 141.74 |
| siRNA22 | NC_001134.8..760565..760687 | 1.431 | 8.857 | 2.837 | 0.092 | 158.18 | 163.64 | 400.66 | 496.10 |
| siRNA22 | NC_001138.5..132745..132864 | -1.513 | 8.805 | 2.814 | 0.093 | 395.45 | 490.92 | 0.00 | 283.49 |
| siRNA22 | NC_001144.5..95527..95645 | 1.433 | 8.857 | 2.784 | 0.095 | 237.27 | 81.82 | 400.66 | 496.10 |
| siRNA22 | NC_001138.5..99103..99217 | 2.230 | 8.180 | 2.775 | 0.096 | 79.09 | 0.00 | 160.26 | 283.49 |
| siRNA22 | NC_001146.8..112405..112520 | -2.159 | 8.088 | 2.382 | 0.123 | 316.36 | 81.82 | 0.00 | 70.87 |
| siRNA22 | NC_001147.6..718257..718379 | -0.926 | 9.457 | 2.244 | 0.134 | 711.81 | 736.38 | 400.66 | 354.36 |
| siRNA22 | NC_001137.3..260849..260964 | -1.750 | 8.373 | 2.038 | 0.153 | 474.54 | 81.82 | 160.26 | 0.00 |
| siRNA22 | NC_001144.5..278124..278240 | 1.970 | 8.079 | 1.967 | 0.161 | 79.09 | 0.00 | 80.13 | 283.49 |
| siRNA22 | NC_001143.9..249576..249697 | 1.630 | 8.372 | 1.795 | 0.180 | 79.09 | 81.82 | 480.79 | 70.87 |
| siRNA22 | NC_001147.6..778526..778649 | 0.819 | 9.591 | 1.775 | 0.183 | 632.72 | 245.46 | 641.05 | 921.33 |
| siRNA22 | NC_001135.5..61082..61201 | 1.126 | 8.922 | 1.678 | 0.195 | 0.00 | 409.10 | 480.79 | 425.23 |
| siRNA22 | NC_001148.4..886763..886886 | 0.816 | 9.488 | 1.665 | 0.197 | 237.27 | 572.74 | 801.31 | 637.84 |
| siRNA22 | NC_001137.3..129917..130040 | 0.804 | 9.359 | 1.423 | 0.233 | 553.63 | 163.64 | 560.92 | 708.71 |
| siRNA22 | NC_001144.5..750966..751085 | 1.376 | 8.274 | 1.373 | 0.241 | 79.09 | 81.82 | 160.26 | 283.49 |
| siRNA22 | NC_001145.3..435282..435397 | -1.262 | 8.455 | 1.311 | 0.252 | 79.09 | 490.92 | 80.13 | 141.74 |
| siRNA22 | NC_001146.8..63697..63802 | 1.026 | 8.669 | 1.287 | 0.257 | 79.09 | 245.46 | 320.53 | 354.36 |
| siRNA22 | NC_001137.3..181214..181335 | -1.232 | 8.460 | 1.260 | 0.262 | 158.18 | 409.10 | 240.39 | 0.00 |
| siRNA22 | NC_001134.8..661931..662051 | 1.016 | 8.661 | 1.154 | 0.283 | 237.27 | 81.82 | 160.26 | 496.10 |
| siRNA22 | NC_001146.8..666064..666181 | -1.035 | 8.607 | 1.146 | 0.284 | 474.54 | 163.64 | 240.39 | 70.87 |
| siRNA22 | NC_001135.5..227669..227788 | -1.305 | 8.189 | 1.109 | 0.292 | 316.36 | 81.82 | 80.13 | 70.87 |
| siRNA22 | NC_001137.3..267818..267920 | -1.305 | 8.189 | 1.109 | 0.292 | 316.36 | 81.82 | 80.13 | 70.87 |
| siRNA22 | NC_001139.9..922274..922392 | 1.344 | 8.268 | 1.069 | 0.301 | 79.09 | 81.82 | 0.00 | 425.23 |
| siRNA22 | NC_001137.3..339396..339520 | 0.501 | 10.618 | 1.039 | 0.308 | 870.00 | 1472.76 | 1762.89 | 1559.17 |
| siRNA22 | NC_001144.5..295754..295871 | 1.093 | 8.456 | 0.962 | 0.327 | 79.09 | 163.64 | 480.79 | 70.87 |
| siRNA22 | NC_001146.8..511656..511778 | 0.617 | 9.493 | 0.910 | 0.340 | 553.63 | 327.28 | 961.58 | 425.23 |
| siRNA22 | NC_001136.10..750836..750957 | 0.689 | 9.183 | 0.909 | 0.340 | 158.18 | 490.92 | 480.79 | 566.97 |
| siRNA22 | NC_001144.5..512540..512658 | -1.037 | 8.368 | 0.908 | 0.341 | 316.36 | 163.64 | 80.13 | 141.74 |
| siRNA22 | NC_001134.8..626188..626381 | -0.668 | 9.089 | 0.834 | 0.361 | 474.54 | 490.92 | 240.39 | 354.36 |
| siRNA22 | NC_001138.5..33472..33594 | 0.661 | 9.030 | 0.763 | 0.382 | 316.36 | 245.46 | 400.66 | 496.10 |
| siRNA22 | NC_001147.6..897042..897151 | -0.855 | 8.536 | 0.740 | 0.390 | 158.18 | 409.10 | 240.39 | 70.87 |
| siRNA22 | NC_001144.5..613132..613257 | 0.593 | 9.137 | 0.684 | 0.408 | 316.36 | 327.28 | 560.92 | 425.23 |
| siRNA22 | NC_001140.6..515475..515596 | 0.743 | 8.745 | 0.659 | 0.417 | 158.18 | 245.46 | 560.92 | 141.74 |
| siRNA22 | NC_001141.2..124764..124890 | -0.500 | 9.453 | 0.648 | 0.421 | 790.90 | 490.92 | 480.79 | 425.23 |
| siRNA22 | NC_001148.4..333504..333624 | -0.659 | 8.807 | 0.636 | 0.425 | 316.36 | 409.10 | 240.39 | 212.61 |
| siRNA22 | NC_001148.4..105418..105535 | 0.843 | 8.363 | 0.598 | 0.439 | 79.09 | 163.64 | 160.26 | 283.49 |
| siRNA22 | NC_001139.9..978873..978992 | 0.618 | 8.860 | 0.571 | 0.450 | 316.36 | 163.64 | 320.53 | 425.23 |
| siRNA22 | NC_001144.5..741758..741877 | 0.849 | 8.367 | 0.568 | 0.451 | 0.00 | 245.46 | 240.39 | 212.61 |
| siRNA22 | NC_001148.4..370062..370170 | -1.038 | 8.088 | 0.566 | 0.452 | 0.00 | 327.28 | 0.00 | 141.74 |
| siRNA22 | NC_001145.3..429224..429347 | 0.442 | 9.409 | 0.480 | 0.488 | 553.63 | 327.28 | 721.18 | 496.10 |
| siRNA22 | NC_001143.9..233573..233755 | -0.434 | 9.330 | 0.447 | 0.504 | 474.54 | 654.56 | 480.79 | 354.36 |
| siRNA22 | NC_001145.3..472882..472984 | 0.830 | 8.086 | 0.441 | 0.507 | 79.09 | 81.82 | 160.26 | 141.74 |
| siRNA22 | NC_001134.8..575819..575940 | -0.657 | 8.449 | 0.420 | 0.517 | 237.27 | 245.46 | 80.13 | 212.61 |
| siRNA22 | NC_001146.8..132093..132202 | -0.801 | 8.283 | 0.419 | 0.518 | 0.00 | 409.10 | 80.13 | 141.74 |
| siRNA22 | NC_001148.4..392848..392964 | -0.634 | 8.455 | 0.381 | 0.537 | 316.36 | 163.64 | 240.39 | 70.87 |
| siRNA22 | NC_001147.6..823858..823974 | 0.476 | 8.804 | 0.329 | 0.566 | 237.27 | 245.46 | 400.66 | 283.49 |
| siRNA22 | NC_001134.8..268993..269111 | -0.547 | 8.610 | 0.328 | 0.567 | 158.18 | 409.10 | 320.53 | 70.87 |
| siRNA22 | NC_001143.9..515951..516072 | 0.374 | 9.275 | 0.307 | 0.579 | 316.36 | 490.92 | 400.66 | 637.84 |
| siRNA22 | NC_001145.3..354141..354263 | 0.463 | 8.799 | 0.300 | 0.584 | 158.18 | 327.28 | 240.39 | 425.23 |
| siRNA22 | NC_001134.8..797493..797593 | -0.620 | 8.453 | 0.298 | 0.585 | 474.54 | 0.00 | 240.39 | 70.87 |
| siRNA22 | NC_001136.10..498005..498107 | 0.610 | 8.280 | 0.293 | 0.588 | 158.18 | 81.82 | 240.39 | 141.74 |
| siRNA22 | NC_001144.5..207838..207959 | 0.599 | 8.277 | 0.286 | 0.593 | 158.18 | 81.82 | 160.26 | 212.61 |
| siRNA22 | NC_001143.9..27636..27783 | -0.382 | 9.086 | 0.271 | 0.602 | 474.54 | 409.10 | 240.39 | 425.23 |
| siRNA22 | NC_001145.3..449468..449589 | -0.356 | 9.188 | 0.262 | 0.609 | 395.45 | 572.74 | 320.53 | 425.23 |
| siRNA22 | NC_001136.10..1326336..1326457 | 0.583 | 8.275 | 0.255 | 0.613 | 79.09 | 163.64 | 80.13 | 283.49 |
| siRNA22 | NC_001145.3..878500..878618 | 0.449 | 8.792 | 0.244 | 0.622 | 316.36 | 163.64 | 80.13 | 566.97 |
| siRNA22 | NC_001134.8..372083..372206 | 0.393 | 8.919 | 0.232 | 0.630 | 158.18 | 409.10 | 240.39 | 496.10 |
| siRNA22 | NC_001145.3..98107..98230 | -0.396 | 8.992 | 0.201 | 0.654 | 79.09 | 736.38 | 480.79 | 141.74 |
| siRNA22 | NC_001139.9..577550..577676 | -0.214 | 10.036 | 0.163 | 0.686 | 1344.54 | 572.74 | 881.45 | 779.58 |
| siRNA22 | NC_001139.9..864219..864337 | -0.473 | 8.191 | 0.158 | 0.691 | 79.09 | 245.46 | 160.26 | 70.87 |
| siRNA22 | NC_001144.5..533317..533434 | -0.517 | 8.185 | 0.153 | 0.696 | 0.00 | 327.28 | 0.00 | 212.61 |
| siRNA22 | NC_001142.9..557787..557900 | -0.452 | 8.193 | 0.136 | 0.712 | 158.18 | 163.64 | 240.39 | 0.00 |
| siRNA22 | NC_001140.6..300637..300755 | -0.391 | 8.368 | 0.130 | 0.719 | 316.36 | 81.82 | 160.26 | 141.74 |
| siRNA22 | NC_001141.2..83696..83816 | -0.309 | 8.675 | 0.122 | 0.727 | 158.18 | 409.10 | 240.39 | 212.61 |
| siRNA22 | NC_001142.9..197865..197989 | 0.153 | 16.507 | 0.113 | 0.737 | 84310.43 | 91801.79 | 102247.65 | 93621.07 |
| siRNA22 | NC_001136.10..1354744..1354856 | -0.296 | 8.676 | 0.109 | 0.741 | 395.45 | 163.64 | 320.53 | 141.74 |
| siRNA22 | NC_001146.8..702823..702946 | -0.298 | 8.804 | 0.108 | 0.742 | 79.09 | 572.74 | 160.26 | 354.36 |
| siRNA22 | NC_001146.8..139467..139586 | -0.303 | 8.679 | 0.104 | 0.747 | 79.09 | 490.92 | 320.53 | 141.74 |
| siRNA22 | NC_001147.6..255592..255818 | -0.181 | 9.703 | 0.104 | 0.747 | 790.90 | 654.56 | 480.79 | 779.58 |
| siRNA22 | NC_001136.10..1251173..1251314 | -0.196 | 9.412 | 0.100 | 0.752 | 632.72 | 490.92 | 560.92 | 425.23 |
| siRNA22 | NC_001144.5..185965..186085 | -0.225 | 9.144 | 0.097 | 0.756 | 316.36 | 572.74 | 480.79 | 283.49 |
| siRNA22 | NC_001139.9..553703..553877 | -0.176 | 9.641 | 0.094 | 0.759 | 790.90 | 572.74 | 721.18 | 496.10 |
| siRNA22 | NC_001137.3..363625..363746 | -0.284 | 8.674 | 0.082 | 0.774 | 553.63 | 0.00 | 320.53 | 141.74 |
| siRNA22 | NC_001139.9..135243..135364 | 0.227 | 8.978 | 0.077 | 0.782 | 553.63 | 81.82 | 400.66 | 354.36 |
| siRNA22 | NC_001134.8..204457..204561 | 0.334 | 8.193 | 0.069 | 0.793 | 158.18 | 81.82 | 320.53 | 0.00 |
| siRNA22 | NC_001134.8..115991..116109 | 0.309 | 8.190 | 0.067 | 0.796 | 79.09 | 163.64 | 240.39 | 70.87 |
| siRNA22 | NC_001136.10..938532..938633 | 0.300 | 8.186 | 0.066 | 0.797 | 158.18 | 81.82 | 160.26 | 141.74 |
| siRNA22 | NC_001147.6..972033..972142 | 0.300 | 8.186 | 0.066 | 0.797 | 158.18 | 81.82 | 160.26 | 141.74 |
| siRNA22 | NC_001139.9..732381..732498 | 0.199 | 8.363 | 0.034 | 0.854 | 158.18 | 163.64 | 80.13 | 283.49 |
| siRNA22 | NC_001136.10..651533..651656 | 0.128 | 9.276 | 0.033 | 0.855 | 237.27 | 654.56 | 320.53 | 637.84 |
| siRNA22 | NC_001144.5..1048034..1048142 | 0.169 | 8.531 | 0.031 | 0.860 | 237.27 | 163.64 | 320.53 | 141.74 |
| siRNA22 | NC_001148.4..23337..23718 | 0.081 | 11.037 | 0.030 | 0.863 | 1898.17 | 1881.85 | 2163.55 | 1842.66 |
| siRNA22 | NC_001148.4..86220..86341 | -0.102 | 9.277 | 0.023 | 0.879 | 395.45 | 572.74 | 320.53 | 566.97 |
| siRNA22 | NC_001136.10..508793..509003 | -0.103 | 8.979 | 0.017 | 0.895 | 237.27 | 490.92 | 240.39 | 425.23 |
| siRNA22 | NC_001146.8..58112..58242 | 0.095 | 8.804 | 0.013 | 0.908 | 316.36 | 245.46 | 320.53 | 283.49 |
| siRNA22 | NC_001134.8..474719..474836 | -0.100 | 8.451 | 0.009 | 0.923 | 79.09 | 327.28 | 160.26 | 212.61 |
| siRNA22 | NC_001144.5..650494..650609 | -0.099 | 8.277 | 0.007 | 0.931 | 237.27 | 81.82 | 80.13 | 212.61 |
| siRNA22 | NC_001148.4..874934..875051 | -0.096 | 8.276 | 0.006 | 0.936 | 316.36 | 0.00 | 80.13 | 212.61 |
| siRNA22 | NC_001147.6..663166..663286 | -0.070 | 8.747 | 0.006 | 0.937 | 316.36 | 245.46 | 480.79 | 70.87 |
| siRNA22 | NC_001145.3..893926..894027 | -0.081 | 8.279 | 0.005 | 0.945 | 316.36 | 0.00 | 160.26 | 141.74 |
| siRNA22 | NC_001146.8..348436..348554 | -0.060 | 8.461 | 0.003 | 0.959 | 0.00 | 409.10 | 400.66 | 0.00 |
| siRNA22 | NC_001146.8..380446..380568 | 0.007 | 9.409 | 0.000 | 0.991 | 395.45 | 654.56 | 480.79 | 566.97 |
| siRNA23 | NC_001136.10..887117..887222 | 4.798 | 8.177 | 7.389 | 0.007 | 0.00 | 0.00 | 160.26 | 354.36 |
| siRNA23 | NC_001139.9..372084..372190 | -4.699 | 8.095 | 6.798 | 0.009 | 79.09 | 409.10 | 0.00 | 0.00 |
| siRNA23 | NC_001137.3..323499..323612 | 3.061 | 8.584 | 6.355 | 0.012 | 79.09 | 0.00 | 160.26 | 637.84 |
| siRNA23 | NC_001142.9..412973..413094 | -2.980 | 8.456 | 6.180 | 0.013 | 316.36 | 409.10 | 0.00 | 70.87 |
| siRNA23 | NC_001138.5..221370..221488 | 2.794 | 8.439 | 5.165 | 0.023 | 79.09 | 0.00 | 240.39 | 425.23 |
| siRNA23 | NC_001148.4..654092..654204 | -2.625 | 8.283 | 4.030 | 0.045 | 395.45 | 163.64 | 0.00 | 70.87 |
| siRNA23 | NC_001136.10..107293..107417 | -1.763 | 8.675 | 3.371 | 0.066 | 316.36 | 490.92 | 0.00 | 212.61 |
| siRNA23 | NC_001143.9..568470..568582 | -1.953 | 8.459 | 3.157 | 0.076 | 316.36 | 327.28 | 160.26 | 0.00 |
| siRNA23 | NC_001137.3..359634..359754 | -1.590 | 8.607 | 2.513 | 0.113 | 553.63 | 163.64 | 160.26 | 70.87 |
| siRNA23 | NC_001141.2..418914..419032 | -1.361 | 8.744 | 2.393 | 0.122 | 474.54 | 327.28 | 160.26 | 141.74 |
| siRNA23 | NC_001136.10..301591..301694 | -2.159 | 8.088 | 2.382 | 0.123 | 316.36 | 81.82 | 0.00 | 70.87 |
| siRNA23 | NC_001134.8..359147..359253 | 1.996 | 8.086 | 2.104 | 0.147 | 79.09 | 0.00 | 240.39 | 141.74 |
| siRNA23 | NC_001139.9..486578..486703 | -0.820 | 9.605 | 1.863 | 0.172 | 1028.18 | 572.74 | 400.66 | 496.10 |
| siRNA23 | NC_001224.1..60181..60382 | 0.881 | 9.275 | 1.679 | 0.195 | 237.27 | 409.10 | 560.92 | 637.84 |
| siRNA23 | NC_001143.9..35827..35967 | -0.968 | 9.087 | 1.573 | 0.210 | 711.81 | 327.28 | 160.26 | 354.36 |
| siRNA23 | NC_001136.10..1237660..1237780 | 1.011 | 8.863 | 1.489 | 0.222 | 237.27 | 163.64 | 480.79 | 354.36 |
| siRNA23 | NC_001224.1..49854..49976 | -1.051 | 8.808 | 1.473 | 0.225 | 553.63 | 245.46 | 240.39 | 141.74 |
| siRNA23 | NC_001144.5..513382..513552 | -0.755 | 9.375 | 1.385 | 0.239 | 632.72 | 654.56 | 480.79 | 283.49 |
| siRNA23 | NC_001143.9..571193..571312 | 1.394 | 8.281 | 1.314 | 0.252 | 0.00 | 163.64 | 320.53 | 141.74 |
| siRNA23 | NC_001145.3..656769..656887 | 1.411 | 8.284 | 1.303 | 0.254 | 79.09 | 81.82 | 400.66 | 70.87 |
| siRNA23 | NC_001145.3..903705..903825 | 1.024 | 8.664 | 1.248 | 0.264 | 237.27 | 81.82 | 240.39 | 425.23 |
| siRNA23 | NC_001143.9..552370..552488 | -1.039 | 8.608 | 1.221 | 0.269 | 395.45 | 245.46 | 240.39 | 70.87 |
| siRNA23 | NC_001140.6..432466..432576 | -1.324 | 8.186 | 1.155 | 0.282 | 237.27 | 163.64 | 0.00 | 141.74 |
| siRNA23 | NC_001135.5..94227..94347 | 1.016 | 8.661 | 1.154 | 0.283 | 237.27 | 81.82 | 160.26 | 496.10 |
| siRNA23 | NC_001141.2..291040..291203 | -0.785 | 9.137 | 1.133 | 0.287 | 632.72 | 409.10 | 160.26 | 425.23 |
| siRNA23 | NC_001148.4..103370..103492 | -0.724 | 9.236 | 1.107 | 0.293 | 474.54 | 654.56 | 240.39 | 425.23 |
| siRNA23 | NC_001135.5..177838..177942 | 1.063 | 8.446 | 1.052 | 0.305 | 79.09 | 163.64 | 240.39 | 283.49 |
| siRNA23 | NC_001139.9..341725..341845 | -0.806 | 8.869 | 1.002 | 0.317 | 395.45 | 409.10 | 240.39 | 212.61 |
| siRNA23 | NC_001143.9..601873..601995 | 1.140 | 8.187 | 0.888 | 0.346 | 79.09 | 81.82 | 240.39 | 141.74 |
| siRNA23 | NC_001148.4..684597..684705 | 1.137 | 8.187 | 0.836 | 0.361 | 0.00 | 163.64 | 240.39 | 141.74 |
| siRNA23 | NC_001137.3..114692..114795 | 1.162 | 8.189 | 0.816 | 0.366 | 158.18 | 0.00 | 320.53 | 70.87 |
| siRNA23 | NC_001143.9..36480..36601 | 0.744 | 8.919 | 0.795 | 0.373 | 79.09 | 409.10 | 320.53 | 496.10 |
| siRNA23 | NC_001142.9..215964..216089 | 0.849 | 8.595 | 0.774 | 0.379 | 79.09 | 245.46 | 160.26 | 425.23 |
| siRNA23 | NC_001134.8..88483..88591 | 0.853 | 8.598 | 0.752 | 0.386 | 0.00 | 327.28 | 240.39 | 354.36 |
| siRNA23 | NC_001148.4..397696..397814 | 1.186 | 8.192 | 0.742 | 0.389 | 158.18 | 0.00 | 400.66 | 0.00 |
| siRNA23 | NC_001134.8..458502..458624 | -0.881 | 8.524 | 0.727 | 0.394 | 395.45 | 163.64 | 0.00 | 283.49 |
| siRNA23 | NC_001134.8..425374..425485 | -0.868 | 8.533 | 0.722 | 0.395 | 79.09 | 490.92 | 160.26 | 141.74 |
| siRNA23 | NC_001139.9..474016..474142 | -0.532 | 9.374 | 0.686 | 0.408 | 474.54 | 736.38 | 480.79 | 354.36 |
| siRNA23 | NC_001142.9..253184..253300 | -1.002 | 8.089 | 0.625 | 0.429 | 237.27 | 81.82 | 80.13 | 70.87 |
| siRNA23 | NC_001148.4..559789..559896 | -1.038 | 8.088 | 0.566 | 0.452 | 0.00 | 327.28 | 0.00 | 141.74 |
| siRNA23 | NC_001139.9..741341..741453 | 0.697 | 8.728 | 0.552 | 0.457 | 158.18 | 245.46 | 80.13 | 566.97 |
| siRNA23 | NC_001145.3..852966..853089 | 0.629 | 8.861 | 0.503 | 0.478 | 474.54 | 0.00 | 400.66 | 354.36 |
| siRNA23 | NC_001142.9..683039..683146 | -0.789 | 8.281 | 0.496 | 0.481 | 158.18 | 245.46 | 80.13 | 141.74 |
| siRNA23 | NC_001148.4..897997..898102 | 0.686 | 8.527 | 0.495 | 0.482 | 237.27 | 81.82 | 320.53 | 212.61 |
| siRNA23 | NC_001141.2..256815..256933 | -0.786 | 8.280 | 0.493 | 0.482 | 237.27 | 163.64 | 80.13 | 141.74 |
| siRNA23 | NC_001145.3..62601..62726 | -0.503 | 9.142 | 0.481 | 0.488 | 316.36 | 654.56 | 320.53 | 354.36 |
| siRNA23 | NC_001147.6..111781..111902 | -0.769 | 8.283 | 0.442 | 0.506 | 316.36 | 81.82 | 160.26 | 70.87 |
| siRNA23 | NC_001135.5..172451..172573 | -0.410 | 9.413 | 0.438 | 0.508 | 553.63 | 654.56 | 480.79 | 425.23 |
| siRNA23 | NC_001136.10..750036..750158 | -0.657 | 8.449 | 0.420 | 0.517 | 237.27 | 245.46 | 80.13 | 212.61 |
| siRNA23 | NC_001143.9..543509..543629 | 0.653 | 8.520 | 0.402 | 0.526 | 79.09 | 245.46 | 80.13 | 425.23 |
| siRNA23 | NC_001142.9..569197..569313 | -0.780 | 8.279 | 0.401 | 0.527 | 395.45 | 0.00 | 80.13 | 141.74 |
| siRNA23 | NC_001136.10..1327925..1328043 | 0.821 | 8.082 | 0.399 | 0.528 | 158.18 | 0.00 | 80.13 | 212.61 |
| siRNA23 | NC_001141.2..176949..177055 | 0.812 | 8.084 | 0.389 | 0.533 | 0.00 | 163.64 | 80.13 | 212.61 |
| siRNA23 | NC_001143.9..532538..532660 | 0.586 | 8.680 | 0.369 | 0.543 | 158.18 | 245.46 | 560.92 | 70.87 |
| siRNA23 | NC_001142.9..451532..451639 | 0.799 | 8.080 | 0.359 | 0.549 | 79.09 | 81.82 | 0.00 | 283.49 |
| siRNA23 | NC_001142.9..470214..470334 | -0.547 | 8.610 | 0.328 | 0.567 | 158.18 | 409.10 | 320.53 | 70.87 |
| siRNA23 | NC_001144.5..539159..539309 | 0.472 | 8.800 | 0.321 | 0.571 | 316.36 | 163.64 | 320.53 | 354.36 |
| siRNA23 | NC_001141.2..51404..51601 | -0.406 | 8.983 | 0.286 | 0.593 | 395.45 | 409.10 | 320.53 | 283.49 |
| siRNA23 | NC_001224.1..33039..33164 | 0.327 | 9.625 | 0.271 | 0.603 | 790.90 | 327.28 | 400.66 | 992.20 |
| siRNA23 | NC_001142.9..299130..299248 | 0.587 | 8.274 | 0.259 | 0.611 | 158.18 | 81.82 | 80.13 | 283.49 |
| siRNA23 | NC_001147.6..87886..88003 | 0.587 | 8.274 | 0.259 | 0.611 | 158.18 | 81.82 | 80.13 | 283.49 |
| siRNA23 | NC_001146.8..756412..756533 | -0.348 | 9.188 | 0.246 | 0.620 | 632.72 | 327.28 | 400.66 | 354.36 |
| siRNA23 | NC_001139.9..21826..21986 | -0.384 | 9.091 | 0.238 | 0.626 | 158.18 | 736.38 | 320.53 | 354.36 |
| siRNA23 | NC_001147.6..553896..554014 | 0.466 | 8.449 | 0.216 | 0.642 | 158.18 | 163.64 | 240.39 | 212.61 |
| siRNA23 | NC_001146.8..33953..34056 | 0.449 | 8.442 | 0.179 | 0.672 | 237.27 | 81.82 | 80.13 | 354.36 |
| siRNA23 | NC_001140.6..143831..143950 | 0.442 | 8.444 | 0.173 | 0.677 | 79.09 | 245.46 | 80.13 | 354.36 |
| siRNA23 | NC_001144.5..156238..156359 | 0.378 | 8.602 | 0.173 | 0.677 | 237.27 | 163.64 | 320.53 | 212.61 |
| siRNA23 | NC_001134.8..114123..114242 | 0.370 | 8.600 | 0.167 | 0.683 | 158.18 | 245.46 | 240.39 | 283.49 |
| siRNA23 | NC_001142.9..293582..293704 | -0.505 | 8.184 | 0.165 | 0.685 | 79.09 | 245.46 | 0.00 | 212.61 |
| siRNA23 | NC_001144.5..244999..245106 | -0.470 | 8.190 | 0.163 | 0.687 | 158.18 | 163.64 | 160.26 | 70.87 |
| siRNA23 | NC_001143.9..541001..541120 | -0.397 | 8.370 | 0.141 | 0.707 | 158.18 | 245.46 | 160.26 | 141.74 |
| siRNA23 | NC_001142.9..169328..169430 | -0.342 | 8.527 | 0.120 | 0.729 | 395.45 | 81.82 | 160.26 | 212.61 |
| siRNA23 | NC_001137.3..327625..327747 | -0.182 | 9.773 | 0.095 | 0.758 | 395.45 | 1145.48 | 560.92 | 779.58 |
| siRNA23 | NC_001137.3..193490..193596 | -0.315 | 8.538 | 0.091 | 0.763 | 158.18 | 327.28 | 400.66 | 0.00 |
| siRNA23 | NC_001148.4..498966..499090 | 0.192 | 9.088 | 0.070 | 0.792 | 395.45 | 327.28 | 480.79 | 354.36 |
| siRNA23 | NC_001142.9..336855..336972 | 0.290 | 8.182 | 0.055 | 0.815 | 237.27 | 0.00 | 80.13 | 212.61 |
| siRNA23 | NC_001144.5..1012932..1013040 | 0.232 | 8.372 | 0.045 | 0.833 | 158.18 | 163.64 | 320.53 | 70.87 |
| siRNA23 | NC_001141.2..429113..429235 | 0.223 | 8.368 | 0.043 | 0.836 | 237.27 | 81.82 | 240.39 | 141.74 |
| siRNA23 | NC_001145.3..484914..485057 | -0.101 | 9.669 | 0.032 | 0.859 | 632.72 | 736.38 | 480.79 | 779.58 |
| siRNA23 | NC_001134.8..702544..702662 | 0.159 | 8.528 | 0.028 | 0.866 | 158.18 | 245.46 | 240.39 | 212.61 |
| siRNA23 | NC_001144.5..83274..83394 | 0.169 | 8.526 | 0.027 | 0.870 | 395.45 | 0.00 | 240.39 | 212.61 |
| siRNA23 | NC_001147.6..662258..662379 | 0.144 | 8.522 | 0.022 | 0.883 | 237.27 | 163.64 | 80.13 | 354.36 |
| siRNA23 | NC_001146.8..534373..534495 | 0.125 | 8.674 | 0.021 | 0.886 | 237.27 | 245.46 | 320.53 | 212.61 |
| siRNA23 | NC_001146.8..580072..580280 | -0.092 | 8.984 | 0.014 | 0.905 | 237.27 | 490.92 | 400.66 | 283.49 |
| siRNA23 | NC_001136.10..637699..637806 | -0.112 | 8.447 | 0.011 | 0.916 | 79.09 | 327.28 | 80.13 | 283.49 |
| siRNA23 | NC_001143.9..64961..65078 | -0.104 | 8.446 | 0.010 | 0.919 | 237.27 | 163.64 | 80.13 | 283.49 |
| siRNA23 | NC_001134.8..544453..544556 | -0.110 | 8.083 | 0.007 | 0.932 | 158.18 | 81.82 | 0.00 | 212.61 |
| siRNA23 | NC_001139.9..598247..598362 | -0.093 | 8.282 | 0.007 | 0.934 | 79.09 | 245.46 | 160.26 | 141.74 |
| siRNA23 | NC_001137.3..87189..87307 | -0.096 | 8.276 | 0.006 | 0.936 | 316.36 | 0.00 | 80.13 | 212.61 |
| siRNA23 | NC_001134.8..85296..85409 | -0.093 | 8.086 | 0.006 | 0.940 | 158.18 | 81.82 | 80.13 | 141.74 |
| siRNA23 | NC_001139.9..497778..497895 | -0.080 | 8.089 | 0.004 | 0.948 | 158.18 | 81.82 | 160.26 | 70.87 |
| siRNA23 | NC_001134.8..124747..124852 | -0.073 | 8.088 | 0.003 | 0.955 | 237.27 | 0.00 | 160.26 | 70.87 |
| siRNA23 | NC_001145.3..367380..367488 | -0.065 | 8.094 | 0.002 | 0.961 | 0.00 | 245.46 | 240.39 | 0.00 |
| siRNA23 | NC_001145.3..355902..356008 | -0.052 | 8.286 | 0.002 | 0.965 | 237.27 | 81.82 | 320.53 | 0.00 |
| siRNA23 | NC_001133.9..191572..192057 | -0.013 | 10.747 | 0.001 | 0.978 | 1502.72 | 1636.40 | 1923.15 | 1204.81 |
| siRNA23 | NC_001142.9..523043..523166 | -0.011 | 9.398 | 0.000 | 0.988 | 632.72 | 409.10 | 160.26 | 850.46 |
| siRNA24 | NC_001136.10..435689..435812 | -2.113 | 9.148 | 7.402 | 0.007 | 711.81 | 654.56 | 160.26 | 141.74 |
| siRNA24 | NC_001148.4..835072..835188 | 4.784 | 8.173 | 6.927 | 0.008 | 0.00 | 0.00 | 80.13 | 425.23 |
| siRNA24 | NC_001144.5..827331..827444 | -2.634 | 8.286 | 4.033 | 0.045 | 158.18 | 409.10 | 0.00 | 70.87 |
| siRNA24 | NC_001146.8..63819..63985 | -1.145 | 9.682 | 3.785 | 0.052 | 711.81 | 1145.48 | 400.66 | 425.23 |
| siRNA24 | NC_001136.10..165771..165891 | 1.993 | 8.533 | 2.834 | 0.092 | 158.18 | 0.00 | 641.05 | 70.87 |
| siRNA24 | NC_001141.2..98505..98628 | -1.278 | 9.098 | 2.818 | 0.093 | 474.54 | 654.56 | 320.53 | 141.74 |
| siRNA24 | NC_001141.2..159409..159529 | 2.226 | 8.180 | 2.763 | 0.096 | 0.00 | 81.82 | 160.26 | 283.49 |
| siRNA24 | NC_001146.8..404091..404206 | -2.434 | 8.193 | 2.722 | 0.099 | 0.00 | 490.92 | 0.00 | 70.87 |
| siRNA24 | NC_001143.9..401951..402061 | -1.611 | 8.602 | 2.609 | 0.106 | 474.54 | 245.46 | 0.00 | 212.61 |
| siRNA24 | NC_001147.6..824085..824196 | -1.789 | 8.369 | 2.429 | 0.119 | 316.36 | 245.46 | 0.00 | 141.74 |
| siRNA24 | NC_001144.5..152983..153105 | -1.787 | 8.367 | 2.134 | 0.144 | 474.54 | 81.82 | 0.00 | 141.74 |
| siRNA24 | NC_001144.5..499681..499781 | -1.787 | 8.367 | 2.134 | 0.144 | 474.54 | 81.82 | 0.00 | 141.74 |
| siRNA24 | NC_001144.5..942936..943050 | 1.984 | 8.082 | 2.096 | 0.148 | 79.09 | 0.00 | 160.26 | 212.61 |
| siRNA24 | NC_001148.4..658381..658493 | 1.984 | 8.082 | 2.096 | 0.148 | 79.09 | 0.00 | 160.26 | 212.61 |
| siRNA24 | NC_001136.10..575167..575290 | -0.869 | 9.533 | 2.080 | 0.149 | 870.00 | 654.56 | 400.66 | 425.23 |
| siRNA24 | NC_001145.3..156387..156527 | 0.660 | 11.533 | 2.016 | 0.156 | 2135.44 | 2209.13 | 2964.86 | 3897.92 |
| siRNA24 | NC_001144.5..359521..359637 | 1.586 | 8.359 | 1.898 | 0.168 | 79.09 | 81.82 | 160.26 | 354.36 |
| siRNA24 | NC_001141.2..175670..175785 | -1.576 | 8.281 | 1.772 | 0.183 | 237.27 | 245.46 | 0.00 | 141.74 |
| siRNA24 | NC_001139.9..39434..39557 | -0.881 | 9.192 | 1.586 | 0.208 | 632.72 | 490.92 | 320.53 | 283.49 |
| siRNA24 | NC_001140.6..239887..240010 | -1.254 | 8.453 | 1.478 | 0.224 | 237.27 | 327.28 | 80.13 | 141.74 |
| siRNA24 | NC_001144.5..439663..439782 | -0.891 | 9.192 | 1.464 | 0.226 | 316.36 | 818.20 | 240.39 | 354.36 |
| siRNA24 | NC_001140.6..299227..299348 | -1.244 | 8.456 | 1.452 | 0.228 | 237.27 | 327.28 | 160.26 | 70.87 |
| siRNA24 | NC_001148.4..60054..60172 | -1.246 | 8.457 | 1.392 | 0.238 | 158.18 | 409.10 | 160.26 | 70.87 |
| siRNA24 | NC_001136.10..1501634..1501750 | 1.376 | 8.274 | 1.373 | 0.241 | 79.09 | 81.82 | 160.26 | 283.49 |
| siRNA24 | NC_001140.6..52147..52254 | 1.390 | 8.276 | 1.359 | 0.244 | 158.18 | 0.00 | 240.39 | 212.61 |
| siRNA24 | NC_001146.8..195664..195786 | 1.402 | 8.280 | 1.332 | 0.248 | 158.18 | 0.00 | 320.53 | 141.74 |
| siRNA24 | NC_001146.8..426293..426445 | -0.854 | 9.039 | 1.270 | 0.260 | 395.45 | 572.74 | 240.39 | 283.49 |
| siRNA24 | NC_001147.6..883375..883489 | 1.050 | 8.677 | 1.214 | 0.271 | 158.18 | 163.64 | 560.92 | 141.74 |
| siRNA24 | NC_001145.3..18209..18319 | -1.309 | 8.190 | 1.188 | 0.276 | 237.27 | 163.64 | 80.13 | 70.87 |
| siRNA24 | NC_001142.9..680542..680668 | -0.527 | 10.232 | 1.020 | 0.313 | 1660.90 | 818.20 | 641.05 | 1063.07 |
| siRNA24 | NC_001148.4..768135..768251 | -1.321 | 8.192 | 0.994 | 0.319 | 0.00 | 409.10 | 80.13 | 70.87 |
| siRNA24 | NC_001139.9..199341..199462 | -0.758 | 9.152 | 0.941 | 0.332 | 395.45 | 654.56 | 560.92 | 70.87 |
| siRNA24 | NC_001138.5..2072..2195 | -0.916 | 8.737 | 0.909 | 0.340 | 632.72 | 81.82 | 80.13 | 283.49 |
| siRNA24 | NC_001148.4..493005..493116 | 1.155 | 8.190 | 0.847 | 0.357 | 79.09 | 81.82 | 320.53 | 70.87 |
| siRNA24 | NC_001138.5..123766..123883 | 1.137 | 8.187 | 0.836 | 0.361 | 0.00 | 163.64 | 240.39 | 141.74 |
| siRNA24 | NC_001141.2..124140..124260 | 0.716 | 8.734 | 0.679 | 0.410 | 158.18 | 245.46 | 240.39 | 425.23 |
| siRNA24 | NC_001136.10..972825..972937 | -0.659 | 8.807 | 0.636 | 0.425 | 316.36 | 409.10 | 240.39 | 212.61 |
| siRNA24 | NC_001137.3..7384..7508 | 0.495 | 9.623 | 0.601 | 0.438 | 474.54 | 572.74 | 320.53 | 1133.94 |
| siRNA24 | NC_001141.2..3206..3329 | -0.594 | 8.925 | 0.571 | 0.450 | 474.54 | 327.28 | 240.39 | 283.49 |
| siRNA24 | NC_001134.8..619276..619402 | 0.708 | 8.735 | 0.569 | 0.451 | 0.00 | 409.10 | 240.39 | 425.23 |
| siRNA24 | NC_001145.3..755079..755253 | -0.492 | 9.454 | 0.555 | 0.456 | 949.09 | 327.28 | 560.92 | 354.36 |
| siRNA24 | NC_001144.5..178850..178965 | -0.996 | 8.088 | 0.555 | 0.456 | 316.36 | 0.00 | 80.13 | 70.87 |
| siRNA24 | NC_001136.10..1213467..1213577 | -0.789 | 8.281 | 0.496 | 0.481 | 158.18 | 245.46 | 80.13 | 141.74 |
| siRNA24 | NC_001136.10..1183302..1183422 | 0.681 | 8.529 | 0.487 | 0.485 | 79.09 | 245.46 | 320.53 | 212.61 |
| siRNA24 | NC_001143.9..156547..156654 | 0.658 | 8.519 | 0.420 | 0.517 | 158.18 | 163.64 | 80.13 | 425.23 |
| siRNA24 | NC_001146.8..288800..288923 | 0.561 | 8.671 | 0.412 | 0.521 | 237.27 | 163.64 | 320.53 | 283.49 |
| siRNA24 | NC_001145.3..249780..249880 | -0.650 | 8.453 | 0.412 | 0.521 | 158.18 | 327.28 | 160.26 | 141.74 |
| siRNA24 | NC_001147.6..803587..803702 | 0.841 | 8.090 | 0.411 | 0.522 | 0.00 | 163.64 | 240.39 | 70.87 |
| siRNA24 | NC_001143.9..383596..383714 | 0.554 | 8.669 | 0.397 | 0.529 | 158.18 | 245.46 | 240.39 | 354.36 |
| siRNA24 | NC_001134.8..708358..708479 | -0.638 | 8.456 | 0.394 | 0.530 | 237.27 | 245.46 | 240.39 | 70.87 |
| siRNA24 | NC_001139.9..410719..410819 | 0.812 | 8.084 | 0.389 | 0.533 | 0.00 | 163.64 | 80.13 | 212.61 |
| siRNA24 | NC_001148.4..372576..372695 | -0.567 | 8.599 | 0.359 | 0.549 | 395.45 | 163.64 | 80.13 | 283.49 |
| siRNA24 | NC_001144.5..991514..991635 | 0.550 | 8.664 | 0.354 | 0.552 | 316.36 | 81.82 | 160.26 | 425.23 |
| siRNA24 | NC_001146.8..490..608 | 0.789 | 8.080 | 0.331 | 0.565 | 0.00 | 163.64 | 0.00 | 283.49 |
| siRNA24 | NC_001140.6..26678..26798 | 0.599 | 8.277 | 0.286 | 0.593 | 158.18 | 81.82 | 160.26 | 212.61 |
| siRNA24 | NC_001147.6..1049057..1049174 | 0.599 | 8.277 | 0.286 | 0.593 | 158.18 | 81.82 | 160.26 | 212.61 |
| siRNA24 | NC_001145.3..237104..237216 | 0.603 | 8.282 | 0.261 | 0.609 | 0.00 | 245.46 | 240.39 | 141.74 |
| siRNA24 | NC_001138.5..115084..115194 | 0.590 | 8.279 | 0.252 | 0.615 | 0.00 | 245.46 | 160.26 | 212.61 |
| siRNA24 | NC_001143.9..463979..464083 | 0.466 | 8.449 | 0.216 | 0.642 | 158.18 | 163.64 | 240.39 | 212.61 |
| siRNA24 | NC_001137.3..413372..413486 | 0.374 | 8.599 | 0.162 | 0.687 | 316.36 | 81.82 | 240.39 | 283.49 |
| siRNA24 | NC_001145.3..680161..680281 | -0.473 | 8.191 | 0.158 | 0.691 | 79.09 | 245.46 | 160.26 | 70.87 |
| siRNA24 | NC_001224.1..27341..27473 | -0.199 | 10.318 | 0.145 | 0.703 | 1660.90 | 736.38 | 801.31 | 1275.68 |
| siRNA24 | NC_001147.6..1012589..1012703 | -0.476 | 8.192 | 0.142 | 0.706 | 0.00 | 327.28 | 160.26 | 70.87 |
| siRNA24 | NC_001136.10..1150044..1150163 | -0.394 | 8.369 | 0.140 | 0.709 | 237.27 | 163.64 | 160.26 | 141.74 |
| siRNA24 | NC_001142.9..92194..92303 | -0.421 | 8.368 | 0.128 | 0.721 | 0.00 | 409.10 | 80.13 | 212.61 |
| siRNA24 | NC_001134.8..26987..27116 | -0.306 | 8.674 | 0.125 | 0.723 | 316.36 | 245.46 | 240.39 | 212.61 |
| siRNA24 | NC_001147.6..536261..536383 | -0.306 | 8.674 | 0.125 | 0.723 | 316.36 | 245.46 | 240.39 | 212.61 |
| siRNA24 | NC_001136.10..743562..743683 | 0.300 | 8.186 | 0.066 | 0.797 | 158.18 | 81.82 | 160.26 | 141.74 |
| siRNA24 | NC_001142.9..318058..318175 | 0.297 | 8.187 | 0.065 | 0.799 | 79.09 | 163.64 | 160.26 | 141.74 |
| siRNA24 | NC_001136.10..240271..240396 | 0.182 | 9.084 | 0.062 | 0.804 | 316.36 | 409.10 | 320.53 | 496.10 |
| siRNA24 | NC_001146.8..372263..372387 | 0.174 | 9.086 | 0.047 | 0.829 | 79.09 | 654.56 | 320.53 | 496.10 |
| siRNA24 | NC_001147.6..965588..965703 | 0.271 | 8.179 | 0.043 | 0.836 | 237.27 | 0.00 | 0.00 | 283.49 |
| siRNA24 | NC_001146.8..633583..633710 | 0.124 | 9.369 | 0.038 | 0.845 | 395.45 | 572.74 | 560.92 | 496.10 |
| siRNA24 | NC_001148.4..242201..242372 | 0.102 | 9.533 | 0.028 | 0.867 | 553.63 | 572.74 | 801.31 | 425.23 |
| siRNA24 | NC_001139.9..851385..851508 | 0.127 | 8.673 | 0.021 | 0.885 | 316.36 | 163.64 | 320.53 | 212.61 |
| siRNA24 | NC_001136.10..196725..196844 | 0.146 | 8.520 | 0.019 | 0.891 | 395.45 | 0.00 | 80.13 | 354.36 |
| siRNA24 | NC_001140.6..92666..92788 | 0.112 | 8.809 | 0.016 | 0.898 | 395.45 | 163.64 | 480.79 | 141.74 |
| siRNA24 | NC_001134.8..490338..490445 | -0.112 | 8.447 | 0.011 | 0.916 | 79.09 | 327.28 | 80.13 | 283.49 |
| siRNA24 | NC_001139.9..876640..876750 | -0.102 | 8.445 | 0.009 | 0.923 | 316.36 | 81.82 | 80.13 | 283.49 |
| siRNA24 | NC_001145.3..291722..291839 | -0.087 | 8.608 | 0.008 | 0.927 | 79.09 | 409.10 | 320.53 | 141.74 |
| siRNA24 | NC_001142.9..530441..530558 | -0.101 | 8.278 | 0.008 | 0.928 | 158.18 | 163.64 | 80.13 | 212.61 |
| siRNA24 | NC_001145.3..792591..792710 | -0.088 | 8.453 | 0.008 | 0.930 | 158.18 | 245.46 | 240.39 | 141.74 |
| siRNA24 | NC_001147.6..261519..261638 | -0.085 | 8.452 | 0.007 | 0.932 | 237.27 | 163.64 | 240.39 | 141.74 |
| siRNA24 | NC_001135.5..41961..42066 | -0.109 | 8.082 | 0.006 | 0.936 | 237.27 | 0.00 | 0.00 | 212.61 |
| siRNA24 | NC_001134.8..472819..472928 | -0.087 | 8.280 | 0.006 | 0.938 | 237.27 | 81.82 | 160.26 | 141.74 |
| siRNA24 | NC_001147.6..362364..362466 | -0.083 | 8.090 | 0.005 | 0.946 | 79.09 | 163.64 | 160.26 | 70.87 |
| siRNA25 | NC_001134.8..330783..330894 | -1.293 | 8.193 | 1.095 | 0.295 | 237.27 | 163.64 | 160.26 | 0.00 |
| siRNA25 | NC_001145.3..509109..509228 | -0.741 | 8.677 | 0.719 | 0.396 | 316.36 | 327.28 | 240.39 | 141.74 |
| siRNA25 | NC_001141.2..109406..109523 | -0.479 | 8.186 | 0.164 | 0.686 | 237.27 | 81.82 | 80.13 | 141.74 |
| siRNA25 | NC_001136.10..219909..220026 | -0.358 | 8.526 | 0.134 | 0.714 | 158.18 | 327.28 | 80.13 | 283.49 |
| siRNA25 | NC_001145.3..270405..270523 | -0.419 | 8.362 | 0.131 | 0.718 | 316.36 | 81.82 | 0.00 | 283.49 |
| siRNA25 | NC_001134.8..406941..407060 | -0.284 | 8.799 | 0.100 | 0.752 | 553.63 | 81.82 | 160.26 | 354.36 |
| siRNA25 | NC_001144.5..1003859..1003971 | 0.297 | 8.187 | 0.065 | 0.799 | 79.09 | 163.64 | 160.26 | 141.74 |
| siRNA25 | NC_001144.5..30439..30554 | -0.115 | 8.083 | 0.008 | 0.930 | 79.09 | 163.64 | 0.00 | 212.61 |
| siRNA26 | NC_001142.9..656598..656713 | -4.688 | 8.092 | 7.169 | 0.007 | 316.36 | 163.64 | 0.00 | 0.00 |
| siRNA26 | NC_001144.5..606970..607094 | -1.736 | 8.927 | 4.024 | 0.045 | 711.81 | 327.28 | 80.13 | 212.61 |
| siRNA26 | NC_001145.3..486779..486890 | -1.005 | 8.090 | 0.651 | 0.420 | 158.18 | 163.64 | 80.13 | 70.87 |
| siRNA26 | NC_001143.9..352352..352469 | 0.818 | 8.083 | 0.416 | 0.519 | 79.09 | 81.82 | 80.13 | 212.61 |
| siRNA26 | NC_001136.10..847898..848019 | -0.556 | 8.607 | 0.359 | 0.549 | 158.18 | 409.10 | 240.39 | 141.74 |
| siRNA26 | NC_001137.3..160385..160504 | 0.161 | 8.528 | 0.029 | 0.864 | 237.27 | 163.64 | 240.39 | 212.61 |
| siRNA27 | NC_001141.2..220105..220231 | -1.884 | 8.744 | 4.162 | 0.041 | 553.63 | 327.28 | 80.13 | 141.74 |
| siRNA27 | NC_001148.4..148672..148783 | 0.300 | 8.186 | 0.066 | 0.797 | 158.18 | 81.82 | 160.26 | 141.74 |
| siRNA27 | NC_001147.6..747222..747334 | -0.083 | 8.090 | 0.005 | 0.946 | 79.09 | 163.64 | 160.26 | 70.87 |
| siRNA27 | NC_001146.8..445826..445943 | -0.064 | 8.093 | 0.003 | 0.960 | 79.09 | 163.64 | 240.39 | 0.00 |
| siRNA28 | NC_001147.6..829603..829711 | -1.560 | 8.283 | 1.773 | 0.183 | 316.36 | 163.64 | 80.13 | 70.87 |
| siRNA28 | NC_001143.9..433254..433368 | -0.470 | 8.190 | 0.163 | 0.687 | 158.18 | 163.64 | 160.26 | 70.87 |
| siRNA28 | NC_001144.5..166672..166791 | -0.269 | 8.810 | 0.103 | 0.749 | 316.36 | 327.28 | 400.66 | 141.74 |
| siRNA28 | NC_001143.9..474401..474520 | 0.216 | 8.978 | 0.080 | 0.777 | 316.36 | 327.28 | 320.53 | 425.23 |
| siRNA29 | NC_001147.6..931933..932052 | 1.049 | 8.672 | 1.175 | 0.278 | 316.36 | 0.00 | 480.79 | 212.61 |
| siRNA29 | NC_001148.4..609027..609143 | -1.014 | 8.092 | 0.571 | 0.450 | 0.00 | 327.28 | 80.13 | 70.87 |
| siRNA29 | NC_001148.4..49749..49853 | -0.624 | 8.460 | 0.307 | 0.580 | 79.09 | 409.10 | 320.53 | 0.00 |
| siRNA29 | NC_001141.2..620..743 | 0.303 | 8.736 | 0.128 | 0.720 | 237.27 | 245.46 | 240.39 | 354.36 |
| siRNA29 | NC_001136.10..704988..705094 | -0.387 | 8.374 | 0.122 | 0.727 | 79.09 | 327.28 | 240.39 | 70.87 |
| siRNA29 | NC_001145.3..792998..793120 | -0.295 | 8.798 | 0.115 | 0.735 | 395.45 | 245.46 | 80.13 | 425.23 |
| siRNA30 | NC_001144.5..202455..202560 | -1.597 | 8.876 | 3.081 | 0.079 | 632.72 | 327.28 | 320.53 | 0.00 |
| siRNA30 | NC_001138.5..109717..109839 | -0.803 | 8.868 | 0.940 | 0.332 | 553.63 | 245.46 | 240.39 | 212.61 |
| siRNA30 | NC_001147.6..662684..662802 | -1.008 | 8.375 | 0.784 | 0.376 | 316.36 | 163.64 | 240.39 | 0.00 |
| siRNA30 | NC_001142.9..159486..159589 | -0.466 | 8.189 | 0.154 | 0.695 | 237.27 | 81.82 | 160.26 | 70.87 |
| siRNA30 | NC_001139.9..524100..524212 | -0.093 | 8.086 | 0.006 | 0.940 | 158.18 | 81.82 | 80.13 | 141.74 |
| siRNA31 | NC_001141.2..312204..312321 | 5.321 | 8.446 | 10.699 | 0.001 | 0.00 | 0.00 | 480.79 | 283.49 |
| siRNA31 | NC_001139.9..360171..360288 | -2.410 | 8.189 | 3.050 | 0.081 | 395.45 | 81.82 | 0.00 | 70.87 |
| siRNA31 | NC_001148.4..378354..378474 | -0.529 | 8.612 | 0.288 | 0.592 | 316.36 | 245.46 | 400.66 | 0.00 |
| siRNA31 | NC_001145.3..869221..869341 | 0.466 | 8.449 | 0.216 | 0.642 | 158.18 | 163.64 | 240.39 | 212.61 |
| siRNA32 | NC_001136.10..1291186..1291303 | 0.300 | 8.186 | 0.066 | 0.797 | 158.18 | 81.82 | 160.26 | 141.74 |
| siRNA33 | NC_001134.8..796921..797043 | -1.260 | 9.241 | 3.307 | 0.069 | 632.72 | 654.56 | 240.39 | 283.49 |
| siRNA33 | NC_001147.6..92223..92404 | 0.038 | 9.138 | 0.003 | 0.957 | 316.36 | 490.92 | 400.66 | 425.23 |
| siRNA34 | NC_001224.1..85594..85712 | -2.747 | 8.879 | 8.265 | 0.004 | 474.54 | 654.56 | 160.26 | 0.00 |
| siRNA34 | NC_001134.8..631830..631950 | -0.090 | 8.454 | 0.008 | 0.930 | 79.09 | 327.28 | 240.39 | 141.74 |
| siRNA35 | NC_001145.3..442121..442235 | -0.650 | 8.453 | 0.412 | 0.521 | 158.18 | 327.28 | 160.26 | 141.74 |
| siRNA36 | NC_001144.5..148708..148814 | 1.400 | 8.591 | 1.946 | 0.163 | 79.09 | 163.64 | 160.26 | 496.10 |
| siRNA36 | NC_001142.9..557210..557333 | 0.772 | 9.220 | 1.073 | 0.300 | 316.36 | 327.28 | 240.39 | 850.46 |
| siRNA36 | NC_001145.3..58494..58611 | -0.641 | 8.457 | 0.359 | 0.549 | 79.09 | 409.10 | 240.39 | 70.87 |
| siRNA36 | NC_001142.9..57398..57508 | 0.253 | 8.181 | 0.038 | 0.846 | 0.00 | 245.46 | 0.00 | 283.49 |
| siRNA38 | NC_001147.6..944303..944413 | -1.026 | 8.087 | 0.622 | 0.430 | 79.09 | 245.46 | 0.00 | 141.74 |
| siRNA38 | NC_001147.6..251402..251522 | 0.368 | 8.595 | 0.136 | 0.713 | 395.45 | 0.00 | 160.26 | 354.36 |
| siRNA39 | NC_001139.9..623387..623506 | 0.454 | 8.447 | 0.196 | 0.658 | 79.09 | 245.46 | 160.26 | 283.49 |
| siRNA43 | NC_001137.3..257116..257220 | 1.984 | 8.082 | 2.096 | 0.148 | 79.09 | 0.00 | 160.26 | 212.61 |

**Table S4. Human mRNA target prediction of the identified yeast EV-derived miRNA sequences**

| **Target mRNA (psRNAtarget)** | | | | | | | | | | | | | | | |
| --- | --- | --- | --- | --- | --- | --- | --- | --- | --- | --- | --- | --- | --- | --- | --- |
| **logFC** | **miRNA_Acc.** | **Target GeneID** | **Gene Symbol** | **Expectation** | **UPE$** | **miRNA_start** | **miRNA_end** | **Target_start** | **Target_end** | **miRNA_aligned_fragment** | **alignment** | **Target_aligned_fragment** | **Inhibition** | **Target_Desc.** | **Multiplicity** |
| -7.29 | GGUAAAAUCCAACGUUGCC | NM_001010851 | ZNF766 | 3 | 18.621 | 1 | 19 | 1469 | 1488 | GGUAAAAUCCAAC-GUUGCC | ::::: :::::::::: .: | UGCAACUGUUGGAUUUUCUC | Cleavage | zinc finger protein 766 | 1 |
| -7.29 | GGUAAAAUCCAACGUUGCC | NM_001143958 | TMEM30A | 2.5 | 15.515 | 1 | 19 | 2418 | 2435 | GGUAAAAUCCAACGUUGCC | :.::::::: ::::::.. | UGUAACGUUG-AUUUUAUU | Translation | transmembrane protein 30A | 1 |
| -7.29 | GGUAAAAUCCAACGUUGCC | NM_018057 | SLC6A15 | 3 | 13.767 | 1 | 19 | 2242 | 2260 | GGUAAAAUCCAACGUUGCC | ::::. ::::::::::. | UGCAAUACUGGAUUUUACU | Cleavage | solute carrier family 6 member 15 | 1 |
| -7.29 | GGUAAAAUCCAACGUUGCC | NM_003012 | SFRP1 | 3 | 20.629 | 1 | 19 | 2228 | 2246 | GGUAAAAUCCAACGUUGCC | :::.: :::.::::::: | CGCAGCUGUGGGUUUUACC | Cleavage | secreted frizzled related protein 1 | 1 |
| -7.29 | GGUAAAAUCCAACGUUGCC | NM_183353 | RLIM | 3 | 14.045 | 1 | 19 | 5228 | 5247 | GGUAAAAUCC-AACGUUGCC | ::..::: :::::::::: | UCCAGUGUUUGGAUUUUACC | Translation | ring finger protein, LIM domain interacting | 1 |
| -7.29 | GGUAAAAUCCAACGUUGCC | NM_002874 | RAD23B | 3 | 14.472 | 1 | 19 | 1234 | 1252 | GGUAAAAUCCAACGUUGCC | :::..::::: :::::.. | AGCAGUGUUGGCUUUUAUU | Cleavage | RAD23 homolog B, nucleotide excision repair protein | 1 |
| -7.29 | GGUAAAAUCCAACGUUGCC | NM_005049 | PWP2 | 2.5 | 17.7 | 1 | 19 | 12 | 30 | GGUAAAAUCCAACGUUGCC | :::.::::::::: :.:: | UGCAGCGUUGGAUUGUGCC | Cleavage | PWP2 small subunit processome component | 1 |
| -7.29 | GGUAAAAUCCAACGUUGCC | NM_020957 | PCDHB16 | 3 | 22.69 | 1 | 19 | 693 | 712 | GGUAAAAUC-CAACGUUGCC | :::.:: :: ::::::.:: | UGCAGCGAUGAGAUUUUGCC | Translation | protocadherin beta 16 | 1 |
| -7.29 | GGUAAAAUCCAACGUUGCC | NM_178812 | MTDH | 3 | 16.091 | 1 | 19 | 1911 | 1929 | GGUAAAAUCCAACGUUGCC | ::::::::::::::.. | UCAAACGUUGGAUUUUAUU | Cleavage | metadherin | 1 |
| -7.29 | GGUAAAAUCCAACGUUGCC | NM_015329 | MAU2 | 2.5 | 21.812 | 1 | 19 | 1044 | 1062 | GGUAAAAUCCAACGUUGCC | :::.:::: :.:::::.: | AGCAGCGUUUGGUUUUAUC | Translation | MAU2 sister chromatid cohesion factor | 1 |
| -7.29 | GGUAAAAUCCAACGUUGCC | NM_001160147 | DDHD1 | 2.5 | 15.581 | 1 | 19 | 8766 | 8784 | GGUAAAAUCCAACGUUGCC | : .:.:::::::::::.: | AGGGAUGUUGGAUUUUAUC | Cleavage | DDHD domain containing 1 | 1 |
| -7.29 | GGUAAAAUCCAACGUUGCC | NM_001242377 | DCP2 | 3 | 14.354 | 1 | 19 | 6836 | 6854 | GGUAAAAUCCAACGUUGCC | ::::.::: .::::::: | AGCAAUGUUUUGUUUUACC | Translation | decapping mRNA 2 | 1 |
| -7.29 | GGUAAAAUCCAACGUUGCC | NM_018235 | CNDP2 | 3 | 14.903 | 1 | 19 | 1548 | 1567 | GGU-AAAAUCCAACGUUGCC | :.:..:::::::::: ::: | AGUAGUGUUGGAUUUUUACC | Cleavage | carnosine dipeptidase 2 | 1 |
| -7.29 | GGUAAAAUCCAACGUUGCC | NM_001204 | BMPR2 | 3 | 14.086 | 1 | 19 | 6228 | 6247 | GGUAAAAUCCA-ACGUUGCC | :::::: : :::.:::::.: | GGCAACAUGUGGGUUUUAUC | Translation | bone morphogenetic protein receptor type 2 | 1 |
| -7.29 | GGUAAAAUCCAACGUUGCC | NM_005502 | ABCA1 | 3 | 16.63 | 1 | 19 | 2083 | 2101 | GGUAAAAUCCAACGUUGCC | :::: ::::: ::::::: | GGCAUCGUUGCAUUUUACA | Translation | ATP binding cassette subfamily A member 1 | 1 |
| -5.26 | UCACUUGACCCUGUCGGUAAG | NM_001164825 | SMIM12 | 2.5 | 22.589 | 1 | 21 | 2856 | 2877 | UCACUUGACC-CUGUCGGUAAG | :.:.::::: :::::.:::: | AGUGCUGACAGAGGUCAGGUGA | Translation | small integral membrane protein 12 | 1 |
| -5.26 | UCACUUGACCCUGUCGGUAAG | NM_052859 | RFT1 | 3 | 24.609 | 1 | 21 | 1535 | 1555 | UCACUUGACCCUGUCGGUAAG | :: :.:::::::::::. | UGGUCCCAUAGGGUCAAGUGG | Cleavage | RFT1 homolog | 1 |
| -5.26 | UCACUUGACCCUGUCGGUAAG | NM_016200 | LSM8 | 3 | 19.891 | 1 | 21 | 2898 | 2918 | UCACUUGACCCUGUCGGUAAG | .: .::::::::::::: | AGGCUCAGCAGGGUCAAGUGA | Cleavage | LSM8 homolog, U6 small nuclear RNA associated | 1 |
| -5.26 | UCACUUGACCCUGUCGGUAAG | NM_001098722 | GNG4 | 3 | 15.728 | 1 | 21 | 1374 | 1394 | UCACUUGACCCUGUCGGUAAG | ::. .:::: :::::::: | UCCACUCGCAGGUUCAAGUGA | Translation | G protein subunit gamma 4 | 1 |
| -5.26 | UCACUUGACCCUGUCGGUAAG | NM_001340 | CYLC2 | 3 | 17.942 | 1 | 21 | 758 | 778 | UCACUUGACCCUGUCGGUAAG | ::: :::: :::::::: | UCCACCUUCAGGUUCAAGUGA | Translation | cylicin 2 | 1 |
| -5.26 | UCACUUGACCCUGUCGGUAAG | NM_032043 | BRIP1 | 3 | 22.701 | 1 | 21 | 231 | 251 | UCACUUGACCCUGUCGGUAAG | .: ::: :::: :::::::: | UUCACCUCCAGGUUCAAGUGA | Translation | BRCA1 interacting helicase 1 | 1 |
| -5.26 | UCACUUGACCCUGUCGGUAAG | NM_014924 | ATG14 | 3 | 17.012 | 1 | 21 | 2596 | 2614 | UCACUUGACCCUGUCGGUAAG | .:::: :.:::::.:::::: | UUUAC--AUAGGGUUAAGUGA | Cleavage | autophagy related 14 | 1 |
| -5.09 | UAAAAGAUUAGGCAAGAAG | NM_001267828 | ZNF839 | 3 | 14.46 | 1 | 19 | 479 | 497 | UAAAAGAUUAGGCAAGAAG | :: ::::: :.::.::::: | CUACUUGCAUGAUUUUUUA | Translation | zinc finger protein 839 | 1 |
| -5.09 | UAAAAGAUUAGGCAAGAAG | NM_001277816 | YTHDF3 | 3 | 19.4 | 1 | 19 | 2425 | 2443 | UAAAAGAUUAGGCAAGAAG | .:::::::.:::: :::: | UUUCUUGCUUAAUGUUUUU | Cleavage | YTH N6-methyladenosine RNA binding protein F3 | 1 |
| -5.09 | UAAAAGAUUAGGCAAGAAG | NM_001130483 | VRK2 | 3 | 9.485 | 1 | 19 | 362 | 380 | UAAAAGAUUAGGCAAGAAG | .:::::::.: ::.:::: | UUUCUUGCUUUAUUUUUUC | Translation | VRK serine/threonine kinase 2 | 1 |
| -5.09 | UAAAAGAUUAGGCAAGAAG | NM_133462 | TTC14 | 3 | 6.625 | 1 | 19 | 490 | 508 | UAAAAGAUUAGGCAAGAAG | .::.::::: :::.:::: | UUUUUUGCCCAAUUUUUUU | Translation | tetratricopeptide repeat domain 14 | 1 |
| -5.09 | UAAAAGAUUAGGCAAGAAG | NM_001042601 | TTC14 | 3 | 6.625 | 1 | 19 | 3237 | 3255 | UAAAAGAUUAGGCAAGAAG | .::.::::: :::.:::: | UUUUUUGCCCAAUUUUUUU | Translation | tetratricopeptide repeat domain 14 | 1 |
| -5.09 | UAAAAGAUUAGGCAAGAAG | NM_019022 | TMX3 | 2.5 | 20.024 | 1 | 19 | 2458 | 2476 | UAAAAGAUUAGGCAAGAAG | ::.:::::: ::::::: | AUUUUUGCCUUCUCUUUUA | Translation | thioredoxin related transmembrane protein 3 | 1 |
| -5.09 | UAAAAGAUUAGGCAAGAAG | NM_001134232 | TMEM106B | 2.5 | 8.545 | 1 | 19 | 2159 | 2178 | UAAAAGAU-UAGGCAAGAAG | .:::::::.:. .::::::: | UUUCUUGCUUGUGUCUUUUA | Cleavage | transmembrane protein 106B | 1 |
| -5.09 | UAAAAGAUUAGGCAAGAAG | NM_020375 | TIGAR | 3 | 10.196 | 1 | 19 | 6505 | 6523 | UAAAAGAUUAGGCAAGAAG | ::::::::::: :. :::: | CUUCUUGCCUACUUAUUUA | Cleavage | TP53 induced glycolysis regulatory phosphatase | 1 |
| -5.09 | UAAAAGAUUAGGCAAGAAG | NM_025142 | TAOK1 | 3 | 15.262 | 1 | 19 | 8303 | 8321 | UAAAAGAUUAGGCAAGAAG | .::::::::: :.::::. | UUUCUUGCCUCCUUUUUUG | Translation | TAO kinase 1 | 1 |
| -5.09 | UAAAAGAUUAGGCAAGAAG | NM_177402 | SYT2 | 3 | 10.936 | 1 | 19 | 6084 | 6102 | UAAAAGAUUAGGCAAGAAG | ::::::.::.:::: ::. | GUUCUUGUCUGAUCUCUUG | Cleavage | synaptotagmin 2 | 1 |
| -5.09 | UAAAAGAUUAGGCAAGAAG | NM_014980 | STXBP5L | 3 | 15.967 | 1 | 19 | 1703 | 1721 | UAAAAGAUUAGGCAAGAAG | .::.: :.:::::.::::. | UUUUUAGUCUAAUUUUUUG | Cleavage | syntaxin binding protein 5L | 1 |
| -5.09 | UAAAAGAUUAGGCAAGAAG | NM_015000 | STK38L | 3 | 14.062 | 1 | 19 | 1350 | 1368 | UAAAAGAUUAGGCAAGAAG | .:::: ::.:::: ::::: | UUUCUAGCUUAAUAUUUUA | Cleavage | serine/threonine kinase 38 like | 1 |
| -5.09 | UAAAAGAUUAGGCAAGAAG | NM_139164 | STARD4 | 2.5 | 14.447 | 1 | 19 | 351 | 369 | UAAAAGAUUAGGCAAGAAG | ::.:::..::::.::::. | AUUUUUGUUUAAUUUUUUG | Cleavage | StAR related lipid transfer domain containing 4 | 1 |
| -5.09 | UAAAAGAUUAGGCAAGAAG | NM_003144 | SSR1 | 1.5 | 14.242 | 1 | 19 | 989 | 1007 | UAAAAGAUUAGGCAAGAAG | .::::: :::::::::::. | UUUCUUACCUAAUCUUUUG | Cleavage | signal sequence receptor subunit 1 | 1 |
| -5.09 | UAAAAGAUUAGGCAAGAAG | NM_001166412 | SMOC2 | 2.5 | 11.282 | 1 | 19 | 1473 | 1491 | UAAAAGAUUAGGCAAGAAG | :::.::: :::::::::: | CUUUUUGGAUAAUCUUUUA | Translation | SPARC related modular calcium binding 2 | 1 |
| -5.09 | UAAAAGAUUAGGCAAGAAG | NM_001163438 | SMIM10 | 3 | 17.57 | 1 | 19 | 123 | 143 | UAAAAGAUUAGGCAA--GAAG | .::: :::::: :::::::: | UUUCCAUUGCCUCAUCUUUUA | Translation | small integral membrane protein 10 | 1 |
| -5.09 | UAAAAGAUUAGGCAAGAAG | NM_001184749 | SLITRK4 | 2.5 | 15.897 | 1 | 19 | 4154 | 4172 | UAAAAGAUUAGGCAAGAAG | :::::::: : ::::::. | AUUCUUGCCCAUUCUUUUG | Translation | SLIT and NTRK like family member 4 | 1 |
| -5.09 | UAAAAGAUUAGGCAAGAAG | NM_052885 | SLC2A13 | 3 | 14.331 | 1 | 19 | 1994 | 2012 | UAAAAGAUUAGGCAAGAAG | .::::: .::::: ::::: | UUUCUUAUCUAAUAUUUUA | Cleavage | solute carrier family 2 member 13 | 1 |
| -5.09 | UAAAAGAUUAGGCAAGAAG | NM_170709 | SGK3 | 3 | 8.577 | 1 | 19 | 2278 | 2296 | UAAAAGAUUAGGCAAGAAG | .::::::. :.::.::::. | UUUCUUGUAUGAUUUUUUG | Translation | serum/glucocorticoid regulated kinase family member 3 | 1 |
| -5.09 | UAAAAGAUUAGGCAAGAAG | NM_183353 | RLIM | 3 | 19.87 | 1 | 19 | 1593 | 1611 | UAAAAGAUUAGGCAAGAAG | :::::::::::::.: :: | CUUCUUGCCUAAUUUCUUU | Cleavage | ring finger protein, LIM domain interacting | 1 |
| -5.09 | UAAAAGAUUAGGCAAGAAG | NM_001136223 | RCOR3 | 3 | 16.725 | 1 | 19 | 428 | 446 | UAAAAGAUUAGGCAAGAAG | .::.:: ..::::.::::: | UUUUUUCUUUAAUUUUUUA | Cleavage | REST corepressor 3 | 1 |
| -5.09 | UAAAAGAUUAGGCAAGAAG | NM_001136224 | RCOR3 | 3 | 16.725 | 1 | 19 | 537 | 555 | UAAAAGAUUAGGCAAGAAG | .::.:: ..::::.::::: | UUUUUUCUUUAAUUUUUUA | Cleavage | REST corepressor 3 | 1 |
| -5.09 | UAAAAGAUUAGGCAAGAAG | NM_001134657 | PRR23C | 2.5 | 11.888 | 1 | 19 | 1223 | 1241 | UAAAAGAUUAGGCAAGAAG | ::.:: ..:::::::::: | AUUUUUCUUUAAUCUUUUA | Cleavage | proline rich 23C | 1 |
| -5.09 | UAAAAGAUUAGGCAAGAAG | NM_003913 | PRP4K | 1.5 | 10.34 | 1 | 19 | 2549 | 2567 | UAAAAGAUUAGGCAAGAAG | .::.:::.:::::.::::: | UUUUUUGUCUAAUUUUUUA | Cleavage | pre-mRNA processing factor kinase PRP4K | 1 |
| -5.09 | UAAAAGAUUAGGCAAGAAG | NM_013261 | PPARGC1A | 3 | 17.293 | 1 | 19 | 2406 | 2424 | UAAAAGAUUAGGCAAGAAG | : :::::.:.::.::::. | AUGCUUGCUUGAUUUUUUG | Cleavage | PPARG coactivator 1 alpha | 1 |
| -5.09 | UAAAAGAUUAGGCAAGAAG | NM_001099771 | POTEF | 3 | 13.526 | 1 | 19 | 89 | 107 | UAAAAGAUUAGGCAAGAAG | .::::::..: ::.::::. | UUUCUUGUUUCAUUUUUUG | Translation | POTE ankyrin domain family member F | 1 |
| -5.09 | UAAAAGAUUAGGCAAGAAG | NM_001083538 | POTEE | 3 | 13.161 | 1 | 19 | 89 | 107 | UAAAAGAUUAGGCAAGAAG | .::::::..: ::.::::. | UUUCUUGUUUCAUUUUUUG | Translation | POTE ankyrin domain family member E | 1 |
| -5.09 | UAAAAGAUUAGGCAAGAAG | NM_001018111 | PODXL | 3 | 13.191 | 1 | 19 | 3104 | 3122 | UAAAAGAUUAGGCAAGAAG | ::.::::.:.::.:::: | GUUUUUGCUUGAUUUUUUU | Cleavage | podocalyxin like | 1 |
| -5.09 | UAAAAGAUUAGGCAAGAAG | NM_002641 | PIGA | 2.5 | 5.947 | 1 | 19 | 1646 | 1664 | UAAAAGAUUAGGCAAGAAG | ::.::::.::::.:::: | AUUUUUGCUUAAUUUUUUU | Cleavage | phosphatidylinositol glycan anchor biosynthesis class A | 1 |
| -5.09 | UAAAAGAUUAGGCAAGAAG | NM_004911 | PDIA4 | 3 | 8.627 | 1 | 19 | 110 | 128 | UAAAAGAUUAGGCAAGAAG | .::.:: ..::::.::::: | UUUUUUUUUUAAUUUUUUA | Cleavage | protein disulfide isomerase family A member 4 | 1 |
| -5.09 | UAAAAGAUUAGGCAAGAAG | NM_006315 | PCGF3 | 3 | 13.056 | 1 | 19 | 3888 | 3906 | UAAAAGAUUAGGCAAGAAG | ::.:::.: :::::::: | GUUUUUGUCAAAUCUUUUU | Translation | polycomb group ring finger 3 | 1 |
| -5.09 | UAAAAGAUUAGGCAAGAAG | NM_018934 | PCDHB14 | 2 | 14.074 | 1 | 19 | 54 | 72 | UAAAAGAUUAGGCAAGAAG | :::.:::: :::::::::. | CUUUUUGCAUAAUCUUUUG | Translation | protocadherin beta 14 | 1 |
| -5.09 | UAAAAGAUUAGGCAAGAAG | NM_080832 | PABPC5 | 2.5 | 9.79 | 1 | 19 | 522 | 540 | UAAAAGAUUAGGCAAGAAG | ::::::..: ::.::::: | AUUCUUGUUUCAUUUUUUA | Translation | poly(A) binding protein cytoplasmic 5 | 1 |
| -5.09 | UAAAAGAUUAGGCAAGAAG | NM_030959 | OR12D3 | 3 | 12.486 | 1 | 19 | 404 | 422 | UAAAAGAUUAGGCAAGAAG | ::.::::::: :.:::: | AUUUUUGCCUAUUUUUUUU | Cleavage | olfactory receptor family 12 subfamily D member 3 | 1 |
| -5.09 | UAAAAGAUUAGGCAAGAAG | NM_001204259 | NR3C1 | 2.5 | 11.559 | 1 | 19 | 563 | 581 | UAAAAGAUUAGGCAAGAAG | .::::: : ::::.::::: | UUUCUUACAUAAUUUUUUA | Translation | nuclear receptor subfamily 3 group C member 1 | 1 |
| -5.09 | UAAAAGAUUAGGCAAGAAG | NM_002505 | NFYA | 3 | 13.968 | 1 | 19 | 2013 | 2031 | UAAAAGAUUAGGCAAGAAG | ::: ::::. :::.::::: | CUUGUUGCUGAAUUUUUUA | Translation | nuclear transcription factor Y subunit alpha | 1 |
| -5.09 | UAAAAGAUUAGGCAAGAAG | NM_025107 | MYCT1 | 2 | 17.39 | 1 | 19 | 27 | 45 | UAAAAGAUUAGGCAAGAAG | .::::::.:: .::::::: | UUUCUUGUCUUGUCUUUUA | Translation | MYC target 1 | 1 |
| -5.09 | UAAAAGAUUAGGCAAGAAG | NM_001145354 | MKLN1 | 2.5 | 13.602 | 1 | 19 | 257 | 275 | UAAAAGAUUAGGCAAGAAG | .::::: :.: :::::::: | UUUCUUACUUUAUCUUUUA | Translation | muskelin 1 | 1 |
| -5.09 | UAAAAGAUUAGGCAAGAAG | NM_152599 | MFSD6L | 2.5 | 14.723 | 1 | 19 | 193 | 210 | UAAAAGAUUAGGCAAGAAG | :::.::::: :::::::: | CUUUUUGCC-AAUCUUUUU | Translation | major facilitator superfamily domain containing 6 like | 1 |
| -5.09 | UAAAAGAUUAGGCAAGAAG | NM_015274 | MAN2B2 | 3 | 13.222 | 1 | 19 | 151 | 169 | UAAAAGAUUAGGCAAGAAG | ::.:: ::::::.:::: | GUUUUUCCCUAAUUUUUUU | Cleavage | mannosidase alpha class 2B member 2 | 1 |
| -5.09 | UAAAAGAUUAGGCAAGAAG | NM_001136049 | LMLN | 2.5 | 6.466 | 1 | 19 | 3245 | 3263 | UAAAAGAUUAGGCAAGAAG | ::.::::.: ::.::::: | AUUUUUGCUUUAUUUUUUA | Translation | leishmanolysin like peptidase | 1 |
| -5.09 | UAAAAGAUUAGGCAAGAAG | NM_001170808 | LARP4 | 2 | 9.052 | 1 | 19 | 3512 | 3530 | UAAAAGAUUAGGCAAGAAG | ::.:: ::::::.::::: | AUUUUUUCCUAAUUUUUUA | Cleavage | La ribonucleoprotein 4 | 1 |
| -5.09 | UAAAAGAUUAGGCAAGAAG | NM_014713 | LAPTM4A | 3 | 13.072 | 1 | 19 | 175 | 193 | UAAAAGAUUAGGCAAGAAG | .::.:::.. :.::::::: | UUUUUUGUUAAGUCUUUUA | Translation | lysosomal protein transmembrane 4 alpha | 1 |
| -5.09 | UAAAAGAUUAGGCAAGAAG | NM_013995 | LAMP2 | 3 | 10.449 | 1 | 19 | 668 | 686 | UAAAAGAUUAGGCAAGAAG | ::: :::..::::::::: | CUUAUUGUUUAAUCUUUUU | Cleavage | lysosomal associated membrane protein 2 | 1 |
| -5.09 | UAAAAGAUUAGGCAAGAAG | NM_001206 | KLF9 | 3 | 15.902 | 1 | 19 | 2638 | 2656 | UAAAAGAUUAGGCAAGAAG | ::.:::..:: :.::::: | AUUUUUGUUUACUUUUUUA | Cleavage | KLF transcription factor 9 | 1 |
| -5.09 | UAAAAGAUUAGGCAAGAAG | NM_001177718 | KLF11 | 3 | 15.628 | 1 | 19 | 2165 | 2183 | UAAAAGAUUAGGCAAGAAG | :.::::.:.:::::::. | AGUUUUGCUUGAUCUUUUG | Cleavage | KLF transcription factor 11 | 1 |
| -5.09 | UAAAAGAUUAGGCAAGAAG | NM_014702 | KIAA0408 | 3 | 12.23 | 1 | 19 | 641 | 659 | UAAAAGAUUAGGCAAGAAG | ::::::..:: :::::: | AUUCUUGUUUAUUCUUUUU | Cleavage | KIAA0408 | 1 |
| -5.09 | UAAAAGAUUAGGCAAGAAG | NM_001100603 | KDELR2 | 3 | 8.466 | 1 | 19 | 3 | 21 | UAAAAGAUUAGGCAAGAAG | : :::::::: ::::::: | CAUCUUGCCUUAUCUUUUU | Translation | KDEL endoplasmic reticulum protein retention receptor 2 | 1 |
| -5.09 | UAAAAGAUUAGGCAAGAAG | NM_006854 | KDELR2 | 3 | 14.867 | 1 | 19 | 178 | 196 | UAAAAGAUUAGGCAAGAAG | : :::::::: ::::::: | CAUCUUGCCUUAUCUUUUU | Translation | KDEL endoplasmic reticulum protein retention receptor 2 | 1 |
| -5.09 | UAAAAGAUUAGGCAAGAAG | NM_004519 | KCNQ3 | 2 | 7.267 | 1 | 19 | 8328 | 8346 | UAAAAGAUUAGGCAAGAAG | ::::: :::.::.::::: | AUUCUUACCUGAUUUUUUA | Cleavage | potassium voltage-gated channel subfamily Q member 3 | 1 |
| -5.09 | UAAAAGAUUAGGCAAGAAG | NM_003740 | KCNK5 | 3 | 9.517 | 1 | 19 | 1320 | 1338 | UAAAAGAUUAGGCAAGAAG | .::.:::..: ::.::::: | UUUUUUGUUUUAUUUUUUA | Translation | potassium two pore domain channel subfamily K member 5 | 1 |
| -5.09 | UAAAAGAUUAGGCAAGAAG | NM_001042705 | IQCJ | 3 | 16.565 | 1 | 19 | 182 | 200 | UAAAAGAUUAGGCAAGAAG | : ::::::.:.::::::: | CCUCUUGCUUGAUCUUUUC | Cleavage | IQ motif containing J | 1 |
| -5.09 | UAAAAGAUUAGGCAAGAAG | NM_001031853 | INSC | 2.5 | 5.355 | 1 | 19 | 314 | 332 | UAAAAGAUUAGGCAAGAAG | :::::::: ::.::::: | AAUCUUGCCUCAUUUUUUA | Translation | INSC spindle orientation adaptor protein | 1 |
| -5.09 | UAAAAGAUUAGGCAAGAAG | NM_002190 | IL17A | 2.5 | 9.595 | 1 | 19 | 229 | 247 | UAAAAGAUUAGGCAAGAAG | :::::::..:: :.::::: | CUUCUUGUUUACUUUUUUA | Cleavage | interleukin 17A | 1 |
| -5.09 | UAAAAGAUUAGGCAAGAAG | NM_016126 | IFT25 | 3 | 11.143 | 1 | 19 | 13 | 31 | UAAAAGAUUAGGCAAGAAG | :::::: :.::.::::: | GCUCUUGCAUGAUUUUUUA | Translation | intraflagellar transport 25 | 1 |
| -5.09 | UAAAAGAUUAGGCAAGAAG | NM_016217 | HECA | 3 | 16.848 | 1 | 19 | 3377 | 3395 | UAAAAGAUUAGGCAAGAAG | ::::::..::.: ::::: | GUUCUUGUUUAGUGUUUUA | Cleavage | hdc homolog, cell cycle regulator | 1 |
| -5.09 | UAAAAGAUUAGGCAAGAAG | NM_005160 | GRK3 | 3 | 13.463 | 1 | 19 | 2403 | 2421 | UAAAAGAUUAGGCAAGAAG | ::.:: ..::::.::::: | GUUUUUUUUUAAUUUUUUA | Cleavage | G protein-coupled receptor kinase 3 | 1 |
| -5.09 | UAAAAGAUUAGGCAAGAAG | NM_006496 | GNAI3 | 3 | 8.706 | 1 | 19 | 3165 | 3183 | UAAAAGAUUAGGCAAGAAG | :::::::::: .:.:::: | CUUCUUGCCUUGUUUUUUU | Translation | G protein subunit alpha i3 | 1 |
| -5.09 | UAAAAGAUUAGGCAAGAAG | NM_015123 | FRMD4B | 2.5 | 12.277 | 1 | 19 | 1510 | 1528 | UAAAAGAUUAGGCAAGAAG | :::.:::: :: ::::::: | CUUUUUGCAUAUUCUUUUA | Translation | FERM domain containing 4B | 1 |
| -5.09 | UAAAAGAUUAGGCAAGAAG | NM_020066 | FMN2 | 3 | 10.934 | 1 | 19 | 109 | 127 | UAAAAGAUUAGGCAAGAAG | : :::::: ::::::::: | CCUCUUGCAUAAUCUUUUU | Translation | formin 2 | 1 |
| -5.09 | UAAAAGAUUAGGCAAGAAG | NM_017946 | FKBP14 | 2.5 | 11.926 | 1 | 19 | 87 | 105 | UAAAAGAUUAGGCAAGAAG | .:::::::.: .:.::::: | UUUCUUGCUUUGUUUUUUA | Translation | FKBP prolyl isomerase 14 | 1 |
| -5.09 | UAAAAGAUUAGGCAAGAAG | NM_198488 | FAM83H | 2 | 16.253 | 1 | 19 | 1394 | 1412 | UAAAAGAUUAGGCAAGAAG | :::::: :: :::::::: | GUUCUUGGCUUAUCUUUUA | Translation | family with sequence similarity 83 member H | 1 |
| -5.09 | UAAAAGAUUAGGCAAGAAG | NM_175876 | EXOC8 | 3 | 14.464 | 1 | 19 | 1390 | 1408 | UAAAAGAUUAGGCAAGAAG | ::.:::..: ::.::::: | AUUUUUGUUUCAUUUUUUA | Translation | exocyst complex component 8 | 1 |
| -5.09 | UAAAAGAUUAGGCAAGAAG | NM_212554 | EEF1AKMT2 | 2.5 | 10.867 | 1 | 19 | 1364 | 1382 | UAAAAGAUUAGGCAAGAAG | ::.:::: ::::.::::. | AUUUUUGCAUAAUUUUUUG | Translation | EEF1A lysine methyltransferase 2 | 1 |
| -5.09 | UAAAAGAUUAGGCAAGAAG | NM_000104 | CYP1B1 | 3 | 11.62 | 1 | 19 | 2900 | 2918 | UAAAAGAUUAGGCAAGAAG | :::::: ..::::::::: | CUUCUUAUUUAAUCUUUUC | Cleavage | cytochrome P450 family 1 subfamily B member 1 | 1 |
| -5.09 | UAAAAGAUUAGGCAAGAAG | NM_001202543 | CUX1 | 3 | 17.381 | 1 | 19 | 2311 | 2329 | UAAAAGAUUAGGCAAGAAG | .::::::: :.:: ::::: | UUUCUUGCAUGAUGUUUUA | Translation | cut like homeobox 1 | 1 |
| -5.09 | UAAAAGAUUAGGCAAGAAG | NM_001164883 | CTNNA2 | 2.5 | 12.32 | 1 | 19 | 923 | 941 | UAAAAGAUUAGGCAAGAAG | .::::: ..::::.::::: | UUUCUUUUUUAAUUUUUUA | Cleavage | catenin alpha 2 | 1 |
| -5.09 | UAAAAGAUUAGGCAAGAAG | NM_020722 | CRACD | 3 | 10.4 | 1 | 19 | 2482 | 2500 | UAAAAGAUUAGGCAAGAAG | .::.:::..:: :.::::: | UUUUUUGUUUACUUUUUUA | Cleavage | capping protein inhibiting regulator of actin dynamics | 1 |
| -5.09 | UAAAAGAUUAGGCAAGAAG | NM_153264 | COL6A5 | 2.5 | 13.269 | 1 | 19 | 633 | 652 | UAAAAGAUUA-GGCAAGAAG | .::.::::. :::::::::. | UUUUUUGCUGUAAUCUUUUG | Translation | collagen type VI alpha 5 chain | 1 |
| -5.09 | UAAAAGAUUAGGCAAGAAG | NM_152890 | COL24A1 | 3 | 8.715 | 1 | 19 | 785 | 803 | UAAAAGAUUAGGCAAGAAG | ::.:::..: ::.::::: | GUUUUUGUUUUAUUUUUUA | Translation | collagen type XXIV alpha 1 chain | 1 |
| -5.09 | UAAAAGAUUAGGCAAGAAG | NM_206808 | CLYBL | 3 | 9.65 | 1 | 19 | 2237 | 2255 | UAAAAGAUUAGGCAAGAAG | .::.:::: ::::.:::: | UUUUUUGCAUAAUUUUUUC | Translation | citramalyl-CoA lyase | 1 |
| -5.09 | UAAAAGAUUAGGCAAGAAG | NM_015226 | CLEC16A | 3 | 19.77 | 1 | 19 | 1876 | 1894 | UAAAAGAUUAGGCAAGAAG | : ::::.::::.::::: | UGUGUUGCUUAAUUUUUUA | Cleavage | C-type lectin domain containing 16A | 1 |
| -5.09 | UAAAAGAUUAGGCAAGAAG | NM_001013625 | CFAP126 | 2.5 | 8.404 | 1 | 19 | 216 | 234 | UAAAAGAUUAGGCAAGAAG | .::.:::.. ::::::::: | UUUUUUGUUGAAUCUUUUA | Translation | cilia and flagella associated protein 126 | 1 |
| -5.09 | UAAAAGAUUAGGCAAGAAG | NM_001257160 | CEP41 | 2.5 | 19.009 | 1 | 19 | 2370 | 2388 | UAAAAGAUUAGGCAAGAAG | :::::: ::.::::::: | GUUCUUGGAUAGUCUUUUA | Translation | centrosomal protein 41 | 1 |
| -5.09 | UAAAAGAUUAGGCAAGAAG | NM_001100624 | CENPN | 3 | 5.646 | 1 | 19 | 730 | 748 | UAAAAGAUUAGGCAAGAAG | .::: : .:::::.::::: | UUUCCUUUCUAAUUUUUUA | Cleavage | centromere protein N | 1 |
| -5.09 | UAAAAGAUUAGGCAAGAAG | NM_001001548 | CD36 | 2.5 | 16.899 | 1 | 19 | 769 | 787 | UAAAAGAUUAGGCAAGAAG | :::: : :::::.::::: | GUUCUAGGCUAAUUUUUUA | Cleavage | CD36 molecule (CD36 blood group) | 1 |
| -5.09 | UAAAAGAUUAGGCAAGAAG | NM_005436 | CCDC6 | 3 | 9.132 | 1 | 19 | 3467 | 3485 | UAAAAGAUUAGGCAAGAAG | .::::: ..::::.::::. | UUUCUUCUUUAAUUUUUUG | Cleavage | coiled-coil domain containing 6 | 1 |
| -5.09 | UAAAAGAUUAGGCAAGAAG | NM_030939 | C6orf62 | 3 | 20.209 | 1 | 19 | 566 | 584 | UAAAAGAUUAGGCAAGAAG | ::.:::::: :: ::::: | AUUUUUGCCUCAUAUUUUA | Translation | chromosome 6 open reading frame 62 | 1 |
| -5.09 | UAAAAGAUUAGGCAAGAAG | NM_017860 | C1orf56 | 2.5 | 10.876 | 1 | 19 | 525 | 543 | UAAAAGAUUAGGCAAGAAG | :.::::.::::.::::: | UAUUUUGCUUAAUUUUUUA | Cleavage | chromosome 1 open reading frame 56 | 1 |
| -5.09 | UAAAAGAUUAGGCAAGAAG | NM_001174123 | C18orf63 | 2 | 10.119 | 1 | 19 | 461 | 480 | UAAAAGAUUA-GGCAAGAAG | .::.::::. :::::::::: | UUUUUUGCUAUAAUCUUUUA | Translation | chromosome 18 open reading frame 63 | 1 |
| -5.09 | UAAAAGAUUAGGCAAGAAG | NM_022344 | C17orf75 | 3 | 15.119 | 1 | 19 | 2856 | 2874 | UAAAAGAUUAGGCAAGAAG | ::.:::..: ::.::::: | GUUUUUGUUUUAUUUUUUA | Translation | chromosome 17 open reading frame 75 | 1 |
| -5.09 | UAAAAGAUUAGGCAAGAAG | NM_017552 | ATAD2B | 3 | 5.366 | 1 | 19 | 1708 | 1726 | UAAAAGAUUAGGCAAGAAG | :::: ::.::::.:::: | AUUCUAGCUUAAUUUUUUU | Cleavage | ATPase family AAA domain containing 2B | 1 |
| -5.09 | UAAAAGAUUAGGCAAGAAG | NM_006420 | ARFGEF2 | 3 | 16.976 | 1 | 19 | 1627 | 1645 | UAAAAGAUUAGGCAAGAAG | ::::::.::: :.:::: | AUUCUUGUCUACUUUUUUC | Cleavage | ADP ribosylation factor guanine nucleotide exchange factor 2 | 1 |
| -5.09 | UAAAAGAUUAGGCAAGAAG | NM_001105193 | AMER3 | 3 | 12.92 | 1 | 19 | 479 | 497 | UAAAAGAUUAGGCAAGAAG | ::.:: ..::::.::::: | AUUUUUUUUUAAUUUUUUA | Cleavage | APC membrane recruitment protein 3 | 1 |
| -5.09 | UAAAAGAUUAGGCAAGAAG | NM_207413 | ALKAL1 | 2.5 | 17.528 | 1 | 19 | 174 | 192 | UAAAAGAUUAGGCAAGAAG | .:::::::.: ::::::: | UUUCUUGCUUUAUCUUUUU | Translation | ALK and LTK ligand 1 | 1 |
| -5.09 | UAAAAGAUUAGGCAAGAAG | NM_001626 | AKT2 | 3 | 13.217 | 1 | 19 | 3301 | 3319 | UAAAAGAUUAGGCAAGAAG | :::.:::. ::::::::: | CUUUUUGUGUAAUCUUUUC | Translation | AKT serine/threonine kinase 2 | 1 |
| -5.09 | UAAAAGAUUAGGCAAGAAG | NM_001628 | AKR1B1 | 2.5 | 10.183 | 1 | 19 | 43 | 61 | UAAAAGAUUAGGCAAGAAG | .::::::::: ::.:::: | UUUCUUGCCUCAUUUUUUU | Translation | aldo-keto reductase family 1 member B | 1 |
| -5.09 | UAAAAGAUUAGGCAAGAAG | NM_014911 | AAK1 | 3 | 15.834 | 1 | 19 | 6091 | 6109 | UAAAAGAUUAGGCAAGAAG | ::::::::.::::.:: :. | CUUCUUGCUUAAUUUUAUG | Cleavage | AP2 associated kinase 1 | 1 |
| -5.09 | CUCACCACUAUUAGAAUC | NM_003033 | ST3GAL1 | 3 | 23.186 | 1 | 18 | 971 | 988 | CUCACCACUAUUAGAAUC | :.::::::: :::: ::: | GGUUCUAAUCGUGGCGAG | Translation | ST3 beta-galactoside alpha-2,3-sialyltransferase 1 | 1 |
| -5.09 | CUCACCACUAUUAGAAUC | NM_012234 | RYBP | 3 | 13.525 | 1 | 18 | 2104 | 2121 | CUCACCACUAUUAGAAUC | :::.::::: :::::.: | CAUUUUAAUAUUGGUGGG | Cleavage | RING1 and YY1 binding protein | 1 |
| -5.09 | CUCACCACUAUUAGAAUC | NM_032015 | RNF26 | 3 | 18.757 | 1 | 18 | 207 | 224 | CUCACCACUAUUAGAAUC | ::: ::.: .:::::::: | GAUGCUGAGGGUGGUGAG | Translation | ring finger protein 26 | 1 |
| -5.09 | CUCACCACUAUUAGAAUC | NM_018717 | MAML3 | 3 | 15.531 | 1 | 18 | 2129 | 2145 | CUCACCACUAUUAGAAUC | ::::.::::: :::::. | GAUUUUAAUA-UGGUGGU | Cleavage | mastermind like transcriptional coactivator 3 | 1 |
| -5.09 | CUCACCACUAUUAGAAUC | NM_002268 | KPNA4 | 2.5 | 14.388 | 1 | 18 | 6201 | 6219 | CUCACCACUAUUA-GAAUC | ::::: :::: ::::::.: | GAUUCAUAAUUGUGGUGGG | Translation | karyopherin subunit alpha 4 | 1 |
| -5.09 | CUCACCACUAUUAGAAUC | NM_205833 | IGSF1 | 3 | 15.102 | 1 | 18 | 363 | 381 | CUCACCACUAUUAG-AAUC | :::: :::.:. ::::::: | GAUUGCUAGUGUUGGUGAG | Cleavage | immunoglobulin superfamily member 1 | 1 |
| -5.09 | CUCACCACUAUUAGAAUC | NM_000829 | GRIA4 | 2.5 | 15.442 | 1 | 18 | 1042 | 1059 | CUCACCACUAUUAGAAUC | :::: :: ::::::::.: | GAUUGUAUUAGUGGUGGG | Translation | glutamate ionotropic receptor AMPA type subunit 4 | 1 |
| -5.09 | CUCACCACUAUUAGAAUC | NM_001077243 | GRIA4 | 2.5 | 15.442 | 1 | 18 | 1209 | 1226 | CUCACCACUAUUAGAAUC | :::: :: ::::::::.: | GAUUGUAUUAGUGGUGGG | Translation | glutamate ionotropic receptor AMPA type subunit 4 | 1 |
| -5.09 | CUCACCACUAUUAGAAUC | NM_001040061 | FOXL2NB | 2.5 | 24.729 | 1 | 18 | 1053 | 1069 | CUCACCACUAUUAGAAUC | :::::::. .::::::.: | GAUUCUAG-GGUGGUGGG | Translation | FOXL2 neighbor | 1 |
| -5.09 | CUCACCACUAUUAGAAUC | NM_001962 | EFNA5 | 3 | 21.722 | 1 | 18 | 1599 | 1616 | CUCACCACUAUUAGAAUC | : ::::: :.::::::.: | GUUUCUACUGGUGGUGGG | Translation | ephrin A5 | 1 |
| -5.09 | CUCACCACUAUUAGAAUC | NM_016651 | DACT1 | 2.5 | 13.072 | 1 | 18 | 218 | 235 | CUCACCACUAUUAGAAUC | :.::.::::.::::::: | GGUUUUAAUGGUGGUGAU | Cleavage | dishevelled binding antagonist of beta catenin 1 | 1 |
| -5.09 | CUCACCACUAUUAGAAUC | NM_003718 | CDK13 | 2.5 | 14.874 | 1 | 18 | 1975 | 1991 | CUCACCACUAUUAGAAUC | :::.::::: ::::::: | AAUUUUAAUA-UGGUGAG | Cleavage | cyclin dependent kinase 13 | 1 |
| -5.09 | CUCACCACUAUUAGAAUC | NM_032991 | CASP3 | 3 | 13.573 | 1 | 18 | 1110 | 1127 | CUCACCACUAUUAGAAUC | .:::::: .:::::::: | UGUUCUAAAGGUGGUGAG | Translation | caspase 3 | 1 |
| -5.09 | CUCACCACUAUUAGAAUC | NM_031895 | CACNG8 | 2.5 | 17.827 | 1 | 18 | 1077 | 1094 | CUCACCACUAUUAGAAUC | ::::::. ::::::::: | UAUUCUAGGAGUGGUGAG | Translation | calcium voltage-gated channel auxiliary subunit gamma 8 | 1 |
| -5.09 | CUCACCACUAUUAGAAUC | NM_182503 | ADAT2 | 3 | 19 | 1 | 18 | 2335 | 2352 | CUCACCACUAUUAGAAUC | .::::.: ::::::::: | AGUUCUGAAAGUGGUGAG | Translation | adenosine deaminase tRNA specific 2 | 1 |
| -4.70 | AUGUAGUCAACCAUAAUGAGGGU | NM_194314 | ZBTB41 | 3 | 17.581 | 1 | 23 | 1811 | 1833 | AUGUAGUCAACCAUAAUGAGGGU | : : :::. :::::::::.:: | UACAUGAUUGAGGUUGACUAUAU | Cleavage | zinc finger and BTB domain containing 41 | 1 |
| -4.70 | AUGUAGUCAACCAUAAUGAGGGU | NM_002834 | PTPN11 | 3 | 16.299 | 1 | 23 | 115 | 137 | AUGUAGUCAACCAUAAUGAGGGU | .:.. :: :.::::::::::: : | GCUUGCAAUGUGGUUGACUACCU | Cleavage | protein tyrosine phosphatase non-receptor type 11 | 1 |
| -4.70 | AUGUAGUCAACCAUAAUGAGGGU | NM_173566 | PRR14L | 3 | 17.36 | 1 | 23 | 3627 | 3649 | AUGUAGUCAACCAUAAUGAGGGU | : :: ::::.::: ::.::..:: | AACCACAUUGUGGGUGGCUGUAU | Translation | proline rich 14 like | 1 |
| -4.70 | AUGUAGUCAACCAUAAUGAGGGU | NM_001256080 | KLK2 | 2.5 | 17.883 | 1 | 23 | 671 | 693 | AUGUAGUCAACCAUAAUGAGGGU | .:.::::::::: ..::::: | UGGUUUAUUAUGGUUUGUUACAU | Cleavage | kallikrein related peptidase 2 | 1 |
| -4.70 | AUGUAGUCAACCAUAAUGAGGGU | NM_001002231 | KLK2 | 2.5 | 17.883 | 1 | 23 | 822 | 844 | AUGUAGUCAACCAUAAUGAGGGU | .:.::::::::: ..::::: | UGGUUUAUUAUGGUUUGUUACAU | Cleavage | kallikrein related peptidase 2 | 1 |
| -4.70 | AUGUAGUCAACCAUAAUGAGGGU | NM_139318 | KCNH5 | 3 | 14.969 | 1 | 23 | 193 | 217 | AUGUAGUCAAC--CAUAAUGAGGGU | .:::::: :::::::.::: | UUGUAUAUUAUGCAGUUGACUGCAU | Translation | potassium voltage-gated channel subfamily H member 5 | 1 |
| -4.70 | AUGUAGUCAACCAUAAUGAGGGU | NM_172375 | KCNH5 | 3 | 14.969 | 1 | 23 | 1127 | 1151 | AUGUAGUCAAC--CAUAAUGAGGGU | .:::::: :::::::.::: | UUGUAUAUUAUGCAGUUGACUGCAU | Translation | potassium voltage-gated channel subfamily H member 5 | 1 |
| -4.70 | AUGUAGUCAACCAUAAUGAGGGU | NM_001097634 | GCNT1 | 3 | 12.756 | 1 | 23 | 2110 | 2132 | AUGUAGUCAACCAUAAUGAGGGU | :: .:.:::::: :::::.::: | ACAUUUAUUAUGUGUGACUGCAU | Translation | glucosaminyl (N-acetyl) transferase 1 | 1 |
| -4.70 | AUGUAGUCAACCAUAAUGAGGGU | NM_014864 | FAM20B | 3 | 18.742 | 1 | 23 | 1841 | 1863 | AUGUAGUCAACCAUAAUGAGGGU | :: :.::::::: ::::: | AUAAACAGUGUGGUUGAAUACAU | Cleavage | FAM20B glycosaminoglycan xylosylkinase | 1 |
| -4.70 | AUGUAGUCAACCAUAAUGAGGGU | NM_001195215 | DENND1B | 3 | 18.549 | 1 | 23 | 5109 | 5131 | AUGUAGUCAACCAUAAUGAGGGU | :.:. :: : :::::::.::.:: | AUCUACACUUUGGUUGAUUAUAU | Cleavage | DENN domain containing 1B | 1 |
| -4.70 | AUGUAGUCAACCAUAAUGAGGGU | NM_005713 | CERT1 | 3 | 17.377 | 1 | 23 | 1745 | 1767 | AUGUAGUCAACCAUAAUGAGGGU | :.. :::: ::::: ::::::: | AUUAUCAUGUUGGUUUACUACAU | Cleavage | ceramide transporter 1 | 1 |
| -4.70 | AUGUAGUCAACCAUAAUGAGGGU | NM_000216 | ANOS1 | 3 | 22.553 | 1 | 23 | 3342 | 3364 | AUGUAGUCAACCAUAAUGAGGGU | :.::::::::::::: :: | GAAGUUAUUAUGGUUGACUUCAA | Cleavage | anosmin 1 | 1 |
| -4.70 | AUGUAGUCAACCAUAAUGAGGGU | NM_012199 | AGO1 | 2.5 | 14.185 | 1 | 23 | 2062 | 2084 | AUGUAGUCAACCAUAAUGAGGGU | ..:: ::::::.:::::: | GGUUCUGUUCUGGUUGGCUACAU | Cleavage | argonaute RISC component 1 | 1 |
| -3.60 | UAACACAGAUCUGUACUG | NM_181486 | TBX5 | 3 | 23.153 | 1 | 18 | 874 | 892 | UAACACAGAUC-UGUACUG | ::::.:: : ::::::::. | CAGUGCAAGCUCUGUGUUG | Translation | T-box transcription factor 5 | 1 |
| -3.60 | UAACACAGAUCUGUACUG | NM_007214 | SEC63 | 3 | 16.984 | 1 | 18 | 80 | 98 | UAACA-CAGAUCUGUACUG | ::::::::: ::: ::::. | CAGUACAGAACUGAUGUUG | Translation | SEC63 homolog, protein translocation regulator | 1 |
| -3.60 | UAACACAGAUCUGUACUG | NM_004866 | SCAMP1 | 3 | 17.514 | 1 | 18 | 3404 | 3421 | UAACACAGAUCUGUACUG | ..::::: ::::::::: | UGGUACAAAUCUGUGUUU | Translation | secretory carrier membrane protein 1 | 1 |
| -3.60 | UAACACAGAUCUGUACUG | NM_173814 | PRTG | 3 | 14.156 | 1 | 18 | 4505 | 4522 | UAACACAGAUCUGUACUG | : ::: :: ::::::::: | CCGUAGAGUUCUGUGUUA | Translation | protogenin | 1 |
| -3.60 | UAACACAGAUCUGUACUG | NM_002710 | PPP1CC | 2.5 | 17.815 | 1 | 18 | 91 | 107 | UAACACAGAUCUGUACUG | ::: :.::::::::::: | CAG-AUAGAUCUGUGUUU | Cleavage | protein phosphatase 1 catalytic subunit gamma | 1 |
| -3.60 | UAACACAGAUCUGUACUG | NM_203453 | PLPP6 | 2.5 | 14.259 | 1 | 18 | 317 | 333 | UAACACAGAUCUGUACUG | ::::: ::::.:::::: | CAGUA-AGAUUUGUGUUU | Cleavage | phospholipid phosphatase 6 | 1 |
| -3.60 | UAACACAGAUCUGUACUG | NM_001104631 | PDE4D | 2.5 | 18.455 | 1 | 18 | 696 | 713 | UAACACAGAUCUGUACUG | ::::::: ::::::: :: | CAGUACAAAUCUGUGCUA | Translation | phosphodiesterase 4D | 1 |
| -3.60 | UAACACAGAUCUGUACUG | NM_152298 | NASP | 3 | 13.809 | 1 | 18 | 204 | 221 | UAACACAGAUCUGUACUG | : :::: :::::::::: | CUGUACUGAUCUGUGUUC | Cleavage | nuclear autoantigenic sperm protein | 1 |
| -3.60 | UAACACAGAUCUGUACUG | NM_006077 | MICU1 | 2.5 | 22.912 | 1 | 18 | 519 | 536 | UAACACAGAUCUGUACUG | ::: : .::::::::::: | CAGGAAGGAUCUGUGUUA | Cleavage | mitochondrial calcium uptake 1 | 1 |
| -3.60 | UAACACAGAUCUGUACUG | NM_021629 | GNB4 | 2 | 16.02 | 1 | 18 | 1448 | 1464 | UAACACAGAUCUGUACUG | ::: :.::.::::::::: | CAG-AUAGGUCUGUGUUA | Cleavage | G protein subunit beta 4 | 1 |
| -3.60 | UAACACAGAUCUGUACUG | NM_001145666 | GLG1 | 2.5 | 22.858 | 1 | 18 | 2291 | 2309 | UAACACAGAUCUGUA-CUG | ::: :.::: ::::::::: | CAGCUGCAGUUCUGUGUUA | Translation | golgi glycoprotein 1 | 1 |
| -3.60 | UAACACAGAUCUGUACUG | NM_001145667 | GLG1 | 2.5 | 22.858 | 1 | 18 | 3402 | 3420 | UAACACAGAUCUGUA-CUG | ::: :.::: ::::::::: | CAGCUGCAGUUCUGUGUUA | Translation | golgi glycoprotein 1 | 1 |
| -3.60 | UAACACAGAUCUGUACUG | NM_000158 | GBE1 | 3 | 14.482 | 1 | 18 | 23 | 41 | UAACACAGAUCUGUA-CUG | ::: :.:::::.:::::: | CAGAUGCAGAUUUGUGUUU | Cleavage | 1,4-alpha-glucan branching enzyme 1 | 1 |
| -3.60 | UAACACAGAUCUGUACUG | NM_001242463 | FBXO32 | 3 | 23.316 | 1 | 18 | 2709 | 2726 | UAACACAGAUCUGUACUG | :: ::: :::::::::: | CACAACAUAUCUGUGUUA | Translation | F-box protein 32 | 1 |
| -3.60 | UAACACAGAUCUGUACUG | NM_014613 | FAF2 | 1 | 15.676 | 1 | 18 | 2349 | 2365 | UAACACAGAUCUGUACUG | ::::::: :::::::::: | CAGUACA-AUCUGUGUUA | Translation | Fas associated factor family member 2 | 1 |
| -3.60 | UAACACAGAUCUGUACUG | NM_001981 | EPS15 | 2.5 | 18.687 | 1 | 18 | 1393 | 1410 | UAACACAGAUCUGUACUG | ..:::. ::::::::::: | UGGUAUUGAUCUGUGUUA | Cleavage | epidermal growth factor receptor pathway substrate 15 | 1 |
| -3.60 | UAACACAGAUCUGUACUG | NM_198256 | E2F6 | 3 | 10.124 | 1 | 18 | 2057 | 2074 | UAACACAGAUCUGUACUG | : ::::::: ::::::: | AAUUACAGAUGUGUGUUA | Cleavage | E2F transcription factor 6 | 1 |
| -3.60 | UAACACAGAUCUGUACUG | NM_001944 | DSG3 | 2.5 | 12.027 | 1 | 18 | 1037 | 1056 | UAACACA-GAUCUG-UACUG | ::::: :::::: ::::::: | CAGUAACAGAUCCUGUGUUA | Cleavage | desmoglein 3 | 1 |
| -3.60 | UAACACAGAUCUGUACUG | NM_001126054 | CASK | 2.5 | 13.67 | 1 | 18 | 951 | 968 | UAACACAGAUCUGUACUG | :: : ::::::::::::. | CAUUUCAGAUCUGUGUUG | Cleavage | calcium/calmodulin dependent serine protein kinase | 1 |
| -3.60 | UAACACAGAUCUGUACUG | NM_014962 | BTBD3 | 2 | 17.92 | 1 | 18 | 2861 | 2877 | UAACACAGAUCUGUACUG | ::::.::::::: ::::: | CAGUGCAGAUCU-UGUUA | Cleavage | BTB domain containing 3 | 1 |
| -3.60 | UAACACAGAUCUGUACUG | NM_207406 | BEND4 | 1.5 | 18.711 | 1 | 18 | 4286 | 4303 | UAACACAGAUCUGUACUG | ::::::::::::: :::: | CAGUACAGAUCUGCGUUA | Cleavage | BEN domain containing 4 | 1 |
| -3.60 | UAACACAGAUCUGUACUG | NM_001159547 | BEND4 | 1.5 | 18.711 | 1 | 18 | 4494 | 4511 | UAACACAGAUCUGUACUG | ::::::::::::: :::: | CAGUACAGAUCUGCGUUA | Cleavage | BEN domain containing 4 | 1 |
| -2.99 | UGAUAACGAACGAGACCU | NM_001193304 | TMEM127 | 3 | 22.35 | 1 | 18 | 3189 | 3206 | UGAUAACGAACGAGACCU | ::::::: ::.:::.:.. | AGGUCUCUUUUGUUGUUG | Translation | transmembrane protein 127 | 1 |
| -2.99 | UGAUAACGAACGAGACCU | NM_014159 | SETD2 | 3 | 13.728 | 1 | 18 | 497 | 514 | UGAUAACGAACGAGACCU | ::::.: ::.::::::: | AGGUUUAAUUUGUUAUCA | Translation | SET domain containing 2, histone lysine methyltransferase | 1 |
| -2.99 | UGAUAACGAACGAGACCU | NM_001077397 | IRF2BP2 | 3 | 11.909 | 1 | 18 | 875 | 891 | UGAUAACGAACGAGACCU | :::::: :::.:::.:.. | AGGUCU-GUUUGUUGUUG | Cleavage | interferon regulatory factor 2 binding protein 2 | 1 |
| -2.99 | UGAUAACGAACGAGACCU | NM_032043 | BRIP1 | 3 | 11.496 | 1 | 18 | 719 | 736 | UGAUAACGAACGAGACCU | ::::.: ::::::::.: | AGGUUUAUUUCGUUAUUA | Translation | BRCA1 interacting helicase 1 | 1 |
| -2.98 | UUUCGAGACUAAACUACACUGGU | NM_001164098 | VCAN | 2.5 | 17.722 | 1 | 23 | 458 | 480 | UUUCGAGACUAAACUACACUGGU | :::::::::: :: :::: | UUUUAUGUAGUUUAGGCUGGAAA | Cleavage | versican | 1 |
| -2.98 | UUUCGAGACUAAACUACACUGGU | NM_005339 | UBE2K | 3 | 9.644 | 1 | 23 | 1723 | 1745 | UUUCGAGACUAAACUACACUGGU | :... ::::::::: :.:: ::: | AUUGCUGUAGUUUAUUUUCUAAA | Translation | ubiquitin conjugating enzyme E2 K | 1 |
| -2.98 | UUUCGAGACUAAACUACACUGGU | NM_138779 | TEX30 | 3 | 12.242 | 1 | 23 | 238 | 260 | UUUCGAGACUAAACUACACUGGU | :..::: ::::: :::.:.:::: | AUUAGUAUAGUUAAGUUUUGAAA | Translation | testis expressed 30 | 1 |
| -2.98 | UUUCGAGACUAAACUACACUGGU | NM_006931 | SLC2A3 | 3 | 21.839 | 1 | 23 | 1999 | 2020 | UUUCGAGACUAAACUACACUGGU | : :::::. ::::: ::.:::: | UCAAGUGUGCUUUAG-CUUGAAA | Cleavage | solute carrier family 2 member 3 | 1 |
| -2.98 | UUUCGAGACUAAACUACACUGGU | NM_001077239 | RNF214 | 3 | 19.893 | 1 | 23 | 248 | 270 | UUUCGAGACUAAACUACACUGGU | :: ::::::.: ::.:::: | UGAUGUUUAGUUUGGCCUUGAAA | Cleavage | ring finger protein 214 | 1 |
| -2.98 | UUUCGAGACUAAACUACACUGGU | NM_014469 | RBMXL2 | 2.5 | 13.478 | 1 | 23 | 172 | 194 | UUUCGAGACUAAACUACACUGGU | :.. :: :::::::::.:.:.:: | AUUCGUUUAGUUUAGUUUUGGAA | Cleavage | RBMX like 2 | 1 |
| -2.98 | UUUCGAGACUAAACUACACUGGU | NM_001142386 | PDK3 | 3 | 18.339 | 1 | 23 | 901 | 923 | UUUCGAGACUAAACUACACUGGU | .:: : ::::::: :.:.:::: | GCCUCUCUAGUUUAUUUUUGAAA | Translation | pyruvate dehydrogenase kinase 3 | 1 |
| -2.98 | UUUCGAGACUAAACUACACUGGU | NM_138348 | OTULIN | 3 | 19.357 | 1 | 23 | 850 | 872 | UUUCGAGACUAAACUACACUGGU | .:: :::.::::::: :.:.:: | GCCUUUGUGGUUUAGUGUUGGAA | Cleavage | OTU deubiquitinase with linear linkage specificity | 1 |
| -2.98 | UUUCGAGACUAAACUACACUGGU | NM_007006 | NUDT21 | 3 | 14.02 | 1 | 23 | 3411 | 3433 | UUUCGAGACUAAACUACACUGGU | : ::::::.::.:.::.: | UCAUUUAUAGUUUGGUUUUGAGA | Cleavage | nudix hydrolase 21 | 1 |
| -2.98 | UUUCGAGACUAAACUACACUGGU | NM_001185119 | MYNN | 3 | 18.574 | 1 | 23 | 786 | 808 | UUUCGAGACUAAACUACACUGGU | : :::: :: :::.:.:::: | GAAAAUGUACUUGAGUUUUGAAA | Translation | myoneurin | 1 |
| -2.98 | UUUCGAGACUAAACUACACUGGU | NM_002267 | KPNA3 | 3 | 10.884 | 1 | 23 | 1043 | 1065 | UUUCGAGACUAAACUACACUGGU | ..: :::: ::::::::. ::: | UUUACUGUAAUUUAGUCUUAAAA | Cleavage | karyopherin subunit alpha 3 | 1 |
| -2.98 | UUUCGAGACUAAACUACACUGGU | NM_005546 | ITK | 3 | 14.517 | 1 | 23 | 2089 | 2111 | UUUCGAGACUAAACUACACUGGU | :.: :::.::::.::.: :::: | AUCUCUGUGGUUUGGUUUAGAAA | Cleavage | IL2 inducible T cell kinase | 1 |
| -2.98 | UUUCGAGACUAAACUACACUGGU | NM_001134407 | GRIN2A | 3 | 19.155 | 1 | 23 | 838 | 859 | UUUCGAGACUAAACUACACUGGU | .::: ::::::::: :: ::::. | GCCAUUGUAGUUUA-UCCCGAAG | Translation | glutamate ionotropic receptor NMDA type subunit 2A | 1 |
| -2.98 | UUUCGAGACUAAACUACACUGGU | NM_014960 | ARSG | 2.5 | 13.645 | 1 | 23 | 87 | 109 | UUUCGAGACUAAACUACACUGGU | :. : :::::::::::.:.:. | CACGCUUUAGUUUAGUCUUGGAG | Cleavage | arylsulfatase G | 1 |
| -2.98 | UUUCGAGACUAAACUACACUGGU | NM_001198665 | ARHGEF12 | 2.5 | 16.555 | 1 | 23 | 4499 | 4521 | UUUCGAGACUAAACUACACUGGU | ... :::: :::.::.:.:::: | UUUGCUGUAUUUUGGUUUUGAAA | Cleavage | Rho guanine nucleotide exchange factor 12 | 1 |
| -2.98 | UUUCGAGACUAAACUACACUGGU | NM_152424 | AMER1 | 2.5 | 10.218 | 1 | 23 | 3874 | 3896 | UUUCGAGACUAAACUACACUGGU | :::. ::::::.:.:::: | UGUUUUGUGCUUUAGUUUUGAAA | Cleavage | APC membrane recruitment protein 1 | 1 |
| -2.80 | UUGAAGACUGAAGGGGAA | NM_005095 | ZMYM4 | 2.5 | 10.987 | 1 | 18 | 2177 | 2194 | UUGAAGACUGAAGGGGAA | :::...::::::.::.:: | UUCUUUUUCAGUUUUUAA | Cleavage | zinc finger MYM-type containing 4 | 1 |
| -2.80 | UUGAAGACUGAAGGGGAA | NM_016353 | ZDHHC2 | 3 | 18.152 | 1 | 18 | 1999 | 2016 | UUGAAGACUGAAGGGGAA | :::::.::: ::.:::: | UUCCCUUUCUGUUUUCAU | Translation | zinc finger DHHC-type palmitoyltransferase 2 | 1 |
| -2.80 | UUGAAGACUGAAGGGGAA | NM_001031732 | YTHDC1 | 2.5 | 14.664 | 1 | 18 | 536 | 553 | UUGAAGACUGAAGGGGAA | ::...: ::::::::::: | UUUUUCCUCAGUCUUCAA | Cleavage | YTH N6-methyladenosine RNA binding protein C1 | 1 |
| -2.80 | UUGAAGACUGAAGGGGAA | NM_015902 | UBR5 | 3 | 17.485 | 1 | 18 | 1343 | 1360 | UUGAAGACUGAAGGGGAA | : ..:::: ::::::::: | UCUUCCUUAAGUCUUCAA | Translation | ubiquitin protein ligase E3 component n-recognin 5 | 1 |
| -2.80 | UUGAAGACUGAAGGGGAA | NM_021648 | TSPYL4 | 3 | 21.864 | 1 | 18 | 1919 | 1936 | UUGAAGACUGAAGGGGAA | ::::::::..::.:: :: | UUCCCCUUUGGUUUUGAA | Cleavage | TSPY like 4 | 1 |
| -2.80 | UUGAAGACUGAAGGGGAA | NM_014818 | TRIM66 | 3 | 13.207 | 1 | 18 | 1900 | 1917 | UUGAAGACUGAAGGGGAA | :::::.:::: :.::..: | UUCCCUUUCAAUUUUUGA | Cleavage | tripartite motif containing 66 | 1 |
| -2.80 | UUGAAGACUGAAGGGGAA | NM_015049 | TRAK2 | 3 | 20.389 | 1 | 18 | 603 | 620 | UUGAAGACUGAAGGGGAA | ::.::.::: ::.::::. | UUUCCUUUCUGUUUUCAG | Translation | trafficking kinesin protein 2 | 1 |
| -2.80 | UUGAAGACUGAAGGGGAA | NM_001142640 | TNRC6C | 2.5 | 13.299 | 1 | 18 | 1366 | 1383 | UUGAAGACUGAAGGGGAA | :::.::::::: :::.:: | UUCUCCUUCAGACUUUAA | Cleavage | trinucleotide repeat containing adaptor 6C | 1 |
| -2.80 | UUGAAGACUGAAGGGGAA | NM_001024843 | TNRC6B | 3 | 21.442 | 1 | 18 | 3758 | 3775 | UUGAAGACUGAAGGGGAA | ::.::.::::::.::... | UUUCCUUUCAGUUUUUGG | Cleavage | trinucleotide repeat containing adaptor 6B | 1 |
| -2.80 | UUGAAGACUGAAGGGGAA | NM_001040284 | TENT4B | 2 | 10.074 | 1 | 18 | 2978 | 2995 | UUGAAGACUGAAGGGGAA | :: :::: :::::::::: | UUGCCCUCCAGUCUUCAA | Translation | terminal nucleotidyltransferase 4B | 1 |
| -2.80 | UUGAAGACUGAAGGGGAA | NM_001980 | STX2 | 3 | 14.739 | 1 | 18 | 1730 | 1747 | UUGAAGACUGAAGGGGAA | ::..:.::..:::::.:: | UUUUCUUUUGGUCUUUAA | Cleavage | syntaxin 2 | 1 |
| -2.80 | UUGAAGACUGAAGGGGAA | NM_194356 | STX2 | 3 | 14.739 | 1 | 18 | 1853 | 1870 | UUGAAGACUGAAGGGGAA | ::..:.::..:::::.:: | UUUUCUUUUGGUCUUUAA | Cleavage | syntaxin 2 | 1 |
| -2.80 | UUGAAGACUGAAGGGGAA | NM_005840 | SPRY3 | 3 | 24.369 | 1 | 18 | 196 | 213 | UUGAAGACUGAAGGGGAA | :::.::::::::::.: | CUCCUCUUCAGUCUUUAC | Cleavage | sprouty RTK signaling antagonist 3 | 1 |
| -2.80 | UUGAAGACUGAAGGGGAA | NM_003601 | SMARCA5 | 2.5 | 15.077 | 1 | 18 | 364 | 381 | UUGAAGACUGAAGGGGAA | ::::.::::::::::: | GUCCCUUUCAGUCUUCAC | Cleavage | SWI/SNF related, matrix associated, actin dependent regulator of chromatin, subfamily a, member 5 | 1 |
| -2.80 | UUGAAGACUGAAGGGGAA | NM_003042 | SLC6A1 | 3 | 18.4 | 1 | 18 | 1761 | 1778 | UUGAAGACUGAAGGGGAA | ::.:.:::.. ::::::: | UUUCUCUUUGUUCUUCAA | Cleavage | solute carrier family 6 member 1 | 1 |
| -2.80 | UUGAAGACUGAAGGGGAA | NM_022127 | SLC28A3 | 3 | 21.428 | 1 | 18 | 1579 | 1596 | UUGAAGACUGAAGGGGAA | : :::.: :::::::::. | UGCCCUUGCAGUCUUCAG | Translation | solute carrier family 28 member 3 | 1 |
| -2.80 | UUGAAGACUGAAGGGGAA | NM_012397 | SERPINB13 | 3 | 8.576 | 1 | 18 | 867 | 884 | UUGAAGACUGAAGGGGAA | ::..::: ::::.::.:: | UUUUCCUCCAGUUUUUAA | Translation | serpin family B member 13 | 1 |
| -2.80 | UUGAAGACUGAAGGGGAA | NM_001127706 | SERPINA1 | 3 | 24.128 | 1 | 18 | 1019 | 1036 | UUGAAGACUGAAGGGGAA | : .:::: :::::::::. | UGUCCCUGCAGUCUUCAG | Translation | serpin family A member 1 | 1 |
| -2.80 | UUGAAGACUGAAGGGGAA | NM_033505 | SELENOI | 3 | 16.107 | 1 | 18 | 3392 | 3409 | UUGAAGACUGAAGGGGAA | ::::.::::. :::::: | UUCCUCUUCGUUCUUCAC | Cleavage | selenoprotein I | 1 |
| -2.80 | UUGAAGACUGAAGGGGAA | NM_007190 | SEC23IP | 3 | 16.367 | 1 | 18 | 1439 | 1456 | UUGAAGACUGAAGGGGAA | :::::.::::: ::::: | UUCCCUUUCAGCCUUCAU | Cleavage | SEC23 interacting protein | 1 |
| -2.80 | UUGAAGACUGAAGGGGAA | NM_001129993 | SANBR | 2.5 | 13.109 | 1 | 18 | 622 | 639 | UUGAAGACUGAAGGGGAA | ::..:.::::::.::.:: | UUUUCUUUCAGUUUUUAA | Cleavage | SANT and BTB domain regulator of CSR | 1 |
| -2.80 | UUGAAGACUGAAGGGGAA | NM_016026 | RDH11 | 3 | 11.848 | 1 | 18 | 67 | 86 | UUGAAGAC--UGAAGGGGAA | :::.:::::: ::.::::: | UUCUCCUUCAAGGUUUUCAA | Cleavage | retinol dehydrogenase 11 | 1 |
| -2.80 | UUGAAGACUGAAGGGGAA | NM_002895 | RBL1 | 3 | 17.547 | 1 | 18 | 560 | 577 | UUGAAGACUGAAGGGGAA | : :.:::: :::.::::: | UGCUCCUUGAGUUUUCAA | Translation | RB transcriptional corepressor like 1 | 1 |
| -2.80 | UUGAAGACUGAAGGGGAA | NM_001276277 | PPIP5K2 | 3 | 10.937 | 1 | 18 | 1614 | 1631 | UUGAAGACUGAAGGGGAA | :: :..::.:::.::::: | UUACUUUUUAGUUUUCAA | Cleavage | diphosphoinositol pentakisphosphate kinase 2 | 1 |
| -2.80 | UUGAAGACUGAAGGGGAA | NM_014510 | PCLO | 2.5 | 5.94 | 1 | 18 | 62 | 79 | UUGAAGACUGAAGGGGAA | ::. :.::::::.::::: | UUUGCUUUCAGUUUUCAA | Cleavage | piccolo presynaptic cytomatrix protein | 1 |
| -2.80 | UUGAAGACUGAAGGGGAA | NM_032632 | PAPOLA | 3 | 14.391 | 1 | 18 | 452 | 469 | UUGAAGACUGAAGGGGAA | ::. :: :.::::::::: | UUUACCAUUAGUCUUCAA | Cleavage | poly(A) polymerase alpha | 1 |
| -2.80 | UUGAAGACUGAAGGGGAA | NM_013358 | PADI1 | 3 | 22.817 | 1 | 18 | 993 | 1010 | UUGAAGACUGAAGGGGAA | :::::::::: ::: ::. | UUCCCCUUCAUUCUGCAG | Cleavage | peptidyl arginine deiminase 1 | 1 |
| -2.80 | UUGAAGACUGAAGGGGAA | NM_018002 | OXR1 | 2 | 14.816 | 1 | 18 | 1616 | 1633 | UUGAAGACUGAAGGGGAA | :::::::::.:::::: : | UUCCCCUUCGGUCUUCCA | Cleavage | oxidation resistance 1 | 1 |
| -2.80 | UUGAAGACUGAAGGGGAA | NM_133331 | NSD2 | 3 | 15.706 | 1 | 18 | 1281 | 1298 | UUGAAGACUGAAGGGGAA | ::.: :::.:::.::.:: | UUUCACUUUAGUUUUUAA | Cleavage | nuclear receptor binding SET domain protein 2 | 1 |
| -2.80 | UUGAAGACUGAAGGGGAA | NM_001042424 | NSD2 | 3 | 15.706 | 1 | 18 | 1281 | 1298 | UUGAAGACUGAAGGGGAA | ::.: :::.:::.::.:: | UUUCACUUUAGUUUUUAA | Cleavage | nuclear receptor binding SET domain protein 2 | 1 |
| -2.80 | UUGAAGACUGAAGGGGAA | NM_001040110 | NRF1 | 3 | 17.58 | 1 | 18 | 1843 | 1860 | UUGAAGACUGAAGGGGAA | ::::: ::: :::::..: | UUCCCAUUCUGUCUUUGA | Translation | nuclear respiratory factor 1 | 1 |
| -2.80 | UUGAAGACUGAAGGGGAA | NM_153604 | MYOCD | 2.5 | 18.394 | 1 | 18 | 1306 | 1323 | UUGAAGACUGAAGGGGAA | :::.: ::.:::.::::: | UUCUCGUUUAGUUUUCAA | Cleavage | myocardin | 1 |
| -2.80 | UUGAAGACUGAAGGGGAA | NM_199181 | MTCL2 | 2 | 9.344 | 1 | 18 | 147 | 164 | UUGAAGACUGAAGGGGAA | :::: :::: :::::::: | UUCCACUUCUGUCUUCAA | Translation | microtubule crosslinking factor 2 | 1 |
| -2.80 | UUGAAGACUGAAGGGGAA | NM_031457 | MS4A8 | 3 | 13.007 | 1 | 18 | 126 | 143 | UUGAAGACUGAAGGGGAA | :: :::::: ::::::: | CUCACCUUCAUUCUUCAA | Cleavage | membrane spanning 4-domains A8 | 1 |
| -2.80 | UUGAAGACUGAAGGGGAA | NM_178336 | MRPL52 | 2.5 | 16.77 | 1 | 18 | 91 | 108 | UUGAAGACUGAAGGGGAA | :::::: :: :::::::. | UUCCCCAUCUGUCUUCAG | Translation | mitochondrial ribosomal protein L52 | 1 |
| -2.80 | UUGAAGACUGAAGGGGAA | NM_181307 | MRPL52 | 2.5 | 16.77 | 1 | 18 | 134 | 151 | UUGAAGACUGAAGGGGAA | :::::: :: :::::::. | UUCCCCAUCUGUCUUCAG | Translation | mitochondrial ribosomal protein L52 | 1 |
| -2.80 | UUGAAGACUGAAGGGGAA | NM_014381 | MLH3 | 3 | 9.197 | 1 | 18 | 459 | 476 | UUGAAGACUGAAGGGGAA | ::.::::::::::::: | UUUCCCUUCAGUCUUCUU | Cleavage | mutL homolog 3 | 1 |
| -2.80 | UUGAAGACUGAAGGGGAA | NM_153450 | MED19 | 3 | 20.17 | 1 | 18 | 146 | 163 | UUGAAGACUGAAGGGGAA | ::..::: .::::::::. | UUUUCCUGUAGUCUUCAG | Translation | mediator complex subunit 19 | 1 |
| -2.80 | UUGAAGACUGAAGGGGAA | NM_005121 | MED13 | 3 | 9.066 | 1 | 18 | 2988 | 3005 | UUGAAGACUGAAGGGGAA | :::.::::. ::::::: | UUCUCCUUUCGUCUUCAU | Translation | mediator complex subunit 13 | 1 |
| -2.80 | UUGAAGACUGAAGGGGAA | NM_030885 | MAP4 | 2.5 | 14.145 | 1 | 18 | 374 | 391 | UUGAAGACUGAAGGGGAA | ::..::::..::.::::: | UUUUCCUUUGGUUUUCAA | Cleavage | microtubule associated protein 4 | 1 |
| -2.80 | UUGAAGACUGAAGGGGAA | NM_016200 | LSM8 | 3 | 16.032 | 1 | 18 | 10988 | 11005 | UUGAAGACUGAAGGGGAA | ::::. ::::::::.:: | CUCCCUAUCAGUCUUUAA | Cleavage | LSM8 homolog, U6 small nuclear RNA associated | 1 |
| -2.80 | UUGAAGACUGAAGGGGAA | NM_198461 | LONRF2 | 3 | 11.092 | 1 | 18 | 1423 | 1440 | UUGAAGACUGAAGGGGAA | ::...:::.:::.::::. | UUUUUCUUUAGUUUUCAG | Cleavage | LON peptidase N-terminal domain and ring finger 2 | 2 |
| -2.80 | UUGAAGACUGAAGGGGAA | NM_198461 | LONRF2 | 3 | 11.217 | 1 | 18 | 1487 | 1504 | UUGAAGACUGAAGGGGAA | ::..:.::.:::.::::. | UUUUCUUUUAGUUUUCAG | Cleavage | LON peptidase N-terminal domain and ring finger 2 | 2 |
| -2.80 | UUGAAGACUGAAGGGGAA | NM_014240 | LIMD1 | 3 | 4.79 | 1 | 18 | 1141 | 1158 | UUGAAGACUGAAGGGGAA | ::: :.:::::::::: : | UUCACUUUCAGUCUUCCA | Cleavage | LIM domain containing 1 | 1 |
| -2.80 | UUGAAGACUGAAGGGGAA | NM_006060 | IKZF1 | 2 | 11.957 | 1 | 18 | 2039 | 2056 | UUGAAGACUGAAGGGGAA | :::::::::.::::: :: | UUCCCCUUCGGUCUUAAA | Cleavage | IKAROS family zinc finger 1 | 1 |
| -2.80 | UUGAAGACUGAAGGGGAA | NM_000195 | HPS1 | 2.5 | 14.267 | 1 | 18 | 1260 | 1277 | UUGAAGACUGAAGGGGAA | ::.:::::.:::.::.:. | UUUCCCUUUAGUUUUUAG | Cleavage | HPS1 biogenesis of lysosomal organelles complex 3 subunit 1 | 1 |
| -2.80 | UUGAAGACUGAAGGGGAA | NM_005708 | GPC6 | 1 | 11.116 | 1 | 18 | 787 | 803 | UUGAAGACUGAAGGGGAA | :::::::::: ::::::: | UUCCCCUUCA-UCUUCAA | Cleavage | glypican 6 | 1 |
| -2.80 | UUGAAGACUGAAGGGGAA | NM_001248 | ENTPD3 | 3 | 9.659 | 1 | 18 | 426 | 443 | UUGAAGACUGAAGGGGAA | ::.::: :..:::::.:: | UUUCCCAUUGGUCUUUAA | Cleavage | ectonucleoside triphosphate diphosphohydrolase 3 | 1 |
| -2.80 | UUGAAGACUGAAGGGGAA | NM_005801 | EIF1 | 3 | 11.84 | 1 | 18 | 749 | 766 | UUGAAGACUGAAGGGGAA | ::::::::::::.:: : | UUCCCCUUCAGUUUUAAU | Cleavage | eukaryotic translation initiation factor 1 | 1 |
| -2.80 | UUGAAGACUGAAGGGGAA | NM_004952 | EFNA3 | 2 | 5.705 | 1 | 18 | 696 | 712 | UUGAAGACUGAAGGGGAA | ::::::::::: :::::. | UUCCCCUUCAG-CUUCAG | Cleavage | ephrin A3 | 1 |
| -2.80 | UUGAAGACUGAAGGGGAA | NM_001142444 | EDC3 | 3 | 19.186 | 1 | 18 | 1493 | 1510 | UUGAAGACUGAAGGGGAA | :.:::::::::::: :: | CUUCCCUUCAGUCUUAAA | Cleavage | enhancer of mRNA decapping 3 | 1 |
| -2.80 | UUGAAGACUGAAGGGGAA | NM_001171184 | DRP2 | 3 | 19.628 | 1 | 18 | 217 | 234 | UUGAAGACUGAAGGGGAA | :.:::::.: ::::::: | GUUCCCUUUAAUCUUCAA | Cleavage | dystrophin related protein 2 | 1 |
| -2.80 | UUGAAGACUGAAGGGGAA | NM_007068 | DMC1 | 3 | 18.679 | 1 | 18 | 348 | 365 | UUGAAGACUGAAGGGGAA | ::::.:::: :.::::: | CUCCCUUUCACUUUUCAA | Cleavage | DNA meiotic recombinase 1 | 1 |
| -2.80 | UUGAAGACUGAAGGGGAA | NM_080796 | DIDO1 | 3 | 10.577 | 1 | 18 | 771 | 788 | UUGAAGACUGAAGGGGAA | ::.: :::::::::.:: | UUUCAAUUCAGUCUUUAA | Cleavage | death inducer-obliterator 1 | 1 |
| -2.80 | UUGAAGACUGAAGGGGAA | NM_001160147 | DDHD1 | 1.5 | 21.436 | 1 | 18 | 4172 | 4189 | UUGAAGACUGAAGGGGAA | :::.:.:::::::::::. | UUCUCUUUCAGUCUUCAG | Cleavage | DDHD domain containing 1 | 1 |
| -2.80 | UUGAAGACUGAAGGGGAA | NM_000777 | CYP3A5 | 2.5 | 11.624 | 1 | 18 | 15 | 32 | UUGAAGACUGAAGGGGAA | :::. :::..:::::::: | UUCUACUUUGGUCUUCAA | Cleavage | cytochrome P450 family 3 subfamily A member 5 | 1 |
| -2.80 | UUGAAGACUGAAGGGGAA | NM_001114173 | CTSC | 3 | 15.669 | 1 | 18 | 5425 | 5442 | UUGAAGACUGAAGGGGAA | ::::.:: ..:::::.:: | UUCCUCUCUGGUCUUUAA | Translation | cathepsin C | 1 |
| -2.80 | UUGAAGACUGAAGGGGAA | NM_148170 | CTSC | 3 | 15.669 | 1 | 18 | 5481 | 5498 | UUGAAGACUGAAGGGGAA | ::::.:: ..:::::.:: | UUCCUCUCUGGUCUUUAA | Translation | cathepsin C | 1 |
| -2.80 | UUGAAGACUGAAGGGGAA | NM_001199302 | CNOT2 | 2.5 | 13.22 | 1 | 18 | 500 | 517 | UUGAAGACUGAAGGGGAA | ::::::::: ::.::..: | UUCCCCUUCUGUUUUUGA | Translation | CCR4-NOT transcription complex subunit 2 | 1 |
| -2.80 | UUGAAGACUGAAGGGGAA | NM_000616 | CD4 | 3 | 2.367 | 1 | 18 | 1331 | 1348 | UUGAAGACUGAAGGGGAA | :::.::: : :::::.:: | UUCUCCUCCUGUCUUUAA | Translation | CD4 molecule | 1 |
| -2.80 | UUGAAGACUGAAGGGGAA | NM_024098 | CCDC86 | 3 | 12.395 | 1 | 18 | 483 | 500 | UUGAAGACUGAAGGGGAA | ::::.:: :: :.::::: | UUCCUCUCCACUUUUCAA | Translation | coiled-coil domain containing 86 | 1 |
| -2.80 | UUGAAGACUGAAGGGGAA | NM_020139 | BDH2 | 2.5 | 3.566 | 1 | 18 | 942 | 959 | UUGAAGACUGAAGGGGAA | :::: ::::: :.::::: | UUCCACUUCAUUUUUCAA | Cleavage | 3-hydroxybutyrate dehydrogenase 2 | 1 |
| -2.80 | UUGAAGACUGAAGGGGAA | NM_006628 | ARPP19 | 3 | 7.671 | 1 | 18 | 1171 | 1188 | UUGAAGACUGAAGGGGAA | ::::::::.. ::::..: | UUCCCCUUUGUUCUUUGA | Cleavage | cAMP regulated phosphoprotein 19 | 1 |
| -2.80 | UUGAAGACUGAAGGGGAA | NM_001008239 | ARK2N | 3 | 9.672 | 1 | 18 | 3710 | 3727 | UUGAAGACUGAAGGGGAA | ::.:::::.::: ::.:: | UUUCCCUUUAGUAUUUAA | Cleavage | arkadia (RNF111) N-terminal like PKA signaling regulator 2N | 1 |
| -2.80 | UUGAAGACUGAAGGGGAA | NM_058172 | ANTXR2 | 2.5 | 14.033 | 1 | 18 | 662 | 679 | UUGAAGACUGAAGGGGAA | :: :::::.:::.:::.: | UUGCCCUUUAGUUUUCGA | Cleavage | ANTXR cell adhesion molecule 2 | 1 |
| -2.80 | UUGAAGACUGAAGGGGAA | NM_004274 | AKAP6 | 3 | 6.015 | 1 | 18 | 332 | 349 | UUGAAGACUGAAGGGGAA | :::::.: :: ::::.:: | UUCCCUUCCACUCUUUAA | Translation | A-kinase anchoring protein 6 | 1 |
| -2.79 | CUAGACGUCAGCGACUCCUUGA | NM_017715 | ZNF3 | 2.5 | 20.222 | 1 | 22 | 193 | 214 | CUAGACGUCAGCGACUCCUUGA | ::: :::::::.::.:.: | GUGUGGACUCGCUGAUGUUUGG | Cleavage | zinc finger protein 3 | 1 |
| -2.79 | CUAGACGUCAGCGACUCCUUGA | NM_014830 | ZBTB39 | 3 | 14.167 | 1 | 22 | 3724 | 3745 | CUAGACGUCAGCGACUCCUUGA | .: ::::.:.::::::::: | GAGAAGAGUUGUUGACGUCUAA | Cleavage | zinc finger and BTB domain containing 39 | 1 |
| -2.79 | CUAGACGUCAGCGACUCCUUGA | NM_006555 | YKT6 | 3 | 24.432 | 1 | 22 | 403 | 423 | CUAGACGUCAGCGACUCCUUGA | ...:::::: ::::.: :::: | GUGGGGAGUCCCUGAUG-CUAG | Cleavage | YKT6 v-SNARE homolog | 1 |
| -2.79 | CUAGACGUCAGCGACUCCUUGA | NM_001201484 | PIGO | 3 | 14.934 | 1 | 22 | 119 | 140 | CUAGACGUCAGCGACUCCUUGA | : :::.: ::::::: ::::: | GCCAGGGGCCGCUGACAUCUAG | Cleavage | phosphatidylinositol glycan anchor biosynthesis class O | 1 |
| -2.79 | CUAGACGUCAGCGACUCCUUGA | NM_153050 | MTMR3 | 3 | 23.643 | 1 | 22 | 4875 | 4895 | CUAGACGUCAGCGACUCCUUGA | ::: ::: :: ::::.::.::: | UCAUGGAUUC-CUGAUGUUUAG | Cleavage | myotubularin related protein 3 | 1 |
| -2.79 | CUAGACGUCAGCGACUCCUUGA | NM_001126049 | KLLN | 2 | 14.735 | 1 | 22 | 1095 | 1116 | CUAGACGUCAGCGACUCCUUGA | .:::::::. ::::.:::::: | AUAAGGAGUUCCUGAUGUCUAG | Cleavage | killin, p53 regulated DNA replication inhibitor | 1 |
| -2.79 | CUAGACGUCAGCGACUCCUUGA | NM_007210 | GALNT6 | 2.5 | 17.713 | 1 | 22 | 45 | 65 | CUAGACGUCAGCGACUCCUUGA | :::: :.:::::.::::.: | AACAGGA-UUGCUGAUGUCUGG | Cleavage | polypeptide N-acetylgalactosaminyltransferase 6 | 1 |
| -2.79 | CUAGACGUCAGCGACUCCUUGA | NM_005808 | CTDSPL | 3 | 20.76 | 1 | 22 | 3421 | 3442 | CUAGACGUCAGCGACUCCUUGA | :::: .::::..::::.: | UAGUGGAGAUGCUGGUGUCUGG | Cleavage | CTD small phosphatase like | 1 |
| -2.76 | GGCUCGCAGGACGAGGGAUU | NM_175039 | ST6GALNAC4 | 3 | 20.801 | 1 | 20 | 1 | 20 | GGCUCGCAGGACGAGGGAUU | ::: ::::::::: ::.: | CUUCCGUCGUCCUGCCAGUC | Cleavage | ST6 N-acetylgalactosaminide alpha-2,6-sialyltransferase 4 | 1 |
| -2.76 | GGCUCGCAGGACGAGGGAUU | NM_018079 | SRBD1 | 3 | 7.657 | 1 | 20 | 494 | 513 | GGCUCGCAGGACGAGGGAUU | :::::.::: ::: :::: | UUUCCCUUGUCAUGCAAGCC | Translation | S1 RNA binding domain 1 | 1 |
| -2.76 | GGCUCGCAGGACGAGGGAUU | NM_014654 | SDC3 | 3 | 15.666 | 1 | 20 | 3388 | 3408 | GGCUCGCAGGA-CGAGGGAUU | .:::::: :::::.:::.: | UGUCCCUCCCUCCUGUGAGUC | Translation | syndecan 3 | 1 |
| -2.76 | GGCUCGCAGGACGAGGGAUU | NM_003681 | PDXK | 2.5 | 21.031 | 1 | 20 | 1981 | 2001 | GGCUCGCAGGAC-GAGGGAUU | .::.::: ::::::.::::. | UGUCUCUCUGUCCUGUGAGCU | Cleavage | pyridoxal kinase | 1 |
| -2.76 | GGCUCGCAGGACGAGGGAUU | NM_001040134 | PALM | 3 | 21.471 | 1 | 20 | 1389 | 1408 | GGCUCGCAGGACGAGGGAUU | :.::::::: : ::::::: | AGUCCCUCGAGCCGCGAGCC | Translation | paralemmin | 1 |
| -2.76 | GGCUCGCAGGACGAGGGAUU | NM_006863 | LILRA1 | 3 | 15.087 | 1 | 20 | 739 | 758 | GGCUCGCAGGACGAGGGAUU | :::.:.: ::::.::::. | UCUCCUUUGACCUGUGAGCU | Translation | leukocyte immunoglobulin like receptor A1 | 1 |
| -2.76 | GGCUCGCAGGACGAGGGAUU | NM_000140 | FECH | 3 | 18.296 | 1 | 20 | 3545 | 3565 | GGCUCGCAGG-ACGAGGGAUU | :.:::::. : ::::.::::: | AGUCCCUUCUGCCUGUGAGCC | Translation | ferrochelatase | 1 |
| -2.76 | GGCUCGCAGGACGAGGGAUU | NM_001013647 | FAM227A | 3 | 14.709 | 1 | 20 | 3221 | 3240 | GGCUCGCAGGACGAGGGAUU | .:::::: ::.:: ::::: | CGUCCCUCAUCUUGGGAGCC | Cleavage | family with sequence similarity 227 member A | 1 |
| -2.76 | GGCUCGCAGGACGAGGGAUU | NM_176819 | DIPK2B | 3 | 22.041 | 1 | 20 | 2308 | 2326 | GGCUCGCAGGACGAGGGAUU | :.:::::: :::::: .::: | AGUCCCUC-UCCUGCUGGCC | Cleavage | divergent protein kinase domain 2B | 1 |
| -2.76 | GGCUCGCAGGACGAGGGAUU | NM_001243565 | DHDDS | 3 | 23.779 | 1 | 20 | 2076 | 2095 | GGCUCGCAGGACGAGGGAUU | .:::.:: ::::.::::: | UGUCCUUCCACCUGUGAGCC | Translation | dehydrodolichyl diphosphate synthase subunit | 1 |
| -2.76 | GGCUCGCAGGACGAGGGAUU | NM_016144 | COMMD10 | 3 | 14.785 | 1 | 20 | 189 | 208 | GGCUCGCAGGACGAGGGAUU | ::..: ::.:::.::::: | UUUCUUUAGUUCUGUGAGCC | Cleavage | COMM domain containing 10 | 1 |
| -2.76 | GGCUCGCAGGACGAGGGAUU | NM_004308 | ARHGAP1 | 2.5 | 20.762 | 1 | 20 | 1453 | 1471 | GGCUCGCAGGACGAGGGAUU | .::::::: :::::: :::: | GAUCCCUCUUCCUGC-AGCC | Cleavage | Rho GTPase activating protein 1 | 1 |
| -2.76 | GGCUCGCAGGACGAGGGAUU | NM_001150 | ANPEP | 3 | 15.862 | 1 | 20 | 136 | 155 | GGCUCGCAGGACGAGGGAUU | ::.::: ::::.::::: | GUUCUCUCUGCCUGUGAGCC | Translation | alanyl aminopeptidase, membrane | 1 |
| -2.75 | UUUUUUUUUUGUUUUGGC | NM_001040653 | ZXDC | 1.5 | 11.391 | 1 | 18 | 7816 | 7833 | UUUUUUUUUUGUUUUGGC | ::::::: ::::.::::: | GCCAAAAAAAAAGAAAAA | Translation | ZXD family zinc finger C | 1 |
| -2.75 | UUUUUUUUUUGUUUUGGC | NM_001013659 | ZNF793 | 1.5 | 15.118 | 1 | 18 | 2384 | 2401 | UUUUUUUUUUGUUUUGGC | :.::::: :::::::::: | GUCAAAAAAAAAAAAAAA | Translation | zinc finger protein 793 | 1 |
| -2.75 | UUUUUUUUUUGUUUUGGC | NM_024733 | ZNF665 | 2 | 12.294 | 1 | 18 | 296 | 313 | UUUUUUUUUUGUUUUGGC | :::::: :::::::::: | UCCAAAAAAAAAAAAAAA | Translation | zinc finger protein 665 | 1 |
| -2.75 | UUUUUUUUUUGUUUUGGC | NM_014897 | ZNF652 | 2.5 | 6.888 | 1 | 18 | 9174 | 9191 | UUUUUUUUUUGUUUUGGC | .::::..:::::::::: | UUCAAAGUAAAAAAAAAA | Cleavage | zinc finger protein 652 | 1 |
| -2.75 | UUUUUUUUUUGUUUUGGC | NM_001134442 | ZNF502 | 1 | 6.139 | 1 | 18 | 523 | 540 | UUUUUUUUUUGUUUUGGC | ::::::: :::::::::: | GCCAAAAAAAAAAAAAAA | Translation | zinc finger protein 502 | 1 |
| -2.75 | UUUUUUUUUUGUUUUGGC | NM_005649 | ZNF354A | 2 | 12.302 | 1 | 18 | 435 | 452 | UUUUUUUUUUGUUUUGGC | ::::::.::::.::::: | ACCAAAAUAAAAGAAAAA | Cleavage | zinc finger protein 354A | 1 |
| -2.75 | UUUUUUUUUUGUUUUGGC | NM_021964 | ZNF148 | 2.5 | 13.41 | 1 | 18 | 952 | 969 | UUUUUUUUUUGUUUUGGC | .::::..:::::::::: | CUCAAAGUAAAAAAAAAA | Cleavage | zinc finger protein 148 | 1 |
| -2.75 | UUUUUUUUUUGUUUUGGC | NM_020338 | ZMIZ1 | 2 | 7.823 | 1 | 18 | 3103 | 3120 | UUUUUUUUUUGUUUUGGC | :::::::::::::::: | AACAAAACAAAAAAAAAA | Cleavage | zinc finger MIZ-type containing 1 | 1 |
| -2.75 | UUUUUUUUUUGUUUUGGC | NM_022470 | ZMAT3 | 2 | 14.702 | 1 | 18 | 1836 | 1853 | UUUUUUUUUUGUUUUGGC | :::::: :::::::::: | UCCAAAAAAAAAAAAAAA | Translation | zinc finger matrin-type 3 | 1 |
| -2.75 | UUUUUUUUUUGUUUUGGC | NM_020917 | ZFP14 | 1.5 | 9.371 | 1 | 18 | 3962 | 3979 | UUUUUUUUUUGUUUUGGC | :.::::: :::::::::: | GUCAAAAAAAAAAAAAAA | Translation | ZFP14 zinc finger protein | 1 |
| -2.75 | UUUUUUUUUUGUUUUGGC | NM_024721 | ZFHX4 | 1.5 | 4.973 | 1 | 18 | 2223 | 2240 | UUUUUUUUUUGUUUUGGC | ::::::: ::.::::::: | GCCAAAAAAAGAAAAAAA | Translation | zinc finger homeobox 4 | 1 |
| -2.75 | UUUUUUUUUUGUUUUGGC | NM_207660 | ZC3H14 | 1.5 | 7.795 | 1 | 18 | 1226 | 1243 | UUUUUUUUUUGUUUUGGC | ::.:::: :::::::::: | GCUAAAAAAAAAAAAAAA | Translation | zinc finger CCCH-type containing 14 | 1 |
| -2.75 | UUUUUUUUUUGUUUUGGC | NM_001002838 | WNK3 | 2 | 15.186 | 1 | 18 | 1900 | 1917 | UUUUUUUUUUGUUUUGGC | :::::: :::::::::: | UCCAAAAAAAAAAAAAAA | Translation | WNK lysine deficient protein kinase 3 | 1 |
| -2.75 | UUUUUUUUUUGUUUUGGC | NM_001201404 | WASF2 | 2 | 13.722 | 1 | 18 | 2691 | 2708 | UUUUUUUUUUGUUUUGGC | :::::: :::::::::: | ACCAAAAAAAAAAAAAAA | Translation | WASP family member 2 | 1 |
| -2.75 | UUUUUUUUUUGUUUUGGC | NM_006990 | WASF2 | 2 | 13.722 | 1 | 18 | 2555 | 2572 | UUUUUUUUUUGUUUUGGC | :::::: :::::::::: | ACCAAAAAAAAAAAAAAA | Translation | WASP family member 2 | 1 |
| -2.75 | UUUUUUUUUUGUUUUGGC | NM_194434 | VAPA | 2 | 7.22 | 1 | 18 | 5508 | 5525 | UUUUUUUUUUGUUUUGGC | :::::: :::::::::: | UCCAAAAAAAAAAAAAAA | Translation | VAMP associated protein A | 1 |
| -2.75 | UUUUUUUUUUGUUUUGGC | NM_001267576 | USP43 | 1.5 | 15.745 | 1 | 18 | 465 | 482 | UUUUUUUUUUGUUUUGGC | ::::::: .::::::::: | GCCAAAAAGAAAAAAAAA | Translation | ubiquitin specific peptidase 43 | 1 |
| -2.75 | UUUUUUUUUUGUUUUGGC | NM_020935 | USP37 | 2.5 | 19.811 | 1 | 18 | 4216 | 4233 | UUUUUUUUUUGUUUUGGC | ::::.::::::.::.:: | GCCAGAACAAAGAAGAAC | Cleavage | ubiquitin specific peptidase 37 | 1 |
| -2.75 | UUUUUUUUUUGUUUUGGC | NM_001080421 | UNC13A | 2 | 5.581 | 1 | 18 | 2239 | 2256 | UUUUUUUUUUGUUUUGGC | :::::: :::::::::: | ACCAAAAAAAAAAAAAAA | Translation | unc-13 homolog A | 1 |
| -2.75 | UUUUUUUUUUGUUUUGGC | NM_001074 | UGT2B7 | 2.5 | 12.399 | 1 | 18 | 93 | 110 | UUUUUUUUUUGUUUUGGC | :..:.:::::::::::: | CCUGAGACAAAAAAAAAA | Cleavage | UDP glucuronosyltransferase family 2 member B7 | 1 |
| -2.75 | UUUUUUUUUUGUUUUGGC | NM_153235 | TXLNB | 2.5 | 13.111 | 1 | 18 | 846 | 863 | UUUUUUUUUUGUUUUGGC | ::::::::.::::.:.: | CCCAAAACAGAAAAGAGA | Cleavage | taxilin beta | 1 |
| -2.75 | UUUUUUUUUUGUUUUGGC | NM_001193421 | TSHZ2 | 1 | 9.996 | 1 | 18 | 135 | 152 | UUUUUUUUUUGUUUUGGC | ::::::: :::::::::: | GCCAAAAAAAAAAAAAAA | Translation | teashirt zinc finger homeobox 2 | 1 |
| -2.75 | UUUUUUUUUUGUUUUGGC | NM_213590 | TRIM13 | 2 | 4.999 | 1 | 18 | 1147 | 1164 | UUUUUUUUUUGUUUUGGC | :::::: :::::::::: | ACCAAAAAAAAAAAAAAA | Translation | tripartite motif containing 13 | 1 |
| -2.75 | UUUUUUUUUUGUUUUGGC | NM_014965 | TRAK1 | 1.5 | 12.271 | 1 | 18 | 144 | 161 | UUUUUUUUUUGUUUUGGC | ::.:::: :::::::::: | GCUAAAAAAAAAAAAAAA | Translation | trafficking kinesin protein 1 | 1 |
| -2.75 | UUUUUUUUUUGUUUUGGC | NM_014494 | TNRC6A | 1.5 | 9.249 | 1 | 18 | 1066 | 1083 | UUUUUUUUUUGUUUUGGC | ::::::: ::::.::::: | GCCAAAAAAAAAGAAAAA | Translation | trinucleotide repeat containing adaptor 6A | 2 |
| -2.75 | UUUUUUUUUUGUUUUGGC | NM_014494 | TNRC6A | 3 | 9.906 | 1 | 18 | 960 | 977 | UUUUUUUUUUGUUUUGGC | ..:::: :::::::::: | UUUAAAAAAAAAAAAAAA | Translation | trinucleotide repeat containing adaptor 6A | 2 |
| -2.75 | UUUUUUUUUUGUUUUGGC | NM_024873 | TNIP3 | 2 | 3.317 | 1 | 18 | 1005 | 1022 | UUUUUUUUUUGUUUUGGC | :::::: :::::::::: | ACCAAAAAAAAAAAAAAA | Translation | TNFAIP3 interacting protein 3 | 1 |
| -2.75 | UUUUUUUUUUGUUUUGGC | NM_001244764 | TNIP3 | 2 | 3.317 | 1 | 18 | 1022 | 1039 | UUUUUUUUUUGUUUUGGC | :::::: :::::::::: | ACCAAAAAAAAAAAAAAA | Translation | TNFAIP3 interacting protein 3 | 1 |
| -2.75 | UUUUUUUUUUGUUUUGGC | NM_001145645 | TNFSF13B | 2 | 6.2 | 1 | 18 | 76 | 93 | UUUUUUUUUUGUUUUGGC | :::::: :::::::::: | ACCAAAAAAAAAAAAAAA | Translation | TNF superfamily member 13b | 1 |
| -2.75 | UUUUUUUUUUGUUUUGGC | NM_001162900 | TMEM217 | 2 | 15.186 | 1 | 18 | 234 | 251 | UUUUUUUUUUGUUUUGGC | :::::: :::::::::: | CCCAAAAAAAAAAAAAAA | Translation | transmembrane protein 217 | 1 |
| -2.75 | UUUUUUUUUUGUUUUGGC | NM_173633 | TMEM145 | 2.5 | 6.688 | 1 | 18 | 214 | 231 | UUUUUUUUUUGUUUUGGC | :::::::.::::::..: | GCCAAAAUAAAAAAGGAU | Cleavage | transmembrane protein 145 | 1 |
| -2.75 | UUUUUUUUUUGUUUUGGC | NM_001080825 | TMEM120B | 2 | 10.226 | 1 | 18 | 2726 | 2743 | UUUUUUUUUUGUUUUGGC | :::::: :::::::::: | CCCAAAAAAAAAAAAAAA | Translation | transmembrane protein 120B | 1 |
| -2.75 | UUUUUUUUUUGUUUUGGC | NM_020698 | TMCC3 | 1 | 12.974 | 1 | 18 | 3156 | 3173 | UUUUUUUUUUGUUUUGGC | ::::::: :::::::::: | GCCAAAAAAAAAAAAAAA | Translation | transmembrane and coiled-coil domain family 3 | 1 |
| -2.75 | UUUUUUUUUUGUUUUGGC | NM_020375 | TIGAR | 2 | 9.934 | 1 | 18 | 5594 | 5611 | UUUUUUUUUUGUUUUGGC | :::::: :::::::::: | UCCAAAAAAAAAAAAAAA | Translation | TP53 induced glycolysis regulatory phosphatase | 1 |
| -2.75 | UUUUUUUUUUGUUUUGGC | NM_024817 | THSD4 | 2 | 3.758 | 1 | 18 | 3160 | 3177 | UUUUUUUUUUGUUUUGGC | :::::: :::::::::: | ACCAAAAGAAAAAAAAAA | Translation | thrombospondin type 1 domain containing 4 | 1 |
| -2.75 | UUUUUUUUUUGUUUUGGC | NM_001040284 | TENT4B | 2 | 2.896 | 1 | 18 | 181 | 198 | UUUUUUUUUUGUUUUGGC | :::::::::::::::: | AACAAAACAAAAAAAAAA | Cleavage | terminal nucleotidyltransferase 4B | 1 |
| -2.75 | UUUUUUUUUUGUUUUGGC | NM_001083962 | TCF4 | 2.5 | 10.508 | 1 | 18 | 4801 | 4818 | UUUUUUUUUUGUUUUGGC | :::::::::::.:::: | UGCAAAACAAAAAGAAAA | Cleavage | transcription factor 4 | 2 |
| -2.75 | UUUUUUUUUUGUUUUGGC | NM_001083962 | TCF4 | 2.5 | 10.939 | 1 | 18 | 4336 | 4353 | UUUUUUUUUUGUUUUGGC | .::::: :::::::::: | UUCAAAAAAAAAAAAAAA | Translation | transcription factor 4 | 2 |
| -2.75 | UUUUUUUUUUGUUUUGGC | NM_001128933 | SYNPO2 | 2 | 11.086 | 1 | 18 | 3760 | 3777 | UUUUUUUUUUGUUUUGGC | :::::: :::::::::: | UCCAAAAAAAAAAAAAAA | Translation | synaptopodin 2 | 1 |
| -2.75 | UUUUUUUUUUGUUUUGGC | NM_003162 | STRN | 2 | 5.374 | 1 | 18 | 8897 | 8914 | UUUUUUUUUUGUUUUGGC | :::::: :::::::::: | ACCAAAAAAAAAAAAAAA | Translation | striatin | 1 |
| -2.75 | UUUUUUUUUUGUUUUGGC | NM_001024858 | SPTB | 2 | 11.086 | 1 | 18 | 349 | 366 | UUUUUUUUUUGUUUUGGC | :::::: :::::::::: | ACCAAAAAAAAAAAAAAA | Translation | spectrin beta, erythrocytic | 1 |
| -2.75 | UUUUUUUUUUGUUUUGGC | NM_001128627 | SPIRE1 | 2.5 | 13.315 | 1 | 18 | 318 | 335 | UUUUUUUUUUGUUUUGGC | :.::..::::::::::: | ACUAAGGCAAAAAAAAAA | Cleavage | spire type actin nucleation factor 1 | 1 |
| -2.75 | UUUUUUUUUUGUUUUGGC | NM_001145819 | SOX6 | 2 | 13.417 | 1 | 18 | 3712 | 3729 | UUUUUUUUUUGUUUUGGC | :::::: :::::::::: | CCCAAAAAAAAAAAAAAA | Translation | SRY-box transcription factor 6 | 1 |
| -2.75 | UUUUUUUUUUGUUUUGGC | NM_024798 | SNX22 | 2 | 10.753 | 1 | 18 | 1000 | 1017 | UUUUUUUUUUGUUUUGGC | :::::: :::::::::: | ACCAAAAAAAAAAAAAAA | Translation | sorting nexin 22 | 1 |
| -2.75 | UUUUUUUUUUGUUUUGGC | NM_133496 | SLC30A7 | 2 | 4.58 | 1 | 18 | 4577 | 4594 | UUUUUUUUUUGUUUUGGC | :::::: :::::::::: | UCCAAAAAAAAAAAAAAA | Translation | solute carrier family 30 member 7 | 1 |
| -2.75 | UUUUUUUUUUGUUUUGGC | NM_001144884 | SLC30A7 | 2 | 4.58 | 1 | 18 | 4261 | 4278 | UUUUUUUUUUGUUUUGGC | :::::: :::::::::: | UCCAAAAAAAAAAAAAAA | Translation | solute carrier family 30 member 7 | 1 |
| -2.75 | UUUUUUUUUUGUUUUGGC | NM_017585 | SLC2A6 | 1 | 14.097 | 1 | 18 | 786 | 803 | UUUUUUUUUUGUUUUGGC | ::::::: :::::::::: | GCCAAAAAAAAAAAAAAA | Translation | solute carrier family 2 member 6 | 1 |
| -2.75 | UUUUUUUUUUGUUUUGGC | NM_000112 | SLC26A2 | 2.5 | 13.752 | 1 | 18 | 4378 | 4395 | UUUUUUUUUUGUUUUGGC | :.::::.:::::.:::: | CCUAAAAUAAAAAGAAAA | Cleavage | solute carrier family 26 member 2 | 1 |
| -2.75 | UUUUUUUUUUGUUUUGGC | NM_001045556 | SLA | 1.5 | 15.954 | 1 | 18 | 869 | 886 | UUUUUUUUUUGUUUUGGC | ::::::: :::::::::. | GCCAAAAAAAAAAAAAAG | Translation | Src like adaptor | 1 |
| -2.75 | UUUUUUUUUUGUUUUGGC | NM_001122681 | SH3BP2 | 2 | 4.968 | 1 | 18 | 6398 | 6415 | UUUUUUUUUUGUUUUGGC | :::::: :::::::::: | ACCAAAAAAAAAAAAAAA | Translation | SH3 domain binding protein 2 | 1 |
| -2.75 | UUUUUUUUUUGUUUUGGC | NM_145799 | SEPTIN6 | 2 | 9.48 | 1 | 18 | 1906 | 1923 | UUUUUUUUUUGUUUUGGC | :::::: :::::::::: | ACCAAAAAAAAAAAAAAA | Translation | septin 6 | 1 |
| -2.75 | UUUUUUUUUUGUUUUGGC | NM_153708 | RTP1 | 2 | 6.27 | 1 | 18 | 378 | 395 | UUUUUUUUUUGUUUUGGC | :::::: :::::::::: | ACCAAAAAAAAAAAAAAA | Translation | receptor transporter protein 1 | 1 |
| -2.75 | UUUUUUUUUUGUUUUGGC | NM_001098783 | RPP14 | 2 | 12.036 | 1 | 18 | 997 | 1014 | UUUUUUUUUUGUUUUGGC | :::::: :::::::::: | ACCAAAAAAAAAAAAAAA | Translation | ribonuclease P/MRP subunit p14 | 2 |
| -2.75 | UUUUUUUUUUGUUUUGGC | NM_001098783 | RPP14 | 3 | 13.308 | 1 | 18 | 1494 | 1511 | UUUUUUUUUUGUUUUGGC | .::::: :::::::::. | CUCAAAAAAAAAAAAAAG | Translation | ribonuclease P/MRP subunit p14 | 2 |
| -2.75 | UUUUUUUUUUGUUUUGGC | NM_005012 | ROR1 | 1.5 | 9.226 | 1 | 18 | 165 | 182 | UUUUUUUUUUGUUUUGGC | ::::.:: :::::::::: | GCCAGAAAAAAAAAAAAA | Translation | receptor tyrosine kinase like orphan receptor 1 | 1 |
| -2.75 | UUUUUUUUUUGUUUUGGC | NM_145051 | RNF183 | 1.5 | 18.5 | 1 | 18 | 399 | 416 | UUUUUUUUUUGUUUUGGC | ::.:::: :::::::::: | GCUAAAAAAAAAAAAAAA | Translation | ring finger protein 183 | 1 |
| -2.75 | UUUUUUUUUUGUUUUGGC | NM_007282 | RNF13 | 1 | 18.142 | 1 | 18 | 248 | 265 | UUUUUUUUUUGUUUUGGC | ::::::::: :::::::: | GCCAAAACAUAAAAAAAA | Translation | ring finger protein 13 | 2 |
| -2.75 | UUUUUUUUUUGUUUUGGC | NM_007282 | RNF13 | 2.5 | 15.369 | 1 | 18 | 692 | 709 | UUUUUUUUUUGUUUUGGC | .::::.::::::.:::: | AUCAAAGCAAAAAGAAAA | Cleavage | ring finger protein 13 | 2 |
| -2.75 | UUUUUUUUUUGUUUUGGC | NM_005440 | RND2 | 2 | 17.685 | 1 | 18 | 1222 | 1239 | UUUUUUUUUUGUUUUGGC | :::::: :::::::::: | UCCAAAAAAAAAAAAAAA | Translation | Rho family GTPase 2 | 1 |
| -2.75 | UUUUUUUUUUGUUUUGGC | NM_001195833 | RINL | 2 | 13.372 | 1 | 18 | 120 | 137 | UUUUUUUUUUGUUUUGGC | :::::: :::::::::: | ACCAAAAAAAAAAAAAAA | Translation | Ras and Rab interactor like | 1 |
| -2.75 | UUUUUUUUUUGUUUUGGC | NM_020663 | RHOJ | 1.5 | 10.507 | 1 | 18 | 1921 | 1938 | UUUUUUUUUUGUUUUGGC | :.::::: :::::::::: | GUCAAAAAAAAAAAAAAA | Translation | ras homolog family member J | 1 |
| -2.75 | UUUUUUUUUUGUUUUGGC | NM_001171080 | RBM41 | 2 | 11.865 | 1 | 18 | 1332 | 1349 | UUUUUUUUUUGUUUUGGC | :::::: :::::::::: | ACCAAAAAAAAAAAAAAA | Translation | RNA binding motif protein 41 | 2 |
| -2.75 | UUUUUUUUUUGUUUUGGC | NM_001171080 | RBM41 | 2.5 | 7.939 | 1 | 18 | 1782 | 1799 | UUUUUUUUUUGUUUUGGC | .::::: :::::::::: | CUCAAAAAAAAAAAAAAA | Translation | RNA binding motif protein 41 | 2 |
| -2.75 | UUUUUUUUUUGUUUUGGC | NM_001164386 | RAPGEF6 | 2 | 4.92 | 1 | 18 | 1356 | 1373 | UUUUUUUUUUGUUUUGGC | :::::: :::::::::: | ACCAAAAAAAAAAAAAAA | Translation | Rap guanine nucleotide exchange factor 6 | 1 |
| -2.75 | UUUUUUUUUUGUUUUGGC | NM_175625 | RAB3IP | 2 | 7.361 | 1 | 18 | 331 | 348 | UUUUUUUUUUGUUUUGGC | ::::::: ::::::::: | GCCAAAAAAAAAAAAAAC | Translation | RAB3A interacting protein | 2 |
| -2.75 | UUUUUUUUUUGUUUUGGC | NM_022456 | RAB3IP | 2 | 7.361 | 1 | 18 | 236 | 253 | UUUUUUUUUUGUUUUGGC | ::::::: ::::::::: | GCCAAAAAAAAAAAAAAC | Translation | RAB3A interacting protein | 2 |
| -2.75 | UUUUUUUUUUGUUUUGGC | NM_175625 | RAB3IP | 2.5 | 11.901 | 1 | 18 | 3850 | 3867 | UUUUUUUUUUGUUUUGGC | ::::::::::::: ::: | UCCAAAACAAAAAAUAAA | Cleavage | RAB3A interacting protein | 2 |
| -2.75 | UUUUUUUUUUGUUUUGGC | NM_022456 | RAB3IP | 2.5 | 11.901 | 1 | 18 | 3755 | 3772 | UUUUUUUUUUGUUUUGGC | ::::::::::::: ::: | UCCAAAACAAAAAAUAAA | Cleavage | RAB3A interacting protein | 2 |
| -2.75 | UUUUUUUUUUGUUUUGGC | NM_006868 | RAB31 | 1.5 | 9.534 | 1 | 18 | 262 | 279 | UUUUUUUUUUGUUUUGGC | ::.:::: :::::::::: | GCUAAAAAAAAAAAAAAA | Translation | RAB31, member RAS oncogene family | 2 |
| -2.75 | UUUUUUUUUUGUUUUGGC | NM_006868 | RAB31 | 2 | 10.276 | 1 | 18 | 1770 | 1788 | UUUUUUUUUUG-UUUUGGC | ::::::. .:::::::::: | GCCAAAGGUAAAAAAAAAA | Translation | RAB31, member RAS oncogene family | 2 |
| -2.75 | UUUUUUUUUUGUUUUGGC | NM_001135769 | PVR | 2 | 12.333 | 1 | 18 | 3822 | 3839 | UUUUUUUUUUGUUUUGGC | :::::: :::::::::: | UCCAAAAAAAAAAAAAAA | Translation | PVR cell adhesion molecule | 1 |
| -2.75 | UUUUUUUUUUGUUUUGGC | NM_005401 | PTPN14 | 2 | 17.515 | 1 | 18 | 1942 | 1959 | UUUUUUUUUUGUUUUGGC | :::::: :::::::::: | UCCAAAAAAAAAAAAAAA | Translation | protein tyrosine phosphatase non-receptor type 14 | 1 |
| -2.75 | UUUUUUUUUUGUUUUGGC | NM_002825 | PTN | 1.5 | 9.729 | 1 | 18 | 118 | 135 | UUUUUUUUUUGUUUUGGC | :: ::::::::::.:::: | GCAAAAACAAAAAGAAAA | Cleavage | pleiotrophin | 1 |
| -2.75 | UUUUUUUUUUGUUUUGGC | NM_173495 | PTCHD1 | 1 | 13.398 | 1 | 18 | 115 | 132 | UUUUUUUUUUGUUUUGGC | ::::::: :::::::::: | GCCAAAAAAAAAAAAAAA | Translation | patched domain containing 1 | 1 |
| -2.75 | UUUUUUUUUUGUUUUGGC | NM_017952 | PTCD3 | 2 | 7.049 | 1 | 18 | 2661 | 2678 | UUUUUUUUUUGUUUUGGC | .::::.::::::::::: | CUCAAAGCAAAAAAAAAA | Cleavage | pentatricopeptide repeat domain 3 | 2 |
| -2.75 | UUUUUUUUUUGUUUUGGC | NM_017952 | PTCD3 | 2.5 | 8.505 | 1 | 18 | 3343 | 3360 | UUUUUUUUUUGUUUUGGC | .::::: :::::::::: | CUCAAAAAAAAAAAAAAA | Translation | pentatricopeptide repeat domain 3 | 2 |
| -2.75 | UUUUUUUUUUGUUUUGGC | NM_021016 | PSG3 | 2.5 | 11.028 | 1 | 18 | 228 | 245 | UUUUUUUUUUGUUUUGGC | .:::.:::::.:::::: | UUCAAGACAAAGAAAAAA | Cleavage | pregnancy specific beta-1-glycoprotein 3 | 1 |
| -2.75 | UUUUUUUUUUGUUUUGGC | NM_005044 | PRKX | 2 | 10.205 | 1 | 18 | 1067 | 1084 | UUUUUUUUUUGUUUUGGC | :::::: :::::::::: | UCCAAAAAAAAAAAAAAA | Translation | protein kinase cAMP-dependent X-linked catalytic subunit | 1 |
| -2.75 | UUUUUUUUUUGUUUUGGC | NM_147180 | PPP3R2 | 2 | 12.941 | 1 | 18 | 1630 | 1647 | UUUUUUUUUUGUUUUGGC | ::::::.:.:::::::: | ACCAAAAUAGAAAAAAAA | Cleavage | protein phosphatase 3 regulatory subunit B, beta | 1 |
| -2.75 | UUUUUUUUUUGUUUUGGC | NM_152542 | PPM1K | 2 | 6.596 | 1 | 18 | 700 | 717 | UUUUUUUUUUGUUUUGGC | :::::: :::::::::: | UCCAAAAAAAAAAAAAAA | Translation | protein phosphatase, Mg2+/Mn2+ dependent 1K | 3 |
| -2.75 | UUUUUUUUUUGUUUUGGC | NM_152542 | PPM1K | 2 | 14.647 | 1 | 18 | 1765 | 1782 | UUUUUUUUUUGUUUUGGC | :: .::::::::.::::: | GCAGAAACAAAAGAAAAA | Cleavage | protein phosphatase, Mg2+/Mn2+ dependent 1K | 3 |
| -2.75 | UUUUUUUUUUGUUUUGGC | NM_152542 | PPM1K | 2.5 | 7.007 | 1 | 18 | 881 | 898 | UUUUUUUUUUGUUUUGGC | .::::: :::::::::: | CUCAAAAAAAAAAAAAAA | Translation | protein phosphatase, Mg2+/Mn2+ dependent 1K | 3 |
| -2.75 | UUUUUUUUUUGUUUUGGC | NM_201439 | PPHLN1 | 1.5 | 10.779 | 1 | 18 | 15 | 32 | UUUUUUUUUUGUUUUGGC | ::.:::: :::::::::: | GCUAAAAAAAAAAAAAAA | Translation | periphilin 1 | 1 |
| -2.75 | UUUUUUUUUUGUUUUGGC | NM_001247994 | POU2F2 | 1 | 13.638 | 1 | 18 | 3989 | 4006 | UUUUUUUUUUGUUUUGGC | ::::::: :::::::::: | GCCAAAAAAAAAAAAAAA | Translation | POU class 2 homeobox 2 | 3 |
| -2.75 | UUUUUUUUUUGUUUUGGC | NM_002698 | POU2F2 | 1 | 13.638 | 1 | 18 | 3936 | 3953 | UUUUUUUUUUGUUUUGGC | ::::::: :::::::::: | GCCAAAAAAAAAAAAAAA | Translation | POU class 2 homeobox 2 | 3 |
| -2.75 | UUUUUUUUUUGUUUUGGC | NM_001247994 | POU2F2 | 2 | 5.227 | 1 | 18 | 491 | 508 | UUUUUUUUUUGUUUUGGC | :::::: :::::::::: | ACCAAAAAAAAAAAAAAA | Translation | POU class 2 homeobox 2 | 3 |
| -2.75 | UUUUUUUUUUGUUUUGGC | NM_001247994 | POU2F2 | 2 | 13.458 | 1 | 18 | 4628 | 4645 | UUUUUUUUUUGUUUUGGC | :: .:::::::.:::::: | GCGGAAACAAAGAAAAAA | Cleavage | POU class 2 homeobox 2 | 3 |
| -2.75 | UUUUUUUUUUGUUUUGGC | NM_002698 | POU2F2 | 2 | 5.227 | 1 | 18 | 438 | 455 | UUUUUUUUUUGUUUUGGC | :::::: :::::::::: | ACCAAAAAAAAAAAAAAA | Translation | POU class 2 homeobox 2 | 3 |
| -2.75 | UUUUUUUUUUGUUUUGGC | NM_002698 | POU2F2 | 2 | 13.458 | 1 | 18 | 4575 | 4592 | UUUUUUUUUUGUUUUGGC | :: .:::::::.:::::: | GCGGAAACAAAGAAAAAA | Cleavage | POU class 2 homeobox 2 | 3 |
| -2.75 | UUUUUUUUUUGUUUUGGC | NM_152305 | POGLUT1 | 1 | 6.076 | 1 | 18 | 706 | 723 | UUUUUUUUUUGUUUUGGC | ::::::: :::::::::: | GCCAAAAAAAAAAAAAAA | Translation | protein O-glucosyltransferase 1 | 1 |
| -2.75 | UUUUUUUUUUGUUUUGGC | NM_001114635 | PLAG1 | 2 | 10.402 | 1 | 18 | 4910 | 4927 | UUUUUUUUUUGUUUUGGC | :.:::::.:.::::.::: | GUCAAAAUAGAAAAGAAA | Cleavage | PLAG1 zinc finger | 2 |
| -2.75 | UUUUUUUUUUGUUUUGGC | NM_001114635 | PLAG1 | 2 | 8.719 | 1 | 18 | 3004 | 3021 | UUUUUUUUUUGUUUUGGC | :..:::: :::::::::: | GUUAAAAAAAAAAAAAAA | Translation | PLAG1 zinc finger | 2 |
| -2.75 | UUUUUUUUUUGUUUUGGC | NM_181524 | PIK3R1 | 2.5 | 9.521 | 1 | 18 | 3919 | 3936 | UUUUUUUUUUGUUUUGGC | .::::::::::::::: | AUCAAAACAAAAAAAAAC | Cleavage | phosphoinositide-3-kinase regulatory subunit 1 | 1 |
| -2.75 | UUUUUUUUUUGUUUUGGC | NM_032458 | PHF6 | 2.5 | 10.793 | 1 | 18 | 3182 | 3199 | UUUUUUUUUUGUUUUGGC | .::::.:::::.::::: | CUCAAAGCAAAAGAAAAA | Cleavage | PHD finger protein 6 | 1 |
| -2.75 | UUUUUUUUUUGUUUUGGC | NM_001015877 | PHF6 | 2.5 | 10.793 | 1 | 18 | 2852 | 2869 | UUUUUUUUUUGUUUUGGC | .::::.:::::.::::: | CUCAAAGCAAAAGAAAAA | Cleavage | PHD finger protein 6 | 1 |
| -2.75 | UUUUUUUUUUGUUUUGGC | NM_001202474 | PGR | 2 | 8.949 | 1 | 18 | 7508 | 7525 | UUUUUUUUUUGUUUUGGC | :::::: :::::::::: | UCCAAAAAAAAAAAAAAA | Translation | progesterone receptor | 1 |
| -2.75 | UUUUUUUUUUGUUUUGGC | NM_001011516 | PDLIM5 | 2 | 12.085 | 1 | 18 | 199 | 216 | UUUUUUUUUUGUUUUGGC | :::::: :::::::::: | ACCAAAAAAAAAAAAAAA | Translation | PDZ and LIM domain 5 | 1 |
| -2.75 | UUUUUUUUUUGUUUUGGC | NM_002570 | PCSK6 | 2.5 | 18.113 | 1 | 18 | 321 | 338 | UUUUUUUUUUGUUUUGGC | :::::.::.::::.::: | ACCAAAGCAGAAAAGAAA | Cleavage | proprotein convertase subtilisin/kexin type 6 | 1 |
| -2.75 | UUUUUUUUUUGUUUUGGC | NM_001040429 | PCDH17 | 1.5 | 6.575 | 1 | 18 | 3221 | 3238 | UUUUUUUUUUGUUUUGGC | ::.:::: :::::::::: | GCUAAAAAAAAAAAAAAA | Translation | protocadherin 17 | 2 |
| -2.75 | UUUUUUUUUUGUUUUGGC | NM_001040429 | PCDH17 | 2 | 9.234 | 1 | 18 | 612 | 629 | UUUUUUUUUUGUUUUGGC | : ::::::::::::::: | ACAAAAACAAAAAAAAAA | Cleavage | protocadherin 17 | 2 |
| -2.75 | UUUUUUUUUUGUUUUGGC | NM_032521 | PARD6B | 2 | 15.423 | 1 | 18 | 1841 | 1858 | UUUUUUUUUUGUUUUGGC | ::::::: ::::::::: | GCCAAAAAAAAAAAAAAU | Translation | par-6 family cell polarity regulator beta | 1 |
| -2.75 | UUUUUUUUUUGUUUUGGC | NM_018440 | PAG1 | 2 | 7.918 | 1 | 18 | 6422 | 6439 | UUUUUUUUUUGUUUUGGC | : ::::::::::::::: | ACAAAAACAAAAAAAAAA | Cleavage | phosphoprotein membrane anchor with glycosphingolipid microdomains 1 | 2 |
| -2.75 | UUUUUUUUUUGUUUUGGC | NM_018440 | PAG1 | 2.5 | 14.588 | 1 | 18 | 2362 | 2379 | UUUUUUUUUUGUUUUGGC | :.:::..:::::::::: | UCUAAAGUAAAAAAAAAA | Cleavage | phosphoprotein membrane anchor with glycosphingolipid microdomains 1 | 2 |
| -2.75 | UUUUUUUUUUGUUUUGGC | NM_001007525 | NWD1 | 2 | 15.636 | 1 | 18 | 2557 | 2574 | UUUUUUUUUUGUUUUGGC | :::::: :::::::::: | UCCAAAAAAAAAAAAAAA | Translation | NACHT and WD repeat domain containing 1 | 1 |
| -2.75 | UUUUUUUUUUGUUUUGGC | NM_153645 | NUP50 | 1.5 | 7.87 | 1 | 18 | 2761 | 2778 | UUUUUUUUUUGUUUUGGC | ::::::: :::::::::. | GCCAAAAAAAAAAAAAAG | Translation | nucleoporin 50 | 1 |
| -2.75 | UUUUUUUUUUGUUUUGGC | NM_022731 | NUCKS1 | 1.5 | 17.969 | 1 | 18 | 766 | 783 | UUUUUUUUUUGUUUUGGC | ::.:::: :::::::::: | GCUAAAAAAAAAAAAAAA | Translation | nuclear casein kinase and cyclin dependent kinase substrate 1 | 1 |
| -2.75 | UUUUUUUUUUGUUUUGGC | NM_199044 | NSUN4 | 2 | 16.899 | 1 | 18 | 2293 | 2310 | UUUUUUUUUUGUUUUGGC | :::::: :::::::::: | ACCAAAAAAAAAAAAAAA | Translation | NOP2/Sun RNA methyltransferase 4 | 2 |
| -2.75 | UUUUUUUUUUGUUUUGGC | NM_199044 | NSUN4 | 2.5 | 18.984 | 1 | 18 | 2484 | 2501 | UUUUUUUUUUGUUUUGGC | .::::: :::::::::: | CUCAAAAAAAAAAAAAAA | Translation | NOP2/Sun RNA methyltransferase 4 | 2 |
| -2.75 | UUUUUUUUUUGUUUUGGC | NM_001040110 | NRF1 | 1.5 | 12.161 | 1 | 18 | 1788 | 1805 | UUUUUUUUUUGUUUUGGC | :::::.::.::.:::::: | GCCAAGACGAAGAAAAAA | Cleavage | nuclear respiratory factor 1 | 1 |
| -2.75 | UUUUUUUUUUGUUUUGGC | NM_001204259 | NR3C1 | 1.5 | 8.489 | 1 | 18 | 749 | 766 | UUUUUUUUUUGUUUUGGC | :.::::: :::::::::: | GUCAAAAAAAAAAAAAAA | Translation | nuclear receptor subfamily 3 group C member 1 | 1 |
| -2.75 | UUUUUUUUUUGUUUUGGC | NM_002522 | NPTX1 | 1 | 9.592 | 1 | 18 | 3663 | 3680 | UUUUUUUUUUGUUUUGGC | ::::::: :::::::::: | GCCAAAAAAAAAAAAAAA | Translation | neuronal pentraxin 1 | 1 |
| -2.75 | UUUUUUUUUUGUUUUGGC | NM_138400 | NOM1 | 1.5 | 13.983 | 1 | 18 | 585 | 602 | UUUUUUUUUUGUUUUGGC | :.::::: :::::::::: | GUCAAAAAAAAAAAAAAA | Translation | nucleolar protein with MIF4G domain 1 | 1 |
| -2.75 | UUUUUUUUUUGUUUUGGC | NM_003634 | NIPSNAP1 | 1.5 | 9.296 | 1 | 18 | 501 | 518 | UUUUUUUUUUGUUUUGGC | ::::::::::::::: :: | GCCAAAACAAAAAAACAA | Cleavage | nipsnap homolog 1 | 1 |
| -2.75 | UUUUUUUUUUGUUUUGGC | NM_001134673 | NFIA | 2 | 1.241 | 1 | 18 | 1492 | 1509 | UUUUUUUUUUGUUUUGGC | : ::::::::::::::: | ACAAAAACAAAAAAAAAA | Cleavage | nuclear factor I A | 1 |
| -2.75 | UUUUUUUUUUGUUUUGGC | NM_005595 | NFIA | 2 | 1.241 | 1 | 18 | 1433 | 1450 | UUUUUUUUUUGUUUUGGC | : ::::::::::::::: | ACAAAAACAAAAAAAAAA | Cleavage | nuclear factor I A | 1 |
| -2.75 | UUUUUUUUUUGUUUUGGC | NM_001136021 | NFATC2 | 1.5 | 12.397 | 1 | 18 | 3616 | 3633 | UUUUUUUUUUGUUUUGGC | :.::::: :::::::::: | GUCAAAAAAAAAAAAAAA | Translation | nuclear factor of activated T cells 2 | 1 |
| -2.75 | UUUUUUUUUUGUUUUGGC | NM_173091 | NFATC2 | 1.5 | 12.397 | 1 | 18 | 3516 | 3533 | UUUUUUUUUUGUUUUGGC | :.::::: :::::::::: | GUCAAAAAAAAAAAAAAA | Translation | nuclear factor of activated T cells 2 | 1 |
| -2.75 | UUUUUUUUUUGUUUUGGC | NM_001172309 | NEXN | 2 | 7.081 | 1 | 18 | 401 | 418 | UUUUUUUUUUGUUUUGGC | : ::::::::::::::: | ACAAAAACAAAAAAAAAA | Cleavage | nexilin F-actin binding protein | 1 |
| -2.75 | UUUUUUUUUUGUUUUGGC | NM_022351 | NECAB1 | 1.5 | 6.915 | 1 | 18 | 2159 | 2176 | UUUUUUUUUUGUUUUGGC | ::::::: ::::::.::: | GCCAAAAAAAAAAAGAAA | Translation | N-terminal EF-hand calcium binding protein 1 | 1 |
| -2.75 | UUUUUUUUUUGUUUUGGC | NM_012334 | MYO10 | 2 | 16.117 | 1 | 18 | 4349 | 4366 | UUUUUUUUUUGUUUUGGC | :::::: :::::::::: | UCCAAAAAAAAAAAAAAA | Translation | myosin X | 1 |
| -2.75 | UUUUUUUUUUGUUUUGGC | NM_052845 | MMAB | 1.5 | 10.604 | 1 | 18 | 1552 | 1569 | UUUUUUUUUUGUUUUGGC | ::.:::: :::::::::: | GCUAAAAAAAAAAAAAAA | Translation | metabolism of cobalamin associated B | 2 |
| -2.75 | UUUUUUUUUUGUUUUGGC | NM_052845 | MMAB | 3 | 14.318 | 1 | 18 | 1725 | 1742 | UUUUUUUUUUGUUUUGGC | ::::: :::::::::: | CACAAAAAAAAAAAAAAA | Translation | metabolism of cobalamin associated B | 2 |
| -2.75 | UUUUUUUUUUGUUUUGGC | NM_172250 | MMAA | 2.5 | 11.758 | 1 | 18 | 3250 | 3267 | UUUUUUUUUUGUUUUGGC | :.::..::::::::::: | UCUAAGGCAAAAAAAAAA | Cleavage | metabolism of cobalamin associated A | 1 |
| -2.75 | UUUUUUUUUUGUUUUGGC | NM_001098624 | MID1 | 2 | 6.526 | 1 | 18 | 1240 | 1257 | UUUUUUUUUUGUUUUGGC | :::::: :::::::::: | ACCAAAAAAAAAAAAAAA | Translation | midline 1 | 1 |
| -2.75 | UUUUUUUUUUGUUUUGGC | NM_001136004 | MICAL3 | 1.5 | 8.248 | 1 | 18 | 2706 | 2723 | UUUUUUUUUUGUUUUGGC | ::::::::::::: :::: | GCCAAAACAAAAACAAAA | Cleavage | microtubule associated monooxygenase, calponin and LIM domain containing 3 | 1 |
| -2.75 | UUUUUUUUUUGUUUUGGC | NM_001256585 | MGLL | 1 | 13.759 | 1 | 18 | 80 | 97 | UUUUUUUUUUGUUUUGGC | ::::::: :::::::::: | GCCAAAAAAAAAAAAAAA | Translation | monoglyceride lipase | 1 |
| -2.75 | UUUUUUUUUUGUUUUGGC | NM_024770 | METTL8 | 2 | 13.201 | 1 | 18 | 919 | 936 | UUUUUUUUUUGUUUUGGC | :::::: :::::::::: | UCCAAAAAAAAAAAAAAA | Translation | methyltransferase 8, tRNA N3-cytidine | 2 |
| -2.75 | UUUUUUUUUUGUUUUGGC | NM_024770 | METTL8 | 2.5 | 8.662 | 1 | 18 | 2190 | 2207 | UUUUUUUUUUGUUUUGGC | .::::: :::::::::: | AUCAAAAAAAAAAAAAAA | Translation | methyltransferase 8, tRNA N3-cytidine | 2 |
| -2.75 | UUUUUUUUUUGUUUUGGC | NM_001270875 | MEAF6 | 1.5 | 23.399 | 1 | 18 | 2164 | 2181 | UUUUUUUUUUGUUUUGGC | :.::::: :::::::::: | GUCAAAAAAAAAAAAAAA | Translation | MYST/Esa1 associated factor 6 | 1 |
| -2.75 | UUUUUUUUUUGUUUUGGC | NM_001270876 | MEAF6 | 1.5 | 23.399 | 1 | 18 | 1834 | 1851 | UUUUUUUUUUGUUUUGGC | :.::::: :::::::::: | GUCAAAAAAAAAAAAAAA | Translation | MYST/Esa1 associated factor 6 | 1 |
| -2.75 | UUUUUUUUUUGUUUUGGC | NM_002393 | MDM4 | 1.5 | 11.331 | 1 | 18 | 2295 | 2312 | UUUUUUUUUUGUUUUGGC | ::.:::: :::::::::: | GCUAAAAAAAAAAAAAAA | Translation | MDM4 regulator of p53 | 1 |
| -2.75 | UUUUUUUUUUGUUUUGGC | NM_001031804 | MAF | 2 | 16.215 | 1 | 18 | 743 | 760 | UUUUUUUUUUGUUUUGGC | ::.::::.:.:.:::::: | GCUAAAAUAGAGAAAAAA | Cleavage | MAF bZIP transcription factor | 1 |
| -2.75 | UUUUUUUUUUGUUUUGGC | NM_025061 | LRRC8E | 2 | 9.699 | 1 | 18 | 799 | 816 | UUUUUUUUUUGUUUUGGC | ::::::: ::::::::: | GCCAAAAAAAAAAAAAAU | Translation | leucine rich repeat containing 8 VRAC subunit E | 1 |
| -2.75 | UUUUUUUUUUGUUUUGGC | NM_001277127 | LRRC31 | 2.5 | 14.637 | 1 | 18 | 296 | 313 | UUUUUUUUUUGUUUUGGC | ::::.:.:::::::.:: | ACCAAGAUAAAAAAAGAA | Cleavage | leucine rich repeat containing 31 | 1 |
| -2.75 | UUUUUUUUUUGUUUUGGC | NM_145175 | LRATD1 | 2 | 5.474 | 1 | 18 | 3353 | 3370 | UUUUUUUUUUGUUUUGGC | :::::: :::::::::: | UCCAAAAAAAAAAAAAAA | Translation | LRAT domain containing 1 | 1 |
| -2.75 | UUUUUUUUUUGUUUUGGC | NM_001261428 | LPIN1 | 1.5 | 10.729 | 1 | 18 | 1720 | 1737 | UUUUUUUUUUGUUUUGGC | :.::::: :::::::::: | GUCAAAAAAAAAAAAAAA | Translation | lipin 1 | 1 |
| -2.75 | UUUUUUUUUUGUUUUGGC | NM_015155 | LARP4B | 2 | 17.196 | 1 | 18 | 2123 | 2140 | UUUUUUUUUUGUUUUGGC | :::::: :::::::::: | ACCAAAAAAAAAAAAAAA | Translation | La ribonucleoprotein 4B | 1 |
| -2.75 | UUUUUUUUUUGUUUUGGC | NM_015315 | LARP1 | 2 | 5.926 | 1 | 18 | 801 | 818 | UUUUUUUUUUGUUUUGGC | :::::: :::::::::: | ACCAAAAAAAAAAAAAAA | Translation | La ribonucleoprotein 1, translational regulator | 1 |
| -2.75 | UUUUUUUUUUGUUUUGGC | NM_002262 | KLRD1 | 2 | 14.529 | 1 | 18 | 1322 | 1339 | UUUUUUUUUUGUUUUGGC | :::::: :::::::::: | UCCAAAAAAAAAAAAAAA | Translation | killer cell lectin like receptor D1 | 1 |
| -2.75 | UUUUUUUUUUGUUUUGGC | NM_001270942 | KLF7 | 2 | 1.847 | 1 | 18 | 2143 | 2160 | UUUUUUUUUUGUUUUGGC | : ::::::::::::::: | ACAAAAACAAAAAAAAAA | Cleavage | KLF transcription factor 7 | 2 |
| -2.75 | UUUUUUUUUUGUUUUGGC | NM_001270943 | KLF7 | 2 | 1.847 | 1 | 18 | 2129 | 2146 | UUUUUUUUUUGUUUUGGC | : ::::::::::::::: | ACAAAAACAAAAAAAAAA | Cleavage | KLF transcription factor 7 | 2 |
| -2.75 | UUUUUUUUUUGUUUUGGC | NM_001270942 | KLF7 | 2.5 | 5.109 | 1 | 18 | 2170 | 2187 | UUUUUUUUUUGUUUUGGC | ::::::.::::::::: | UACAAAACGAAAAAAAAA | Cleavage | KLF transcription factor 7 | 2 |
| -2.75 | UUUUUUUUUUGUUUUGGC | NM_001270943 | KLF7 | 2.5 | 5.109 | 1 | 18 | 2156 | 2173 | UUUUUUUUUUGUUUUGGC | ::::::.::::::::: | UACAAAACGAAAAAAAAA | Cleavage | KLF transcription factor 7 | 2 |
| -2.75 | UUUUUUUUUUGUUUUGGC | NM_175737 | KLB | 2 | 13.443 | 1 | 18 | 632 | 649 | UUUUUUUUUUGUUUUGGC | :::::: :::::::::: | UCCAAAAAAAAAAAAAAA | Translation | klotho beta | 2 |
| -2.75 | UUUUUUUUUUGUUUUGGC | NM_175737 | KLB | 3 | 15.9 | 1 | 18 | 1236 | 1253 | UUUUUUUUUUGUUUUGGC | .:::::.::::::::: | CUCAAAAUAAAAAAAAAU | Cleavage | klotho beta | 2 |
| -2.75 | UUUUUUUUUUGUUUUGGC | NM_007054 | KIF3A | 2.5 | 11.005 | 1 | 18 | 348 | 365 | UUUUUUUUUUGUUUUGGC | .:::::.:.:::::::: | UUCAAAAUAGAAAAAAAA | Cleavage | kinesin family member 3A | 1 |
| -2.75 | UUUUUUUUUUGUUUUGGC | NM_024874 | KIAA0319L | 1.5 | 11.841 | 1 | 18 | 787 | 804 | UUUUUUUUUUGUUUUGGC | :.::::: :::::::::: | GUCAAAAAAAAAAAAAAA | Translation | KIAA0319 like | 1 |
| -2.75 | UUUUUUUUUUGUUUUGGC | NM_003685 | KHSRP | 1 | 9.692 | 1 | 18 | 269 | 286 | UUUUUUUUUUGUUUUGGC | ::::::: :::::::::: | GCCAAAAAAAAAAAAAAA | Translation | KH-type splicing regulatory protein | 1 |
| -2.75 | UUUUUUUUUUGUUUUGGC | NM_020122 | KCMF1 | 1.5 | 9.347 | 1 | 18 | 4969 | 4986 | UUUUUUUUUUGUUUUGGC | .:::::::::::::::: | CUCAAAACAAAAAAAAAA | Cleavage | potassium channel modulatory factor 1 | 1 |
| -2.75 | UUUUUUUUUUGUUUUGGC | NM_032817 | ITIH5 | 2.5 | 10.889 | 1 | 18 | 3224 | 3241 | UUUUUUUUUUGUUUUGGC | ::::::::::::::.: | AACAAAACAAAAAAAAGA | Cleavage | inter-alpha-trypsin inhibitor heavy chain 5 | 1 |
| -2.75 | UUUUUUUUUUGUUUUGGC | NM_002214 | ITGB8 | 2.5 | 10.927 | 1 | 18 | 3236 | 3253 | UUUUUUUUUUGUUUUGGC | ..::::.:::::::::: | AUUAAAAUAAAAAAAAAA | Cleavage | integrin subunit beta 8 | 1 |
| -2.75 | UUUUUUUUUUGUUUUGGC | NM_001199469 | ISY1 | 2 | 23.304 | 1 | 18 | 686 | 703 | UUUUUUUUUUGUUUUGGC | :::::: :::::::::: | CCCAAAAAAAAAAAAAAA | Translation | ISY1 splicing factor homolog | 1 |
| -2.75 | UUUUUUUUUUGUUUUGGC | NM_004136 | IREB2 | 2 | 13.33 | 1 | 18 | 2393 | 2410 | UUUUUUUUUUGUUUUGGC | :::::: :::::::::: | ACCAAAAAAAAAAAAAAA | Translation | iron responsive element binding protein 2 | 1 |
| -2.75 | UUUUUUUUUUGUUUUGGC | NM_002192 | INHBA | 2 | 4.4 | 1 | 18 | 108 | 125 | UUUUUUUUUUGUUUUGGC | :::::::::::::::: | AACAAAACAAAAAAAAAA | Cleavage | inhibin subunit beta A | 1 |
| -2.75 | UUUUUUUUUUGUUUUGGC | NM_000599 | IGFBP5 | 1.5 | 11.626 | 1 | 18 | 4429 | 4446 | UUUUUUUUUUGUUUUGGC | :::.::: :::::::::: | GCCGAAAAAAAAAAAAAA | Translation | insulin like growth factor binding protein 5 | 1 |
| -2.75 | UUUUUUUUUUGUUUUGGC | NM_006460 | HEXIM1 | 1 | 6.745 | 1 | 18 | 1029 | 1046 | UUUUUUUUUUGUUUUGGC | ::::::::::::::::: | ACCAAAACAAAAAAAAAA | Cleavage | HEXIM P-TEFb complex subunit 1 | 1 |
| -2.75 | UUUUUUUUUUGUUUUGGC | NM_001884 | HAPLN1 | 2 | 15.1 | 1 | 18 | 567 | 584 | UUUUUUUUUUGUUUUGGC | :::::: :::::::::: | ACCAAAAAAAAAAAAAAA | Translation | hyaluronan and proteoglycan link protein 1 | 1 |
| -2.75 | UUUUUUUUUUGUUUUGGC | NM_001267614 | GPR161 | 2 | 22.006 | 1 | 18 | 246 | 263 | UUUUUUUUUUGUUUUGGC | :.:::.::::::::::: | UCUAAAGCAAAAAAAAAA | Cleavage | G protein-coupled receptor 161 | 1 |
| -2.75 | UUUUUUUUUUGUUUUGGC | NM_001184819 | GNL3L | 2 | 8.743 | 1 | 18 | 467 | 484 | UUUUUUUUUUGUUUUGGC | :::::: :::::::::: | CCCAAAAAAAAAAAAAAA | Translation | G protein nucleolar 3 like | 1 |
| -2.75 | UUUUUUUUUUGUUUUGGC | NM_182978 | GNAL | 1 | 16.579 | 1 | 18 | 1547 | 1564 | UUUUUUUUUUGUUUUGGC | ::::::: :::::::::: | GCCAAAAAAAAAAAAAAA | Translation | G protein subunit alpha L | 1 |
| -2.75 | UUUUUUUUUUGUUUUGGC | NM_017660 | GATAD2A | 2 | 16.321 | 1 | 18 | 1775 | 1792 | UUUUUUUUUUGUUUUGGC | :::::: :::::::::: | ACCAAAAAAAAAAAAAAA | Translation | GATA zinc finger domain containing 2A | 1 |
| -2.75 | UUUUUUUUUUGUUUUGGC | NM_021167 | GATAD1 | 2 | 20.003 | 1 | 18 | 2660 | 2677 | UUUUUUUUUUGUUUUGGC | :::::: :::::::::: | UCCAAAAAAAAAAAAAAA | Translation | GATA zinc finger domain containing 1 | 2 |
| -2.75 | UUUUUUUUUUGUUUUGGC | NM_021167 | GATAD1 | 3 | 12.055 | 1 | 18 | 1570 | 1587 | UUUUUUUUUUGUUUUGGC | .::::: :.:::::::: | CUCAAAAAAGAAAAAAAA | Translation | GATA zinc finger domain containing 1 | 2 |
| -2.75 | UUUUUUUUUUGUUUUGGC | NM_000812 | GABRB1 | 1 | 7.432 | 1 | 18 | 169 | 186 | UUUUUUUUUUGUUUUGGC | : :::::::::::::::: | GACAAAACAAAAAAAAAA | Cleavage | gamma-aminobutyric acid type A receptor subunit beta1 | 1 |
| -2.75 | UUUUUUUUUUGUUUUGGC | NM_145866 | FZD3 | 2 | 11.451 | 1 | 18 | 8014 | 8031 | UUUUUUUUUUGUUUUGGC | :::::: :::::::::: | ACCAAAAAAAAAAAAAAA | Translation | frizzled class receptor 3 | 1 |
| -2.75 | UUUUUUUUUUGUUUUGGC | NM_033260 | FOXQ1 | 1.5 | 17.951 | 1 | 18 | 524 | 541 | UUUUUUUUUUGUUUUGGC | ::::::: :.:::::::: | GCCAAAAGAGAAAAAAAA | Translation | forkhead box Q1 | 1 |
| -2.75 | UUUUUUUUUUGUUUUGGC | NM_001455 | FOXO3 | 1.5 | 9.842 | 1 | 18 | 3892 | 3909 | UUUUUUUUUUGUUUUGGC | :.::::: :::::::::: | GUCAAAAAAAAAAAAAAA | Translation | forkhead box O3 | 1 |
| -2.75 | UUUUUUUUUUGUUUUGGC | NM_139241 | FGD4 | 2.5 | 17.35 | 1 | 18 | 495 | 512 | UUUUUUUUUUGUUUUGGC | ..:::::::::.::::: | UUUAAAACAAAAGAAAAA | Cleavage | FYVE, RhoGEF and PH domain containing 4 | 2 |
| -2.75 | UUUUUUUUUUGUUUUGGC | NM_139241 | FGD4 | 3 | 11.922 | 1 | 18 | 4348 | 4365 | UUUUUUUUUUGUUUUGGC | :..:..::::::::::: | ACUGAGGCAAAAAAAAAA | Cleavage | FYVE, RhoGEF and PH domain containing 4 | 2 |
| -2.75 | UUUUUUUUUUGUUUUGGC | NM_203301 | FBXO33 | 2 | 4.743 | 1 | 18 | 1027 | 1044 | UUUUUUUUUUGUUUUGGC | :::::::::::::::: | AACAAAACAAAAAAAAAA | Cleavage | F-box protein 33 | 1 |
| -2.75 | UUUUUUUUUUGUUUUGGC | NM_001201543 | FAM161A | 2 | 12.463 | 1 | 18 | 798 | 815 | UUUUUUUUUUGUUUUGGC | :::::: :::::::::: | UCCAAAAAAAAAAAAAAA | Translation | FAM161 centrosomal protein A | 1 |
| -2.75 | UUUUUUUUUUGUUUUGGC | NM_001076778 | FAM107A | 1 | 16.643 | 1 | 18 | 1125 | 1142 | UUUUUUUUUUGUUUUGGC | ::::::: :::::::::: | GCCAAAAAAAAAAAAAAA | Translation | family with sequence similarity 107 member A | 1 |
| -2.75 | UUUUUUUUUUGUUUUGGC | NM_001424 | EMP2 | 1 | 9.806 | 1 | 18 | 116 | 133 | UUUUUUUUUUGUUUUGGC | ::::::: :::::::::: | GCCAAAAAAAAAAAAAAA | Translation | epithelial membrane protein 2 | 1 |
| -2.75 | UUUUUUUUUUGUUUUGGC | NM_001204510 | EIF4A1 | 2 | 10.355 | 1 | 18 | 327 | 344 | UUUUUUUUUUGUUUUGGC | :::::: :::::::::: | CCCAAAAAAAAAAAAAAA | Translation | eukaryotic translation initiation factor 4A1 | 1 |
| -2.75 | UUUUUUUUUUGUUUUGGC | NM_001416 | EIF4A1 | 2 | 10.355 | 1 | 18 | 212 | 229 | UUUUUUUUUUGUUUUGGC | :::::: :::::::::: | CCCAAAAAAAAAAAAAAA | Translation | eukaryotic translation initiation factor 4A1 | 1 |
| -2.75 | UUUUUUUUUUGUUUUGGC | NM_013302 | EEF2K | 2 | 15.936 | 1 | 18 | 1659 | 1676 | UUUUUUUUUUGUUUUGGC | ::::::::: ::::::: | ACCAAAACAACAAAAAAA | Cleavage | eukaryotic elongation factor 2 kinase | 1 |
| -2.75 | UUUUUUUUUUGUUUUGGC | NM_000115 | EDNRB | 1.5 | 9.793 | 1 | 18 | 72 | 89 | UUUUUUUUUUGUUUUGGC | :::::::::::: ::::: | GCCAAAACAAAACAAAAA | Cleavage | endothelin receptor type B | 1 |
| -2.75 | UUUUUUUUUUGUUUUGGC | NM_006141 | DYNC1LI2 | 2.5 | 14.074 | 1 | 18 | 2371 | 2388 | UUUUUUUUUUGUUUUGGC | ..::::.:::::::::: | UUUAAAAUAAAAAAAAAA | Cleavage | dynein cytoplasmic 1 light intermediate chain 2 | 3 |
| -2.75 | UUUUUUUUUUGUUUUGGC | NM_006141 | DYNC1LI2 | 3 | 13.609 | 1 | 18 | 119 | 136 | UUUUUUUUUUGUUUUGGC | ..:::: :::::::::: | UUUAAAAAAAAAAAAAAA | Translation | dynein cytoplasmic 1 light intermediate chain 2 | 3 |
| -2.75 | UUUUUUUUUUGUUUUGGC | NM_006141 | DYNC1LI2 | 3 | 17.983 | 1 | 18 | 1372 | 1389 | UUUUUUUUUUGUUUUGGC | :.:::: :::...::::: | GUCAAACCAAGGGAAAAA | Cleavage | dynein cytoplasmic 1 light intermediate chain 2 | 3 |
| -2.75 | UUUUUUUUUUGUUUUGGC | NM_001012729 | DUXA | 2 | 6.35 | 1 | 18 | 1134 | 1151 | UUUUUUUUUUGUUUUGGC | :::::::::::::::: | AACAAAACAAAAAAAAAA | Cleavage | double homeobox A | 1 |
| -2.75 | UUUUUUUUUUGUUUUGGC | NM_001946 | DUSP6 | 2.5 | 13.719 | 1 | 18 | 618 | 635 | UUUUUUUUUUGUUUUGGC | :::::::::::::.:: | AACAAAACAAAAAAAGAA | Cleavage | dual specificity phosphatase 6 | 1 |
| -2.75 | UUUUUUUUUUGUUUUGGC | NM_001009934 | DNASE1L1 | 2.5 | 14.476 | 1 | 18 | 875 | 892 | UUUUUUUUUUGUUUUGGC | ::::...:::::::::: | CCCAAGGUAAAAAAAAAA | Cleavage | deoxyribonuclease 1 like 1 | 1 |
| -2.75 | UUUUUUUUUUGUUUUGGC | NM_015291 | DNAJC16 | 2 | 10.866 | 1 | 18 | 2834 | 2851 | UUUUUUUUUUGUUUUGGC | :::::: :::::::::: | UCCAAAAAAAAAAAAAAA | Translation | DnaJ heat shock protein family (Hsp40) member C16 | 1 |
| -2.75 | UUUUUUUUUUGUUUUGGC | NM_001037954 | DIXDC1 | 2 | 13.592 | 1 | 18 | 2941 | 2958 | UUUUUUUUUUGUUUUGGC | :::::: :::::::::: | UCCAAAAAAAAAAAAAAA | Translation | DIX domain containing 1 | 1 |
| -2.75 | UUUUUUUUUUGUUUUGGC | NM_000555 | DCX | 2 | 12.429 | 1 | 18 | 1052 | 1069 | UUUUUUUUUUGUUUUGGC | :::::: :::::::::: | ACCAAAAAAAAAAAAAAA | Translation | doublecortin | 1 |
| -2.75 | UUUUUUUUUUGUUUUGGC | NM_001242377 | DCP2 | 2 | 9.831 | 1 | 18 | 7900 | 7917 | UUUUUUUUUUGUUUUGGC | :::::: :::::::::: | ACCAAAAAAAAAAAAAAA | Translation | decapping mRNA 2 | 1 |
| -2.75 | UUUUUUUUUUGUUUUGGC | NM_177538 | CYP20A1 | 2 | 12.05 | 1 | 18 | 4285 | 4302 | UUUUUUUUUUGUUUUGGC | :::::: :::::::::: | UCCAAAAAAAAAAAAAAA | Translation | cytochrome P450 family 20 subfamily A member 1 | 1 |
| -2.75 | UUUUUUUUUUGUUUUGGC | NM_018947 | CYCS | 2 | 12.539 | 1 | 18 | 3593 | 3610 | UUUUUUUUUUGUUUUGGC | :::::: :::::::::: | UCCAAAAAAAAAAAAAAA | Translation | cytochrome c, somatic | 1 |
| -2.75 | UUUUUUUUUUGUUUUGGC | NM_003478 | CUL5 | 2 | 4.274 | 1 | 18 | 2450 | 2467 | UUUUUUUUUUGUUUUGGC | :::::: :::::::::: | UCCAAAAAAAAAAAAAAA | Translation | cullin 5 | 1 |
| -2.75 | UUUUUUUUUUGUUUUGGC | NM_001269044 | CTCFL | 2 | 7.138 | 1 | 18 | 146 | 163 | UUUUUUUUUUGUUUUGGC | :::::: :::::::::: | ACCAAAAAAAAAAAAAAA | Translation | CCCTC-binding factor like | 1 |
| -2.75 | UUUUUUUUUUGUUUUGGC | NM_052854 | CREB3L1 | 2 | 12.751 | 1 | 18 | 644 | 661 | UUUUUUUUUUGUUUUGGC | :::::: :::::::::: | ACCAAAAAAAAAAAAAAA | Translation | cAMP responsive element binding protein 3 like 1 | 1 |
| -2.75 | UUUUUUUUUUGUUUUGGC | NM_003389 | CORO2A | 2 | 15.257 | 1 | 18 | 1259 | 1276 | UUUUUUUUUUGUUUUGGC | :::::: :::::::::: | UCCAAAAAAAAAAAAAAA | Translation | coronin 2A | 2 |
| -2.75 | UUUUUUUUUUGUUUUGGC | NM_003389 | CORO2A | 2.5 | 8.441 | 1 | 18 | 741 | 758 | UUUUUUUUUUGUUUUGGC | .::::: :::::::::: | CUCAAAAAAAAAAAAAAA | Translation | coronin 2A | 2 |
| -2.75 | UUUUUUUUUUGUUUUGGC | NM_000088 | COL1A1 | 2 | 4.842 | 1 | 18 | 223 | 240 | UUUUUUUUUUGUUUUGGC | :::::: :::::::::: | ACCAAAAAAAAAAAAAAA | Translation | collagen type I alpha 1 chain | 1 |
| -2.75 | UUUUUUUUUUGUUUUGGC | NM_003654 | CHST1 | 1.5 | 13.972 | 1 | 18 | 714 | 731 | UUUUUUUUUUGUUUUGGC | ::::::: .::::::::: | GCCAAAAAGAAAAAAAAA | Translation | carbohydrate sulfotransferase 1 | 1 |
| -2.75 | UUUUUUUUUUGUUUUGGC | NM_014157 | CFAP263 | 2 | 15.267 | 1 | 18 | 3043 | 3060 | UUUUUUUUUUGUUUUGGC | ::::.:.:::.:::::.: | GCCAGAGCAAGAAAAAGA | Cleavage | cilia and flagella associated protein 263 | 1 |
| -2.75 | UUUUUUUUUUGUUUUGGC | NM_018718 | CEP41 | 2 | 5.522 | 1 | 18 | 1355 | 1372 | UUUUUUUUUUGUUUUGGC | :::::: :::::::::: | ACCAAAAAAAAAAAAAAA | Translation | centrosomal protein 41 | 1 |
| -2.75 | UUUUUUUUUUGUUUUGGC | NM_020802 | CEP126 | 1 | 9.058 | 1 | 18 | 1843 | 1860 | UUUUUUUUUUGUUUUGGC | ::::::: :::::::::: | GCCAAAAAAAAAAAAAAA | Translation | centrosomal protein 126 | 3 |
| -2.75 | UUUUUUUUUUGUUUUGGC | NM_020802 | CEP126 | 2.5 | 20.114 | 1 | 18 | 947 | 964 | UUUUUUUUUUGUUUUGGC | .::::: :::::::::: | CUCAAAAAAAAAAAAAAA | Translation | centrosomal protein 126 | 3 |
| -2.75 | UUUUUUUUUUGUUUUGGC | NM_020802 | CEP126 | 3 | 16.483 | 1 | 18 | 3046 | 3063 | UUUUUUUUUUGUUUUGGC | ..:::: :::::::::: | CUUAAAAGAAAAAAAAAA | Translation | centrosomal protein 126 | 3 |
| -2.75 | UUUUUUUUUUGUUUUGGC | NM_001792 | CDH2 | 2 | 9.411 | 1 | 18 | 974 | 991 | UUUUUUUUUUGUUUUGGC | :::::: :::::::::: | ACCAAAAAAAAAAAAAAA | Translation | cadherin 2 | 1 |
| -2.75 | UUUUUUUUUUGUUUUGGC | NM_003671 | CDC14B | 1.5 | 14.061 | 1 | 18 | 1139 | 1156 | UUUUUUUUUUGUUUUGGC | ::.:::: :::::::::: | GCUAAAAAAAAAAAAAAA | Translation | cell division cycle 14B | 1 |
| -2.75 | UUUUUUUUUUGUUUUGGC | NM_004367 | CCR6 | 2 | 11.967 | 1 | 18 | 389 | 406 | UUUUUUUUUUGUUUUGGC | :.::::::.:::::::: | ACUAAAACAGAAAAAAAA | Cleavage | C-C motif chemokine receptor 6 | 2 |
| -2.75 | UUUUUUUUUUGUUUUGGC | NM_004367 | CCR6 | 2.5 | 15.671 | 1 | 18 | 1571 | 1588 | UUUUUUUUUUGUUUUGGC | .::::: :::::::::: | CUCAAAAAAAAAAAAAAA | Translation | C-C motif chemokine receptor 6 | 2 |
| -2.75 | UUUUUUUUUUGUUUUGGC | NM_025059 | CCDC170 | 2 | 11.066 | 1 | 18 | 2040 | 2057 | UUUUUUUUUUGUUUUGGC | :::::: :::::::::: | UCCAAAAAAAAAAAAAAA | Translation | coiled-coil domain containing 170 | 2 |
| -2.75 | UUUUUUUUUUGUUUUGGC | NM_025059 | CCDC170 | 3 | 3.305 | 1 | 18 | 851 | 868 | UUUUUUUUUUGUUUUGGC | .::.:: :::::::::: | CUCAGAAAAAAAAAAAAA | Translation | coiled-coil domain containing 170 | 2 |
| -2.75 | UUUUUUUUUUGUUUUGGC | NM_001199671 | CALU | 1 | 10.214 | 1 | 18 | 1407 | 1424 | UUUUUUUUUUGUUUUGGC | ::::::: :::::::::: | GCCAAAAAAAAAAAAAAA | Translation | calumenin | 1 |
| -2.75 | UUUUUUUUUUGUUUUGGC | NM_001199673 | CALU | 1 | 10.214 | 1 | 18 | 1480 | 1497 | UUUUUUUUUUGUUUUGGC | ::::::: :::::::::: | GCCAAAAAAAAAAAAAAA | Translation | calumenin | 1 |
| -2.75 | UUUUUUUUUUGUUUUGGC | NM_001167674 | CADM2 | 2.5 | 9.117 | 1 | 18 | 3844 | 3861 | UUUUUUUUUUGUUUUGGC | :::::::::::::::. | UACAAAACAAAAAAAAAG | Cleavage | cell adhesion molecule 2 | 2 |
| -2.75 | UUUUUUUUUUGUUUUGGC | NM_001167674 | CADM2 | 3 | 2.897 | 1 | 18 | 5626 | 5643 | UUUUUUUUUUGUUUUGGC | ::::::::::::::: | AAAAAAACAAAAAAAAAA | Cleavage | cell adhesion molecule 2 | 2 |
| -2.75 | UUUUUUUUUUGUUUUGGC | NM_014333 | CADM1 | 2 | 11.659 | 1 | 18 | 2188 | 2205 | UUUUUUUUUUGUUUUGGC | : ::::::::::::::: | ACAAAAACAAAAAAAAAA | Cleavage | cell adhesion molecule 1 | 1 |
| -2.75 | UUUUUUUUUUGUUUUGGC | NM_006078 | CACNG2 | 2.5 | 11.782 | 1 | 18 | 415 | 432 | UUUUUUUUUUGUUUUGGC | ..:::.::::::::::: | AUUAAAGCAAAAAAAAAA | Cleavage | calcium voltage-gated channel auxiliary subunit gamma 2 | 2 |
| -2.75 | UUUUUUUUUUGUUUUGGC | NM_006078 | CACNG2 | 2.5 | 7.904 | 1 | 18 | 102 | 119 | UUUUUUUUUUGUUUUGGC | :: ::::::::::: ::: | GCAAAAACAAAAAACAAA | Cleavage | calcium voltage-gated channel auxiliary subunit gamma 2 | 2 |
| -2.75 | UUUUUUUUUUGUUUUGGC | NM_001129841 | CACNA1C | 2 | 13.381 | 1 | 18 | 5402 | 5419 | UUUUUUUUUUGUUUUGGC | :::::: :::::::::: | UCCAAAAAAAAAAAAAAA | Translation | calcium voltage-gated channel subunit alpha1 C | 1 |
| -2.75 | UUUUUUUUUUGUUUUGGC | NM_017688 | BSPRY | 1.5 | 10.927 | 1 | 18 | 266 | 283 | UUUUUUUUUUGUUUUGGC | ::.:::: :::::::::: | GCUAAAAAAAAAAAAAAA | Translation | B-box and SPRY domain containing | 1 |
| -2.75 | UUUUUUUUUUGUUUUGGC | NM_004331 | BNIP3L | 2 | 9.505 | 1 | 18 | 510 | 527 | UUUUUUUUUUGUUUUGGC | :::::::::::::::: | AACAAAACAAAAAAAAAA | Cleavage | BCL2 interacting protein 3 like | 1 |
| -2.75 | UUUUUUUUUUGUUUUGGC | NM_022898 | BCL11B | 2.5 | 16.657 | 1 | 18 | 2270 | 2287 | UUUUUUUUUUGUUUUGGC | ::::::::.::::::: | UACAAAACAAGAAAAAAA | Cleavage | BCL11 transcription factor B | 1 |
| -2.75 | UUUUUUUUUUGUUUUGGC | NM_001178093 | BCAT1 | 2.5 | 12.667 | 1 | 18 | 7570 | 7587 | UUUUUUUUUUGUUUUGGC | :::::::::::.:::: | UACAAAACAAAAAGAAAA | Cleavage | branched chain amino acid transaminase 1 | 2 |
| -2.75 | UUUUUUUUUUGUUUUGGC | NM_001178093 | BCAT1 | 3 | 5.307 | 1 | 18 | 3180 | 3197 | UUUUUUUUUUGUUUUGGC | .::.:::::::::::: | UGUAAGACAAAAAAAAAA | Cleavage | branched chain amino acid transaminase 1 | 2 |
| -2.75 | UUUUUUUUUUGUUUUGGC | NM_001142568 | BBX | 2 | 4.503 | 1 | 18 | 386 | 403 | UUUUUUUUUUGUUUUGGC | :::::::::::::::: | AACAAAACAAAAAAAAAA | Cleavage | BBX high mobility group box domain containing | 2 |
| -2.75 | UUUUUUUUUUGUUUUGGC | NM_001142568 | BBX | 2 | 13.465 | 1 | 18 | 656 | 673 | UUUUUUUUUUGUUUUGGC | : ::::: :::::::::: | GGCAAAAAAAAAAAAAAA | Translation | BBX high mobility group box domain containing | 2 |
| -2.75 | UUUUUUUUUUGUUUUGGC | NM_001276286 | BBX | 2 | 4.503 | 1 | 18 | 316 | 333 | UUUUUUUUUUGUUUUGGC | :::::::::::::::: | AACAAAACAAAAAAAAAA | Cleavage | BBX high mobility group box domain containing | 2 |
| -2.75 | UUUUUUUUUUGUUUUGGC | NM_001276286 | BBX | 2 | 13.465 | 1 | 18 | 586 | 603 | UUUUUUUUUUGUUUUGGC | : ::::: :::::::::: | GGCAAAAAAAAAAAAAAA | Translation | BBX high mobility group box domain containing | 2 |
| -2.75 | UUUUUUUUUUGUUUUGGC | NM_002973 | ATXN2 | 1.5 | 12.167 | 1 | 18 | 359 | 376 | UUUUUUUUUUGUUUUGGC | ::.:::: :::::::::: | GCUAAAAAAAAAAAAAAA | Translation | ataxin 2 | 1 |
| -2.75 | UUUUUUUUUUGUUUUGGC | NM_000489 | ATRX | 2 | 8.323 | 1 | 18 | 1425 | 1442 | UUUUUUUUUUGUUUUGGC | :::::: :::::::::: | UCCAAAAAAAAAAAAAAA | Translation | ATRX chromatin remodeler | 1 |
| -2.75 | UUUUUUUUUUGUUUUGGC | NM_033064 | ATCAY | 2.5 | 11.205 | 1 | 18 | 1712 | 1729 | UUUUUUUUUUGUUUUGGC | .:::::.:::::.:::: | CUCAAAAUAAAAAGAAAA | Cleavage | ATCAY kinesin light chain interacting caytaxin | 1 |
| -2.75 | UUUUUUUUUUGUUUUGGC | NM_024708 | ASB7 | 2.5 | 8.983 | 1 | 18 | 131 | 148 | UUUUUUUUUUGUUUUGGC | :..:::.:::::::::: | UCUGAAAUAAAAAAAAAA | Cleavage | ankyrin repeat and SOCS box containing 7 | 1 |
| -2.75 | UUUUUUUUUUGUUUUGGC | NM_018482 | ASAP1 | 2 | 10.457 | 1 | 18 | 1995 | 2012 | UUUUUUUUUUGUUUUGGC | : ::::::::::::::: | ACAAAAACAAAAAAAAAA | Cleavage | ArfGAP with SH3 domain, ankyrin repeat and PH domain 1 | 2 |
| -2.75 | UUUUUUUUUUGUUUUGGC | NM_018482 | ASAP1 | 2.5 | 5.514 | 1 | 18 | 1977 | 1994 | UUUUUUUUUUGUUUUGGC | .::::: :::::::::: | UUCAAAAAAAAAAAAAAA | Translation | ArfGAP with SH3 domain, ankyrin repeat and PH domain 1 | 2 |
| -2.75 | UUUUUUUUUUGUUUUGGC | NM_024669 | ANKRD55 | 2 | 15.642 | 1 | 18 | 393 | 410 | UUUUUUUUUUGUUUUGGC | :::::::::::::::: | AACAAAACAAAAAAAAAA | Cleavage | ankyrin repeat domain 55 | 1 |
| -2.75 | UUUUUUUUUUGUUUUGGC | NM_019046 | ANKRD16 | 2 | 10.284 | 1 | 18 | 804 | 821 | UUUUUUUUUUGUUUUGGC | :::::: :::::::::: | UCCAAAAAAAAAAAAAAA | Translation | ankyrin repeat domain 16 | 1 |
| -2.75 | UUUUUUUUUUGUUUUGGC | NM_015208 | ANKRD12 | 2 | 9.283 | 1 | 18 | 4299 | 4316 | UUUUUUUUUUGUUUUGGC | :::::: :::::::::: | UCCAAAAAAAAAAAAAAA | Translation | ankyrin repeat domain 12 | 2 |
| -2.75 | UUUUUUUUUUGUUUUGGC | NM_015208 | ANKRD12 | 2.5 | 9.959 | 1 | 18 | 4080 | 4097 | UUUUUUUUUUGUUUUGGC | .::::: :::::::::: | CUCAAAAAAAAAAAAAAA | Translation | ankyrin repeat domain 12 | 2 |
| -2.75 | UUUUUUUUUUGUUUUGGC | NM_016006 | ABHD5 | 2 | 9.289 | 1 | 18 | 3028 | 3045 | UUUUUUUUUUGUUUUGGC | :::::: :::::::::: | ACCAAAAAAAAAAAAAAA | Translation | abhydrolase domain containing 5, lysophosphatidic acid acyltransferase | 1 |
| -2.66 | UUGGACCAGUUCGACUACGAUA | NM_032291 | SGIP1 | 3 | 13.894 | 1 | 22 | 889 | 910 | UUGGACCAGUUCGACUACGAUA | ::::::.::::::::. :. | UUCCGUAGUUGAACUGGUUAAG | Cleavage | SH3GL interacting endocytic adaptor 1 | 1 |
| -2.66 | UUGGACCAGUUCGACUACGAUA | NM_001105543 | PLXNA4 | 3 | 19.399 | 1 | 22 | 1320 | 1341 | UUGGACCAGUUCGACUACGAUA | : : :: ::::::::::: | UUGGGAAUUCUAACUGGUCCAA | Cleavage | plexin A4 | 1 |
| -2.66 | UUGGACCAGUUCGACUACGAUA | NM_001199462 | PDCD2 | 3 | 17.077 | 1 | 22 | 1840 | 1861 | UUGGACCAGUUCGACUACGAUA | : :: : ::::::::::: | AGAGGGAGACAAACUGGUCCAA | Cleavage | programmed cell death 2 | 1 |
| -2.66 | UUGGACCAGUUCGACUACGAUA | NM_016500 | PBDC1 | 3 | 19.448 | 1 | 22 | 167 | 186 | UUGGACCAGUUCGACUACGAUA | :.::: .:::::::::.:: | CCUUGUA--UGAACUGGUCUAA | Cleavage | polysaccharide biosynthesis domain containing 1 | 1 |
| -2.66 | UUGGACCAGUUCGACUACGAUA | NM_018285 | IMP3 | 2 | 16.89 | 1 | 22 | 342 | 363 | UUGGACCAGUUCGACUACGAUA | : :.::.::.:.::::::.::: | UCUUGUGGUUGGACUGGUUCAA | Cleavage | IMP U3 small nucleolar ribonucleoprotein 3 | 1 |
| -2.66 | UUGGACCAGUUCGACUACGAUA | NM_001256487 | GOLGB1 | 3 | 21.37 | 1 | 22 | 859 | 880 | UUGGACCAGUUCGACUACGAUA | : :::: .:::::::.:: | GCCAGGAGUCUGACUGGUCUAA | Cleavage | golgin B1 | 1 |
| -2.66 | UUGGACCAGUUCGACUACGAUA | NM_004227 | CYTH3 | 3 | 24.225 | 1 | 22 | 1276 | 1297 | UUGGACCAGUUCGACUACGAUA | ::.:: ::::::::: :: | GCCGGUGGUGGAACUGGUCAAA | Cleavage | cytohesin 3 | 1 |
| -2.65 | AACUAUUAGAUUGCAAAUC | NM_017612 | ZCCHC8 | 3 | 9.499 | 1 | 19 | 1464 | 1482 | AACUAUUAGAUUGCAAAUC | :::::: : ::::::::: | GAUUUGAACCCUAAUAGUU | Translation | zinc finger CCHC-type containing 8 | 1 |
| -2.65 | AACUAUUAGAUUGCAAAUC | NM_020119 | ZC3HAV1 | 3 | 20.04 | 1 | 19 | 3993 | 4011 | AACUAUUAGAUUGCAAAUC | :::::.: :. ::::::: | UAUUUGUAUUUCAAUAGUU | Translation | zinc finger CCCH-type containing, antiviral 1 | 1 |
| -2.65 | AACUAUUAGAUUGCAAAUC | NM_004620 | TRAF6 | 3 | 16.963 | 1 | 19 | 3102 | 3120 | AACUAUUAGAUUGCAAAUC | :::::: :.:.:..::::: | GAUUUGAAGUUUGGUAGUU | Cleavage | TNF receptor associated factor 6 | 1 |
| -2.65 | AACUAUUAGAUUGCAAAUC | NM_181783 | TMTC3 | 3 | 16.908 | 1 | 19 | 3901 | 3919 | AACUAUUAGAUUGCAAAUC | :::: : ::::::::::: | GAUUAGAAAUCUAAUAGUA | Cleavage | transmembrane O-mannosyltransferase targeting cadherins 3 | 1 |
| -2.65 | AACUAUUAGAUUGCAAAUC | NM_020123 | TM9SF3 | 3 | 15.257 | 1 | 19 | 1650 | 1668 | AACUAUUAGAUUGCAAAUC | .::: .:.:.:::::::: | AGUUUCUAGUUUAAUAGUU | Cleavage | transmembrane 9 superfamily member 3 | 1 |
| -2.65 | AACUAUUAGAUUGCAAAUC | NM_001161708 | SYNC | 1.5 | 15.98 | 1 | 19 | 547 | 564 | AACUAUUAGAUUGCAAAUC | :::::: ::::::::.::: | GAUUUG-AAUCUAAUGGUU | Cleavage | syncoilin, intermediate filament protein | 1 |
| -2.65 | AACUAUUAGAUUGCAAAUC | NM_030786 | SYNC | 1.5 | 15.98 | 1 | 19 | 609 | 626 | AACUAUUAGAUUGCAAAUC | :::::: ::::::::.::: | GAUUUG-AAUCUAAUGGUU | Cleavage | syncoilin, intermediate filament protein | 1 |
| -2.65 | AACUAUUAGAUUGCAAAUC | NM_003569 | STX7 | 3 | 14.182 | 1 | 19 | 221 | 239 | AACUAUUAGAUUGCAAAUC | :.: : ::::.:::::::: | GGUCUUCAAUUUAAUAGUU | Cleavage | syntaxin 7 | 1 |
| -2.65 | AACUAUUAGAUUGCAAAUC | NM_144949 | SOCS5 | 3 | 10.172 | 1 | 19 | 762 | 780 | AACUAUUAGAUUGCAAAUC | ::: ::::.:::::: ::: | GAUGUGCAGUCUAAUUGUU | Cleavage | suppressor of cytokine signaling 5 | 1 |
| -2.65 | AACUAUUAGAUUGCAAAUC | NM_052885 | SLC2A13 | 3 | 8.45 | 1 | 19 | 210 | 227 | AACUAUUAGAUUGCAAAUC | ::::: .::::::::: :: | GAUUU-UAAUCUAAUAAUU | Cleavage | solute carrier family 2 member 13 | 1 |
| -2.65 | AACUAUUAGAUUGCAAAUC | NM_020336 | RALGAPB | 3 | 10.549 | 1 | 19 | 341 | 359 | AACUAUUAGAUUGCAAAUC | ::::::::: ::. :::: | AAUUUGCAAUAUAGAAGUU | Translation | Ral GTPase activating protein non-catalytic subunit beta | 1 |
| -2.65 | AACUAUUAGAUUGCAAAUC | NM_206855 | QKI | 3 | 12.838 | 1 | 19 | 14206 | 14224 | AACUAUUAGAUUGCAAAUC | ::: : ::::::::::: | AAUUGACCAUCUAAUAGUU | Cleavage | QKI, KH domain containing RNA binding | 1 |
| -2.65 | AACUAUUAGAUUGCAAAUC | NM_206854 | QKI | 3 | 12.838 | 1 | 19 | 13241 | 13259 | AACUAUUAGAUUGCAAAUC | ::: : ::::::::::: | AAUUGACCAUCUAAUAGUU | Cleavage | QKI, KH domain containing RNA binding | 1 |
| -2.65 | AACUAUUAGAUUGCAAAUC | NM_206853 | QKI | 3 | 12.838 | 1 | 19 | 11980 | 11998 | AACUAUUAGAUUGCAAAUC | ::: : ::::::::::: | AAUUGACCAUCUAAUAGUU | Cleavage | QKI, KH domain containing RNA binding | 1 |
| -2.65 | AACUAUUAGAUUGCAAAUC | NM_001007257 | PRDM2 | 3 | 10.096 | 1 | 19 | 980 | 998 | AACUAUUAGAUUGCAAAUC | :::::.:.:.::::: :: | AAUUUGUAGUUUAAUAUUU | Cleavage | PR/SET domain 2 | 1 |
| -2.65 | AACUAUUAGAUUGCAAAUC | NM_001102560 | PLPP5 | 3 | 17.476 | 1 | 19 | 141 | 160 | AACUAUUAGAU-UGCAAAUC | :::::::: ::.:::: ::: | GAUUUGCACAUUUAAUUGUU | Translation | phospholipid phosphatase 5 | 1 |
| -2.65 | AACUAUUAGAUUGCAAAUC | NM_002665 | PLGLB2 | 2 | 11.163 | 1 | 19 | 2615 | 2633 | AACUAUUAGAUUGCAAAUC | ::::::::::. :.::::: | GAUUUGCAAUUAAGUAGUU | Cleavage | plasminogen like B2 | 1 |
| -2.65 | AACUAUUAGAUUGCAAAUC | NM_014819 | PJA2 | 3 | 19.373 | 1 | 19 | 1397 | 1415 | AACUAUUAGAUUGCAAAUC | :::::.:::.::. :::: | AAUUUGUAAUUUAGGAGUU | Cleavage | praja ring finger ubiquitin ligase 2 | 1 |
| -2.65 | AACUAUUAGAUUGCAAAUC | NM_020403 | PCDH9 | 2 | 9.28 | 1 | 19 | 1561 | 1580 | AACUAUUAGAUU-GCAAAUC | :.::::: :::::.:::::: | GGUUUGCUAAUCUGAUAGUU | Cleavage | protocadherin 9 | 1 |
| -2.65 | AACUAUUAGAUUGCAAAUC | NM_001145373 | OTUD1 | 3 | 18.546 | 1 | 19 | 442 | 460 | AACUAUUAGAUUGCAAAUC | .::::..::.:..::::: | AGUUUGUGAUUUGGUAGUU | Cleavage | OTU deubiquitinase 1 | 1 |
| -2.65 | AACUAUUAGAUUGCAAAUC | NM_145321 | OSBPL3 | 2.5 | 14.29 | 1 | 19 | 533 | 551 | AACUAUUAGAUUGCAAAUC | ::::::: :.::.:.::: | CAUUUGCAUUUUAGUGGUU | Translation | oxysterol binding protein like 3 | 1 |
| -2.65 | AACUAUUAGAUUGCAAAUC | NM_005387 | NUP98 | 3 | 12.328 | 1 | 19 | 453 | 471 | AACUAUUAGAUUGCAAAUC | :::::.: :.::::::: | UAUUUGUAUUUUAAUAGUA | Translation | nucleoporin 98 and 96 precursor | 1 |
| -2.65 | AACUAUUAGAUUGCAAAUC | NM_182543 | NSUN6 | 3 | 13.687 | 1 | 19 | 275 | 293 | AACUAUUAGAUUGCAAAUC | :::::..:::::::. :: | CAUUUGUGAUCUAAUGUUU | Cleavage | NOP2/Sun RNA methyltransferase 6 | 1 |
| -2.65 | AACUAUUAGAUUGCAAAUC | NM_003489 | NRIP1 | 3 | 11.853 | 1 | 19 | 1197 | 1215 | AACUAUUAGAUUGCAAAUC | ::::::::: : .::::: | AAUUUGCAAUAUUGUAGUU | Translation | nuclear receptor interacting protein 1 | 1 |
| -2.65 | AACUAUUAGAUUGCAAAUC | NM_004784 | NDST3 | 2.5 | 10.084 | 1 | 19 | 1140 | 1158 | AACUAUUAGAUUGCAAAUC | :::::.::::::::. :: | UAUUUGUAAUCUAAUGUUU | Cleavage | N-deacetylase and N-sulfotransferase 3 | 1 |
| -2.65 | AACUAUUAGAUUGCAAAUC | NM_178812 | MTDH | 2 | 15.659 | 1 | 19 | 5379 | 5397 | AACUAUUAGAUUGCAAAUC | :::::: : ::::::::: | CAUUUGCCAGCUAAUAGUU | Translation | metadherin | 1 |
| -2.65 | AACUAUUAGAUUGCAAAUC | NM_001166139 | LCORL | 3 | 7.562 | 1 | 19 | 624 | 642 | AACUAUUAGAUUGCAAAUC | :::: . ::.:::::::: | UAUUUAUCAUUUAAUAGUU | Cleavage | ligand dependent nuclear receptor corepressor like | 1 |
| -2.65 | AACUAUUAGAUUGCAAAUC | NM_016027 | LACTB2 | 2.5 | 6.091 | 1 | 19 | 220 | 238 | AACUAUUAGAUUGCAAAUC | ::: .:::::::::::: | AAUUCAUAAUCUAAUAGUU | Cleavage | lactamase beta 2 | 1 |
| -2.65 | AACUAUUAGAUUGCAAAUC | NM_020768 | KCTD16 | 3 | 13.011 | 1 | 19 | 1502 | 1520 | AACUAUUAGAUUGCAAAUC | :::::. : .:::::::: | AAUUUGUCAAUUAAUAGUU | Translation | potassium channel tetramerization domain containing 16 | 1 |
| -2.65 | AACUAUUAGAUUGCAAAUC | NM_014485 | HPGDS | 3 | 13.283 | 1 | 19 | 273 | 292 | AACUAUUA-GAUUGCAAAUC | :: :::::.:: ::::.::: | GAAUUGCAGUCAUAAUGGUU | Cleavage | hematopoietic prostaglandin D synthase | 1 |
| -2.65 | AACUAUUAGAUUGCAAAUC | NM_201595 | GTF2A1 | 3 | 10.299 | 1 | 19 | 3398 | 3416 | AACUAUUAGAUUGCAAAUC | : :::::: :::::::: | UUUGUGCAAUGUAAUAGUU | Translation | general transcription factor IIA subunit 1 | 1 |
| -2.65 | AACUAUUAGAUUGCAAAUC | NM_020918 | GPAM | 2.5 | 15.034 | 1 | 19 | 660 | 679 | AACUAUUAGAUUGCAA-AUC | ::: :::.:::: ::::::: | GAUAUUGUAAUCAAAUAGUU | Cleavage | glycerol-3-phosphate acyltransferase, mitochondrial | 1 |
| -2.65 | AACUAUUAGAUUGCAAAUC | NM_020912 | FLYWCH1 | 3 | 9.92 | 1 | 19 | 4 | 22 | AACUAUUAGAUUGCAAAUC | :::::. :::::::.:: | UAUUUGUUAUCUAAUGGUG | Cleavage | FLYWCH-type zinc finger 1 | 1 |
| -2.65 | AACUAUUAGAUUGCAAAUC | NM_001126123 | ENOSF1 | 2.5 | 15.37 | 1 | 19 | 233 | 251 | AACUAUUAGAUUGCAAAUC | :::: :: :.:::::::: | CAUUUCCAUUUUAAUAGUU | Translation | enolase superfamily member 1 | 1 |
| -2.65 | AACUAUUAGAUUGCAAAUC | NM_202758 | ENOSF1 | 2.5 | 14.892 | 1 | 19 | 1317 | 1335 | AACUAUUAGAUUGCAAAUC | :::: :: :.:::::::: | CAUUUCCAUUUUAAUAGUU | Translation | enolase superfamily member 1 | 1 |
| -2.65 | AACUAUUAGAUUGCAAAUC | NM_024763 | DNAI4 | 3 | 14.818 | 1 | 19 | 168 | 186 | AACUAUUAGAUUGCAAAUC | .::: :::::::: :::: | AGUUUACAAUCUAAGAGUU | Cleavage | dynein axonemal intermediate chain 4 | 1 |
| -2.65 | AACUAUUAGAUUGCAAAUC | NM_016565 | COA4 | 2.5 | 7.832 | 1 | 19 | 329 | 347 | AACUAUUAGAUUGCAAAUC | :::: :::::::::.:: | AAUUUACAAUCUAAUGGUA | Cleavage | cytochrome c oxidase assembly factor 4 homolog | 1 |
| -2.65 | AACUAUUAGAUUGCAAAUC | NM_001099642 | CMTR2 | 3 | 16.413 | 1 | 19 | 1072 | 1091 | AACU-AUUAGAUUGCAAAUC | :::::::.: :::: :::: | UAUUUGCAGUGUAAUGAGUU | Translation | cap methyltransferase 2 | 1 |
| -2.65 | AACUAUUAGAUUGCAAAUC | NM_001201551 | CFHR4 | 3 | 12.785 | 1 | 19 | 132 | 151 | AAC-UAUUAGAUUGCAAAUC | :::::::: .::::: ::: | AAUUUGCAACUUAAUAUGUU | Translation | complement factor H related 4 | 1 |
| -2.65 | AACUAUUAGAUUGCAAAUC | NM_004642 | CDK2AP1 | 3 | 13.548 | 1 | 19 | 182 | 201 | AACUA-UUAGAUUGCAAAUC | :::::::: :.::: ::::: | GAUUUGCAUUUUAAGUAGUU | Translation | cyclin dependent kinase 2 associated protein 1 | 1 |
| -2.65 | AACUAUUAGAUUGCAAAUC | NM_032783 | CBR4 | 2.5 | 9.343 | 1 | 19 | 1986 | 2004 | AACUAUUAGAUUGCAAAUC | :::::.:::.::::: :: | AAUUUGUAAUUUAAUACUU | Cleavage | carbonyl reductase 4 | 2 |
| -2.65 | AACUAUUAGAUUGCAAAUC | NM_032783 | CBR4 | 3 | 13.775 | 1 | 19 | 1555 | 1572 | AACUAUUAGAUUGCAAAUC | :.::: :::: ::.::::: | GGUUU-CAAUGUAGUAGUU | Translation | carbonyl reductase 4 | 2 |
| -2.65 | AACUAUUAGAUUGCAAAUC | NM_033656 | BRWD1 | 3 | 15.76 | 1 | 19 | 3827 | 3845 | AACUAUUAGAUUGCAAAUC | :::::::: .::: :::: | AAUUUGCAACUUAACAGUU | Translation | bromodomain and WD repeat domain containing 1 | 1 |
| -2.65 | AACUAUUAGAUUGCAAAUC | NM_017552 | ATAD2B | 2.5 | 16.33 | 1 | 19 | 939 | 957 | AACUAUUAGAUUGCAAAUC | : : :::::.:::::::: | CAGUGGCAAUUUAAUAGUU | Cleavage | ATPase family AAA domain containing 2B | 1 |
| -2.65 | AACUAUUAGAUUGCAAAUC | NM_006380 | APPBP2 | 3 | 15.097 | 1 | 19 | 1651 | 1670 | AACUAUUA-GAUUGCAAAUC | :::::::.:: :: ::::: | UAUUUGCAGUCCUAUUAGUU | Cleavage | amyloid beta precursor protein binding protein 2 | 1 |
| -2.65 | AACUAUUAGAUUGCAAAUC | NM_001276713 | ANKDD1B | 3 | 12.002 | 1 | 19 | 761 | 779 | AACUAUUAGAUUGCAAAUC | ::::.::::::: :::: | UUUUUGUAAUCUAAGAGUU | Cleavage | ankyrin repeat and death domain containing 1B | 1 |
| -2.63 | UAUCGCUACGAGGGUGGACUGAAA | NM_001128309 | TSPAN14 | 3 | 12.388 | 1 | 24 | 1983 | 2005 | UAUCGCUACGAGGGUGGACUGAAA | :::: :::::::: .:: :::::: | UUUCUGUCCACCCCUGU-GCGAUA | Translation | tetraspanin 14 | 1 |
| -2.63 | UAUCGCUACGAGGGUGGACUGAAA | NM_007173 | PRSS23 | 3 | 11.306 | 1 | 24 | 2049 | 2072 | UAUCGCUACGAGGGUGGACUGAAA | :::::.:.:. :::.:::: | AACAUGUCCAUCUUUUUAGUGAUA | Translation | serine protease 23 | 1 |
| -2.63 | UAUCGCUACGAGGGUGGACUGAAA | NM_199420 | POLQ | 3 | 19.978 | 1 | 24 | 406 | 429 | UAUCGCUACGAGGGUGGACUGAAA | ::: . :.:::::: ::.:.:.:: | UUUGGUUUCACCCUGGUGGUGGUA | Translation | DNA polymerase theta | 1 |
| -2.63 | UAUCGCUACGAGGGUGGACUGAAA | NM_144963 | FAM91A1 | 2.5 | 14.264 | 1 | 24 | 749 | 772 | UAUCGCUACGAGGGUGGACUGAAA | :::::.::.::::: ::: | UUAUUUUCCACUCUUGUAGCUAUA | Cleavage | family with sequence similarity 91 member A1 | 1 |
| -2.63 | UAUCGCUACGAGGGUGGACUGAAA | NM_007202 | AKAP10 | 3 | 13.312 | 1 | 24 | 357 | 380 | UAUCGCUACGAGGGUGGACUGAAA | :.. : ::: :::::::.:.:: | ACUUGAUGCACACUCGUAGUGGUA | Cleavage | A-kinase anchoring protein 10 | 1 |
| -2.63 | UAUCGCUACGAGGGUGGACUGAAA | NM_001106 | ACVR2B | 2.5 | 11.901 | 1 | 24 | 9214 | 9237 | UAUCGCUACGAGGGUGGACUGAAA | :.::::::.::::: ::: | UCACUUUUCACCCUUGUAGCUAUA | Cleavage | activin A receptor type 2B | 1 |
| -2.63 | GGCCCGUCGCAGACGUUUCCUG | NM_004395 | DBN1 | 3 | 12.292 | 1 | 22 | 765 | 786 | GGCCCGUCGCAGACGUUUCCUG | :::: ::::. :::::.: | GCACAAACUUCUGUAACGGGUC | Cleavage | drebrin 1 | 1 |
| -2.63 | GGCCCGUCGCAGACGUUUCCUG | NM_001130087 | ABLIM2 | 2.5 | 23.114 | 1 | 22 | 524 | 545 | GGCCCGUCGCAGACGUUUCCUG | : :..::::::.::.:::.: | UUGCAGGCGUCUGUGAUGGGUC | Cleavage | actin binding LIM protein family member 2 | 1 |
| -2.62 | AACAGGAAGACAAUUGAGACAAA | NM_032433 | ZNF333 | 3 | 16.015 | 1 | 23 | 352 | 374 | AACAGGAAGACAAUUGAGACAAA | : : :::.:.::: :::..:::: | UAUUUCUUAGUUGCCUUUUUGUU | Translation | zinc finger protein 333 | 1 |
| -2.62 | AACAGGAAGACAAUUGAGACAAA | NM_001243256 | ZIC4 | 2.5 | 18.881 | 1 | 23 | 1244 | 1266 | AACAGGAAGACAAUUGAGACAAA | :: ::.::::::::: .:::: | GUUCCCUUAAUUGUCUUGUUGUU | Cleavage | Zic family member 4 | 1 |
| -2.62 | AAAGAUGAAAAGAACCUUGA | NM_001127399 | YPEL5 | 3 | 11.75 | 1 | 20 | 784 | 803 | AAAGAUGAAAAGAACCUUGA | : ::: ::.::::::::: : | UAAAGUUUUUUUUCAUCUCU | Cleavage | yippee like 5 | 1 |
| -2.62 | AAAGAUGAAAAGAACCUUGA | NM_032312 | YIPF4 | 2.5 | 10.386 | 1 | 20 | 724 | 743 | AAAGAUGAAAAGAACCUUGA | : ::: :::: ::.:::::: | UAAAGUUUCUGUUUAUCUUU | Translation | Yip1 domain family member 4 | 1 |
| -2.62 | AAAGAUGAAAAGAACCUUGA | NM_139281 | WDR36 | 3 | 11.951 | 1 | 20 | 1011 | 1030 | AAAGAUGAAAAGAACCUUGA | ::.::::.::::.::.:: | CCAGGGUUUUUUUUAUUUUG | Cleavage | WD repeat domain 36 | 1 |
| -2.62 | AACAGGAAGACAAUUGAGACAAA | NM_006646 | WASF3 | 3 | 11.862 | 1 | 23 | 2166 | 2188 | AACAGGAAGACAAUUGAGACAAA | : :.:.:::::::::::: | UAAUUGUUAGUUGUCUUCCUGUA | Cleavage | WASP family member 3 | 1 |
| -2.62 | AAAGAUGAAAAGAACCUUGA | NM_017667 | VPS50 | 3 | 9.972 | 1 | 20 | 1966 | 1985 | AAAGAUGAAAAGAACCUUGA | ::::::.::::. :.::: | AAAAGGUUUUUUUUUUUUUU | Cleavage | VPS50 subunit of EARP/GARPII complex | 1 |
| -2.62 | AAAGAUGAAAAGAACCUUGA | NM_017684 | VPS13C | 2 | 13.73 | 1 | 20 | 371 | 389 | AAAGAUGAAAAGAACCUUGA | :::::: ::::.::.::: | GAAAGGUU-UUUUUAUUUUU | Cleavage | vacuolar protein sorting 13 homolog C | 1 |
| -2.62 | AAAGAUGAAAAGAACCUUGA | NM_007146 | VEZF1 | 3 | 18.478 | 1 | 20 | 1432 | 1451 | AAAGAUGAAAAGAACCUUGA | ::::.:::::::.::: | UUUUGGUUUUUUUCAUUUUU | Cleavage | vascular endothelial zinc finger 1 | 1 |
| -2.62 | AACAGGAAGACAAUUGAGACAAA | NM_007124 | UTRN | 3 | 15.88 | 1 | 23 | 1063 | 1085 | AACAGGAAGACAAUUGAGACAAA | :: .:.::::.:::.:::: | AAACUCCUAGUUGUUUUCUUGUU | Cleavage | utrophin | 1 |
| -2.62 | AAAGAUGAAAAGAACCUUGA | NM_020245 | TULP4 | 3 | 10.805 | 1 | 20 | 2179 | 2198 | AAAGAUGAAAAGAACCUUGA | ::::::.::::. :.::: | AGAAGGUUUUUUUUUUUUUU | Cleavage | TUB like protein 4 | 1 |
| -2.62 | AAAGAUGAAAAGAACCUUGA | NM_001007466 | TULP4 | 3 | 10.805 | 1 | 20 | 2273 | 2292 | AAAGAUGAAAAGAACCUUGA | ::::::.::::. :.::: | AGAAGGUUUUUUUUUUUUUU | Cleavage | TUB like protein 4 | 1 |
| -2.62 | AAAGAUGAAAAGAACCUUGA | NM_021643 | TRIB2 | 3 | 20.482 | 1 | 20 | 1725 | 1744 | AAAGAUGAAAAGAACCUUGA | ::::::.::::. :.::: | AGAAGGUUUUUUUUUUUUUU | Cleavage | tribbles pseudokinase 2 | 1 |
| -2.62 | AAAGAUGAAAAGAACCUUGA | NM_198485 | TPRG1 | 3 | 12.52 | 1 | 20 | 1216 | 1235 | AAAGAUGAAAAGAACCUUGA | ::::: :::::::: :.::: | UCAAGUUUCUUUUCUUUUUU | Cleavage | tumor protein p63 regulated 1 | 1 |
| -2.62 | AAAGAUGAAAAGAACCUUGA | NM_001190943 | TNFSF10 | 3 | 12.124 | 1 | 20 | 871 | 890 | AAAGAUGAAAAGAACCUUGA | :.::::.::::. ::::: | GGAGGGUUUUUUUUUUCUUU | Cleavage | TNF superfamily member 10 | 1 |
| -2.62 | AAAGAUGAAAAGAACCUUGA | NM_003268 | TLR5 | 3 | 17.648 | 1 | 20 | 87 | 106 | AAAGAUGAAAAGAACCUUGA | : .:::::.::::. ::::: | UGGAGGUUUUUUUUUUCUUU | Cleavage | toll like receptor 5 | 1 |
| -2.62 | AAAGAUGAAAAGAACCUUGA | NM_022566 | TLNRD1 | 3 | 17.872 | 1 | 20 | 325 | 345 | AAAGAUGAAAAGAACCU-UGA | ::: .::::: ::::::.::: | UCAUGGGUUCAUUUCAUUUUU | Translation | talin rod domain containing 1 | 1 |
| -2.62 | AAAGAUGAAAAGAACCUUGA | NM_007005 | TLE4 | 3 | 16.771 | 1 | 20 | 521 | 540 | AAAGAUGAAAAGAACCUUGA | :::::.::::..:.::: | AUUAGGUUUUUUUUGUUUUU | Cleavage | TLE family member 4, transcriptional corepressor | 1 |
| -2.62 | AAAGAUGAAAAGAACCUUGA | NM_005994 | TBX2 | 3 | 8.836 | 1 | 20 | 710 | 729 | AAAGAUGAAAAGAACCUUGA | :.:: :::.:::::::.:: | UUAAAGUUUUUUUCAUUUUA | Cleavage | T-box transcription factor 2 | 1 |
| -2.62 | AAAGAUGAAAAGAACCUUGA | NM_005843 | STAM2 | 3 | 14.889 | 1 | 20 | 153 | 172 | AAAGAUGAAAAGAACCUUGA | ::::::.::::. :.::: | AAAAGGUUUUUUUUUUUUUU | Cleavage | signal transducing adaptor molecule 2 | 1 |
| -2.62 | AACAGGAAGACAAUUGAGACAAA | NM_001012968 | SPIN4 | 3 | 9.946 | 1 | 23 | 2486 | 2508 | AACAGGAAGACAAUUGAGACAAA | :.:::::: :.::::::: | GAGUUUUCAAUUUUUUUCCUGUG | Translation | spindlin family member 4 | 1 |
| -2.62 | AAAGAUGAAAAGAACCUUGA | NM_001146210 | SPDYE6 | 2.5 | 13.834 | 1 | 20 | 631 | 650 | AAAGAUGAAAAGAACCUUGA | .:: ::::::::::.::: | AUAAUUUUCUUUUCAUUUUU | Cleavage | speedy/RINGO cell cycle regulator family member E6 | 1 |
| -2.62 | AAAGAUGAAAAGAACCUUGA | NM_001004351 | SPDYE3 | 2.5 | 12.249 | 1 | 20 | 630 | 649 | AAAGAUGAAAAGAACCUUGA | .:: ::::::::::.::: | AUAAUUUUCUUUUCAUUUUU | Cleavage | speedy/RINGO cell cycle regulator family member E3 | 1 |
| -2.62 | AAAGAUGAAAAGAACCUUGA | NM_175064 | SPDYE1 | 2.5 | 10.563 | 1 | 20 | 638 | 657 | AAAGAUGAAAAGAACCUUGA | .:: ::::::::::.::: | AUAAUUUUCUUUUCAUUUUU | Cleavage | speedy/RINGO cell cycle regulator family member E1 | 1 |
| -2.62 | AAAGAUGAAAAGAACCUUGA | NM_014752 | SPCS2 | 2 | 18.014 | 1 | 20 | 754 | 773 | AAAGAUGAAAAGAACCUUGA | :::::.:::::::.::: | UAUAGGUUUUUUUCAUUUUU | Cleavage | signal peptidase complex subunit 2 | 1 |
| -2.62 | AAAGAUGAAAAGAACCUUGA | NM_138473 | SP1 | 3 | 12.422 | 1 | 20 | 2929 | 2948 | AAAGAUGAAAAGAACCUUGA | : : :::.:::::::.::: | CCUAAGUUUUUUUCAUUUUU | Cleavage | Sp1 transcription factor | 1 |
| -2.62 | AACAGGAAGACAAUUGAGACAAA | NM_052870 | SNX18 | 1.5 | 14.639 | 1 | 23 | 833 | 855 | AACAGGAAGACAAUUGAGACAAA | ::..::::::::.::::: | CCUCCCUUGAUUGUCUUUCUGUU | Cleavage | sorting nexin 18 | 1 |
| -2.62 | AACAGGAAGACAAUUGAGACAAA | NM_144682 | SLFN13 | 3 | 12.547 | 1 | 23 | 3680 | 3702 | AACAGGAAGACAAUUGAGACAAA | : :: ::::: ::::::. :::: | UGUGCCUCAAGUGUCUUUAUGUU | Cleavage | schlafen family member 13 | 1 |
| -2.62 | AAAGAUGAAAAGAACCUUGA | NM_001258379 | SLC4A7 | 3 | 16.138 | 1 | 20 | 3674 | 3693 | AAAGAUGAAAAGAACCUUGA | ::::::::.::::. :.::: | UCAAGGUUUUUUUUUUUUUU | Cleavage | solute carrier family 4 member 7 | 1 |
| -2.62 | AAAGAUGAAAAGAACCUUGA | NM_017515 | SLC35F2 | 3 | 16.819 | 1 | 20 | 427 | 446 | AAAGAUGAAAAGAACCUUGA | ::::::::.::::. :.::: | UCAAGGUUUUUUUUUUUUUU | Cleavage | solute carrier family 35 member F2 | 1 |
| -2.62 | AACAGGAAGACAAUUGAGACAAA | NM_017515 | SLC35F2 | 2.5 | 9.802 | 1 | 23 | 611 | 633 | AACAGGAAGACAAUUGAGACAAA | :: : :::::::.::..:::: | CUUCCCACAAUUGUUUUUUUGUU | Cleavage | solute carrier family 35 member F2 | 1 |
| -2.62 | AAAGAUGAAAAGAACCUUGA | NM_004733 | SLC33A1 | 2.5 | 13.475 | 1 | 20 | 1227 | 1246 | AAAGAUGAAAAGAACCUUGA | .:::::::::: ::: ::: | AUAAGGUUCUUUGCAUGUUU | Cleavage | solute carrier family 33 member 1 | 1 |
| -2.62 | AAAGAUGAAAAGAACCUUGA | NM_001102396 | SIKE1 | 3 | 10.107 | 1 | 20 | 954 | 974 | AAAGAU-GAAAAGAACCUUGA | ::.::::.::::. :::::: | GCAGGGUUUUUUUUUAUCUUU | Cleavage | suppressor of IKBKE 1 | 1 |
| -2.62 | AAAGAUGAAAAGAACCUUGA | NM_170709 | SGK3 | 3 | 13.138 | 1 | 20 | 1092 | 1111 | AAAGAUGAAAAGAACCUUGA | :. : ::::: ::::::::: | UUUAUGUUCUAUUCAUCUUU | Translation | serum/glucocorticoid regulated kinase family member 3 | 1 |
| -2.62 | AAAGAUGAAAAGAACCUUGA | NM_032861 | SERAC1 | 2.5 | 15.807 | 1 | 20 | 1615 | 1634 | AAAGAUGAAAAGAACCUUGA | :: ::::::: :::::.::: | UCUAGGUUCUGUUCAUUUUU | Translation | serine active site containing 1 | 1 |
| -2.62 | AAAGAUGAAAAGAACCUUGA | NM_004404 | SEPTIN2 | 3 | 18.032 | 1 | 20 | 1517 | 1536 | AAAGAUGAAAAGAACCUUGA | ::: :.:::::::.::: | GACAGGGUUUUUUCAUUUUU | Cleavage | septin 2 | 1 |
| -2.62 | AAAGAUGAAAAGAACCUUGA | NM_000997 | RPL37 | 3 | 17.975 | 1 | 20 | 817 | 836 | AAAGAUGAAAAGAACCUUGA | : ..::::.::::..:.::: | UGGGGGUUUUUUUUGUUUUU | Cleavage | ribosomal protein L37 | 1 |
| -2.62 | AAAGAUGAAAAGAACCUUGA | NM_207396 | RNF207 | 2.5 | 13.006 | 1 | 20 | 77 | 96 | AAAGAUGAAAAGAACCUUGA | : : ::::.::::.::.::: | UGAUGGUUUUUUUUAUUUUU | Cleavage | ring finger protein 207 | 1 |
| -2.62 | AAAGAUGAAAAGAACCUUGA | NM_001085400 | RELL1 | 3 | 15.973 | 1 | 20 | 685 | 704 | AAAGAUGAAAAGAACCUUGA | ::::::.::::. :.::: | AGAAGGUUUUUUUUUUUUUU | Cleavage | RELT like 1 | 1 |
| -2.62 | AAAGAUGAAAAGAACCUUGA | NM_172071 | RC3H1 | 3 | 12.839 | 1 | 20 | 1183 | 1202 | AAAGAUGAAAAGAACCUUGA | .::::::.::::. :.::: | GUAAGGUUUUUUUUUUUUUU | Cleavage | ring finger and CCCH-type domains 1 | 1 |
| -2.62 | AACAGGAAGACAAUUGAGACAAA | NM_001198844 | RBM4 | 3 | 8.236 | 1 | 23 | 2347 | 2369 | AACAGGAAGACAAUUGAGACAAA | : :::::: :::.::.:::: | GAUCUCUCAAAUGUUUUUCUGUA | Cleavage | RNA binding motif protein 4 | 1 |
| -2.62 | AAAGAUGAAAAGAACCUUGA | NM_170672 | RASGRP3 | 2 | 14.971 | 1 | 20 | 439 | 458 | AAAGAUGAAAAGAACCUUGA | ..::::.:::::::.::: | GAGGGGUUUUUUUCAUUUUU | Cleavage | RAS guanyl releasing protein 3 | 1 |
| -2.62 | AAAGAUGAAAAGAACCUUGA | NM_213589 | RAPH1 | 3 | 18.18 | 1 | 20 | 4047 | 4066 | AAAGAUGAAAAGAACCUUGA | :.::::::.::::. :.::: | UUAAGGUUUUUUUUUUUUUU | Cleavage | Ras association (RalGDS/AF-6) and pleckstrin homology domains 1 | 1 |
| -2.62 | AAAGAUGAAAAGAACCUUGA | NM_001099666 | PTAR1 | 2 | 19.815 | 1 | 20 | 6752 | 6771 | AAAGAUGAAAAGAACCUUGA | :. ::::::::::.::.::: | UUUAGGUUCUUUUUAUUUUU | Cleavage | protein prenyltransferase alpha subunit repeat containing 1 | 1 |
| -2.62 | AACAGGAAGACAAUUGAGACAAA | NM_001173489 | PRRG1 | 2.5 | 13.691 | 1 | 23 | 3253 | 3275 | AACAGGAAGACAAUUGAGACAAA | : ::::.: :::::::::: | ACAGGCUCAGUGGUCUUCCUGUA | Cleavage | proline rich and Gla domain 1 | 1 |
| -2.62 | AAAGAUGAAAAGAACCUUGA | NM_003913 | PRP4K | 3 | 19.509 | 1 | 20 | 2140 | 2159 | AAAGAUGAAAAGAACCUUGA | :::::: :::::::::: | ACAAGGUGAUUUUCAUCUUC | Cleavage | pre-mRNA processing factor kinase PRP4K | 1 |
| -2.62 | AAAGAUGAAAAGAACCUUGA | NM_002737 | PRKCA | 2.5 | 20.995 | 1 | 20 | 4259 | 4278 | AAAGAUGAAAAGAACCUUGA | :.::::.::::..:.::: | GGAGGGUUUUUUUUGUUUUU | Cleavage | protein kinase C alpha | 1 |
| -2.62 | AAAGAUGAAAAGAACCUUGA | NM_003620 | PPM1D | 3 | 23.002 | 1 | 20 | 2630 | 2649 | AAAGAUGAAAAGAACCUUGA | ::: ::: :::::::::: | ACAAUGUUAUUUUCAUCUUC | Cleavage | protein phosphatase, Mg2+/Mn2+ dependent 1D | 1 |
| -2.62 | AAAGAUGAAAAGAACCUUGA | NM_001080475 | PLEKHM3 | 3 | 15.41 | 1 | 20 | 5982 | 6001 | AAAGAUGAAAAGAACCUUGA | ::::: :.:: :.:::::: | ACAAGGAUUUUCUUAUCUUU | Translation | pleckstrin homology domain containing M3 | 1 |
| -2.62 | AACAGGAAGACAAUUGAGACAAA | NM_032177 | PHAX | 3 | 14.193 | 1 | 23 | 107 | 129 | AACAGGAAGACAAUUGAGACAAA | :::::::.:::.:::: | GGAAAGACAAUUGUUUUCUUGUU | Cleavage | phosphorylated adaptor for RNA export | 1 |
| -2.62 | AAAGAUGAAAAGAACCUUGA | NM_199203 | PEDS1-UBE2V1 | 3 | 9.278 | 1 | 20 | 1400 | 1419 | AAAGAUGAAAAGAACCUUGA | : ::::::.::::. :.::: | UAAAGGUUUUUUUUUUUUUU | Cleavage | PEDS1-UBE2V1 readthrough | 1 |
| -2.62 | AAAGAUGAAAAGAACCUUGA | NM_148977 | PANK1 | 3 | 10.044 | 1 | 20 | 1052 | 1071 | AAAGAUGAAAAGAACCUUGA | :.:: : :.:::::::.::: | UUAAAGGUUUUUUCAUUUUU | Cleavage | pantothenate kinase 1 | 1 |
| -2.62 | AAAGAUGAAAAGAACCUUGA | NM_006703 | NUDT3 | 2 | 12.281 | 1 | 20 | 551 | 569 | AAAGAUGAAAAGAACCUUGA | ::::::::.::::: ::::: | UCAAGGUUUUUUUC-UCUUU | Cleavage | nudix hydrolase 3 | 1 |
| -2.62 | AAAGAUGAAAAGAACCUUGA | NM_173159 | NPAS3 | 2.5 | 9.628 | 1 | 20 | 2943 | 2963 | AAAG-AUGAAAAGAACCUUGA | ::.:::::::::.:: :::: | ACAGGGUUCUUUUUAUACUUU | Cleavage | neuronal PAS domain protein 3 | 1 |
| -2.62 | AAAGAUGAAAAGAACCUUGA | NM_001037675 | NBPF9 | 3 | 17.65 | 1 | 20 | 334 | 353 | AAAGAUGAAAAGAACCUUGA | ::::::: :: :::::::: | UCAAGGUCAUUGUCAUCUUU | Translation | NBPF member 9 | 1 |
| -2.62 | AAAGAUGAAAAGAACCUUGA | NM_001277444 | NBPF9 | 3 | 18.123 | 1 | 20 | 334 | 353 | AAAGAUGAAAAGAACCUUGA | ::::::: :: :::::::: | UCAAGGUCAUUGUCAUCUUU | Translation | NBPF member 9 | 1 |
| -2.62 | AAAGAUGAAAAGAACCUUGA | NM_001102663 | NBPF15 | 3 | 18.123 | 1 | 20 | 334 | 353 | AAAGAUGAAAAGAACCUUGA | ::::::: :: :::::::: | UCAAGGUCAUUGUCAUCUUU | Translation | NBPF member 15 | 1 |
| -2.62 | AAAGAUGAAAAGAACCUUGA | NM_173638 | NBPF15 | 3 | 18.123 | 1 | 20 | 334 | 353 | AAAGAUGAAAAGAACCUUGA | ::::::: :: :::::::: | UCAAGGUCAUUGUCAUCUUU | Translation | NBPF member 15 | 1 |
| -2.62 | AAAGAUGAAAAGAACCUUGA | NM_015383 | NBPF14 | 3 | 17.3 | 1 | 20 | 334 | 353 | AAAGAUGAAAAGAACCUUGA | ::::::: :: :::::::: | UCAAGGUCAUUGUCAUCUUU | Translation | NBPF member 14 | 1 |
| -2.62 | AAAGAUGAAAAGAACCUUGA | NM_057175 | NAA15 | 2.5 | 12.363 | 1 | 20 | 2528 | 2547 | AAAGAUGAAAAGAACCUUGA | .:: ::::::::..:.::: | GUAAAGUUCUUUUUGUUUUU | Cleavage | N-alpha-acetyltransferase 15, NatA auxiliary subunit | 1 |
| -2.62 | AAAGAUGAAAAGAACCUUGA | NM_198468 | MMS22L | 2 | 13.887 | 1 | 20 | 3172 | 3191 | AAAGAUGAAAAGAACCUUGA | :.:.: :::::::.:::::: | UUAGGCUUCUUUUUAUCUUU | Cleavage | MMS22 like, DNA repair protein | 1 |
| -2.62 | AAAGAUGAAAAGAACCUUGA | NM_002338 | LSAMP | 2.5 | 16.763 | 1 | 20 | 7693 | 7710 | AAAGAUGAAAAGAACCUUGA | :::::::: ::::::.::: | UCAAGGUU--UUUCAUUUUU | Translation | limbic system associated membrane protein | 1 |
| -2.62 | AAAGAUGAAAAGAACCUUGA | NM_015350 | LRRC8B | 2 | 3.845 | 1 | 20 | 3568 | 3587 | AAAGAUGAAAAGAACCUUGA | :: :::::::::::::: | AAAAUUUUCUUUUCAUCUUU | Cleavage | leucine rich repeat containing 8 VRAC subunit B | 1 |
| -2.62 | AACAGGAAGACAAUUGAGACAAA | NM_015350 | LRRC8B | 3 | 6.368 | 1 | 23 | 674 | 696 | AACAGGAAGACAAUUGAGACAAA | ::::.:.:: :: ::::::::: | CUUGUUUUAACUGACUUCCUGUU | Translation | leucine rich repeat containing 8 VRAC subunit B | 1 |
| -2.62 | AAAGAUGAAAAGAACCUUGA | NM_022896 | LPIN3 | 3 | 15.813 | 1 | 20 | 249 | 268 | AAAGAUGAAAAGAACCUUGA | ::.:::::::::. :.::: | GCAGGGUUCUUUUUUUUUUU | Cleavage | lipin 3 | 1 |
| -2.62 | AAAGAUGAAAAGAACCUUGA | NM_017635 | KMT5B | 3 | 16.328 | 1 | 20 | 1343 | 1362 | AAAGAUGAAAAGAACCUUGA | : :::::::::::::.: : | ACCAGGUUCUUUUCAUUUCU | Cleavage | lysine methyltransferase 5B | 1 |
| -2.62 | AACAGGAAGACAAUUGAGACAAA | NM_020805 | KLHL14 | 2.5 | 13.911 | 1 | 23 | 954 | 976 | AACAGGAAGACAAUUGAGACAAA | :. ::.:::::::.::::: | AGAAUUCCAGUUGUCUUUCUGUU | Cleavage | kelch like family member 14 | 1 |
| -2.62 | AACAGGAAGACAAUUGAGACAAA | NM_138444 | KCTD12 | 3 | 13.287 | 1 | 23 | 3973 | 3996 | AACAGGAAGACAAUU-GAGACAAA | :: ::.: ::::::.:::::::: | UUCGUUUGGAAUUGUUUUCCUGUU | Cleavage | potassium channel tetramerization domain containing 12 | 1 |
| -2.62 | AACAGGAAGACAAUUGAGACAAA | NM_000214 | JAG1 | 3 | 14.721 | 1 | 23 | 479 | 501 | AACAGGAAGACAAUUGAGACAAA | ::: :.:.:::::: ::..:::: | UUUUUUUUAAUUGUAUUUUUGUU | Translation | jagged canonical Notch ligand 1 | 1 |
| -2.62 | AAAGAUGAAAAGAACCUUGA | NM_002214 | ITGB8 | 3 | 13.149 | 1 | 20 | 3743 | 3762 | AAAGAUGAAAAGAACCUUGA | :..:: ::::::::::.:: | UUGAGCUUCUUUUCAUUUUG | Cleavage | integrin subunit beta 8 | 1 |
| -2.62 | AAAGAUGAAAAGAACCUUGA | NM_005544 | IRS1 | 3 | 11.151 | 1 | 20 | 4414 | 4433 | AAAGAUGAAAAGAACCUUGA | .:.::::.::::. ::::: | GUAGGGUUUUUUUUUUCUUU | Cleavage | insulin receptor substrate 1 | 1 |
| -2.62 | AAAGAUGAAAAGAACCUUGA | NM_002163 | IRF8 | 2 | 16.676 | 1 | 20 | 393 | 412 | AAAGAUGAAAAGAACCUUGA | ::::::: ::::..::::: | ACAAGGUUGUUUUUGUCUUU | Cleavage | interferon regulatory factor 8 | 1 |
| -2.62 | AAAGAUGAAAAGAACCUUGA | NM_004136 | IREB2 | 3 | 17.421 | 1 | 20 | 2179 | 2198 | AAAGAUGAAAAGAACCUUGA | :::::::::: :: ::.::: | UCAAGGUUCUCUUAAUUUUU | Translation | iron responsive element binding protein 2 | 2 |
| -2.62 | AAAGAUGAAAAGAACCUUGA | NM_004136 | IREB2 | 3 | 14.061 | 1 | 20 | 2495 | 2515 | AAAG-AUGAAAAGAACCUUGA | ::.::::.::::.:: :::: | GCAGGGUUUUUUUUAUACUUU | Cleavage | iron responsive element binding protein 2 | 2 |
| -2.62 | AACAGGAAGACAAUUGAGACAAA | NM_004506 | HSF2 | 3 | 12.778 | 1 | 23 | 313 | 335 | AACAGGAAGACAAUUGAGACAAA | :.:.. ::::.:::::::: | AAAUUUUUGUUUGUUUUCCUGUU | Cleavage | heat shock transcription factor 2 | 1 |
| -2.62 | AACAGGAAGACAAUUGAGACAAA | NM_032410 | HOOK3 | 2 | 12.703 | 1 | 23 | 9194 | 9216 | AACAGGAAGACAAUUGAGACAAA | .:: :::::::::.:::: | UUAACUUCCAUUGUCUUCUUGUU | Cleavage | hook microtubule tethering protein 3 | 1 |
| -2.62 | AAAGAUGAAAAGAACCUUGA | NM_001113239 | HIPK2 | 2.5 | 22.277 | 1 | 20 | 2856 | 2875 | AAAGAUGAAAAGAACCUUGA | :: :::::::::::.:: | AAAAUGUUCUUUUCAUUUUA | Cleavage | homeodomain interacting protein kinase 2 | 1 |
| -2.62 | AAAGAUGAAAAGAACCUUGA | NM_001267051 | GPR155 | 3 | 17.638 | 1 | 20 | 1128 | 1147 | AAAGAUGAAAAGAACCUUGA | .::::::::::. :.::: | AAGAGGUUCUUUUUCUUUUU | Cleavage | G protein-coupled receptor 155 | 1 |
| -2.62 | AACAGGAAGACAAUUGAGACAAA | NM_001130009 | GEN1 | 2.5 | 9.403 | 1 | 23 | 1414 | 1436 | AACAGGAAGACAAUUGAGACAAA | : ::::::::: ::.:::: | UCAGCCUCAAUUGUAUUUCUGUA | Translation | GEN1 Holliday junction 5' flap endonuclease | 1 |
| -2.62 | AACAGGAAGACAAUUGAGACAAA | NM_001129908 | GASK1A | 3 | 11.175 | 1 | 23 | 1197 | 1219 | AACAGGAAGACAAUUGAGACAAA | :: ::: ::::::::..:::: | CUUUUCUGCAUUGUCUUUUUGUU | Cleavage | golgi associated kinase 1A | 1 |
| -2.62 | AAAGAUGAAAAGAACCUUGA | NM_000813 | GABRB2 | 1.5 | 13.084 | 1 | 20 | 2846 | 2866 | AAAGAUGAAAAGA-ACCUUGA | :.::::: :.::::::::::: | UUAAGGUGUUUUUUCAUCUUU | Cleavage | gamma-aminobutyric acid type A receptor subunit beta2 | 1 |
| -2.62 | AAAGAUGAAAAGAACCUUGA | NM_032664 | FUT10 | 2.5 | 17.75 | 1 | 20 | 1464 | 1483 | AAAGAUGAAAAGAACCUUGA | ::::::.::::: :.::: | GGAAGGUUUUUUUCUUUUUU | Cleavage | fucosyltransferase 10 | 1 |
| -2.62 | AAAGAUGAAAAGAACCUUGA | NM_001039885 | FKRP | 3 | 13.765 | 1 | 20 | 1557 | 1576 | AAAGAUGAAAAGAACCUUGA | .:.::::.::::.:: ::: | AUAGGGUUUUUUUUAUGUUU | Cleavage | fukutin related protein | 1 |
| -2.62 | AAAGAUGAAAAGAACCUUGA | NM_000508 | FGA | 1 | 11.638 | 1 | 20 | 181 | 200 | AAAGAUGAAAAGAACCUUGA | .::::::.::::.:::::: | GUAAGGUUUUUUUUAUCUUU | Cleavage | fibrinogen alpha chain | 1 |
| -2.62 | AACAGGAAGACAAUUGAGACAAA | NM_001077498 | FAM222B | 3 | 16.273 | 1 | 23 | 293 | 315 | AACAGGAAGACAAUUGAGACAAA | :: :::..: : :::::::::: | CUUUUCUUGAAUCUCUUCCUGUU | Translation | family with sequence similarity 222 member B | 1 |
| -2.62 | AAAGAUGAAAAGAACCUUGA | NM_207446 | FAM174B | 2.5 | 11.47 | 1 | 20 | 1267 | 1286 | AAAGAUGAAAAGAACCUUGA | ::::: .::::.::.::: | GAAAGGUGUUUUUUAUUUUU | Cleavage | family with sequence similarity 174 member B | 1 |
| -2.62 | AAAGAUGAAAAGAACCUUGA | NM_001142521 | FAM111A | 2.5 | 6.455 | 1 | 20 | 593 | 613 | AAAGAUGAAAAGAACCU-UGA | ::: ::: :.::::::::::: | UCAUAGGGUUUUUUCAUCUUU | Cleavage | FAM111 trypsin like peptidase A | 1 |
| -2.62 | AAAGAUGAAAAGAACCUUGA | NM_003633 | ENC1 | 3 | 21.957 | 1 | 20 | 2291 | 2310 | AAAGAUGAAAAGAACCUUGA | ::::::::: :.::.:: | AGAAGGUUCUUCUUAUUUUG | Translation | ectodermal-neural cortex 1 | 1 |
| -2.62 | AAAGAUGAAAAGAACCUUGA | NM_015433 | EEF1AKMT3 | 2 | 14.655 | 1 | 20 | 994 | 1013 | AAAGAUGAAAAGAACCUUGA | : : ::::::::::::::: | ACUAAGUUCUUUUCAUCUUU | Cleavage | EEF1A lysine methyltransferase 3 | 1 |
| -2.62 | AAAGAUGAAAAGAACCUUGA | NM_206914 | EEF1AKMT3 | 2 | 14.655 | 1 | 20 | 1364 | 1383 | AAAGAUGAAAAGAACCUUGA | : : ::::::::::::::: | ACUAAGUUCUUUUCAUCUUU | Cleavage | EEF1A lysine methyltransferase 3 | 1 |
| -2.62 | AACAGGAAGACAAUUGAGACAAA | NM_080664 | DTD2 | 2 | 10.12 | 1 | 23 | 273 | 295 | AACAGGAAGACAAUUGAGACAAA | :::.::::::.::..:::: | AUGUUCUUAAUUGUUUUUUUGUU | Cleavage | D-aminoacyl-tRNA deacylase 2 | 1 |
| -2.62 | AAAGAUGAAAAGAACCUUGA | NM_017741 | DCAF16 | 2.5 | 11.283 | 1 | 20 | 814 | 833 | AAAGAUGAAAAGAACCUUGA | :.:.::::: :::.::.::: | UUAGGGUUCCUUUUAUUUUU | Translation | DDB1 and CUL4 associated factor 16 | 1 |
| -2.62 | AAAGAUGAAAAGAACCUUGA | NM_001918 | DBT | 3 | 23.495 | 1 | 20 | 3447 | 3466 | AAAGAUGAAAAGAACCUUGA | :::::: :::::::::. :: | UCAAGGCUCUUUUCAUUAUU | Cleavage | dihydrolipoamide branched chain transacylase E2 | 1 |
| -2.62 | AAAGAUGAAAAGAACCUUGA | NM_021149 | COTL1 | 3 | 17.949 | 1 | 20 | 1223 | 1242 | AAAGAUGAAAAGAACCUUGA | : :: :::.::::..:.::: | UAAAUGUUUUUUUUGUUUUU | Cleavage | coactosin like F-actin binding protein 1 | 1 |
| -2.62 | AACAGGAAGACAAUUGAGACAAA | NM_001185056 | CLDN11 | 3 | 13.851 | 1 | 23 | 1810 | 1832 | AACAGGAAGACAAUUGAGACAAA | :.:.: ::::.::.::::: | UGAUUUUUAUUUGUUUUUCUGUU | Cleavage | claudin 11 | 1 |
| -2.62 | AAAGAUGAAAAGAACCUUGA | NM_152389 | CFAP65 | 3 | 18.624 | 1 | 20 | 210 | 229 | AAAGAUGAAAAGAACCUUGA | : ::::::.::::. :.::: | UGAAGGUUUUUUUUUUUUUU | Cleavage | cilia and flagella associated protein 65 | 1 |
| -2.62 | AAAGAUGAAAAGAACCUUGA | NM_001172640 | CELF1 | 2.5 | 11.094 | 1 | 20 | 155 | 174 | AAAGAUGAAAAGAACCUUGA | ..:::::.::::..:.::: | CUGAGGUUUUUUUUGUUUUU | Cleavage | CUGBP Elav-like family member 1 | 1 |
| -2.62 | AAAGAUGAAAAGAACCUUGA | NM_004642 | CDK2AP1 | 3 | 11.954 | 1 | 20 | 210 | 229 | AAAGAUGAAAAGAACCUUGA | : :::::.::::..:.::: | CCCAGGUUUUUUUUGUUUUU | Cleavage | cyclin dependent kinase 2 associated protein 1 | 1 |
| -2.62 | AAAGAUGAAAAGAACCUUGA | NM_001038707 | CDC42SE1 | 3 | 8.952 | 1 | 20 | 1915 | 1934 | AAAGAUGAAAAGAACCUUGA | ..::::::::::. :.::: | CUGAGGUUCUUUUUUUUUUU | Cleavage | CDC42 small effector 1 | 1 |
| -2.62 | AACAGGAAGACAAUUGAGACAAA | NM_006136 | CAPZA2 | 2.5 | 13.362 | 1 | 23 | 203 | 225 | AACAGGAAGACAAUUGAGACAAA | ::: .:.:: ::::::.::::: | UUUAGUUUAAAUGUCUUUCUGUU | Cleavage | capping actin protein of muscle Z-line subunit alpha 2 | 1 |
| -2.62 | AAAGAUGAAAAGAACCUUGA | NM_001170794 | BACH2 | 3 | 12.56 | 1 | 20 | 4256 | 4275 | AAAGAUGAAAAGAACCUUGA | :::::: .::::..:.::: | ACAAGGUGUUUUUUGUUUUU | Cleavage | BTB domain and CNC homolog 2 | 1 |
| -2.62 | AAAGAUGAAAAGAACCUUGA | NM_001198665 | ARHGEF12 | 2.5 | 7.542 | 1 | 20 | 2338 | 2357 | AAAGAUGAAAAGAACCUUGA | : :: ::::::::.::::: | UGAAUGUUCUUUUUAUCUUA | Cleavage | Rho guanine nucleotide exchange factor 12 | 1 |
| -2.62 | AACAGGAAGACAAUUGAGACAAA | NM_006380 | APPBP2 | 2.5 | 9.721 | 1 | 23 | 1209 | 1231 | AACAGGAAGACAAUUGAGACAAA | ::: :: : :::::::.::::: | UUUCACUAACUUGUCUUUCUGUU | Cleavage | amyloid beta precursor protein binding protein 2 | 1 |
| -2.62 | AAAGAUGAAAAGAACCUUGA | NM_001166050 | APBB2 | 3 | 13.524 | 1 | 20 | 2976 | 2995 | AAAGAUGAAAAGAACCUUGA | :.:: :::: :::.::.::: | UUAAAGUUCCUUUUAUUUUU | Translation | amyloid beta precursor protein binding family B member 2 | 1 |
| -2.62 | AACAGGAAGACAAUUGAGACAAA | NM_001277333 | ANKRD62 | 3 | 12.558 | 1 | 23 | 748 | 770 | AACAGGAAGACAAUUGAGACAAA | : : :.:.:::: :.::.::::: | UCUAUUUUAAUUAUUUUUCUGUU | Translation | ankyrin repeat domain 62 | 1 |
| -2.62 | AAAGAUGAAAAGAACCUUGA | NM_015305 | ANGEL1 | 3 | 16.879 | 1 | 20 | 1539 | 1558 | AAAGAUGAAAAGAACCUUGA | :.:::::::::: : :.::: | UUAAGGUUCUUUCCUUUUUU | Cleavage | angel homolog 1 | 1 |
| -2.62 | AAAGAUGAAAAGAACCUUGA | NM_130847 | AMOTL1 | 3 | 15.188 | 1 | 20 | 5418 | 5438 | AAAGAUGAAAAGA-ACCUUGA | ::::::: :.::::..:.::: | UCAAGGUGUUUUUUUGUUUUU | Cleavage | angiomotin like 1 | 1 |
| -2.62 | AAAGAUGAAAAGAACCUUGA | NM_020686 | ABAT | 3 | 6.973 | 1 | 20 | 1727 | 1746 | AAAGAUGAAAAGAACCUUGA | :::::::.: ::::.::: | GCAAGGUUUUCCUCAUUUUU | Translation | 4-aminobutyrate aminotransferase | 1 |
| -2.62 | UAGGGCUCAAAACCGAUGACU | NM_145044 | ZNF501 | 3 | 15.996 | 1 | 21 | 814 | 834 | UAGGGCUCAAAACCGAUGACU | .::. ::::::::..::: | CUGUAUUUGUUUUGAGUUCUA | Cleavage | zinc finger protein 501 | 1 |
| -2.62 | UAGGGCUCAAAACCGAUGACU | NM_015144 | ZCCHC14 | 2.5 | 11.627 | 1 | 21 | 827 | 847 | UAGGGCUCAAAACCGAUGACU | ::::: .:: ::::::::::: | AGUCAGUGGCUUUGAGCCCUA | Cleavage | zinc finger CCHC-type containing 14 | 1 |
| -2.62 | UAGGGCUCAAAACCGAUGACU | NM_017662 | TRPM6 | 3 | 16.91 | 1 | 21 | 1406 | 1427 | UAGGGCUCA-AAACCGAUGACU | :::::.::::: :::: :::: | UGUCAUUGGUUUGUGAGGCCUA | Translation | transient receptor potential cation channel subfamily M member 6 | 1 |
| -2.62 | UAGGGCUCAAAACCGAUGACU | NM_001127496 | SPRY4 | 3 | 21.786 | 1 | 21 | 1331 | 1351 | UAGGGCUCAAAACCGAUGACU | .: :: .:: ::::::::::. | GGCCACUGGGUUUGAGCCCUG | Cleavage | sprouty RTK signaling antagonist 4 | 1 |
| -2.62 | UAGGGCUCAAAACCGAUGACU | NM_001004308 | RTL4 | 3 | 12.177 | 1 | 21 | 1040 | 1060 | UAGGGCUCAAAACCGAUGACU | : ::::.: : ::::::.::: | ACUCAUUGCUAUUGAGCUCUA | Translation | retrotransposon Gag like 4 | 1 |
| -2.62 | UAGGGCUCAAAACCGAUGACU | NM_004279 | PMPCB | 3 | 12.258 | 1 | 21 | 1896 | 1916 | UAGGGCUCAAAACCGAUGACU | .::.::::: ::::::: | UUGUAUUGGUUUAAAGCCCUA | Translation | peptidase, mitochondrial processing subunit beta | 1 |
| -2.62 | UAGGGCUCAAAACCGAUGACU | NM_001543 | NDST1 | 3 | 21.062 | 1 | 21 | 3601 | 3622 | UAGGG-CUCAAAACCGAUGACU | : :::.: ::::::: ::::: | UGACAUUGUUUUUGAGGCCCUA | Cleavage | N-deacetylase and N-sulfotransferase 1 | 1 |
| -2.62 | UAGGGCUCAAAACCGAUGACU | NM_001195522 | MPC1L | 3 | 15.017 | 1 | 21 | 77 | 96 | UAGGGCUCAAAACCGAUGACU | :: : :::::::::: :.::: | AGACCUCGGUUUUGA-CUCUA | Cleavage | mitochondrial pyruvate carrier 1 like | 1 |
| -2.62 | UAGGGCUCAAAACCGAUGACU | NM_030624 | KLHL15 | 3 | 16.657 | 1 | 21 | 2387 | 2407 | UAGGGCUCAAAACCGAUGACU | :: ::: :::::::::..:: | UGUAAUCAGUUUUGAGCUUUA | Cleavage | kelch like family member 15 | 1 |
| -2.62 | UAGGGCUCAAAACCGAUGACU | NM_173529 | C18orf54 | 3 | 14.768 | 1 | 21 | 154 | 174 | UAGGGCUCAAAACCGAUGACU | ::: :::::::.: :::: | ACCCAUAGGUUUUGGGACCUA | Cleavage | chromosome 18 open reading frame 54 | 1 |
| -2.32 | CAGGAUCCCUUUUCGGAGG | NM_016399 | TRIAP1 | 2.5 | 18.803 | 1 | 19 | 433 | 451 | CAGGAUCCCUUUUCGGAGG | :::.:::::::::::. : | GCUCUGAAAAGGGAUCUGG | Cleavage | TP53 regulated inhibitor of apoptosis 1 | 1 |
| -2.32 | CAGGAUCCCUUUUCGGAGG | NM_007375 | TARDBP | 2 | 11.629 | 1 | 19 | 2359 | 2377 | CAGGAUCCCUUUUCGGAGG | ::::.: :::::::::.:: | CCUCUGUAAAGGGAUCUUG | Cleavage | TAR DNA binding protein | 1 |
| -2.32 | CAGGAUCCCUUUUCGGAGG | NM_014980 | STXBP5L | 3 | 14.667 | 1 | 19 | 1084 | 1102 | CAGGAUCCCUUUUCGGAGG | ..:. :.::::::::.::: | UUUUGGGAAAGGGAUUCUG | Cleavage | syntaxin binding protein 5L | 1 |
| -2.32 | CAGGAUCCCUUUUCGGAGG | NM_024104 | SMIM7 | 3 | 19.306 | 1 | 19 | 838 | 856 | CAGGAUCCCUUUUCGGAGG | .::..::::.:: :::::: | UCUUUGAAAGGGAAUCCUG | Cleavage | small integral membrane protein 7 | 1 |
| -2.32 | CAGGAUCCCUUUUCGGAGG | NM_134262 | RORA | 2.5 | 13.106 | 1 | 19 | 2586 | 2604 | CAGGAUCCCUUUUCGGAGG | ..::: :.:::::::.::: | UUUCCCAGAAGGGAUUCUG | Cleavage | RAR related orphan receptor A | 1 |
| -2.32 | CAGGAUCCCUUUUCGGAGG | NM_022841 | RFX7 | 3 | 13.299 | 1 | 19 | 1790 | 1809 | CAGGAUC-CCUUUUCGGAGG | :::.::::::: :::..:: | ACUCUGAAAAGGAGAUUUUG | Cleavage | regulatory factor X7 | 1 |
| -2.32 | CAGGAUCCCUUUUCGGAGG | NM_021183 | RAP2C | 3 | 16.472 | 1 | 19 | 829 | 847 | CAGGAUCCCUUUUCGGAGG | :::..:::::::::: .:: | CCUUUGAAAAGGGAUAUUG | Cleavage | RAP2C, member of RAS oncogene family | 1 |
| -2.32 | CAGGAUCCCUUUUCGGAGG | NM_018061 | PRPF38B | 3 | 17.735 | 1 | 19 | 426 | 444 | CAGGAUCCCUUUUCGGAGG | ::..::.:: :::::.:: | ACUUUGAGAAUGGAUCUUG | Translation | pre-mRNA processing factor 38B | 1 |
| -2.32 | CAGGAUCCCUUUUCGGAGG | NM_176877 | PATJ | 3 | 17.066 | 1 | 19 | 2507 | 2525 | CAGGAUCCCUUUUCGGAGG | .:..:::: :::::.::: | GUUUUGAAAUGGGAUUCUG | Translation | PATJ crumbs cell polarity complex component | 1 |
| -2.32 | CAGGAUCCCUUUUCGGAGG | NM_020772 | NUFIP2 | 3 | 19.139 | 1 | 19 | 5411 | 5429 | CAGGAUCCCUUUUCGGAGG | :::.::::: :::: ::: | GCUCUGAAAAUGGAUACUG | Translation | nuclear FMR1 interacting protein 2 | 1 |
| -2.32 | CAGGAUCCCUUUUCGGAGG | NM_001184691 | NPNT | 2.5 | 15.28 | 1 | 19 | 1011 | 1029 | CAGGAUCCCUUUUCGGAGG | .:: ::::: :::::::: | AUUCAGAAAAAGGAUCCUG | Translation | nephronectin | 1 |
| -2.32 | CAGGAUCCCUUUUCGGAGG | NM_002500 | NEUROD1 | 3 | 17.589 | 1 | 19 | 1340 | 1358 | CAGGAUCCCUUUUCGGAGG | .::.: ::::::::.::: | UCUUCUUAAAGGGAUUCUG | Cleavage | neuronal differentiation 1 | 1 |
| -2.32 | CAGGAUCCCUUUUCGGAGG | NM_001012418 | MYLK4 | 2.5 | 15.979 | 1 | 19 | 2440 | 2458 | CAGGAUCCCUUUUCGGAGG | ::..::::: :::::.:: | ACUUUGAAAAUGGAUCUUG | Translation | myosin light chain kinase family member 4 | 1 |
| -2.32 | CAGGAUCCCUUUUCGGAGG | NM_006037 | HDAC4 | 2.5 | 16.048 | 1 | 19 | 1312 | 1330 | CAGGAUCCCUUUUCGGAGG | :.: .:.:::::::::::: | CUUAUGGAAAGGGAUCCUG | Cleavage | histone deacetylase 4 | 1 |
| -2.32 | CAGGAUCCCUUUUCGGAGG | NM_005477 | HCN4 | 3 | 23.113 | 1 | 19 | 1904 | 1922 | CAGGAUCCCUUUUCGGAGG | ::::: :.: ::::::.:: | CCUCCCAGAUGGGAUCUUG | Translation | hyperpolarization activated cyclic nucleotide gated potassium channel 4 | 1 |
| -2.32 | CAGGAUCCCUUUUCGGAGG | NM_001079871 | HAP1 | 3 | 24.206 | 1 | 19 | 1266 | 1284 | CAGGAUCCCUUUUCGGAGG | ::::.:::: :::: :::: | CCUCUGAAAUGGGACCCUG | Translation | huntingtin associated protein 1 | 1 |
| -2.32 | CAGGAUCCCUUUUCGGAGG | NM_015597 | GPSM1 | 3 | 22.785 | 1 | 19 | 520 | 539 | CAGG-AUCCCUUUUCGGAGG | :: ::::.::::::: :::: | CCCCCGAGAAGGGAUGCCUG | Cleavage | G protein signaling modulator 1 | 1 |
| -2.32 | CAGGAUCCCUUUUCGGAGG | NM_000821 | GGCX | 3 | 11.944 | 1 | 19 | 4728 | 4746 | CAGGAUCCCUUUUCGGAGG | ::::: :::: ::::::: | CCUCCAAAAAUAGAUCCUG | Translation | gamma-glutamyl carboxylase | 1 |
| -2.32 | CAGGAUCCCUUUUCGGAGG | NM_177996 | EPB41L1 | 3 | 19.411 | 1 | 19 | 1 | 19 | CAGGAUCCCUUUUCGGAGG | ::::.: .::: ::::::: | CCUCUGUGAAGAGAUCCUG | Cleavage | erythrocyte membrane protein band 4.1 like 1 | 1 |
| -2.32 | CAGGAUCCCUUUUCGGAGG | NM_001252507 | C14orf132 | 3 | 18.54 | 1 | 19 | 3275 | 3293 | CAGGAUCCCUUUUCGGAGG | :::: ::.:: ::.::::: | CCUCAGAGAAAGGGUCCUG | Translation | chromosome 14 open reading frame 132 | 1 |
| -2.32 | CAGGAUCCCUUUUCGGAGG | NM_032199 | ARID5B | 3 | 14.227 | 1 | 19 | 2634 | 2652 | CAGGAUCCCUUUUCGGAGG | :::.::: :: ::::.:: | ACUCUGAACAGAGAUCUUG | Translation | AT-rich interaction domain 5B | 1 |
| -2.32 | CAGGAUCCCUUUUCGGAGG | NM_203382 | AMACR | 3 | 19.137 | 1 | 19 | 257 | 275 | CAGGAUCCCUUUUCGGAGG | .::.. :::::::::::: | UCUUUCAAAAGGGAUCCUU | Cleavage | alpha-methylacyl-CoA racemase | 1 |
| -2.32 | CAGGAUCCCUUUUCGGAGG | NM_014914 | AGAP1 | 2 | 21.802 | 1 | 19 | 5649 | 5667 | CAGGAUCCCUUUUCGGAGG | .::.:.::::::::.::: | AUUCUGGAAAGGGAUUCUG | Cleavage | ArfGAP with GTPase domain, ankyrin repeat and PH domain 1 | 1 |
| -2.28 | GCGAGGUCGAGCUGGAUCGC | NM_001008925 | RCHY1 | 3 | 13.085 | 1 | 20 | 1295 | 1313 | GCGAGGUCGAGCUGGAUCGC | .:::::::: ::::.:.: | AUGAUCCAGC-CGACUUUGA | Translation | ring finger and CHY zinc finger domain containing 1 | 1 |
| -2.28 | GCGAGGUCGAGCUGGAUCGC | NM_015436 | RCHY1 | 3 | 13.085 | 1 | 20 | 1114 | 1132 | GCGAGGUCGAGCUGGAUCGC | .:::::::: ::::.:.: | AUGAUCCAGC-CGACUUUGA | Translation | ring finger and CHY zinc finger domain containing 1 | 1 |
| -2.28 | GCGAGGUCGAGCUGGAUCGC | NM_005374 | MPP2 | 2.5 | 10.363 | 1 | 20 | 1809 | 1828 | GCGAGGUCGAGCUGGAUCGC | .:::.:::::: .:::.:: | CUGAUUCAGCUCUGCCUUGC | Cleavage | MAGUK p55 scaffold protein 2 | 1 |
| -2.28 | GCGAGGUCGAGCUGGAUCGC | NM_020853 | FAM234B | 3 | 19.686 | 1 | 20 | 1873 | 1892 | GCGAGGUCGAGCUGGAUCGC | :.:::::::.:::.:.: | UGGGUCCAGCUUGACUUUGG | Cleavage | family with sequence similarity 234 member B | 1 |
| -2.28 | GCGAGGUCGAGCUGGAUCGC | NM_003792 | EDF1 | 3 | 19.506 | 1 | 20 | 22 | 40 | GCGAGGUCGAGCUGGAUCGC | ::: ::::::: ::.::::. | GCGCUCCAGCU-GAUCUCGU | Translation | endothelial differentiation related factor 1 | 1 |
| -2.28 | GCGAGGUCGAGCUGGAUCGC | NM_153500 | CAMKK2 | 2 | 21.031 | 1 | 20 | 104 | 121 | GCGAGGUCGAGCUGGAUCGC | :::: :::::::::::: | UGGAUC--GCUCGACCUCGC | Cleavage | calcium/calmodulin dependent protein kinase kinase 2 | 1 |
| -2.28 | GCGAGGUCGAGCUGGAUCGC | NM_001497 | B4GALT1 | 3 | 14.176 | 1 | 20 | 755 | 774 | GCGAGGUCGAGCUGGAUCGC | .::::.::::.::..:.:. | AUGAUCUAGCUUGAUUUUGU | Cleavage | beta-1,4-galactosyltransferase 1 | 1 |
| -2.28 | GCGAGGUCGAGCUGGAUCGC | NM_000051 | ATM | 3 | 22.856 | 1 | 20 | 1699 | 1716 | GCGAGGUCGAGCUGGAUCGC | ::::: :::.:::::::. | UCGAUC--GCUUGACCUCGU | Cleavage | ATM serine/threonine kinase | 1 |
| -2.14 | AGACAUUGUCGACAGGACA | NM_017672 | TRPM7 | 2.5 | 15.359 | 1 | 19 | 1495 | 1511 | AGACAUUGUCGACAGGACA | :::::::: :::::::.: | UGUCCUGU--ACAAUGUUU | Translation | transient receptor potential cation channel subfamily M member 7 | 1 |
| -2.14 | AGACAUUGUCGACAGGACA | NM_144569 | SPOCD1 | 3 | 14.187 | 1 | 19 | 119 | 138 | AGACAUUGUC-GACAGGACA | ::::::. ::::::::.: | CUUCCUGUUUGACAAUGUUU | Translation | SPOC domain containing 1 | 1 |
| -2.14 | AGACAUUGUCGACAGGACA | NM_138324 | PCSK6 | 3 | 11.484 | 1 | 19 | 345 | 363 | AGACAUUGUCGACAGGACA | : ::: :: ::::::::: | GGCCCUUUCAACAAUGUCU | Translation | proprotein convertase subtilisin/kexin type 6 | 1 |
| -2.14 | AGACAUUGUCGACAGGACA | NM_138323 | PCSK6 | 3 | 11.484 | 1 | 19 | 386 | 404 | AGACAUUGUCGACAGGACA | : ::: :: ::::::::: | GGCCCUUUCAACAAUGUCU | Translation | proprotein convertase subtilisin/kexin type 6 | 1 |
| -2.14 | AGACAUUGUCGACAGGACA | NM_014586 | HUNK | 3 | 13.048 | 1 | 19 | 3726 | 3744 | AGACAUUGUCGACAGGACA | ::..:: .::::::::.: | AGUUUUGAUGACAAUGUUU | Cleavage | hormonally up-regulated Neu-associated kinase | 1 |
| -2.14 | AGACAUUGUCGACAGGACA | NM_018200 | HMG20A | 3 | 23.236 | 1 | 19 | 801 | 819 | AGACAUUGUCGACAGGACA | .::::.:::::::::: | CCAUCUGUUGACAAUGUCU | Cleavage | high mobility group 20A | 1 |
| -2.14 | AGACAUUGUCGACAGGACA | NM_033083 | EAF1 | 3 | 12.323 | 1 | 19 | 2765 | 2781 | AGACAUUGUCGACAGGACA | ::: :::: ::::::::: | UGUGCUGU--ACAAUGUCU | Translation | ELL associated factor 1 | 1 |
| -2.14 | AGACAUUGUCGACAGGACA | NM_033225 | CSMD1 | 3 | 15.222 | 1 | 19 | 618 | 637 | AGACAUUGU-CGACAGGACA | :::.:::::: : :::::.: | UGUUCUGUCGCAGAAUGUUU | Translation | CUB and Sushi multiple domains 1 | 1 |
| -2.14 | AGACAUUGUCGACAGGACA | NM_023077 | COA7 | 3 | 16.605 | 1 | 19 | 206 | 222 | AGACAUUGUCGACAGGACA | :::.:::: :::::::.: | UGUUCUGU--ACAAUGUUU | Translation | cytochrome c oxidase assembly factor 7 | 1 |
| -2.14 | AGACAUUGUCGACAGGACA | NM_024590 | ARSJ | 3 | 11.885 | 1 | 19 | 1932 | 1950 | AGACAUUGUCGACAGGACA | :::..::::::::.: ::: | UGUUUUGUCGACAGUAUCU | Cleavage | arylsulfatase family member J | 1 |
| -2.14 | AGACAUUGUCGACAGGACA | NM_007038 | ADAMTS5 | 3 | 16.235 | 1 | 19 | 5860 | 5878 | AGACAUUGUCGACAGGACA | :::.:::. : ::::::: | GGUCUUGUUUAAAAUGUCU | Translation | ADAM metallopeptidase with thrombospondin type 1 motif 5 | 1 |
| -2.14 | AGACAUUGUCGACAGGACA | NM_001025091 | ABCF1 | 3 | 17.761 | 1 | 19 | 730 | 748 | AGACAUUGUCGACAGGACA | :::.:: .::::.:::.: | AGUCUUGCUGACAGUGUUU | Cleavage | ATP binding cassette subfamily F member 1 | 1 |
| -2.13 | UUCGAAUCUCUUAGCAAC | NM_144969 | ZDHHC15 | 2.5 | 17.308 | 1 | 18 | 3819 | 3836 | UUCGAAUCUCUUAGCAAC | :: :::::::::::::. | GUAGCUAAGAGAUUCGGU | Cleavage | zinc finger DHHC-type palmitoyltransferase 15 | 1 |
| -2.13 | UUCGAAUCUCUUAGCAAC | NM_001039693 | TYW5 | 3 | 11.053 | 1 | 18 | 1917 | 1934 | UUCGAAUCUCUUAGCAAC | :::.:.:::::::.::. | UUUGUUGAGAGAUUUGAG | Cleavage | tRNA-yW synthesizing protein 5 | 1 |
| -2.13 | UUCGAAUCUCUUAGCAAC | NM_031216 | SEH1L | 3 | 19.27 | 1 | 18 | 1274 | 1291 | UUCGAAUCUCUUAGCAAC | ::: ::.::::::: ::: | GUUUCUGAGAGAUUAGAA | Cleavage | SEH1 like nucleoporin | 1 |
| -2.13 | UUCGAAUCUCUUAGCAAC | NM_130831 | OPA1 | 2.5 | 12.766 | 1 | 18 | 2754 | 2771 | UUCGAAUCUCUUAGCAAC | ::::.:.::: :::.::: | GUUGUUGAGAUAUUUGAA | Cleavage | OPA1 mitochondrial dynamin like GTPase | 1 |
| -2.13 | UUCGAAUCUCUUAGCAAC | NM_013393 | MRM2 | 3 | 22.244 | 1 | 18 | 74 | 91 | UUCGAAUCUCUUAGCAAC | ::: ::..::::::.::. | GUUCCUGGGAGAUUUGAG | Cleavage | mitochondrial rRNA methyltransferase 2 | 1 |
| -2.13 | UUCGAAUCUCUUAGCAAC | NM_144578 | MAPK1IP1L | 2 | 12.422 | 1 | 18 | 2112 | 2129 | UUCGAAUCUCUUAGCAAC | :::::::: .::::.::: | GUUGCUAAAGGAUUUGAA | Translation | mitogen-activated protein kinase 1 interacting protein 1 like | 1 |
| -2.13 | UUCGAAUCUCUUAGCAAC | NM_007357 | COG2 | 3 | 18.753 | 1 | 18 | 219 | 236 | UUCGAAUCUCUUAGCAAC | ::: .::::: :::.::: | GUUUUUAAGACAUUUGAA | Cleavage | component of oligomeric golgi complex 2 | 1 |
| -2.13 | GUUGGAAUCCGCUAAGGA | NM_030625 | TET1 | 3 | 14.595 | 1 | 18 | 325 | 342 | GUUGGAAUCCGCUAAGGA | :..:::: :::::..::: | UUUUUAGAGGAUUUUAAC | Translation | tet methylcytosine dioxygenase 1 | 1 |
| -2.13 | GUUGGAAUCCGCUAAGGA | NM_005835 | SLC17A2 | 3 | 13.559 | 1 | 18 | 202 | 219 | GUUGGAAUCCGCUAAGGA | ::::::: :: :::.:.: | UCCUUAGAGGUUUCUAGC | Translation | solute carrier family 17 member 2 | 1 |
| -2.13 | GUUGGAAUCCGCUAAGGA | NM_001243131 | RPL13 | 3 | 22.114 | 1 | 18 | 1690 | 1707 | GUUGGAAUCCGCUAAGGA | :.. :.:.:::::::::: | UUUCUGGUGGAUUCCAAC | Cleavage | ribosomal protein L13 | 1 |
| -2.13 | GUUGGAAUCCGCUAAGGA | NM_001003792 | RBMS3 | 2.5 | 17.196 | 1 | 18 | 3913 | 3930 | GUUGGAAUCCGCUAAGGA | :::::.:. ::::.:::: | UCCUUGGUAGAUUUCAAC | Translation | RNA binding motif single stranded interacting protein 3 | 1 |
| -2.13 | GUUGGAAUCCGCUAAGGA | NM_206907 | PRKAA1 | 2.5 | 21.096 | 1 | 18 | 2211 | 2228 | GUUGGAAUCCGCUAAGGA | ::.:::: ::::::.::. | UCUUUAGGGGAUUCUAAU | Translation | protein kinase AMP-activated catalytic subunit alpha 1 | 1 |
| -2.13 | GUUGGAAUCCGCUAAGGA | NM_002035 | KDSR | 3 | 22.256 | 1 | 18 | 1324 | 1341 | GUUGGAAUCCGCUAAGGA | :: ::::.:::::...:: | UCAUUAGUGGAUUUUGAC | Cleavage | 3-ketodihydrosphingosine reductase | 1 |
| -2.13 | GUUGGAAUCCGCUAAGGA | NM_001134651 | EIF4E3 | 3 | 10.927 | 1 | 18 | 1604 | 1620 | GUUGGAAUCCGCUAAGGA | :::::::.: :::.::: | UCCUUAGUG-AUUUCAAA | Translation | eukaryotic translation initiation factor 4E family member 3 | 1 |
| -2.13 | GUUGGAAUCCGCUAAGGA | NM_144607 | CYB5D1 | 3 | 12.646 | 1 | 18 | 2219 | 2236 | GUUGGAAUCCGCUAAGGA | ::.: ::.:: ::::::: | UCUUCAGUGGCUUCCAAC | Cleavage | cytochrome b5 domain containing 1 | 1 |
| -2.11 | CGUCUACGGAUGUGUAAUACUCGC | NM_001159293 | ZNF737 | 2.5 | 15.467 | 1 | 24 | 4260 | 4283 | CGUCUACGGAUGUGUAAUACUCGC | .:::: ::::::::::: | AAUUAGGUUACUCAUCCGUAGACU | Cleavage | zinc finger protein 737 | 1 |
| -2.11 | CGUCUACGGAUGUGUAAUACUCGC | NM_001202476 | UBE2E1 | 3 | 16.457 | 1 | 24 | 849 | 872 | CGUCUACGGAUGUGUAAUACUCGC | ::::::: :.:::::.: | CUGUACUUUACACAGCUGUAGAUG | Translation | ubiquitin conjugating enzyme E2 E1 | 1 |
| -2.11 | CGUCUACGGAUGUGUAAUACUCGC | NM_022444 | SLC13A1 | 2.5 | 14.227 | 1 | 24 | 1166 | 1188 | CGUCUACGGAUGUGUAAUACUCGC | :.:: :::::::::. :::::: | GUGACAAUUACACAUU-GUAGACA | Cleavage | solute carrier family 13 member 1 | 1 |
| -2.11 | CGUCUACGGAUGUGUAAUACUCGC | NM_015559 | SETBP1 | 3 | 10.344 | 1 | 24 | 1519 | 1542 | CGUCUACGGAUGUGUAAUACUCGC | :: ::: ::. ::::::::.: | UGAAGAAUUCCAUUUCCGUAGAUG | Translation | SET binding protein 1 | 1 |
| -2.11 | CGUCUACGGAUGUGUAAUACUCGC | NM_213589 | RAPH1 | 2 | 17.156 | 1 | 24 | 979 | 1002 | CGUCUACGGAUGUGUAAUACUCGC | :::::::.:::.:::::: | GGUGUUAUUACAUAUCUGUAGACA | Cleavage | Ras association (RalGDS/AF-6) and pleckstrin homology domains 1 | 1 |
| -2.11 | CGUCUACGGAUGUGUAAUACUCGC | NM_001197080 | IFRD1 | 3 | 18.703 | 1 | 24 | 1258 | 1281 | CGUCUACGGAUGUGUAAUACUCGC | ::: :: :::::::::: | CAUGCCAUUUCAAAUCCGUAGACU | Cleavage | interferon related developmental regulator 1 | 1 |
| -2.11 | CGUCUACGGAUGUGUAAUACUCGC | NM_014333 | CADM1 | 3 | 20.809 | 1 | 24 | 770 | 793 | CGUCUACGGAUGUGUAAUACUCGC | ::: ...:::.::::::: | UCUUUAAUUCUGUAUCUGUAGACG | Cleavage | cell adhesion molecule 1 | 1 |
| -2.01 | AAGCAGAAUACGGUAAGC | NM_015035 | ZHX3 | 3 | 21.588 | 1 | 18 | 727 | 745 | AAGCAGAA-UACGGUAAGC | :::::::: :::::::: | UGUUACCGUAAUUCUGCUU | Cleavage | zinc fingers and homeoboxes 3 | 1 |
[truncated: 215,343 more chars]
