# Supplementary figures and images for: Impact of Culture Duration on the Properties and Functionality of Yeast-Derived Extracellular Vesicles
Source: Biomater Res. 2025 May 6;29:0201. doi: 10.34133/bmr.0201 (PMC12053258; doi:10.34133/bmr.0201)

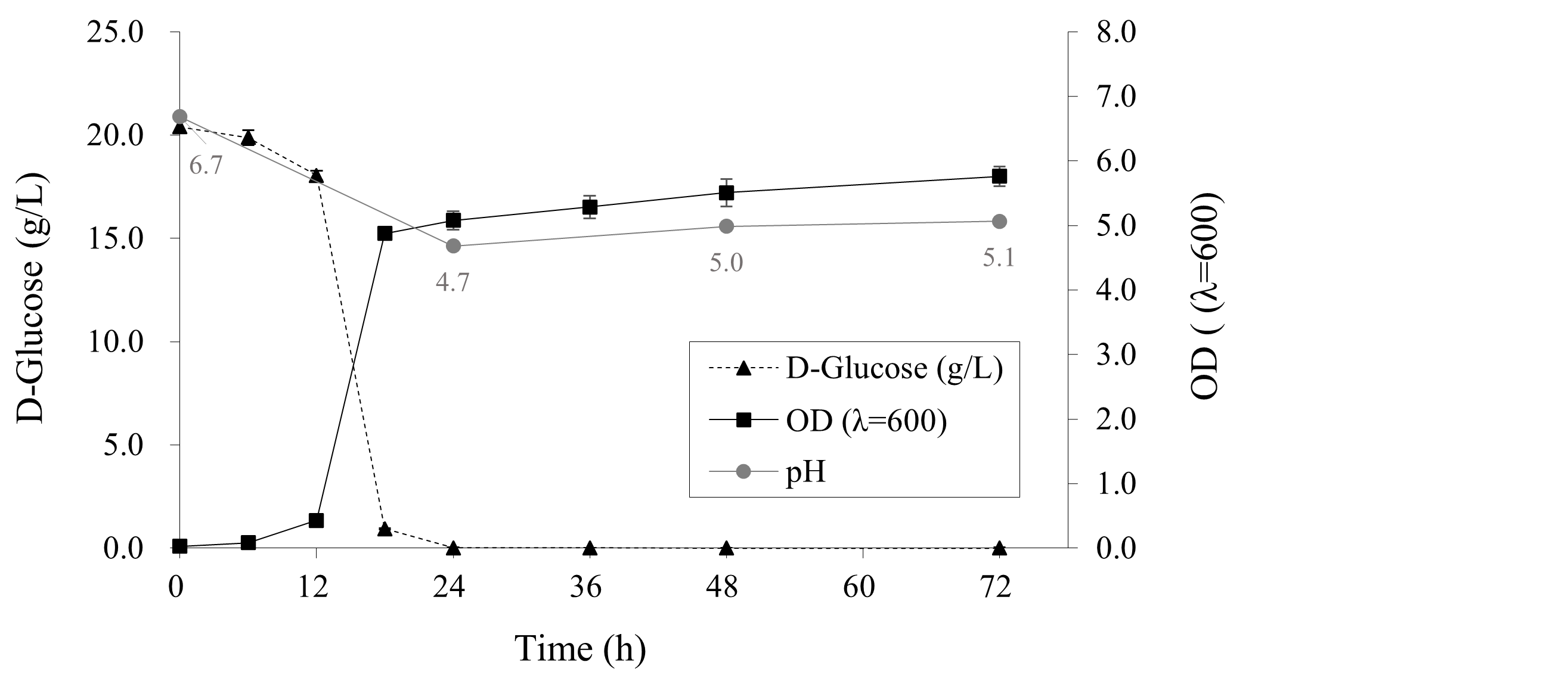

Supplement: Supplementary 1 — Figs. S1 to S4 Tables S1 to S4 [file bmr.0201.f1.zip › Figure S1.tif]

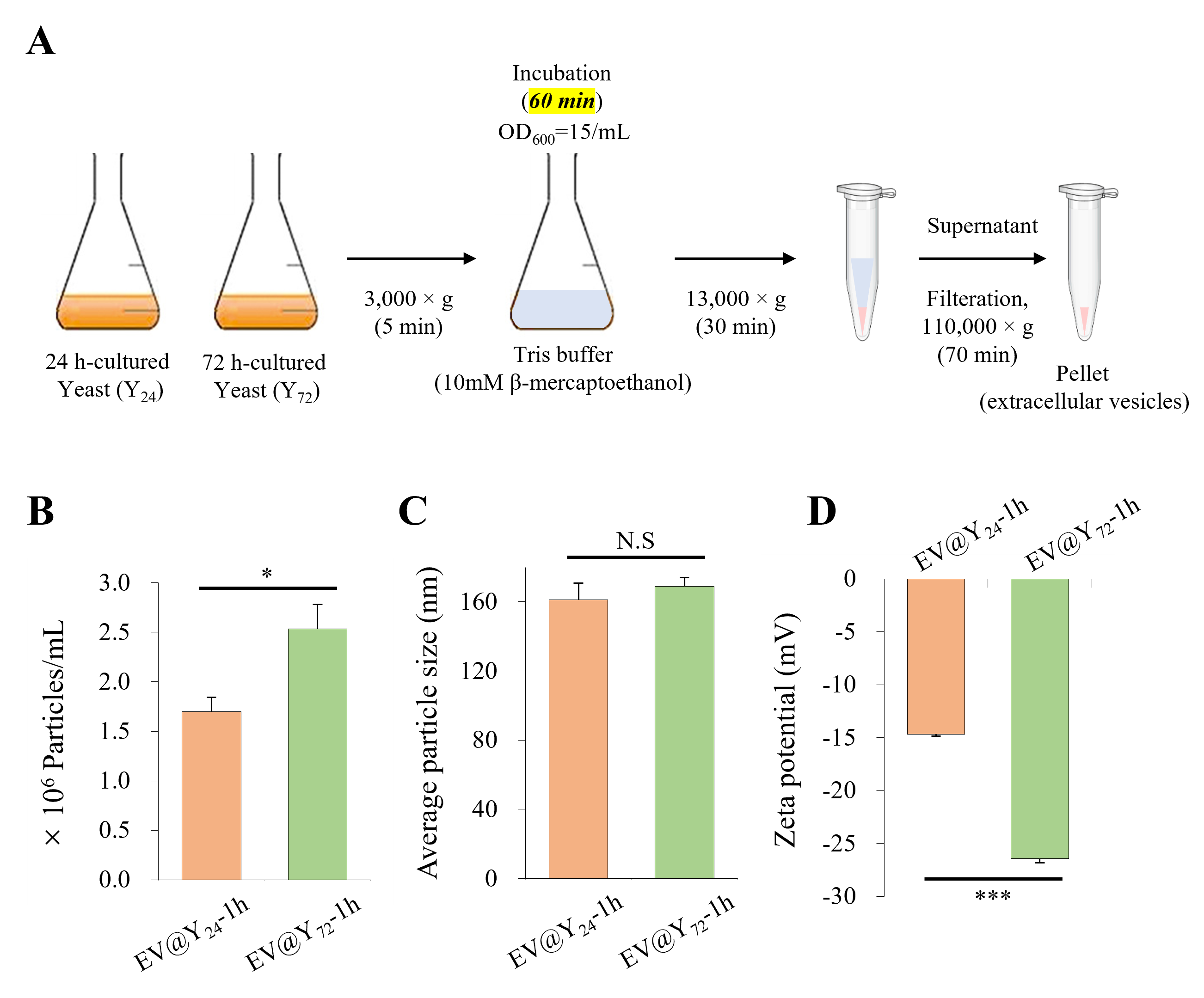

Supplement: Supplementary 1 — Figs. S1 to S4 Tables S1 to S4 [file bmr.0201.f1.zip › Figure S2.tif]

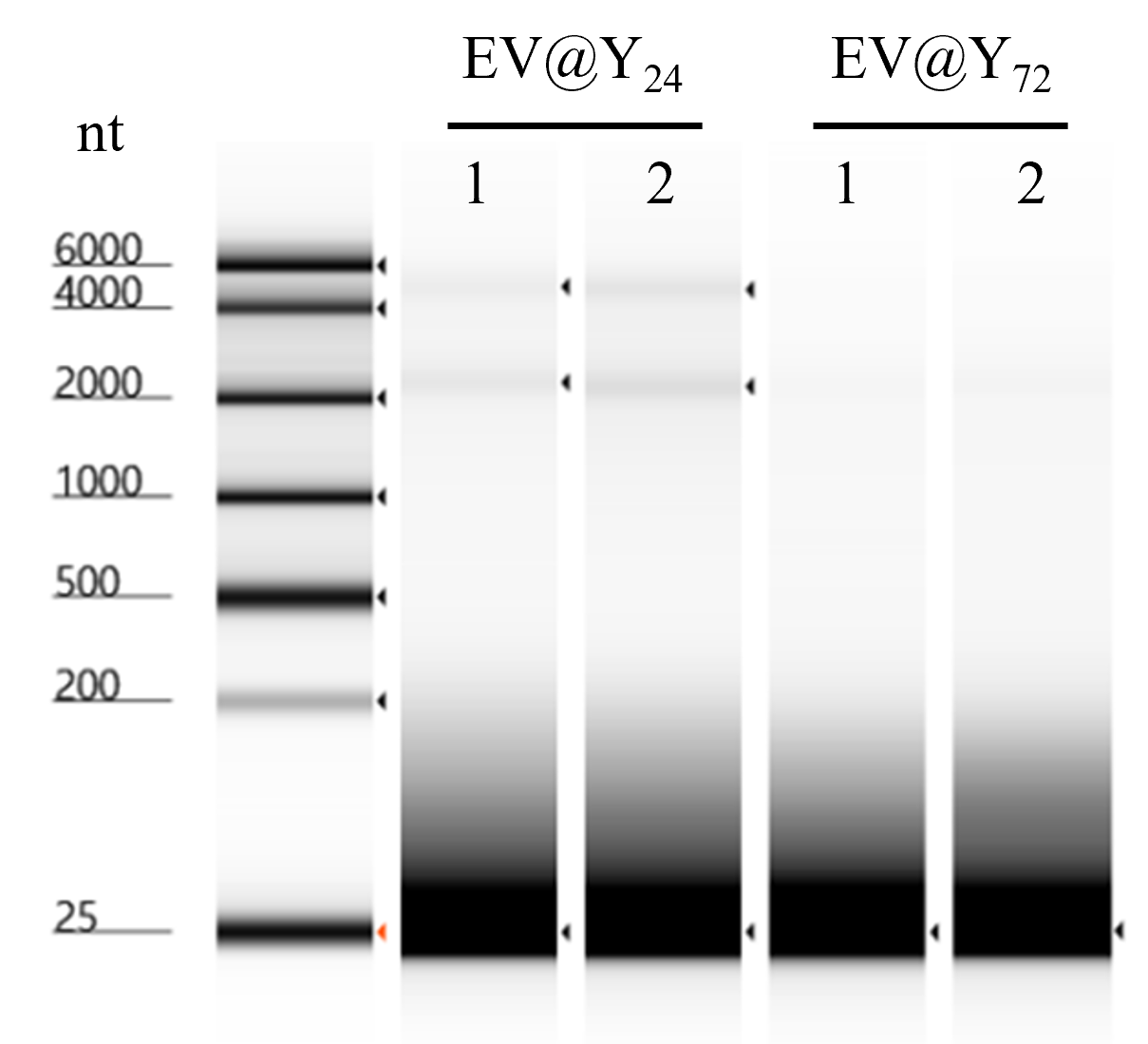

Supplement: Supplementary 1 — Figs. S1 to S4 Tables S1 to S4 [file bmr.0201.f1.zip › Figure S3.tif]

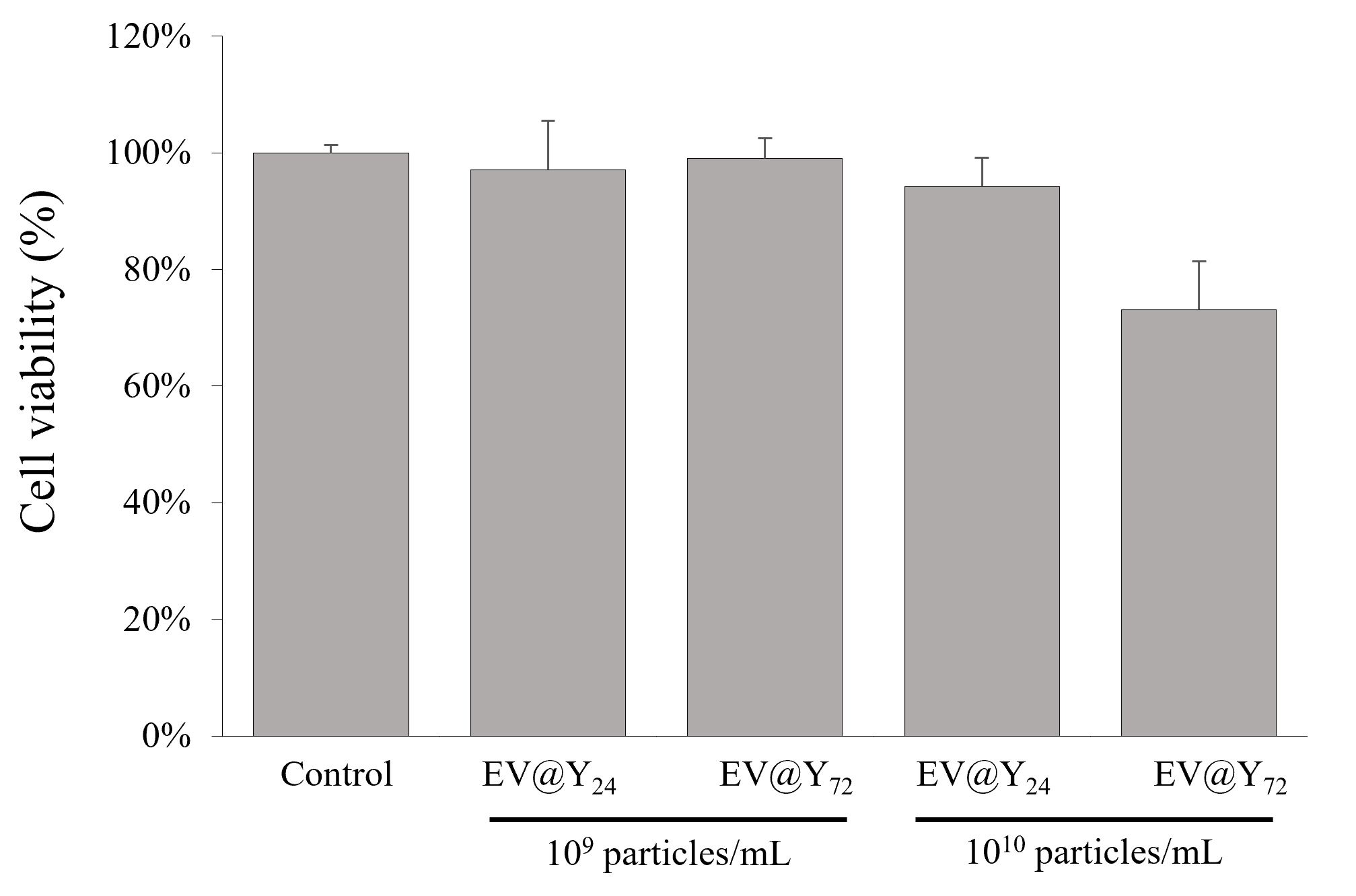

Supplement: Supplementary 1 — Figs. S1 to S4 Tables S1 to S4 [file bmr.0201.f1.zip › Figure S4.tif]
